# HGT-Finder: A New Tool for Horizontal Gene Transfer Finding and Application to *Aspergillus* genomes

**DOI:** 10.3390/toxins7104035

**Published:** 2015-10-09

**Authors:** Marcus Nguyen, Alex Ekstrom, Xueqiong Li, Yanbin Yin

**Affiliations:** 1 Department of Computer Science, Northern Illinois University, DeKalb, IL 60115-2857, USA; E-Mails: z1605032@students.niu.edu (M.N.); z1664374@students.niu.edu (A.E.); 2 Department of Biological Sciences, Northern Illinois University, Montgomery Hall 325A, DeKalb, IL 60115-2857, USA; E-Mail: xueqiongli@imau.edu.cn; 3 College of Life Sciences, Inner Mongolia Agricultural University, 306 Zhaowuda Road, Hohhot 010018, Inner Mongolia, China

**Keywords:** horizontal gene transfer, HGT, gene clusters, secondary metabolism, *Aspergillus*, bioinformatics, software

## Abstract

Horizontal gene transfer (HGT) is a fast-track mechanism that allows genetically unrelated organisms to exchange genes for rapid environmental adaptation. We developed a new phyletic distribution-based software, HGT-Finder, which implements a novel bioinformatics algorithm to calculate a horizontal transfer index and a probability value for each query gene. Applying this new tool to the *Aspergillus fumigatus*, *Aspergillus flavus*, and *Aspergillus nidulans* genomes, we found 273, 542, and 715 transferred genes (HTGs), respectively. HTGs have shorter length, higher guanine-cytosine (GC) content, and relaxed selection pressure. Metabolic process and secondary metabolism functions are significantly enriched in HTGs. Gene clustering analysis showed that 61%, 41% and 74% of HTGs in the three genomes form physically linked gene clusters (HTGCs). Overlapping manually curated, secondary metabolite gene clusters (SMGCs) with HTGCs found that 9 of the 33 *A. fumigatus* SMGCs and 31 of the 65 *A. nidulans* SMGCs share genes with HTGCs, and that HTGs are significantly enriched in SMGCs. Our genome-wide analysis thus presented very strong evidence to support the hypothesis that HGT has played a very critical role in the evolution of SMGCs. The program is freely available at http://cys.bios.niu.edu/HGTFinder/HGTFinder.tar.gz.

## 1. Introduction

Horizontal gene transfer (HGT) is a major force that shapes the genome evolution in prokaryotes, which creates genomic innovations in response to environmental adaptation [1]. HGT is biased to occur among species that are phylogenetically closely related [2] and among species sharing the same ecological environments [3]. Recently, increasing evidence has shown that HGT can take place across different life domains. For example, cellulases were found to be transferred from bacteria to nematodes [4], bacterial toxin genes were transferred into eukaryotes [5], and antibacterial lysozymes were transferred into various eukaryotes and archaea [6]. Therefore, HGT has also played a significant role in distributing genes in eukaryotes. In addition to the individual gene studies [7,8,9], large-scale, genome-wide HGT studies in eukaryotes have also been published and recently summarized in a few review articles [10,11,12,13].

Fungi are the most researched eukaryotes that have been surveyed for HGT, probably because they have the most sequenced genomes (more than 500 complete/draft genomes so far). Numerous cases of HGTs have been reported including some genome-wide detection of fungi-fungi and fungi-bacteria gene transfers [7,8,9,14,15,16,17,18,19]. One of the most interesting findings made in these studies is that genes are often transferred as physically linked gene clusters, many of which encode enzymes of the secondary/specialized metabolic pathways. For example, Rokas A. *et al.* have characterized the sterigmatocystin (ST) gene cluster (24 genes) [20], the bikaverin gene cluster (6 genes) [21], and the galactose utilization gene cluster (5 genes) [22] to be horizontally transferred between distant fungal taxa. The same lab also showed evidence that the enzymes in metabolic gene clusters are more likely to be transferred than the non-clustered enzymes [15].

The most accurate, golden-standard method to identify horizontally transferred genes (HTGs) is the gene-by-gene phylogenetic analysis, which compares the target gene phylogeny with a well-established species phylogeny to identify genes with incongruences [16,19,23]. This method has limitations though: (i) a well-established species phylogeny often does not exist, especially for non-model organisms; (ii) computing gene phylogeny on a whole genome scale is very time consuming and often complicated by gene duplications and independent gene losses. Therefore, surrogate methods have been developed, including the nucleotide composition-based method and the patchy phyletic distribution method, in order to apply to genome-scale HGT detection. The composition-based method is known to be very fast, as it does not require comparison with other genomes. It, however, suffers from low accuracy because many HTGs do not have atypical base composition and many genes with atypical compositions are not horizontally transferred [16,24,25]. The patchy phyletic distribution method has many variants, but they all process the sequence similarity search result to investigate the taxonomic closeness of the top matches. The simplest variant asks the question: does my gene of X have its best hit in Y, where X and Y are two distant taxa? The phyletic distribution method is often used as the first step in conjunction with the phylogeny-based method to pre-scan a large number of genes in order to narrow down to a small number of genes for detailed phylogenetic analysis. There have been at least four computer softwares published implementing the phyletic distribution method: Pyphy [26], PhyloGenie [27], DarkHorse [28], and HGTector [29]. All these tools were originally designed to find HTGs in prokaryotes, do not have a rigorous statistical assessment of the predictions, and require extensive human intervention.

Here we developed a new phyletic distribution-based bioinformatics software, ***HGT-Finder***, for HGT detection in fungal genomes. Compared to previous tools, HGT-Finder: (i) can be used for HGT detection in both prokaryotes and eukaryotes, (ii) can report a statistical *P* value for each gene to indicate how likely it is to be horizontally transferred, and (iii) is fully automated (requires minimal human intervention), as well as very easy to install and run. At the core of our method is a mathematical function that considers not only the sequence similarity between the query and its top hits, but also a newly defined taxonomic distance between the query species and the hit species. By design, it can identify HTG candidates from a distant species. We have applied this new tool to three *Aspergillus* model genomes and focused on presenting the technical details and uses of this new tool. We also looked at the results of the genome-wide analysis of HTGs in terms of their functions, sequence features, and gene clustering. Additionally, we also compared HGT-Finder predictions with previously published HGT results and tools.

## 2. Results and Discussion

### 2.1. HGT-Finder: A New Tool to Find Horizontal Gene Transfer

The algorithm behind HGT-Finder is provided in the **Methods** section. The inputs to this software include: (i) the BLAST search result (tabular format-outfmt 6) of a query set (e.g., proteins of a genome) against the NCBI nonredundant protein (NCBI-nr) database and (ii) the NCBI Taxonomy database. The output of this program is a tabular format file containing the following key information: protein ID, *X* value (transfer index value), *P* value and *Q* value. *X* is calculated using a mathematical formula detailed in Methods. In brief, for each pair of query and BLAST subject species, a novel taxonomic distance *D* is calculated such that *D* ∈ [0, 1], and a BLAST similarity measure *R* (BLAST bit score ratio relative to the self-hit, see Methods) is calculated such that *R* ∈ [0, 1]. The *X* for each query considers *D* and *R* values of all of its BLAST subjects. The *P* value is calculated according to the statistical distribution of the *X* for all query proteins.

**Figure 1:**
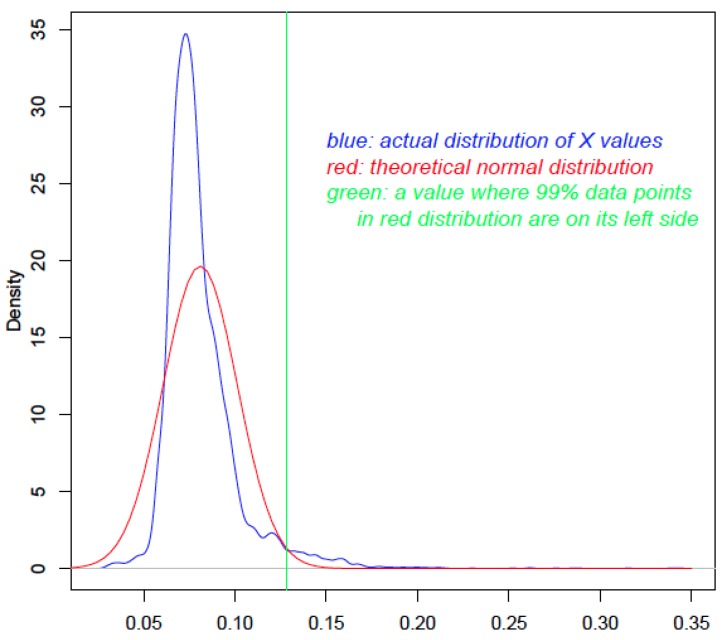
The use of statistical distribution to calculate *P* values. The *x*-axis shows the *X* value. The blue curve is the distribution of the *X* values of 9577 Aspfu proteins. The red curve is the theoretical distribution that has the same mean and standard deviation as the blue curve. The green line is drawn to indicate the cutoff value; any *X* value larger than that in the blue curve will have a *P* value <0.01.

More specifically, the *X* values for all query proteins are plotted (blue curve in Figure 1). The mean and standard deviation values are calculated, which are used to generate a theoretical normal distribution (red curve in Figure 1). The actual distribution and the theoretical normal distribution are then compared to calculate a probability value for each query protein using the *pnorm* function of the R software (R Development Core Team) (www.r-project.org). The *P* value is used to reject the null hypothesis that the to-be-tested value from the actual distribution is smaller than a particular value in the normal distribution (green vertical line in Figure 1). Thus, proteins with a higher *X* will typically have smaller *P* values and are more likely to be true HTGs. Since the number of statistical tests to be done is equal to the number of genes in the query set, there will be multiple testing errors that are to be corrected [30]. The qvalue package of the Bioconductor software (https://www.bioconductor.org) is used to convert the *P* value to a corrected *Q* value, which is a more accurate metric to determine statistical significance.

### 2.2. Use Different R Thresholds to Detect Horizontally Transferred Genes (HTGs)

**Figure 2:**
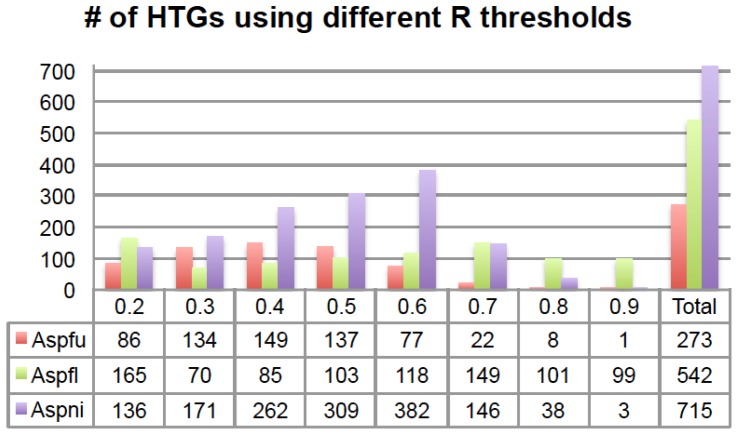
The number of HTGs predicted using different R thresholds. The *x*-axis is the R threshold and the *y*-axis is the number of HTGs. The last column shows the total number of HTGs after removing overlaps. # means “number”.

One very important parameter in running HGT-Finder is the R threshold (see Methods), which is used, prior to the calculation of *X*, to remove BLAST hits that are less similar to the query. For example, one can use *R* > 0.2, meaning that only hits with *R* > 0.2 will be used for the *X* calculation. In order to study the impact of this R threshold on HGT predictions, we have run HGT-Finder using *Q* value <0.01 and a range of R thresholds from 0.2 to 0.9 on the *Aspergillus fumigatus* Af293 (**Aspfu**), *Aspergillus flavus* NRRL3357 (**Aspfl**), and *Aspergillus nidulans* FGSC A4 (**Aspni**) protein sets to predict HTGs. Hence we obtained eight HTGs sets for each species (Figure 2).

Some HTGs were predicted with multiple R thresholds. For example, in total 273 Aspfu proteins were found in at least one of the eight sets and 47 of the 273 were found in at least four of the eight sets (Table S1); among those 47 genes, 45 of them were found in the *R* > 0.5 set. Similarly, for Aspfl in total 542 proteins were found in at least one of the eight sets and 49 were found in at least four of the eight sets (Table S1); among those 49 genes, 43 of them were found in the *R* > 0.5 set. For Aspni, a total of 715 proteins were found in at least one of the eight sets and 101 were found in at least four of the eight sets (Table S1); among those 101 genes, 100 of them were found in the *R* > 0.5 set. Therefore, for all the three genomes, it is always the *R* > 0.5 set that contains the most genes that are shared by the other R threshold sets.

However, using a single R threshold will certainly result in a loss of many HTGs. The higher the R threshold that is used, the fewer BLAST hits that will be considered in the *X* calculation. For example, if *R* > 0.5 is used, BLAST hits with an *R* less than 0.5 will be removed prior to calculating *X*. Moreover, a higher R threshold will result in more query proteins that will fail to have an *X* calculation. For example, if *R* > 0.9 is used, those query proteins that do not have very similar hits in the database will not have an *X* value calculated. This explains why, in Figure 2, there are fewer HTGs predicted for *R* > 0.8 and *R* > 0.9 sets for all three genomes.

**Figure 3:**
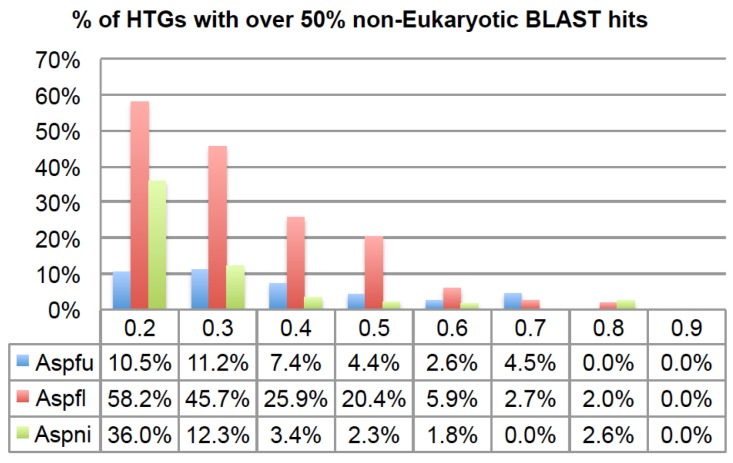
The percentage of HTGs that have more than 50% BLAST hits from non-eukaryotic species using different R thresholds. The *x*-axis is the R thresholds and the *y*-axis is the percentage of HTGs.

Lastly, a lower R threshold tends to predict more ancient HGTs while a higher R threshold tends to predict more recent HGTs. To verify this, using different R thresholds, we calculated the percentage of HTGs having over 50% of BLAST hits from non-Eukaryotes (*i.e.*, Bacteria, Archaea and Viruses). Figure 3 shows that, for all the three genomes, there is a clear trend that when using lower R thresholds, a higher percentage of HTGs are found to have more than 50% of their BLAST hits from different domains of life. This indicates that they might be derived from more ancient HGTs when assuming very recent inter-domain transfers (*i.e.*, with high *R*) are rare. If an HTG has more inter-domain BLAST hits, it is more likely to be an ancient HTG. On the other hand, if an HTG has all of its BLAST hits within the same taxonomic group, e.g., phylum, it is a more recent HTG. Therefore, by default, our HGT-Finder program runs all eight R thresholds and the users are advised to combine the HTGs from all these eight runs to obtain a complete list of HTGs.

Figure 2 and Figure 3 also show that although Aspni has more HTGs than the other genomes, Aspfl has a higher percentage of inter-domain HTGs, which agrees with another recent report [14].

### 2.3. Verify HTGs Using an Approximate Method and Phylogenetic Analysis

**Figure 4:**
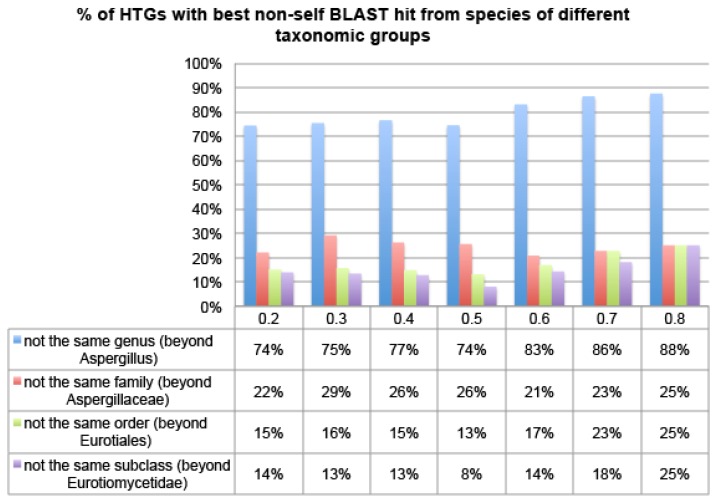
The percentage of Aspfu HTGs that have more than 50% BLAST hits from non-Eukaryotic species using different R thresholds. The *x*-axis is the R threshold and the *y*-axis is the percentage of HTGs.

In order to quickly confirm the HGT predictions, we have examined the non-self best hit of the HTG candidates, where “non-self” means that the BLAST subject protein is from a species with a different taxonomy ID. The complete data for *R* > 0.5 for the three genomes are available in Table S2. The NCBI-nr database contains protein sequences from 15 sequenced nuclear genomes of the *Aspergillus* genus (Table S3). If an HTG candidate were transferred from outside of the genus, then its top BLAST hits (here we use the best hit for simplicity) would be from a different genus, family, order, class, phylum or kingdom with an increasing evolutionary distance to the recipient. Figure 4 shows that over 74% of HTG candidates of Aspfu have their best non-self hit from species of different genera, over 20% from even different families, and over 15% from even different orders, irrespective of which R thresholds were used. This pattern is even more pronounced in the other two genomes, Aspfl and Aspni (Figures S1 and S2). This suggests that HGT-Finder does succeed in making meaningful predictions.

This simple and approximate method, although very informative, fast, and easy to execute, cannot conclusively verify HTGs because a best hit from a distant organism could also be due to other reasons such as: (i) the subject gene may be recently transferred from (not to) the query genome; (ii) the query gene evolved very rapidly so it becomes very different from its orthologous genes in closely related species; (iii) the orthologous genes in closely related species were independently lost during evolution.

**Figure 5:**
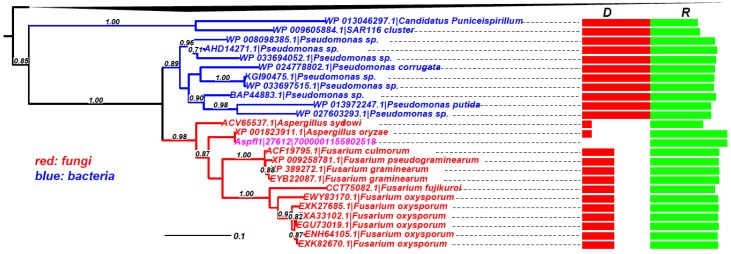
Phylogeny of Aspfl1|27612|7000001155802518 (GenBank ID: AFLA_091530) as an example of HTG. The query protein (in pink)’s homologs are restricted to very few fungal species, two other *Aspergillus* species and a few Fusarium species. The rest of the homologs are all from Proteobacteria. The boxes on the right show the taxonomic distance (D) in green and sequence similarity (R) in red. The black triangle on the top represents the collapsed *Proteobacteria* homologs.

As mentioned above, the gene-by-gene phylogenetic analysis, although not computationally suitable for large-scale analysis, is the golden standard method to claim a gene is a HTG. We have performed phylogenetic analyses on the *R* > 0.5 set for Aspfl. In total, there are 103 HTGs predicted in Aspfl by HGT-Finder (Figure 2), 73 of which have at least four BLAST hits in other taxa and thus applicable for building phylogenies. Figure 5 shows an example (Aspfl1|27612, Peptidase M24) phylogeny, which clearly indicates that the common ancestor of the fungal proteins, including the query protein, must have been transferred from some *Pseudomonas* bacteria (*Gammaproteobacteria*). Phylogenies of other genes are combined and made available in Supplemental data file 1. We have manually inspected all these phylogenies containing protein hits with *R* > 0.2 in order to determine if they are true HTGs. Among these 73 genes, phylogenies seem to support 68 (93%) of them to be HTGs (Table S3), which include: (i) 30 that have hits in smaller numbers of fungi but many bacteria, suggesting transfers from bacteria; (ii) 38 have hits in very few *Aspergillus* genomes (mostly restricted to Aspfl and the very closely related *A. oryzae*) and are phylogenetically clustered with hits of different fungal genera, or even more distant taxonomic groups, suggesting transfers from distant fungi. The remaining five genes do not have a strong phylogenetic signal to suggest that they are HTGs. One of the five is a very conserved ribosomal protein (jgi|Aspfl1|30709), which is restricted to Aspfl and *A. oryzae* of the *Aspergillus* genus, and further clustered with a termite (*Coptotermes formosanus*) protein, suggesting a recent gene transfer into termite (Supplemental data file 1).

It should be noted that some of the 38 fungi-fungi HTG candidates might have very complex evolutionary trajectories. For example, jgi|Aspfl1|31710 (Supplemental data file 1) might have been recently transferred from other fungi (very few *Aspergillus* hits); furthermore, all of the fungi hits might have been transferred from bacteria in an earlier event (not many fungi hits but numerous bacteria hits). Compared to bacteria-fungi transfers, fungi-fungi transfers are more difficult to detect, because patchy phyletic distribution of BLAST hits could also be a result of independent gene loss occurred in closely related species [31]. Even when the phylogeny is available, to reliably distinguish the two possibilities (gene transfer and gene loss) is still not easy, which is complicated by the incompleteness and biased sequence sampling of the BLAST database. It would therefore be safer to conclude that the 38 fungi-fungi HTG candidates have been confirmed to have patchy taxonomic distribution based on phylogenetic analyses. Nevertheless, they have a higher likelihood to be horizontally transferred because independently losing these genes in most of the closely related *Aspergillus* genomes (Table S3) is a less parsimonious explanation than the HGT hypothesis.

We have also manually examined the 30 HTGs that have less than three hits (with *R* > 0.5) by inspecting the BLAST output with *R* > 0.2 and searching them using NCBI’s Blink service. When relaxing the R threshold to 0.2, most of the 30 HTGs have more hits. We found that: (i) five of the 30 genes must have been transferred from bacteria, (ii) 17 might have been transferred from distant fungi more recently, (iii) three have complex evolution involving possible recent transfers from distant fungi and more ancient transfers from bacteria, (iv) two might have involved *Metazoa* in the transfer, and (v) the remaining three seem to be orphan genes. We have made comments on all the 103 Aspfl HTGs in Table S3 based on our manual curation. Such detailed phylogenetic analyses suggest that our HGT-Finder program indeed performs well in identifying true HTGs. It should be noted that we have used a very stringent *Q* value < 0.01 as the cutoff to keep statistically significant candidates. The number of HTGs may thus have been underestimated.

### 2.4. Test the Performance of HGT-Finder Using Simulated Data

The above phylogenetic verification suggests that HGT-Finder has a fairly high specificity (93% for the case of 73 Aspfl proteins). We have created simulated data to test the sensitivity of HGT-Finder using the procedure as follows. We randomly selected 100 *Escherichia coli* MG1655 (prokaryote) proteins and merged them with the 12,604 Aspfl proteins for HGT detection. The idea is that if we pretend that these 100 *E. coli* proteins were Aspfl proteins, how many of them could be correctly identified as HTGs in the Aspfl simulated dataset? We have also repeated the same procedure with 100 randomly selected *Fusarium fujikuroi* (Fusfu, a fungus of a different taxonomic class than *Aspergillus*) proteins. Table 1 shows that, using *Q* value < 0.01 as the statistical cutoff, HGT-Finder has a sensitivity = 95% for *E. coli* using *R* > 0.6 and sensitivity = 92% for Fusfu using *R* > 0.7, which are also the overall sensitivity values (combining predictions from all R thresholds) for the two simulated datasets.

**Table 1:** The number (out of 100) of HTGs identified in the two simulated datasets using different R thresholds. # means “number”.

R Threshold	# of *E. coli* Proteins Found to be HTGs	# of Fusfu Proteins Found to be HTGs
2	42	49
3	55	55
4	70	65
5	81	77
6	95	88
7	0	92
8	0	0
9	0	0
All	95	92

### 2.5. Function of HGT Genes

Previous studies have suggested that metabolic enzymes are prone to be horizontally transferred [10,32,33], which has never been tested using strict statistical approaches in fungi. In brief, *h* is the number of genes with a certain function in the HTG set and *H* is the total number of HTGs; this *h*/*H* ratio has to be compared to the genome background ratio *t*/*T*, where *t* is the number of genes with that function in the genome and *T* is the total number of genes in the genome. We have performed hypergeometric enrichment tests on the Gene Ontology (GO) annotations of 273 Aspfu, 542 Aspfl, and 715 Aspni HTGs (numbers from Figure 2) by comparing them with the genome background. Table 2, Table 3 and Table 4 (complete datasets are in Table S4) list the top GO functions in the three genomes that have at least 10 assigned HTGs.

**Table 2:** GO functional categories having at least 10 assigned HTGs in Aspfu. # means “number”.

GO Name	GO ID	# of Assigned HTGs	# of Assigned Genes in the Genome	*P* Value (Red Font if <0.05)
un-annotated by GO	-	141	4008	0.0001964405
metabolic process	GO:0008152	31	893	0.1191704
catalytic activity	GO:0003824	28	883	0.2647851
binding	GO:0005488	17	538	0.3332352
ribonuclease H activity	GO:0004523	16	19	7.89 × 10^−23^
nucleic acid binding	GO:0003676	16	401	0.09561368
oxidoreductase activity	GO:0016491	16	522	0.3859507
RNA-dependent DNA replication	GO:0006278	14	23	8.17 × 10^−17^
RNA-directed DNA polymerase activity	GO:0003964	14	23	8.17 × 10^−17^
RNA binding	GO:0003723	14	102	8.23 × 10^−7^
integral to membrane	GO:0016021	13	516	0.6891403
transport	GO:0006810	12	467	0.6582828
carbohydrate metabolic process	GO:0005975	11	225	0.05062836
transporter activity	GO:0005215	11	292	0.1936068
membrane	GO:0016020	11	431	0.6636644
hydrolase activity, hydrolyzing *O*-glycosyl compounds	GO:0004553	10	141	0.006067584

**Table 3:** GO functional categories having at least 10 assigned HTGs in Aspfl.

GO Name	GO ID	# of Assigned HTGs	# of Assigned Genes in the Genome	*P* Value (Red Font if <0.05)
Un-annotated by GO	-	279	5513	3.32823 × 10^−6^
catalytic activity	GO:0003824	74	1309	0.00360352
metabolic process	GO:0008152	73	1304	0.004876894
oxidoreductase activity	GO:0016491	40	820	0.1634237
binding	GO:0005488	39	788	0.1454589
electron transport	GO:0006118	23	494	0.3167226
ATP binding	GO:0005524	19	633	0.9504606
transport	GO:0006810	18	684	0.9885602
integral to membrane	GO:0016021	16	700	0.997863
membrane	GO:0016020	15	553	0.9738793
hydrolase activity	GO:0016787	13	208	0.09374698
iron ion binding	GO:0005506	13	232	0.1696784
transporter activity	GO:0005215	13	476	0.9629008
proteolysis	GO:0006508	12	227	0.2362409
nucleus	GO:0005634	12	631	0.9995651
pyridoxal phosphate binding	GO:0030170	11	100	0.002880844
biosynthetic process	GO:0009058	10	94	0.005585243
DNA binding	GO:0003677	10	435	0.9879336

**Table 4:** GO functional categories having at least 10 assigned HTGs in Aspni.

GO Name	GO ID	# of Assigned HTGs	# of Assigned Genes in the Genome	*P* Value (Red Font if <0.05)
Un-annotated by GO	-	242	4367	0.9999737
metabolic process	GO:0008152	147	1058	6.12 × 10^−19^
catalytic activity	GO:0003824	136	1046	4.77 × 10^−15^
oxidoreductase activity	GO:0016491	106	668	1.06 × 10^−17^
binding	GO:0005488	84	633	6.18 × 10^−10^
electron transport	GO:0006118	73	411	5.94 × 10^−15^
transport	GO:0006810	72	560	4.38 × 10^−8^
integral to membrane	GO:0016021	67	573	4.20 × 10^−6^
transporter activity	GO:0005215	60	382	3.42 × 10^−10^
heme binding	GO:0020037	43	183	1.85 × 10^−13^
membrane	GO:0016020	43	470	0.02210711
monooxygenase activity	GO:0004497	39	165	2.07 × 10^−12^
iron ion binding	GO:0005506	37	175	2.47 × 10^−10^
nucleus	GO:0005634	32	671	0.987011
carbohydrate metabolic process	GO:0005975	25	215	0.004820523
DNA binding	GO:0003677	23	449	0.9321919
*L*-arabinose isomerase activity	GO:0008733	22	97	3.05 × 10^−7^
hydrolase activity	GO:0016787	22	172	0.002504542
zinc ion binding	GO:0008270	22	696	0.9999923
carbohydrate transport	GO:0008643	18	92	3.16 × 10^−5^
sugar:hydrogen symporter activity	GO:0005351	18	94	4.27 × 10^−5^
hydrolase activity, hydrolyzing *O*-glycosyl compounds	GO:0004553	16	132	0.01493863
cofactor binding	GO:0048037	15	71	5.65 × 10^−5^
FAD binding	GO:0050660	15	138	0.04270059
transcription factor activity	GO:0003700	15	409	0.9974484
regulation of transcription, DNA-dependent	GO:0006355	15	456	0.999605
proteolysis	GO:0006508	14	184	0.3491031
phosphopantetheine binding	GO:0031177	13	50	1.71 × 10^−5^
unspecific monooxygenase activity	GO:0050381	12	44	2.13 × 10^−5^
nucleic acid binding	GO:0003676	12	394	0.9996286
F420H2 dehydrogenase activity	GO:0043738	11	47	0.000211107
malolactic enzyme activity	GO:0043883	11	47	0.000211107
regulation of oxidoreductase activity	GO:0051341	11	47	0.000211107
sulfur oxygenase reductase activity	GO:0043826	11	47	0.000211107
DNA integration	GO:0015074	10	26	3.37 × 10^−6^
peroxidase activity	GO:0004601	10	29	1.06 × 10^−5^
aromatic compound metabolic process	GO:0006725	10	66	0.01182745
ATP binding	GO:0005524	10	554	1

Not all genes could be annotated by GO (only 56% Aspfl, 59% Aspfu and 59% Aspni have GO annotations), which are listed by the “un-annotated by GO” category in the first line of each table. The tables show that un-annotated genes are enriched in the HTG sets for Aspfu and Aspfl, but not in the HTG set for Aspni. “Metabolic process” and “catalytic activity,” the two high-level GO categories that involve most enzymes in the genome, are enriched in the HTG sets for Aspfl and Aspni, but not in the HTG set for Aspfu. Compared to the other two genomes, Aspfu has four unique GO categories: “ribonuclease H activity,” “RNA-dependent DNA replication,” “RNA-directed DNA polymerase activity,” and “RNA binding” that have the lowest *P* values (most enriched). These four categories are very much redundant with each other sharing 14 HTGs. A keyword search of these 14 genes at NCBI found that these genes were annotated as “reverse transcriptase, RNaseH.” They are now labeled as “discontinued.” This is probably because they were contaminants or mistakenly predicted genes originally submitted by the data producer, but were later removed by NCBI. Since the genome data used in this paper was downloaded from JGI, these genes were included in our analyses. In Aspni, most of the top GO categories are enriched in HTGs, which is not surprising because Aspni has a higher percentage of HTGs (6.7%) than Aspfl (4.2%) and Aspfu (2.8%).

We have performed the similar hypergeometric enrichment tests on the KEGG (Kyoto Encyclopedia of Genes and Genomes pathway) and KOG (Eukaryotic Orthologous Groups of proteins) annotations for the HTGs by comparing them with the genome background. The results also showed that more functional categories of KEGG and KOG are enriched in HTGs of Aspni than Aspfu and Aspfl. Interestingly, the “Biosynthesis of Secondary Metabolites” category is enriched in HTGs of Aspni (*P* value = 0.002) and Aspfu (*P* value = 0.04). In Aspni, “Carbohydrate Metabolism” (*P* value = 0.0007), “Lipid Metabolism” (*P* value = 0.002), and “Metabolism of Other Amino Acids” (*P* value = 0.04) are all enriched in HTGs.

### 2.6. Sequence Properties of HTGs: Guanine Cytosine, Length, K_a_, K_s_

In bacteria, HTGs were shown to have a lower GC content and more relaxed selection [34,35,36]. In Table 5, we have compared the sequence properties of HTGs and non-HTGs in the three fungi. We found that, in all three fungi, HTGs have significantly shorter length, higher GC content at the third position of codons (GC3), higher *K*_a_ (the number of nonsynonymous substitutions per non-synonymous site), higher *K*_s_ (the number of synonymous substitutions per synonymous site), and higher *K*_a_/*K*_s_ ratio. We used GC at the third position of codons because the third position is more freely changeable and less affected by translational selection than the other two positions. In bacteria, the lower GC content of HTGs might be related to the suppression of gene expression of HTGs [37]. Hence, it is surprising that, in opposition to what is found in bacteria, fungi HTGs have higher GC content than non-HTGs. The shorter length of HTGs might be due to the simpler protein domain architectures [38] in HTGs.

**Table 5:** Sequence properties of HTGs *vs.* non-HTGs in the three genomes.

In Parentheses are Hypotheses Supported by the Wilcoxon Rank Tests		Aspfu	Aspfl	Aspni
Length median/mean	HTG	861/1143	620/824	1170/1312
	non-HTG	1257/1487	1185/1408	1239/1466
	*P* value (shorter in HTG)	<2.2 × 10^−16^	<2.2 × 10^−16^	4.32 × 10^−6^
GC3 median/mean	HTG	0.64/0.63	0.57/0.57	0.62/0.61
	non-HTG	0.59/0.60	0.56/0.57	0.58/0.59
	*P* value (higher in HTG)	2.29 × 10^−7^	0.003305	<2.2 × 10^−16^
K_a_ median/mean	HTG	0.04/0.11	0.41/0.42	2.21/2.27
	non-HTG	0.02/0.06	0.20/0.28	1.53/1.66
	*P* value (higher in HTG)	5.10 × 10^−15^	6.684 × 10^−13^	<2.2 × 10^−16^
K_s_ median/mean	HTG	0.17/0.48	1.86/2.09	0.62/0.58
	non-HTG	0.12/0.22	1.72/1.83	0.19/0.27
	*P* value (higher in HTG)	<2.2 × 10^−16^	0.01375	<2.2 × 10^−16^
K_a_/K_s_ median/mean	HTG	0.23/0.30	0.25/0.35	0.26/0.27
	non-HTG	0.20/0.24	0.12/0.16	0.13/0.15
	*P* value (higher in HTG)	0.0004822	3.264 × 10^−13^	<2.2 × 10^−16^

*K*_a_ measures the nucleotide substitutions that cause amino acid changes, which are under very strong selection pressure, while *K*_s_ measures the nucleotide substitutions that do not lead to amino acid changes, which are more neutral to selection. The *K*_a_/*K*_s_ ratio is widely used as a proxy to evaluate the intensity of selection. For most genes, this ratio should be close to 0 due to purifying selection (most nucleotide substitutions in the coding regions do not change the protein products). For genes that are newly incorporated into the host genome, it is not surprising that, in order to explore the new environment and network, they are allowed to have more freedom to change, in sequence, under a more relaxed selection pressure.

### 2.7. Horizontally Transferred Gene Clusters (HTGCs)

**Figure 6:**
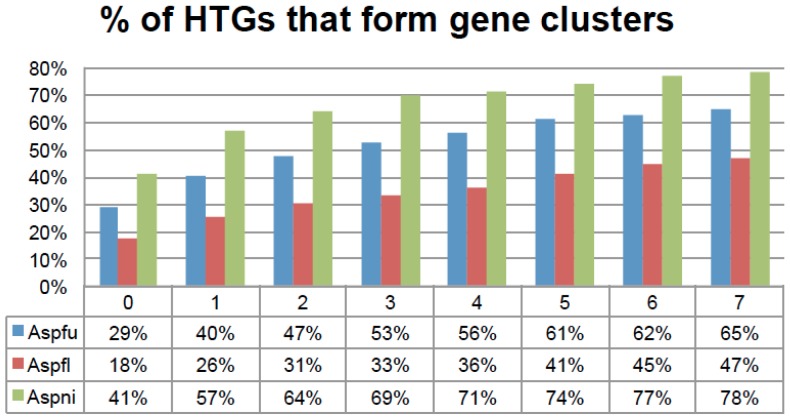
The percentage of HTGs that form physically linked gene clusters on chromosomes. The *x*-axis is the *N* thresholds and the *y*-axis is the percentage of HTGs. *N* is used to define gene clusters. For example if two HTGs are separated by less than *N* non-HTGs, these *N* + 2 genes will belong to one gene cluster. More HTGs will be included until the N threshold is not met. *N* is explored from 0 to 7 in this figure.

The evolution of metabolic gene clusters (MGCs), especially those involved in secondary metabolism, are affected by HGT [15]. A dozen HGT cases in MGCs have been summarized in a recent review [39]. We have implemented a program in the HGT-Finder software to examine the genomic locations of HTGs and further derive horizontally transferred gene clusters (HTGCs). We defined an HTGC as a group of physically linked genes containing at least two HTGs separated by less than *N* non-HTGs, where *N* was explored from 0 to 7 (Figure 6 and Table S5). We have also tried to add another restriction: the base pair distance between two adjacent genes in the HTGC should be less than 10 kb, which appeared to have little effect on the results (Table S5).

For *N* = 5, the 273 Aspfu, 542 Aspfl, and 715 Aspni HTGs yielded 57, 84, and 129 HTGCs, respectively, which encompass 326, 421, and 1034 genes in total including 167 (61%), 215 (41%), and 530 (74%) HTGs. A permutation experiment that randomly selected (100 times) the same amount of genes from the genome and then ran our gene clustering program suggested that such gene clustering of HTGs is not random but statistically significant (*P* value = 4.1 × 10^−246^ for Aspfu, *P* value = 0.01 for Aspfl, and *P* value = 3.7 × 10^−23^ for Aspni when *N* = 5). This is an indication that these HTGs have a very strong tendency to form physically linked gene clusters.

### 2.8. Overlap between Horizontally Transferred Gene Clusters (HTGCs) and Secondary Metabolism Gene Clusters

We went further to investigate how our predicted HTGCs overlap with the secondary metabolism gene clusters (SMGCs). We obtained a list of manually curated SMGCs for Aspfu (251 genes of 33 clusters) and Aspni (458 genes of 65 clusters) from [40]. Comparing these genes with the HTGs in the HTGCs of the two genomes, we found that: (i) in Aspni, 98 of the 458 SMGs are HTGs (Table S6), a hypergeometric test returned a *P* value = 8.6 × 10^−27^, suggesting SMGs are very much enriched in the HTG set; and similarly (ii) in Aspfu, 22 of the 251 SMGs are HTGs (Table S6) with a hypergeometric test *P* value = 1.9 × 10^−6^, also supporting that SMGs are enriched in the HTG set.

**Figure 7:**
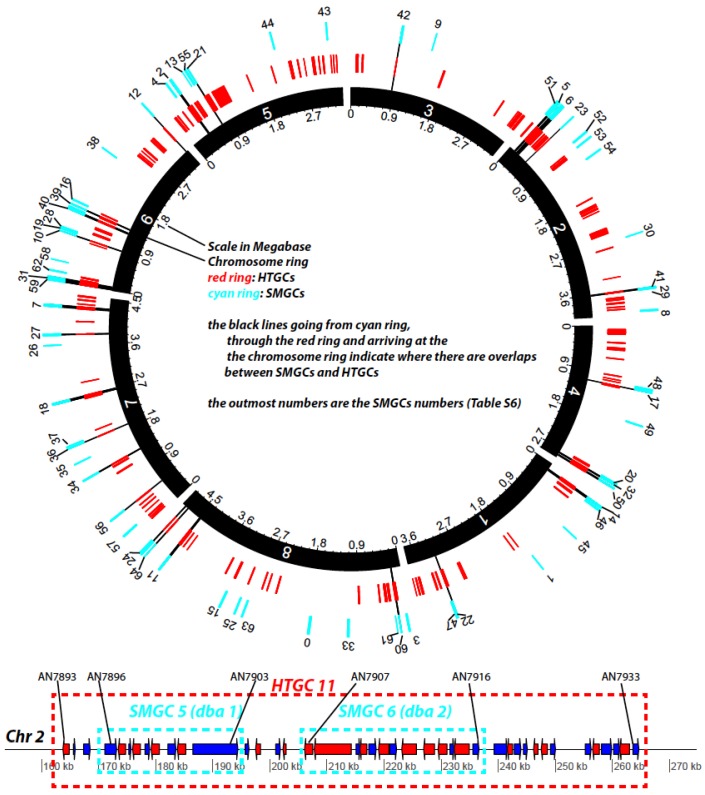
Diagram representation of HTGCs and SMGCs in Aspni. The top graph is a Circos plot [42] of the chromosomal distribution of HTGCs and SMGCs in Aspni. The outmost numbers are the IDs of SMGCs, which were extracted from [40]. The functional descriptions of these SMGCs are available in Table S6. The bottom linear graph, as an example of overlapping between HTGCs and SMGCs, shows the detailed genomic neighborhood of SMGC 5 and 6 (cyan frames) as well as the overlapping HTGC 11 (red frame).

Figure 7 provides an overall representation of the 65 SMGCs (cyan ring) and 129 HTGCs (red ring) on the Aspni chromosomes. The bottom of the figure shows an example of two adjacent SMGCs, Derivative of Benzaldehyde1 (dba) and F9775 hybrid cluster 1 (named dba 1 in the figure, nine genes) and Derivative of Benzaldehyde1 (dba) and F9775 hybrid cluster 2 (named dba 2, 10 genes), being enclosed by the large HTGC 11 (42 genes). All nine genes in dba 1 are HTGs and eight of the 10 genes in dba 2 are HTGs. The detailed information about the component genes is provided in Table S6. A hypothesis was proposed 15 years ago that HGTs play a significant role in the evolution of SMCs in fungi [41]. Our genome-wide analysis presented here provides very strong evidence to support this hypothesis.

### 2.9. Comparisons with Published Results and Tools

In Section 2.3, we showed that most predictions made by HGT-Finder are supported by phylogenetic analysis. One question remains: how do HGT-Finder predictions compare with published results? We have compared the predictions of HGT-Finder with published HTG sets for the three *Aspergillus* genomes.

Aspfu has been surveyed previously for HGTs using a composition-based method [17] where 214 genes were reported to be horizontally transferred. Aspfu, Aspfl, and Aspni have also been studied for prokaryotes-fungi gene transfers using a phyletic distribution method followed by phylogenetic analysis [14]; 20 Aspfu, 45 Aspfl, and 14 Aspni genes were found to be HTGs from prokaryotes (named TIG2010 set here). For Aspfu, we have compared our 273 HTG set with the composition-based 214 HTG set, and found that 16 (7.5% of 214) HTGs were shared by both sets. This is not surprising because it is known that composition-based methods tend to identify different HTGs compared to other methods [16,24,25]. We have also compared our HGT-Finder sets (273 Aspfu, 542 Aspfl, and 715 Aspni) against the TIG2010 sets. We found that six (30% of 20) Aspfu, 12 (26.7% of 45) Aspfl, and four (28.6% of 14) Aspni were shared between the HGT-Finder and the TIG2010 sets. These percentages suggest that HGT-Finder might have missed many prokaryotes-fungi HTGs. Another explanation is that, with new genome data added to the database, many HTGs found in TIG2010 now turned out to be non-HTGs. It should be noted that TIG2010 just focused on prokaryotes-fungi gene transfers and our HGT-Finder can find transfers from all kinds of organisms.

We have further compared HGT-Finder with DarkHorse [28], one of the four published phyletic distribution-based softwares. DarkHorse was selected for comparison because it was a relatively recent development and the easiest to install and run based on our own experience. Other tools are either very difficult to install or require extensive human intervention to run. DarkHorse ranked genes based on a “lineage probability index” (*LPI*) that has a range between 0 and 1. Although it does not provide a statistical distribution-based probability value for each gene, according to its tutorial, an empirical LPI score <0.6 is recommended to be a safe cutoff to call HTGs. After running DarkHorse on the three fungal genomes with an LPI threshold of *LPI* < 0.6 and default parameters, we found 231 Aspfl and 397 Aspni HTGs, but only three Aspfu HTGs. Overlapping these DarkHorse sets with our HGT-Finder sets revealed that no Aspfu HTGs, 102 (44% of 231) Aspfl HTGs, and 74 (19% of 397) Aspni HTGs are shared by the two programs.

This surprising finding suggests that, just like the composition-based method, different phyletic distribution-based methods also produce very different HTG predictions. Therefore, it is not wise to use one surrogate method’s prediction to evaluate the other surrogate method’s performance. Gene-by-gene phylogenetic analysis, although performed at a much lower throughput, is the only gold-standard method to evaluate any HGT detection programs. A common practice in the literature is to take a two-step approach: run surrogate tools to narrow down to a short list of HTG candidates (e.g., from 10,000 to a few hundred genes for a typical fungal genome), and then use phylogenetic analysis to verify the candidates on a gene-by-gene basis [10,19]. Since different phyletic distribution-based tools tend to identify different sets of HTGs, our recommendation is to combine outputs from multiple tools and then perform phylogenetic analysis. HGT-Finder will be a very valuable addition to the toolbox of HGT research because many of its predictions can be verified by phylogenetic analysis, it is fully automated, and much easier to install and run.

HGT-Finder requires pre-annotated genomes (*i.e.*, protein-coding genes should be predicted prior to the HGT-Finder run). Because a statistical distribution of *X* values is needed for the *P* and *Q* value calculation, HGT-Finder will work best for genome-scale HGT detection and may not work for individual genes. The HGT-Finder program with source code, example files (with Aspfl BLAST output), and documents are freely available at http://cys.bios.niu.edu/HGTFinder/HGTFinder.tar.gz, which can be run on command-line terminals of OS X and Linux computers.

## 3. Materials and Methods

### 3.1. Data Sources

We downloaded the protein set, the CDS (coding sequence) set, the gene annotation set (including GO, KOG and KEGG), and the GFF (general feature format) set of *Aspergillus fumigatus* Af293 (9781 genes), *Aspergillus nidulans* FGSC A4 (10,680 genes), and *Aspergillus flavus* NRRL3357 (12,604 genes) from the Joint Genome Institute’s MycoCosm database [43] in May, 2015. Sequences in the protein sets were searched using BLASTP against the NCBI-nr database. The NCBI Taxonomy database was downloaded and parsed to retrieve the taxonomy linkage information of each hit protein, which was used as input for HGT-Finder.

### 3.2. Algorithm for HGT Detection

For each protein *g* of the query genome *Q*, examine its BLAST hits: 

for each hit genome *H*_i_, calculate:

similarity ratio: $R=\frac{S^{\prime }}{S}$ where *S*’ is the bit score hitting the best hit protein in *H*_i_ and *S* is the bit score for *g* hitting itself, and

taxonomic distance: $D=\frac{N'}{N}$

How *D* is calculated: 

if *Q* has *N* levels in its taxonomic lineage (separated by “;” in example below) according to the NCBI taxonomy database, *N*’ will be the number of steps from the last level tracing back to the taxonomic level *T* containing both *Q* and *H*_i_ with respect to *Q*’s lineage

e.g., *N* = 15, *N*’ = 7, *Q* = *Aspergillus fumigatus*, and *H*_i_ = Bipolaris maydis C5 in example below (*T* is **leotiomyceta**, denoted by “*”)

*Q*: cellular organisms; Eukaryota; Opisthokonta; Fungi; Dikarya; Ascomycota; saccharomyceta; Pezizomycotina; *leotiomyceta*; Eurotiomycetes; Eurotiomycetidae; Eurotiales; Aspergillaceae; *Aspergillus*; *Aspergillus fumigatus*

*H*_i_: cellular organisms; Eukaryota; Opisthokonta; Fungi; Dikarya; Ascomycota; saccharomyceta; Pezizomycotina; *leotiomyceta*; dothideomyceta; Dothideomycetes; Pleosporomycetidae; Pleosporales; Pleosporineae; Pleosporaceae; Bipolaris; Bipolaris maydis;Bipolaris maydis C5.

For each query protein, we will then calculate a transfer index: X = $\frac{\sum_{\text{i}=1}^{\text{k}}\frac{\text{RD}}{\text{i}}}{\sqrt{\text{k}}}$, where *k* is the number of hit genomes; *i* is the rank in the BLAST output sorted based on *E*-value. Therefore *X* ∈ [0,1], a higher *X* means a higher probability of being HTG. This equation gives top hits a higher weight. A query protein having top hits (large *R* and small *i*) from distant species (large *D*) will have a higher *X*.

### 3.3. Evolutionary Analysis

For phylogenetic analysis, protein sequences were aligned using MAFFT v6.850b [44] and the output, multiple sequence alignment, was used as input to build an approximate maximum-likelihood phylogenetic tree using FastTree v.2.1.8 [45], which implements an ultrafast and fairly accurate approximate maximum-likelihood method. The accuracy of FastTree phylogeny is considered to be slightly better than PhyML with default parameters; it is also 100 to 1000 times faster and requires much less computer memory. FastTree analyses were conducted with default parameters: the amino acid substitution matrix was JTT, the number of rate categories of sites (CAT model) was 20, and the local support values of each node were computed by resampling the site likelihoods 1000 times and performing the Shimodaira-Hasegawa test.

For *K*_a_/*K*_s_ analysis, we selected *Aspergillus ochraceoroseus* IBT 24754 (Aspoc) as the subject genome for comparison with Aspni, *Aspergillus terreus* NIH 2624 (Aspte) for comparison with Aspfl, and *Neosartorya fischeri* NRRL 181 (Neofi) for comparison with Aspfu. Protein sets of the three subject genomes were downloaded from JGI. BLASTP was run to compare each pair of genomes (Aspni *vs.* Aspoc, Aspfl *vs.* Aspte, and Aspfu *vs.* Neofi). The reciprocal best BLASTP hit method [46] was then taken to derive orthologous gene pairs between the query and the subject genomes. For each orthologous gene pair, the two protein sequences were aligned using MAFFT. Afterwards, the amino acid alignment was converted into a codon alignment using pal2nal [47]. The codon alignment for each orthologous gene pair was input into the yn00 program of PAML [48] to calculate the *K*_a_, *K*_s_ and the *K*_a_/*K*_s_ ratio.

## Supplementary Materials

Supplementary materials can be accessed at: http://www.mdpi.com/2072-6651/7/10/4035/s1.
