# Supplementary material for: HGT-Finder: A New Tool for Horizontal Gene Transfer Finding and Application to Aspergillus genomes
Source: Toxins (Basel). 2015 Oct 9;7(10):4035–53. doi: 10.3390/toxins7104035 (PMC4626719; doi:10.3390/toxins7104035)
Supplement: Supplementary file 1 [file toxins-07-04035-s001.zip › toxins-97781-supplementary/toxins-97781-figure S1-S2.pdf]

% of HTGs with best non-self BLAST hit from species of different taxonomic groups

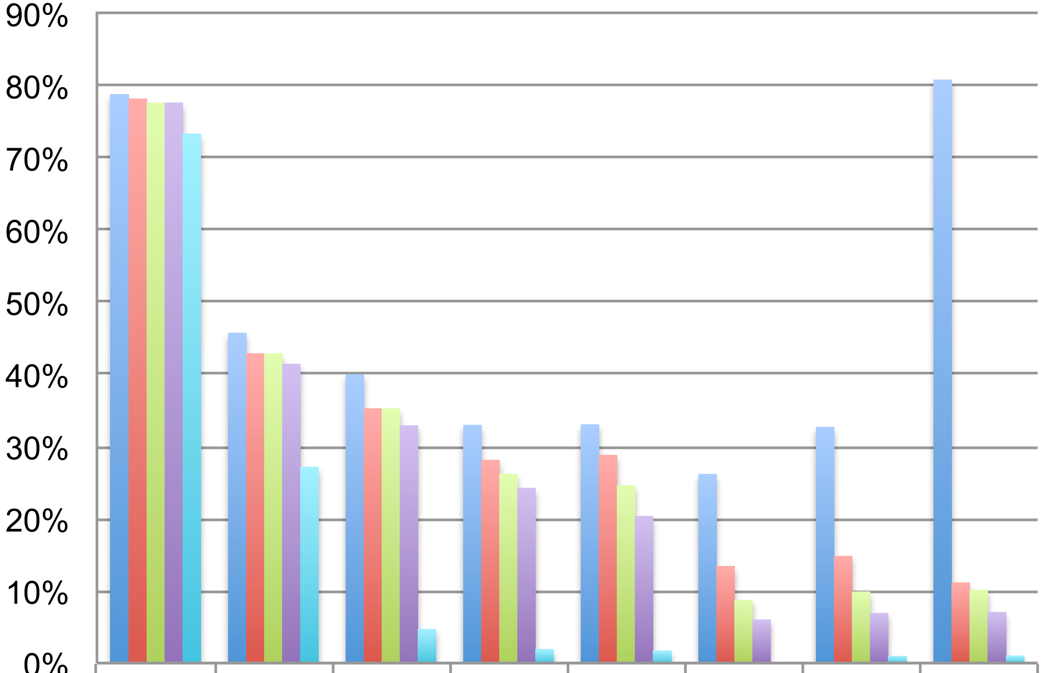

	0.2	0.3	0.4	0.5	0.6	0.7	0.8	0.9
not the same genus (Aspergillus)	79%	46%	40%	33%	33%	26%	33%	81%
not the same family (Aspergillaceae)	78%	43%	35%	28%	29%	13%	15%	11%
not the same order (Eurotiales)	78%	43%	35%	26%	25%	9%	10%	10%
not the same subclass (Eurotiomycetidae)	78%	41%	33%	24%	20%	6%	7%	7%
not leotiomyceta	73%	27%	5%	2%	2%	0%	1%	1%

% of HTGs with best non-self BLAST hit from species of different taxonomic groups

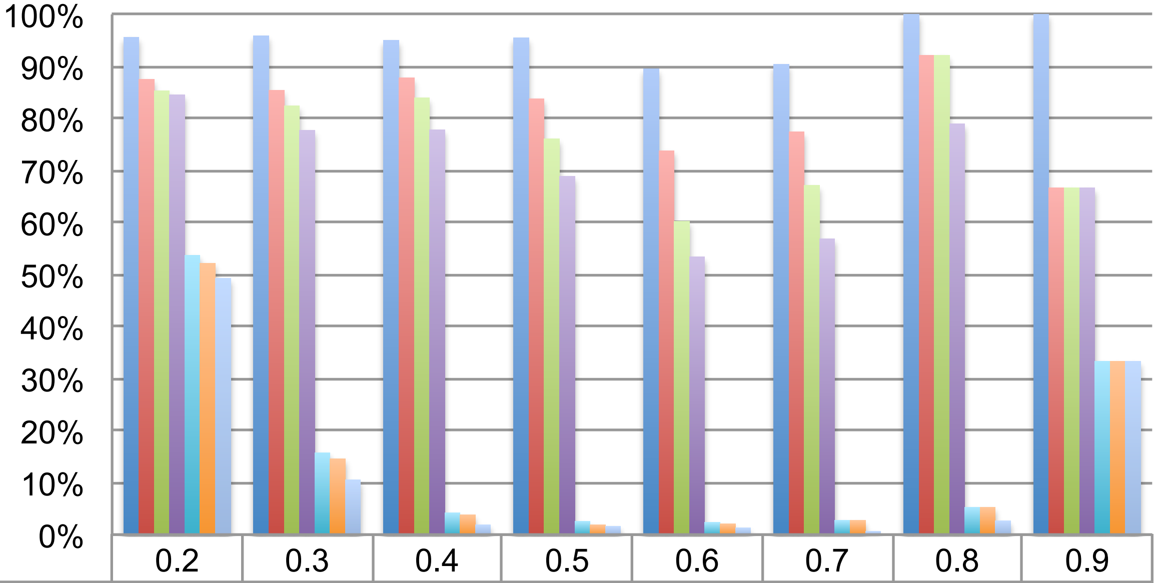

	0.2	0.3	0.4	0.5	0.6	0.7	0.8	0.9
not the same genus (Aspergillus)	96%	96%	95%	95%	90%	90%	100%	100.0%
not the same family (Aspergillaceae)	88%	85%	88%	84%	74%	77%	92%	66.7%
not the same order (Eurotiales)	85%	82%	84%	76%	60%	67%	92%	66.7%
not the same subclass (Eurotiomycetidae)	85%	78%	78%	69%	53%	57%	79%	66.7%
not leotiomyceta	54%	16%	4%	3%	2%	3%	5%	33.3%
not saccharomyceta	52%	15%	4%	2%	2%	3%	5%	33.3%
not Dikarya	49%	11%	2%	2%	1%	1%	3%	33.3%
