# Supplementary material for: HGT-Finder: A New Tool for Horizontal Gene Transfer Finding and Application to Aspergillus genomes
Source: Toxins (Basel). 2015 Oct 9;7(10):4035–53. doi: 10.3390/toxins7104035 (PMC4626719; doi:10.3390/toxins7104035)
Supplement: Supplementary file 1 [file toxins-07-04035-s001.zip › toxins-97781-supplementary/toxins-97781-Supplemental data file 1.pdf]

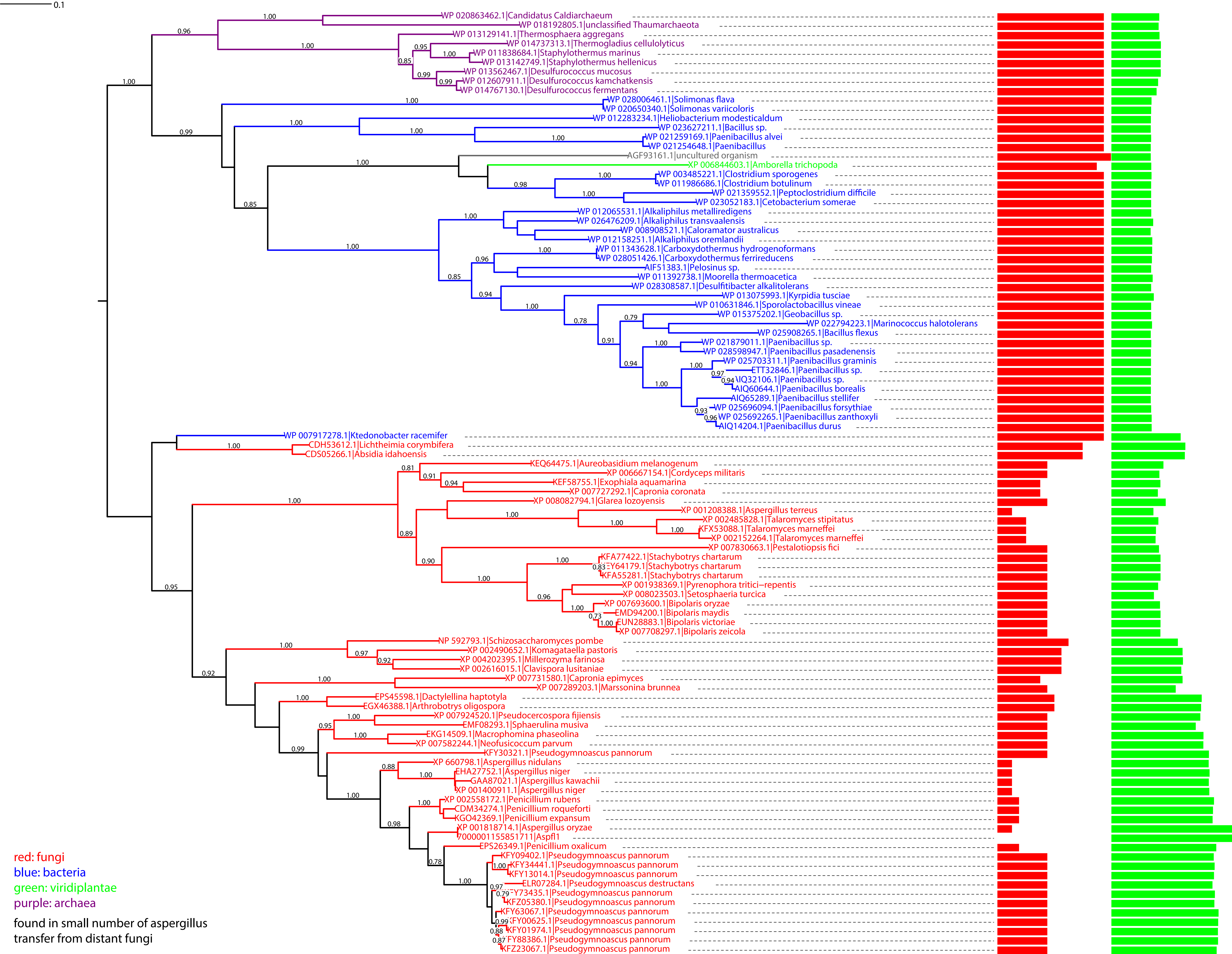
0.1
WP 020863462.1|Candidatus Caldiarchaeum
WP 018192805.1|unclassified Thaumarchaeota
WP 013129141.1|Thermosphaera aggregans
WP 014737313.1|Thermogladius cellulolyticus
WP 011838684.1|Staphylothermus marinus
WP 013142749.1|Staphylothermus hellenicus
WP 013562467.1|Desulfurococcus mucosus
WP 012607911.1|Desulfurococcus kamchatkensis
WP 014767130.1|Desulfurococcus fermentans
WP 028006461.1|Solimonas flava
WP 020650340.1|Solimonas variicoloris
WP 012283234.1|Heliobacterium modesticaldum
WP 023627211.1|Bacillus sp.
WP 021259169.1|Paenibacillus alvei
WP 021254648.1|Paenibacillus
AGF93161.1|uncultured organism
XP 006844603.1|Amborella trichopoda
WP 003485221.1|Clostridium sporogenes
WP 011986686.1|Clostridium botulinum
WP 021359552.1|Peptoclostridium difficile
WP 023052183.1|Cetobacterium somerae
WP 012065531.1|Alkaliphilus metalliredigens
WP 026476209.1|Alkaliphilus transvaalensis
WP 008908521.1|Caloramator australicus
WP 012158251.1|Alkaliphilus oremlandii
WP 011343628.1|Carboxydothermus hydrogenoformans
WP 028051426.1|Carboxydothermus ferrireducens
AIF51383.1|Pelosinus sp.
WP 011392738.1|Moorella thermoacetica
WP 028308587.1|Desulfitibacter alkalitolerans
WP 013075993.1|Kyrpidia tusciae
WP 010631846.1|Sporolactobacillus vineae
WP 015375202.1|Geobacillus sp.
WP 022794223.1|Marinococcus halotolerans
WP 025908265.1|Bacillus flexus
WP 021879011.1|Paenibacillus sp.
WP 028598947.1|Paenibacillus pasadenensis
WP 025703311.1|Paenibacillus graminis
ETT32846.1|Paenibacillus sp.
AIQ32106.1|Paenibacillus sp.
AIQ60644.1|Paenibacillus borealis
AIQ65289.1|Paenibacillus stellifer
WP 025696094.1|Paenibacillus forsythiae
WP 025692265.1|Paenibacillus zanthoxyli
AIQ14204.1|Paenibacillus durus
WP 007917278.1|Ktedonobacter racemifer
CDH53612.1|Lichtheimia corymbifera
CDS05266.1|Absidia idahoensis
KEQ64475.1|Aureobasidium melanogenum
XP 006667154.1|Cordyceps militaris
KEF58755.1|Exophiala aquamarina
XP 007727292.1|Capronia coronata
XP 008082794.1|Glarea lozoyensis
XP 001208388.1|Aspergillus terreus
XP 002485828.1|Talaromyces stipitatus
KFX53088.1|Talaromyces marneffei
XP 002152264.1|Talaromyces marneffei
XP 007830663.1|Pestalotiopsis fici
KFA77422.1|Stachybotrys chartarum
EY64179.1|Stachybotrys chartarum
KFA55281.1|Stachybotrys chartarum
XP 001938369.1|Pyrenophora tritici−repentis
XP 008023503.1|Setosphaeria turcica
XP 007693600.1|Bipolaris oryzae
EMD94200.1|Bipolaris maydis
EUN28883.1|Bipolaris victoriae
XP 007708297.1|Bipolaris zeicola
NP 592793.1|Schizosaccharomyces pombe
XP 002490652.1|Komagataella pastoris
XP 004202395.1|Millerozyma farinosa
XP 002616015.1|Clavispora lusitaniae
XP 007731580.1|Capronia epimyces
XP 007289203.1|Marssonina brunnea
EPS45598.1|Dactylellina haptotyla
EGX46388.1|Arthrobotrys oligospora
XP 007924520.1|Pseudocercospora fijiensis
EMF08293.1|Sphaerulina musiva
EKG14509.1|Macrophomina phaseolina
XP 007582244.1|Neofusicoccum parvum
KFY30321.1|Pseudogymnoascus pannorum
XP 660798.1|Aspergillus nidulans
EHA27752.1|Aspergillus niger
GAA87021.1|Aspergillus kawachii
XP 001400911.1|Aspergillus niger
XP 002558172.1|Penicillium rubens
CDM34274.1|Penicillium roqueforti
KGO42369.1|Penicillium expansum
XP 001818714.1|Aspergillus oryzae
7000001155851711|Aspfl1
EPS26349.1|Penicillium oxalicum
KFY09402.1|Pseudogymnoascus pannorum
KFY34441.1|Pseudogymnoascus pannorum
KFY13014.1|Pseudogymnoascus pannorum
ELR07284.1|Pseudogymnoascus destructans
KFY73435.1|Pseudogymnoascus pannorum
KFZ05380.1|Pseudogymnoascus pannorum
KFY63067.1|Pseudogymnoascus pannorum
KFY00625.1|Pseudogymnoascus pannorum
KFY01974.1|Pseudogymnoascus pannorum
KFY88386.1|Pseudogymnoascus pannorum
KFZ23067.1|Pseudogymnoascus pannorum
red: fungi
blue: bacteria
green: viridiplantae
purple: archaea
found in small number of aspergillus
transfer from distant fungi

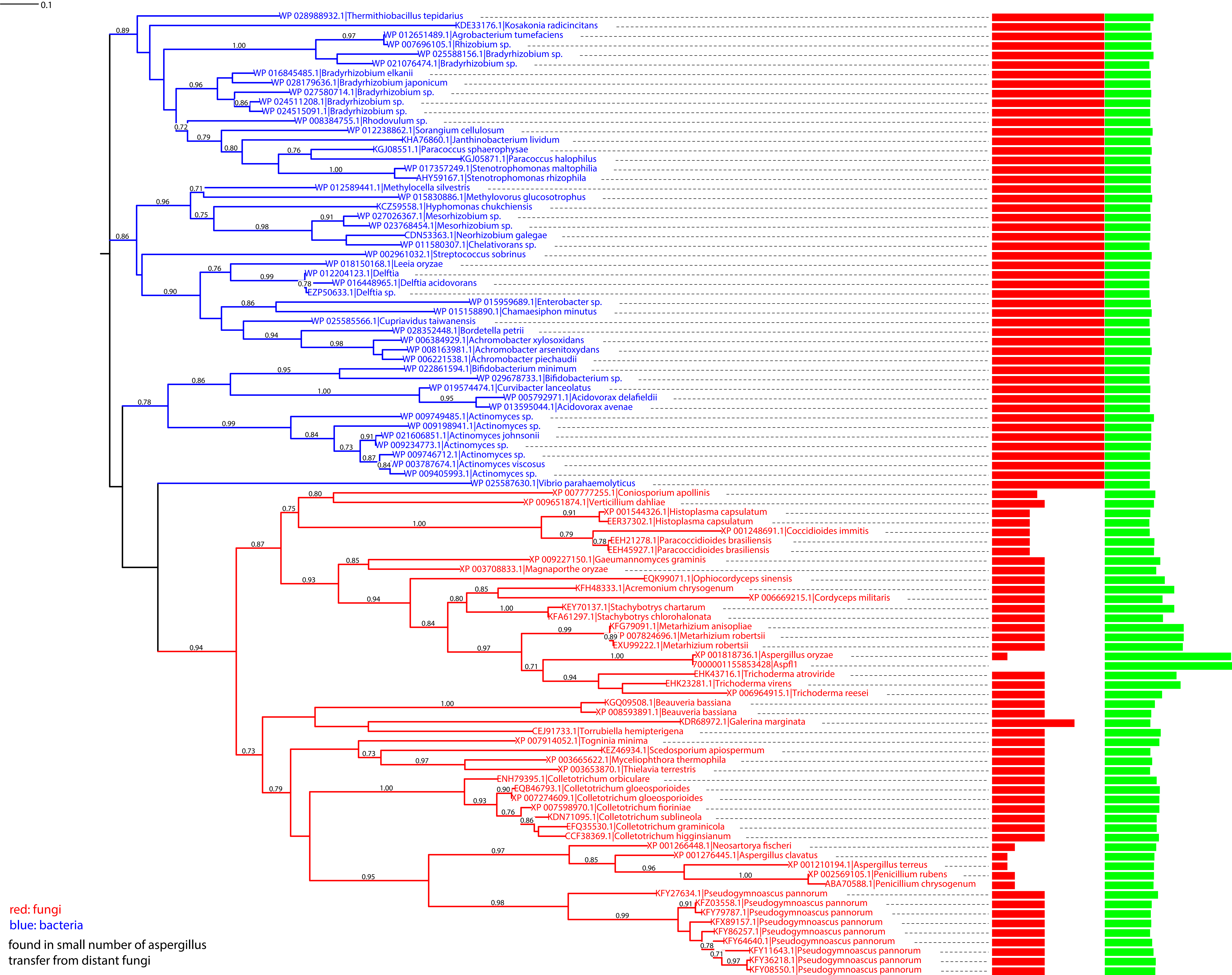
0.1
WP 028988932.1|Thermithiobacillus tepidarius
KDE33176.1|Kosakonia radicincitans
WP 012651489.1|Agrobacterium tumefaciens
WP 007696105.1|Rhizobium sp.
WP 025588156.1|Bradyrhizobium sp.
WP 021076474.1|Bradyrhizobium sp.
WP 016845485.1|Bradyrhizobium elkanii
WP 028179636.1|Bradyrhizobium japonicum
WP 027580714.1|Bradyrhizobium sp.
WP 024511208.1|Bradyrhizobium sp.
WP 024515091.1|Bradyrhizobium sp.
WP 008384755.1|Rhodovulum sp.
WP 012238862.1|Sorangium cellulosum
KHA76860.1|Janthinobacterium lividum
KGJ08551.1|Paracoccus sphaerophysae
KGJ05871.1|Paracoccus halophilus
WP 017357249.1|Stenotrophomonas maltophilia
AHY59167.1|Stenotrophomonas rhizophila
WP 012589441.1|Methylocella silvestris
WP 015830886.1|Methylovorus glucosotrophus
KCZ59558.1|Hyphomonas chukchiensis
WP 027026367.1|Mesorhizobium sp.
WP 023768454.1|Mesorhizobium sp.
CDN53363.1|Neorhizobium galegae
WP 011580307.1|Chelativorans sp.
WP 002961032.1|Streptococcus sobrinus
WP 018150168.1|Leeia oryzae
WP 012204123.1|Delftia
WP 016448965.1|Delftia acidovorans
EZP50633.1|Delftia sp.
WP 015959689.1|Enterobacter sp.
WP 015158890.1|Chamaesiphon minutus
WP 025585566.1|Cupriavidus taiwanensis
WP 028352448.1|Bordetella petrii
WP 006384929.1|Achromobacter xylosoxidans
WP 008163981.1|Achromobacter arsenitoxydans
WP 006221538.1|Achromobacter piechaudii
WP 022861594.1|Bifidobacterium minimum
WP 029678733.1|Bifidobacterium sp.
WP 019574474.1|Curvibacter lanceolatus
WP 005792971.1|Acidovorax delafieldii
WP 013595044.1|Acidovorax avenae
WP 009749485.1|Actinomyces sp.
WP 009198941.1|Actinomyces sp.
WP 021606851.1|Actinomyces johnsonii
WP 009234773.1|Actinomyces sp.
WP 009746712.1|Actinomyces sp.
WP 003787674.1|Actinomyces viscosus
WP 009405993.1|Actinomyces sp.
WP 025587630.1|Vibrio parahaemolyticus
XP 007777255.1|Coniosporium apollinis
XP 009651874.1|Verticillium dahliae
XP 001544326.1|Histoplasma capsulatum
EER37302.1|Histoplasma capsulatum
XP 001248691.1|Coccidioides immitis
EEH21278.1|Paracoccidioides brasiliensis
EEH45927.1|Paracoccidioides brasiliensis
XP 009227150.1|Gaeumannomyces graminis
XP 003708833.1|Magnaporthe oryzae
EQK99071.1|Ophiocordyceps sinensis
KFH48333.1|Acremonium chrysogenum
XP 006669215.1|Cordyceps militaris
KEY70137.1|Stachybotrys chartarum
KFA61297.1|Stachybotrys chlorohalonata
KFG79091.1|Metarhizium anisopliae
P 007824696.1|Metarhizium robertsii
EXU99222.1|Metarhizium robertsii
XP 001818736.1|Aspergillus oryzae
7000001155853428|Aspfl1
EHK43716.1|Trichoderma atroviride
EHK23281.1|Trichoderma virens
XP 006964915.1|Trichoderma reesei
KGQ09508.1|Beauveria bassiana
XP 008593891.1|Beauveria bassiana
KDR68972.1|Galerina marginata
CEJ91733.1|Torrubiella hemipterigena
XP 007914052.1|Togninia minima
KEZ46934.1|Scedosporium apiospermum
XP 003665622.1|Myceliophthora thermophila
XP 003653870.1|Thielavia terrestris
ENH79395.1|Colletotrichum orbiculare
EQB46793.1|Colletotrichum gloeosporioides
XP 007274609.1|Colletotrichum gloeosporioides
XP 007598970.1|Colletotrichum fioriniae
KDN71095.1|Colletotrichum sublineola
EFQ35530.1|Colletotrichum graminicola
CCF38369.1|Colletotrichum higginsianum
XP 001266448.1|Neosartorya fischeri
XP 001276445.1|Aspergillus clavatus
XP 001210194.1|Aspergillus terreus
XP 002569105.1|Penicillium rubens
ABA70588.1|Penicillium chrysogenum
KFY27634.1|Pseudogymnoascus pannorum
KFZ03558.1|Pseudogymnoascus pannorum
KFY79787.1|Pseudogymnoascus pannorum
KFX89157.1|Pseudogymnoascus pannorum
KFY86257.1|Pseudogymnoascus pannorum
KFY64640.1|Pseudogymnoascus pannorum
KFY11643.1|Pseudogymnoascus pannorum
KFY36218.1|Pseudogymnoascus pannorum
KFY08550.1|Pseudogymnoascus pannorum
red: fungi
blue: bacteria
found in small number of aspergillus
transfer from distant fungi

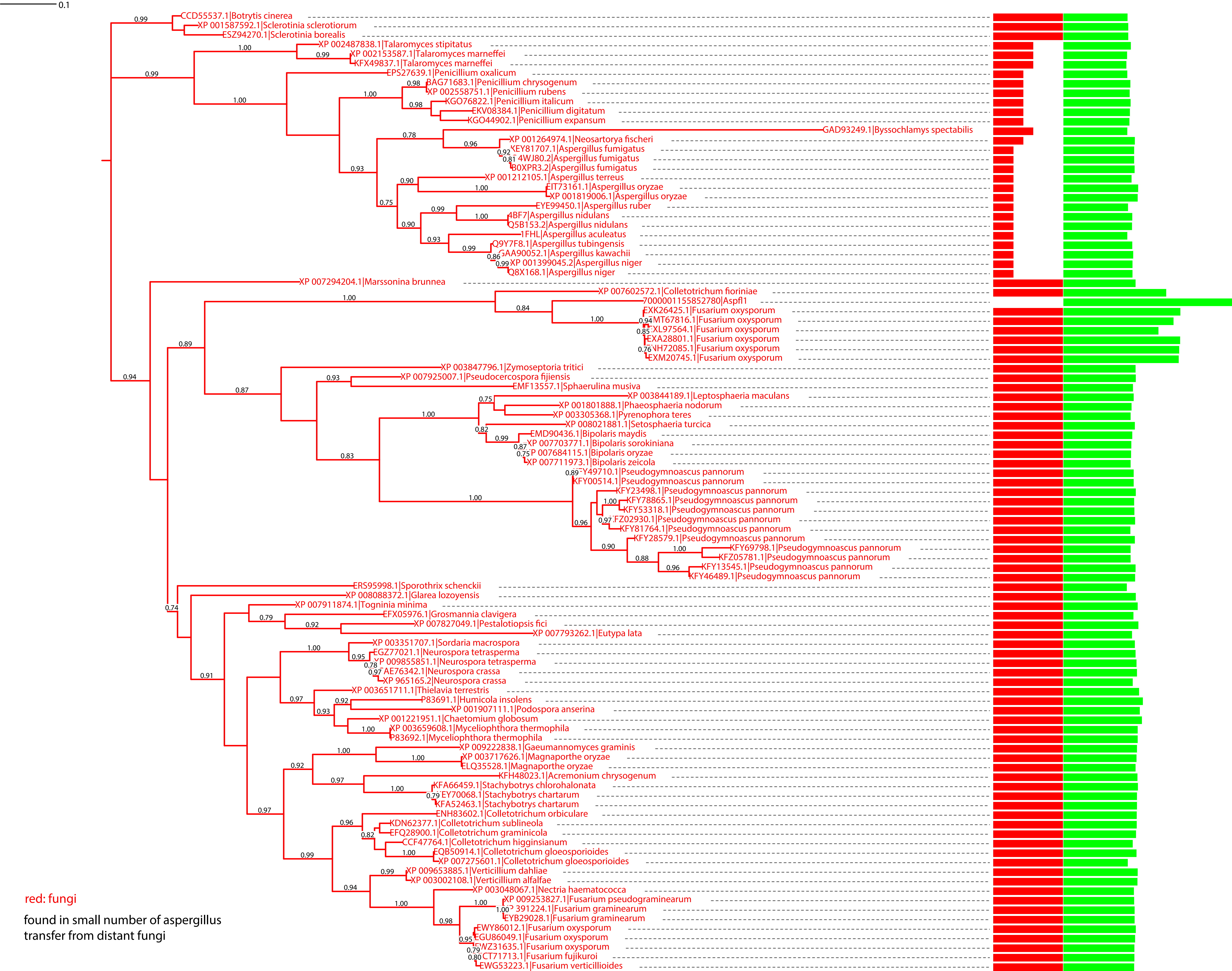

0.1
CCD55537.1|Botrytis cinerea
XP 001587592.1|Sclerotinia sclerotiorum
ESZ94270.1|Sclerotinia borealis
XP 002487838.1|Talaromyces stipitatus
XP 002153587.1|Talaromyces marneffei
KFX49837.1|Talaromyces marneffei
EPS27639.1|Penicillium oxalicum
BAG71683.1|Penicillium chrysogenum
XP 002558751.1|Penicillium rubens
KGO76822.1|Penicillium italicum
EKV08384.1|Penicillium digitatum
KGO44902.1|Penicillium expansum
GAD93249.1|Byssochlamys spectabilis
XP 001264974.1|Neosartorya fischeri
KEY81707.1|Aspergillus fumigatus
4WJ80.2|Aspergillus fumigatus
B0XPR3.2|Aspergillus fumigatus
XP 001212105.1|Aspergillus terreus
EIT73161.1|Aspergillus oryzae
XP 001819006.1|Aspergillus oryzae
EYE99450.1|Aspergillus ruber
4BF7|Aspergillus nidulans
Q5B153.2|Aspergillus nidulans
1FHL|Aspergillus aculeatus
Q9Y7F8.1|Aspergillus tubingensis
GAA90052.1|Aspergillus kawachii
XP 001399045.2|Aspergillus niger
Q8X168.1|Aspergillus niger
XP 007294204.1|Marssonina brunnea
XP 007602572.1|Colletotrichum fioriniae
7000001155852780|Aspfl1
EXK26425.1|Fusarium oxysporum
EMT67816.1|Fusarium oxysporum
XL97564.1|Fusarium oxysporum
EXA28801.1|Fusarium oxysporum
NH72085.1|Fusarium oxysporum
EXM20745.1|Fusarium oxysporum
XP 003847796.1|Zymoseptoria tritici
XP 007925007.1|Pseudocercospora fijiensis
EMF13557.1|Sphaerulina musiva
XP 003844189.1|Leptosphaeria maculans
XP 001801888.1|Phaeosphaeria nodorum
XP 003305368.1|Pyrenophora teres
XP 008021881.1|Setosphaeria turcica
EMD90436.1|Bipolaris maydis
XP 007703771.1|Bipolaris sorokiniana
P 007684115.1|Bipolaris oryzae
XP 007711973.1|Bipolaris zeicola
Y49710.1|Pseudogymnoascus pannorum
KFY00514.1|Pseudogymnoascus pannorum
KFY23498.1|Pseudogymnoascus pannorum
KFY78865.1|Pseudogymnoascus pannorum
KFY53318.1|Pseudogymnoascus pannorum
FZ02930.1|Pseudogymnoascus pannorum
KFY81764.1|Pseudogymnoascus pannorum
KFY28579.1|Pseudogymnoascus pannorum
KFY69798.1|Pseudogymnoascus pannorum
KFZ05781.1|Pseudogymnoascus pannorum
KFY13545.1|Pseudogymnoascus pannorum
KFY46489.1|Pseudogymnoascus pannorum
ERS95998.1|Sporothrix schenckii
XP 008088372.1|Glarea lozoyensis
XP 007911874.1|Togninia minima
EFX05976.1|Grosmannia clavigera
XP 007827049.1|Pestalotiopsis fici
XP 007793262.1|Eutypa lata
XP 003351707.1|Sordaria macrospora
EGZ77021.1|Neurospora tetrasperma
XP 009855851.1|Neurospora tetrasperma
AE76342.1|Neurospora crassa
XP 965165.2|Neurospora crassa
XP 003651711.1|Thielavia terrestris
P83691.1|Humicola insolens
XP 001907111.1|Podospora anserina
XP 001221951.1|Chaetomium globosum
XP 003659608.1|Myceliophthora thermophila
P83692.1|Myceliophthora thermophila
XP 009222838.1|Gaeumannomyces graminis
XP 003717626.1|Magnaporthe oryzae
ELQ35528.1|Magnaporthe oryzae
KFH48023.1|Acremonium chrysogenum
KFA66459.1|Stachybotrys chlorohalonata
EY70068.1|Stachybotrys chartarum
KFA52463.1|Stachybotrys chartarum
ENH83602.1|Colletotrichum orbiculare
KDN62377.1|Colletotrichum sublineola
EFQ28900.1|Colletotrichum graminicola
CCF47764.1|Colletotrichum higginsianum
EQB50914.1|Colletotrichum gloeosporioides
XP 007275601.1|Colletotrichum gloeosporioides
XP 009653885.1|Verticillium dahliae
XP 003002108.1|Verticillium alfalfae
XP 003048067.1|Nectria haematococca
XP 009253827.1|Fusarium pseudograminearum
391224.1|Fusarium graminearum
EYB29028.1|Fusarium graminearum
EWY86012.1|Fusarium oxysporum
EGU86049.1|Fusarium oxysporum
WZ31635.1|Fusarium oxysporum
CT71713.1|Fusarium fujikuroi
EWG53223.1|Fusarium verticillioides
red: fungi
found in small number of aspergillus
transfer from distant fungi

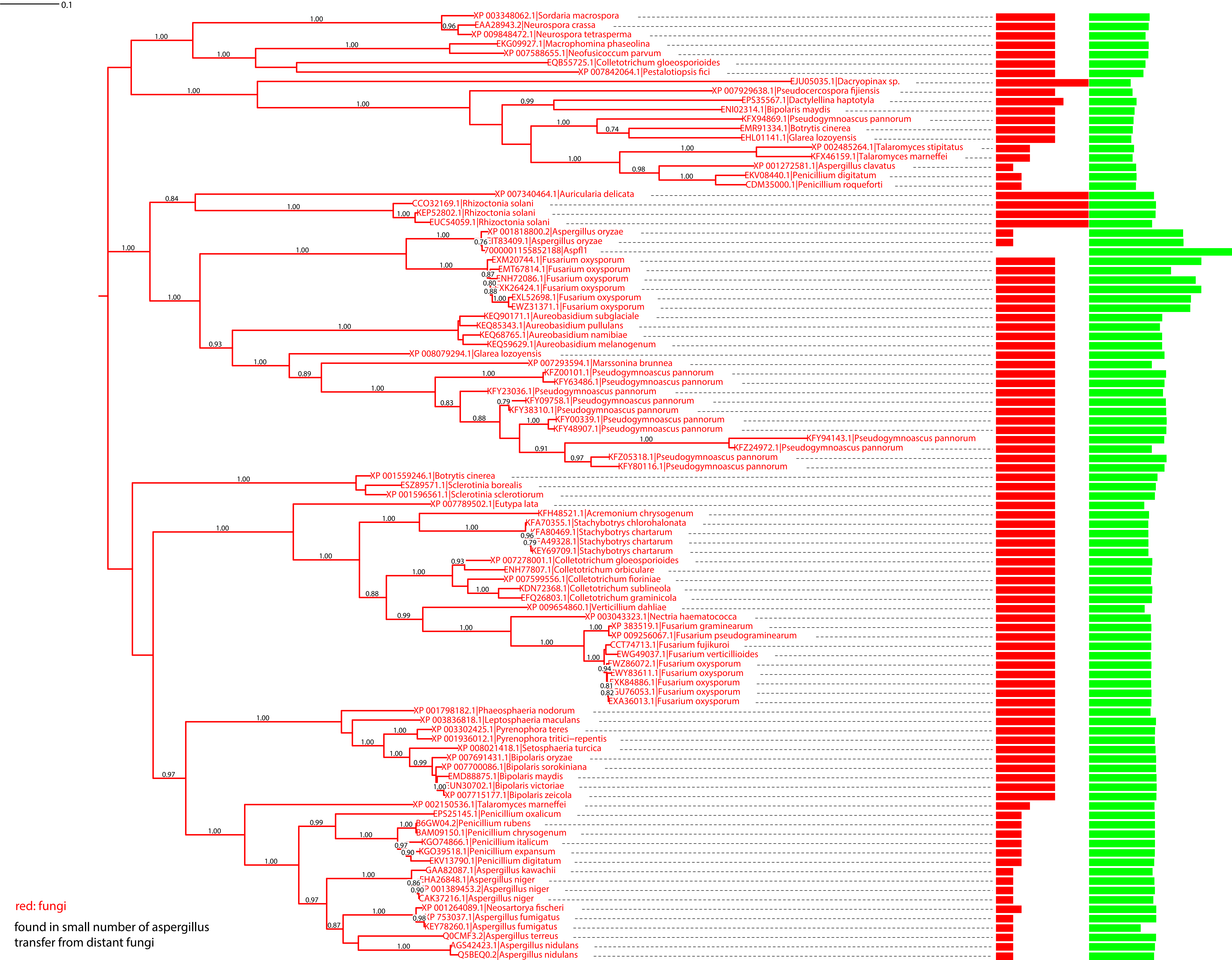
0.1
XP 003348062.1|Sordaria macrospora
EAA28943.2|Neurospora crassa
XP 009848472.1|Neurospora tetrasperma
EKG09927.1|Macrophomina phaseolina
XP 007588655.1|Neofusicoccum parvum
EQB55725.1|Colletotrichum gloeosporioides
XP 007842064.1|Pestalotiopsis fici
EJU05035.1|Dacryopinax sp.
XP 007929638.1|Pseudocercospora fijiensis
EPS35567.1|Dactylellina haptotyla
ENI02314.1|Bipolaris maydis
KFX94869.1|Pseudogymnoascus pannorum
EMR91334.1|Botrytis cinerea
EHL01141.1|Glarea lozoyensis
XP 002485264.1|Talaromyces stipitatus
KFX46159.1|Talaromyces marneffei
XP 001272581.1|Aspergillus clavatus
EKV08440.1|Penicillium digitatum
CDM35000.1|Penicillium roqueforti
XP 007340464.1|Auricularia delicata
CCO32169.1|Rhizoctonia solani
KEP52802.1|Rhizoctonia solani
EUC54059.1|Rhizoctonia solani
XP 001818800.2|Aspergillus oryzae
IT83409.1|Aspergillus oryzae
7000001155852188|Aspfl1
EXM20744.1|Fusarium oxysporum
EMT67814.1|Fusarium oxysporum
ENH72086.1|Fusarium oxysporum
XK26424.1|Fusarium oxysporum
EXL52698.1|Fusarium oxysporum
EWZ31371.1|Fusarium oxysporum
KEQ90171.1|Aureobasidium subglaciale
KEQ85343.1|Aureobasidium pullulans
KEQ68765.1|Aureobasidium namibiae
KEQ59629.1|Aureobasidium melanogenum
XP 008079294.1|Glarea lozoyensis
XP 007293594.1|Marssonina brunnea
KFZ00101.1|Pseudogymnoascus pannorum
KFY63486.1|Pseudogymnoascus pannorum
KFY23036.1|Pseudogymnoascus pannorum
KFY09758.1|Pseudogymnoascus pannorum
KFY38310.1|Pseudogymnoascus pannorum
KFY00339.1|Pseudogymnoascus pannorum
KFY48907.1|Pseudogymnoascus pannorum
KFY94143.1|Pseudogymnoascus pannorum
KFZ24972.1|Pseudogymnoascus pannorum
KFZ05318.1|Pseudogymnoascus pannorum
KFY80116.1|Pseudogymnoascus pannorum
XP 001559246.1|Botrytis cinerea
ESZ89571.1|Sclerotinia borealis
XP 001596561.1|Sclerotinia sclerotiorum
XP 007789502.1|Eutypa lata
KFH48521.1|Acremonium chrysogenum
KFA70355.1|Stachybotrys chlorohalonata
KFA80469.1|Stachybotrys chartarum
A49328.1|Stachybotrys chartarum
KEY69709.1|Stachybotrys chartarum
XP 007278001.1|Colletotrichum gloeosporioides
ENH77807.1|Colletotrichum orbiculare
XP 007599556.1|Colletotrichum fioriniae
KDN72368.1|Colletotrichum sublineola
EFQ26803.1|Colletotrichum graminicola
XP 009654860.1|Verticillium dahliae
XP 003043323.1|Nectria haematococca
XP 383519.1|Fusarium graminearum
XP 009256067.1|Fusarium pseudograminearum
CCT74713.1|Fusarium fujikuroi
EWG49037.1|Fusarium verticillioides
EWZ86072.1|Fusarium oxysporum
EWY83611.1|Fusarium oxysporum
EXK84886.1|Fusarium oxysporum
GU76053.1|Fusarium oxysporum
EXA36013.1|Fusarium oxysporum
XP 001798182.1|Phaeosphaeria nodorum
XP 003836818.1|Leptosphaeria maculans
XP 003302425.1|Pyrenophora teres
XP 001936012.1|Pyrenophora tritici–repentis
XP 008021418.1|Setosphaeria turcica
XP 007691431.1|Bipolaris oryzae
XP 007700086.1|Bipolaris sorokiniana
EMD88875.1|Bipolaris maydis
EUN30702.1|Bipolaris victoriae
XP 007715177.1|Bipolaris zeicola
XP 002150536.1|Talaromyces marneffei
EPS25145.1|Penicillium oxalicum
B6GW04.2|Penicillium rubens
BAM09150.1|Penicillium chrysogenum
KGO74866.1|Penicillium italicum
KGO39518.1|Penicillium expansum
EKV13790.1|Penicillium digitatum
GAA82087.1|Aspergillus kawachii
EHA26848.1|Aspergillus niger
P 001389453.2|Aspergillus niger
CAK37216.1|Aspergillus niger
XP 001264089.1|Neosartorya fischeri
P 753037.1|Aspergillus fumigatus
KEY78260.1|Aspergillus fumigatus
Q0CMF3.2|Aspergillus terreus
AGS42423.1|Aspergillus nidulans
Q5BEQ0.2|Aspergillus nidulans
red: fungi
found in small number of aspergillus
transfer from distant fungi

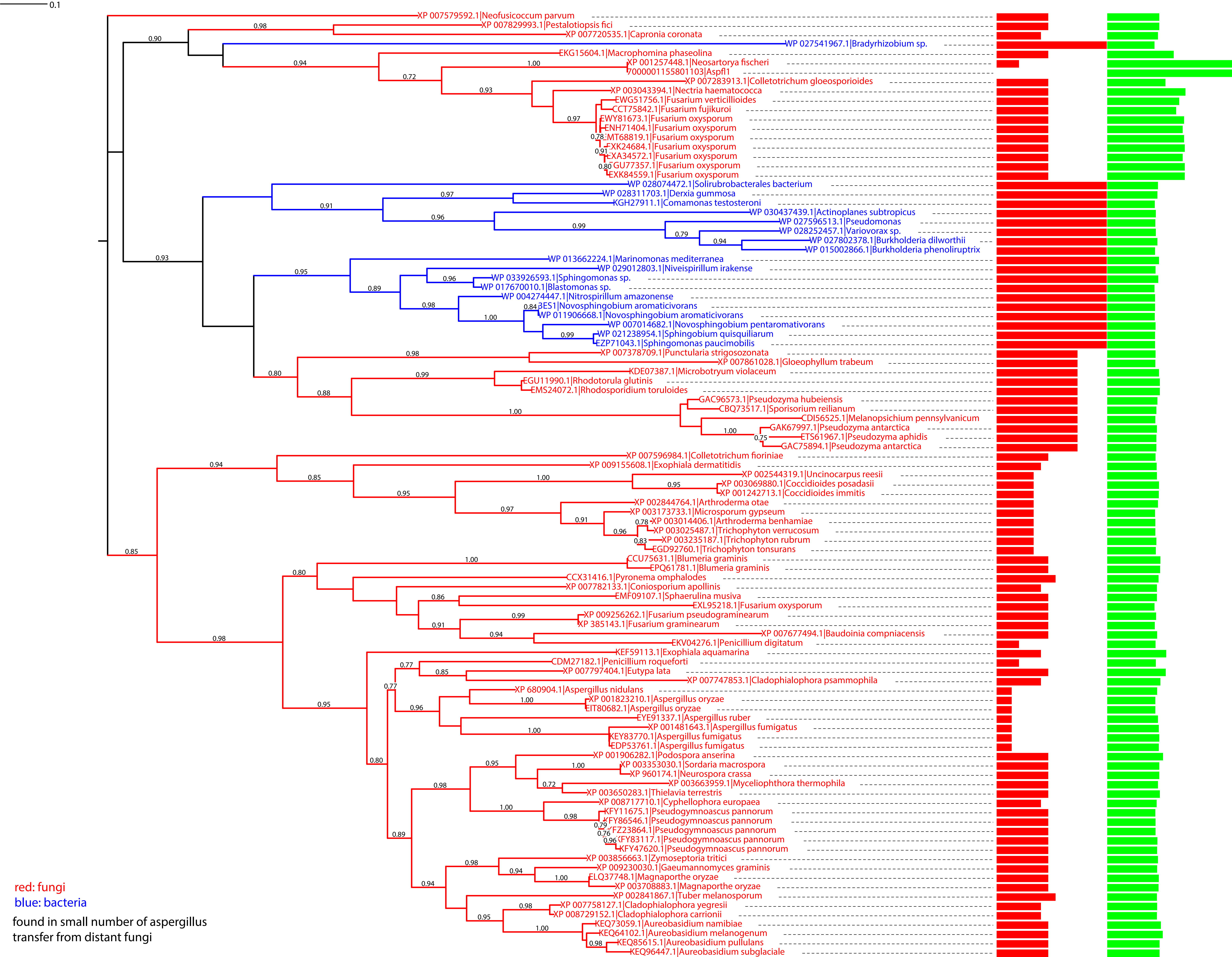
0.1
XP 007579592.1|Neofusicoccum parvum
XP 007829993.1|Pestalotiopsis fici
XP 007720535.1|Capronia coronata
WP 027541967.1|Bradyrhizobium sp.
EKG15604.1|Macrophomina phaseolina
XP 001257448.1|Neosartorya fischeri
7000001155801103|Aspfl1
XP 007283913.1|Colletotrichum gloeosporioides
XP 003043394.1|Nectria haematococca
EWG51756.1|Fusarium verticillioides
CCT75842.1|Fusarium fujikuroi
EWY81673.1|Fusarium oxysporum
ENH71404.1|Fusarium oxysporum
MT68819.1|Fusarium oxysporum
FXK24684.1|Fusarium oxysporum
EXA34572.1|Fusarium oxysporum
GU77357.1|Fusarium oxysporum
EXK84559.1|Fusarium oxysporum
WP 028074472.1|Solirubrobacterales bacterium
WP 028311703.1|Derxia gummosa
KGH27911.1|Comamonas testosteroni
WP 030437439.1|Actinoplanes subtropicus
WP 027596513.1|Pseudomonas
WP 028252457.1|Variovorax sp.
WP 027802378.1|Burkholderia dilworthii
WP 015002866.1|Burkholderia phenoliruptrix
WP 013662224.1|Marinomonas mediterranea
WP 029012803.1|Niveispirillum irakense
WP 033926593.1|Sphingomonas sp.
WP 017670010.1|Blastomonas sp.
WP 004274447.1|Nitrospirillum amazonense
3ES1|Novosphingobium aromaticivorans
WP 011906668.1|Novosphingobium aromaticivorans
WP 007014682.1|Novosphingobium pentaromativorans
WP 021238954.1|Sphingobium quisquiliarum
EZP71043.1|Sphingomonas paucimobilis
XP 007378709.1|Punctularia strigosozonata
XP 007861028.1|Gloeophyllum trabeum
KDE07387.1|Microbotryum violaceum
EGU11990.1|Rhodotorula glutinis
EMS24072.1|Rhodosporidium toruloides
GAC96573.1|Pseudozyma hubeiensis
CBQ73517.1|Sporisorium reilianum
CDI56525.1|Melanopsichium pennsylvanicum
GAK67997.1|Pseudozyma antarctica
ETS61967.1|Pseudozyma aphidis
GAC75894.1|Pseudozyma antarctica
XP 007596984.1|Colletotrichum fioriniae
XP 009155608.1|Exophiala dermatitidis
XP 002544319.1|Uncinocarpus reesii
XP 003069880.1|Coccidioides posadasii
XP 001242713.1|Coccidioides immitis
XP 002844764.1|Arthroderma otae
XP 003173733.1|Microsporum gypseum
XP 003014406.1|Arthroderma benhamiae
XP 003025487.1|Trichophyton verrucosum
XP 003235187.1|Trichophyton rubrum
EGD92760.1|Trichophyton tonsurans
CCU75631.1|Blumeria graminis
EPQ61781.1|Blumeria graminis
CCX31416.1|Pyronema omphalodes
XP 007782133.1|Coniosporium apollinis
EMF09107.1|Sphaerulina musiva
EXL95218.1|Fusarium oxysporum
XP 009256262.1|Fusarium pseudograminearum
XP 385143.1|Fusarium graminearum
XP 007677494.1|Baudoinia compniacensis
EKV04276.1|Penicillium digitatum
KEF59113.1|Exophiala aquamarina
CDM27182.1|Penicillium roqueforti
XP 007797404.1|Eutypa lata
XP 007747853.1|Cladophialophora psammophila
XP 680904.1|Aspergillus nidulans
XP 001823210.1|Aspergillus oryzae
EIT80682.1|Aspergillus oryzae
EYE91337.1|Aspergillus ruber
XP 001481643.1|Aspergillus fumigatus
KEY83770.1|Aspergillus fumigatus
EDP53761.1|Aspergillus fumigatus
XP 001906282.1|Podospora anserina
XP 003353030.1|Sordaria macrospora
XP 960174.1|Neurospora crassa
XP 003663959.1|Myceliophthora thermophila
XP 003650283.1|Thielavia terrestris
XP 008717710.1|Cyphellophora europaea
KFY11675.1|Pseudogymnoascus pannorum
KFY86546.1|Pseudogymnoascus pannorum
FZ23864.1|Pseudogymnoascus pannorum
KFY83117.1|Pseudogymnoascus pannorum
KFY47620.1|Pseudogymnoascus pannorum
XP 003856663.1|Zymoseptoria tritici
XP 009230030.1|Gaeumannomyces graminis
ELQ37748.1|Magnaporthe oryzae
XP 003708883.1|Magnaporthe oryzae
XP 002841867.1|Tuber melanosporum
XP 007758127.1|Cladophialophora yegresii
XP 008729152.1|Cladophialophora carrionii
KEQ73059.1|Aureobasidium namibiae
KEQ64102.1|Aureobasidium melanogenum
KEQ85615.1|Aureobasidium pullulans
KEQ96447.1|Aureobasidium subglaciale
red: fungi
blue: bacteria
found in small number of aspergillus
transfer from distant fungi

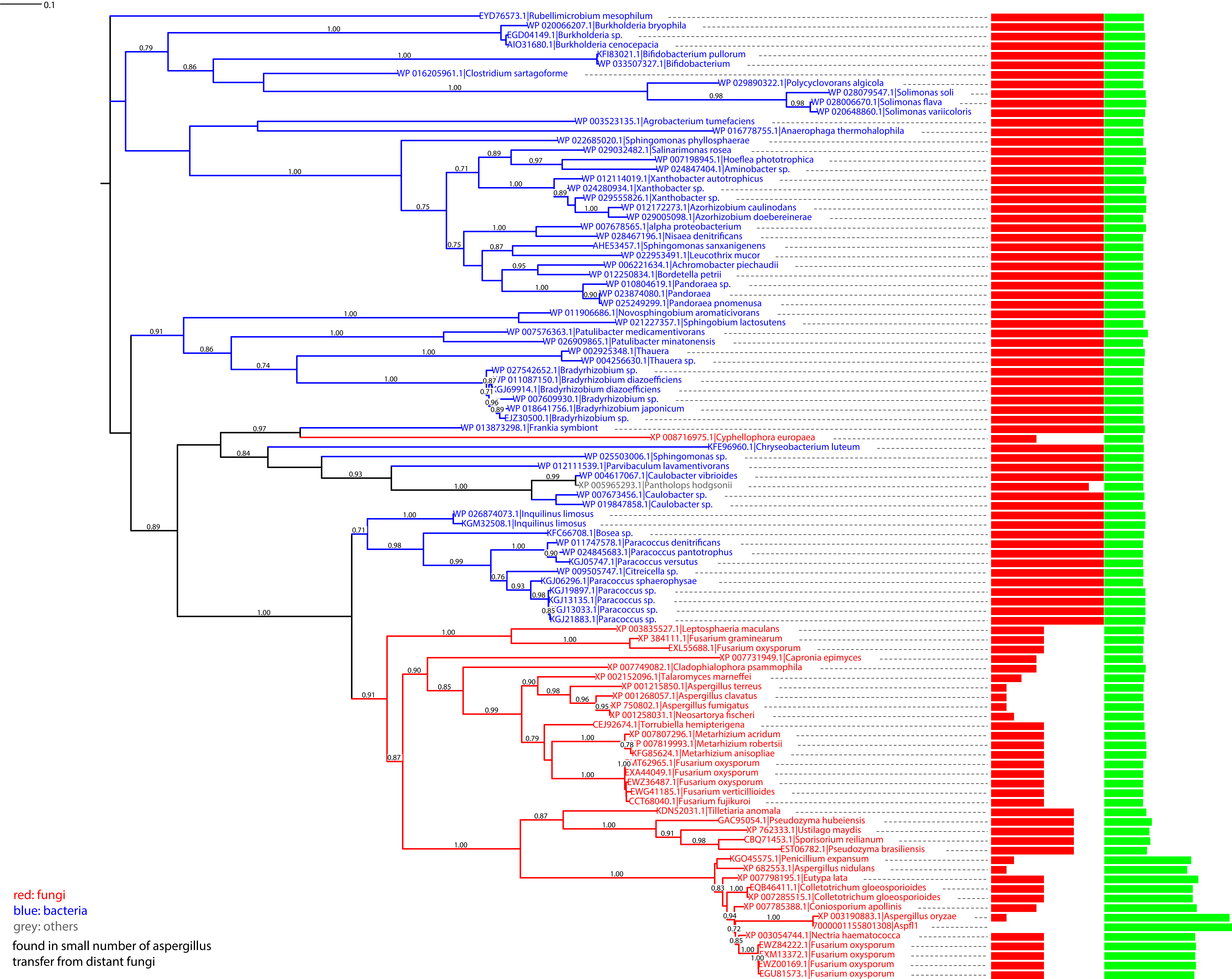
0.1
EYD76573.1|Rubellimicrobium mesophilum
WP 020066207.1|Burkholderia bryophila
EGD04149.1|Burkholderia sp.
AIO31680.1|Burkholderia cenocepacia
KFI83021.1|Bifidobacterium pullorum
WP 033507327.1|Bifidobacterium
WP 016205961.1|Clostridium sartagoforme
WP 029890322.1|Polycyclovorans algicola
WP 028079547.1|Solimonas soli
WP 028006670.1|Solimonas flava
WP 020648860.1|Solimonas variicoloris
WP 003523135.1|Agrobacterium tumefaciens
WP 016778755.1|Anaerophaga thermohalophila
WP 022685020.1|Sphingomonas phyllosphaerae
WP 029032482.1|Salinarimonas rosea
WP 007198945.1|Hoeflea phototrophica
WP 024847404.1|Aminobacter sp.
WP 012114019.1|Xanthobacter autotrophicus
WP 024280934.1|Xanthobacter sp.
WP 029555826.1|Xanthobacter sp.
WP 012172273.1|Azorhizobium caulinodans
WP 029005098.1|Azorhizobium doebereinerae
WP 007678565.1|alpha proteobacterium
WP 028467196.1|Nisaea denitrificans
AHE53457.1|Sphingomonas sanxanigenens
WP 022953491.1|Leucothrix mucor
WP 006221634.1|Achromobacter piechaudii
WP 012250834.1|Bordetella petrii
WP 010804619.1|Pandoraea sp.
WP 023874080.1|Pandoraea
WP 025249299.1|Pandoraea pnomenusa
WP 011906686.1|Novosphingobium aromaticivorans
WP 021227357.1|Sphingobium lactosutens
WP 007576363.1|Patulibacter medicamentivorans
WP 026909865.1|Patulibacter minatonensis
WP 002925348.1|Thauera
WP 004256630.1|Thauera sp.
WP 027542652.1|Bradyrhizobium sp.
/P 011087150.1|Bradyrhizobium diazoefficiens
GJ69914.1|Bradyrhizobium diazoefficiens
WP 007609930.1|Bradyrhizobium sp.
WP 018641756.1|Bradyrhizobium japonicum
EJZ30500.1|Bradyrhizobium sp.
WP 013873298.1|Frankia symbiont
XP 008716975.1|Cyphellophora europaea
KFE96960.1|Chryseobacterium luteum
WP 025503006.1|Sphingomonas sp.
WP 012111539.1|Parvibaculum lavamentivorans
WP 004617067.1|Caulobacter vibrioides
XP 005965293.1|Pantholops hodgsonii
WP 007673456.1|Caulobacter sp.
WP 019847858.1|Caulobacter sp.
WP 026874073.1|Inquilinus limosus
KGM32508.1|Inquilinus limosus
KFC66708.1|Bosea sp.
WP 011747578.1|Paracoccus denitrificans
WP 024845683.1|Paracoccus pantotrophus
KGJ05747.1|Paracoccus versutus
WP 009505747.1|Citreicella sp.
KGJ06296.1|Paracoccus sphaerophysae
KGJ19897.1|Paracoccus sp.
KGJ13135.1|Paracoccus sp.
GJ13033.1|Paracoccus sp.
KGJ21883.1|Paracoccus sp.
XP 003835527.1|Leptosphaeria maculans
XP 384111.1|Fusarium graminearum
EXL55688.1|Fusarium oxysporum
XP 007731949.1|Capronia epimyces
XP 007749082.1|Cladophialophora psammophila
XP 002152096.1|Talaromyces marneffei
XP 001215850.1|Aspergillus terreus
XP 001268057.1|Aspergillus clavatus
XP 750802.1|Aspergillus fumigatus
XP 001258031.1|Neosartorya fischeri
CEJ92674.1|Torrubiella hemipterigena
XP 007807296.1|Metarhizium acridum
P 007819993.1|Metarhizium robertsii
KFG85624.1|Metarhizium anisopliae
MT62965.1|Fusarium oxysporum
EXA44049.1|Fusarium oxysporum
EWZ36487.1|Fusarium oxysporum
EWG41185.1|Fusarium verticillioides
CCT68040.1|Fusarium fujikuroi
KDN52031.1|Tilletiaria anomala
GAC95054.1|Pseudozyma hubeiensis
XP 762333.1|Ustilago maydis
CBQ71453.1|Sporisorium reilianum
EST06782.1|Pseudozyma brasiliensis
KGO45575.1|Penicillium expansum
XP 682553.1|Aspergillus nidulans
XP 007798195.1|Eutypa lata
EQB46411.1|Colletotrichum gloeosporioides
XP 007285515.1|Colletotrichum gloeosporioides
XP 007785388.1|Coniosporium apollinis
XP 003190883.1|Aspergillus oryzae
7000001155801308|Aspfl1
XP 003054744.1|Nectria haematococca
EWZ84222.1|Fusarium oxysporum
XM13372.1|Fusarium oxysporum
EWZ00169.1|Fusarium oxysporum
EGU81573.1|Fusarium oxysporum
red: fungi
blue: bacteria
grey: others
found in small number of aspergillus
transfer from distant fungi

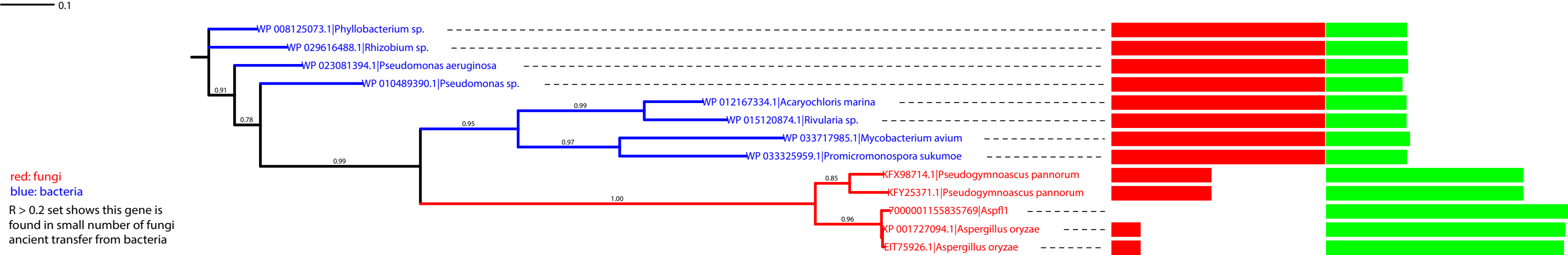

0.1
WP 008125073.1|Phyllobacterium sp.
WP 029616488.1|Rhizobium sp.
WP 023081394.1|Pseudomonas aeruginosa
WP 010489390.1|Pseudomonas sp.
WP 012167334.1|Acaryochloris marina
WP 015120874.1|Rivularia sp.
WP 033717985.1|Mycobacterium avium
WP 033325959.1|Promicromonospora sukumoe
KFX98714.1|Pseudogymnoascus pannorum
KFY25371.1|Pseudogymnoascus pannorum
7000001155835769|Aspfl1
XP 001727094.1|Aspergillus oryzae
EIT75926.1|Aspergillus oryzae
0.91
0.78
0.99
0.95
0.99
0.97
1.00
0.85
0.96
red: fungi
blue: bacteria
R > 0.2 set shows this gene is
found in small number of fungi
ancient transfer from bacteria

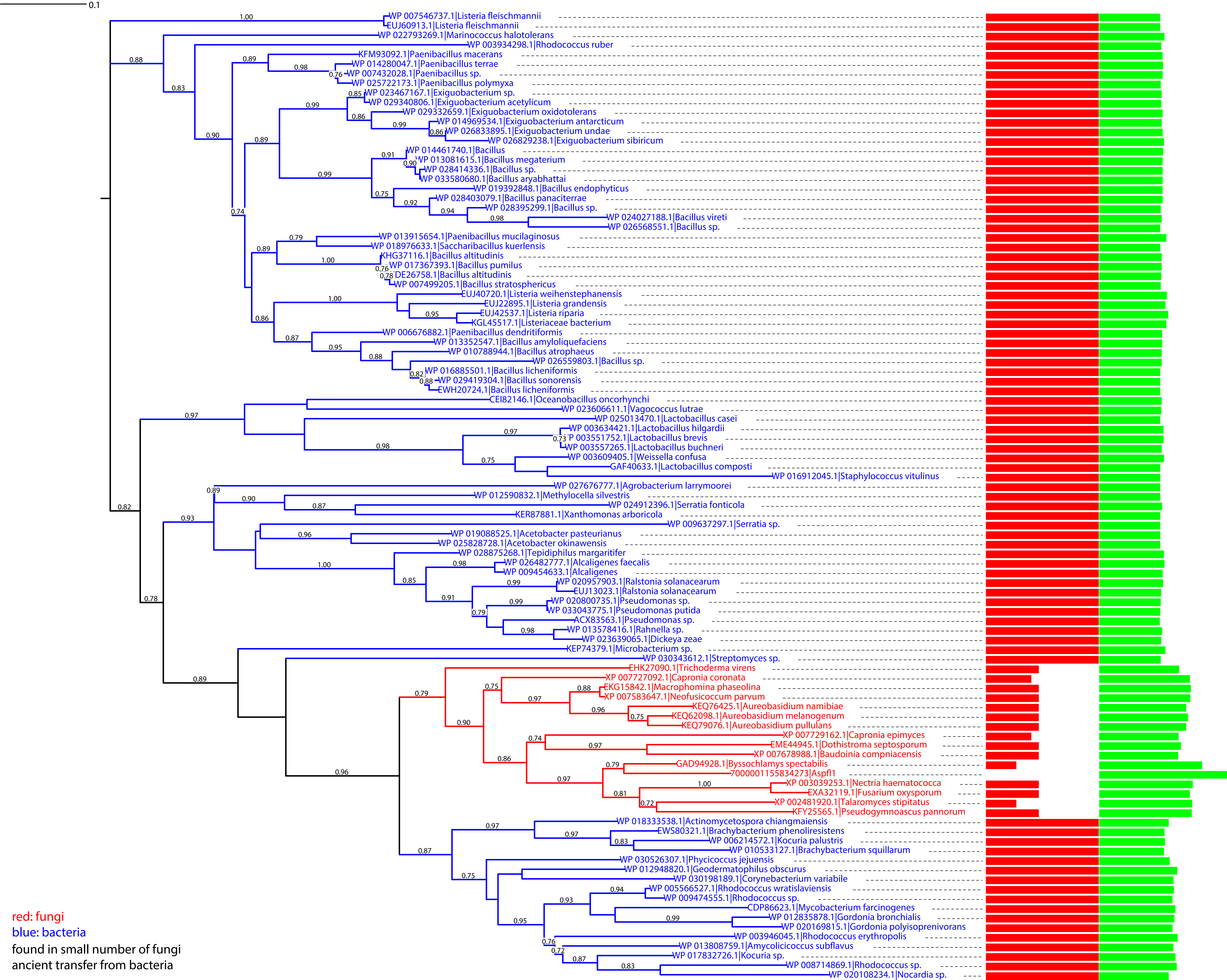
red: fungi
blue: bacteria
found in small number of fungi
ancient transfer from bacteria

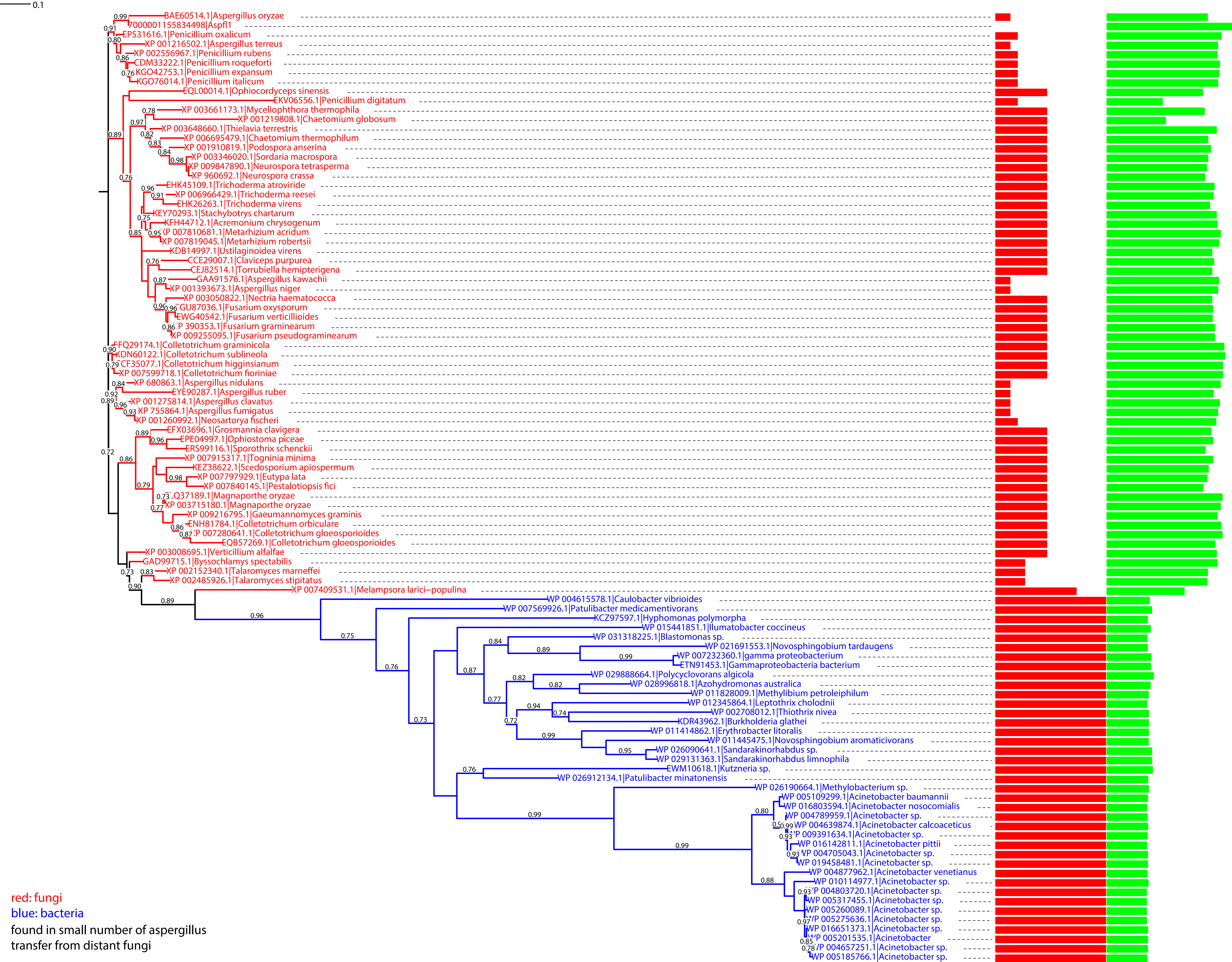

0.1
BAE60514.1|Aspergillus oryzae
7000001155834498|Aspfl1
EPS31616.1|Penicillium oxalicum
XP 001216502.1|Aspergillus terreus
XP 002556967.1|Penicillium rubens
CDM33222.1|Penicillium roqueforti
KGO42753.1|Penicillium expansum
KGO76014.1|Penicillium italicum
EQL00014.1|Ophiocordyceps sinensis
EKV06556.1|Penicillium digitatum
XP 003661173.1|Myceliophthora thermophila
XP 001219808.1|Chaetomium globosum
XP 003648660.1|Thielavia terrestris
XP 006695479.1|Chaetomium thermophilum
XP 001910819.1|Podospora anserina
XP 003346020.1|Sordaria macrospora
XP 009847890.1|Neurospora tetrasperma
XP 960692.1|Neurospora crassa
EHK45109.1|Trichoderma atroviride
XP 006966429.1|Trichoderma reesei
EHK26263.1|Trichoderma virens
KEY70293.1|Stachybotrys chartarum
KFH44712.1|Acremonium chrysogenum
XP 007810681.1|Metarhizium acridum
XP 007819045.1|Metarhizium robertsii
KDB14997.1|Ustilaginoidea virens
CCE29007.1|Claviceps purpurea
CEJ82514.1|Torrubiella hemipterigena
GAA91576.1|Aspergillus kawachii
XP 001393673.1|Aspergillus niger
XP 003050822.1|Nectria haematococca
GU87036.1|Fusarium oxysporum
EWG40542.1|Fusarium verticillioides
P 390353.1|Fusarium graminearum
XP 009255095.1|Fusarium pseudograminearum
FQ29174.1|Colletotrichum graminicola
KDN60122.1|Colletotrichum sublineola
CF35077.1|Colletotrichum higginsianum
XP 007599718.1|Colletotrichum fioriniae
XP 680863.1|Aspergillus nidulans
EYE90287.1|Aspergillus ruber
XP 001275814.1|Aspergillus clavatus
XP 755864.1|Aspergillus fumigatus
XP 001260992.1|Neosartorya fischeri
EFX03696.1|Grosmannia clavigera
EPE04997.1|Ophiostoma piceae
ERS99116.1|Sporothrix schenckii
XP 007915317.1|Togninia minima
KEZ38622.1|Scedosporium apiospermum
XP 007797929.1|Eutypa lata
XP 007840145.1|Pestalotiopsis fici
Q37189.1|Magnaporthe oryzae
XP 003715180.1|Magnaporthe oryzae
XP 009216795.1|Gaeumannomyces graminis
ENH81784.1|Colletotrichum orbiculare
P 007280641.1|Colletotrichum gloeosporioides
EQB57269.1|Colletotrichum gloeosporioides
XP 003008695.1|Verticillium alfalfae
GAD99715.1|Byssochlamys spectabilis
XP 002152340.1|Talaromyces marneffei
XP 002485926.1|Talaromyces stipitatus
XP 007409531.1|Melampsora larici–populina
WP 004615578.1|Caulobacter vibrioides
WP 007569926.1|Patulibacter medicamentivorans
KCZ97597.1|Hyphomonas polymorpha
WP 015441851.1|Ilumatobacter coccineus
WP 031318225.1|Blastomonas sp.
WP 021691553.1|Novosphingobium tardaugens
WP 007232360.1|gamma proteobacterium
ETN91453.1|Gammaproteobacteria bacterium
WP 029888664.1|Polycyclovorans algicola
WP 028996818.1|Azohydromonas australica
WP 011828009.1|Methylibium petroleiphilum
WP 012345864.1|Leptothrix cholodnii
WP 002708012.1|Thiothrix nivea
KDR43962.1|Burkholderia glathei
WP 011414862.1|Erythrobacter litoralis
WP 011445475.1|Novosphingobium aromaticivorans
WP 026090641.1|Sandarakinorhabdus sp.
WP 029131363.1|Sandarakinorhabdus limnophila
EWM10618.1|Kutzneria sp.
WP 026912134.1|Patulibacter minatonensis
WP 026190664.1|Methylobacterium sp.
WP 005109299.1|Acinetobacter baumannii
WP 016803594.1|Acinetobacter nosocomialis
WP 004789959.1|Acinetobacter sp.
WP 004639874.1|Acinetobacter calcoaceticus
P 009391634.1|Acinetobacter sp.
WP 016142811.1|Acinetobacter pittii
WP 004705043.1|Acinetobacter sp.
WP 019458481.1|Acinetobacter sp.
WP 004877962.1|Acinetobacter venetianus
WP 010114977.1|Acinetobacter sp.
P 004803720.1|Acinetobacter sp.
WP 005317455.1|Acinetobacter sp.
WP 005260089.1|Acinetobacter sp.
P 005275636.1|Acinetobacter sp.
WP 016651373.1|Acinetobacter sp.
P 005201535.1|Acinetobacter
P 004657251.1|Acinetobacter sp.
WP 005185766.1|Acinetobacter sp.
red: fungi
blue: bacteria
found in small number of aspergillus
transfer from distant fungi

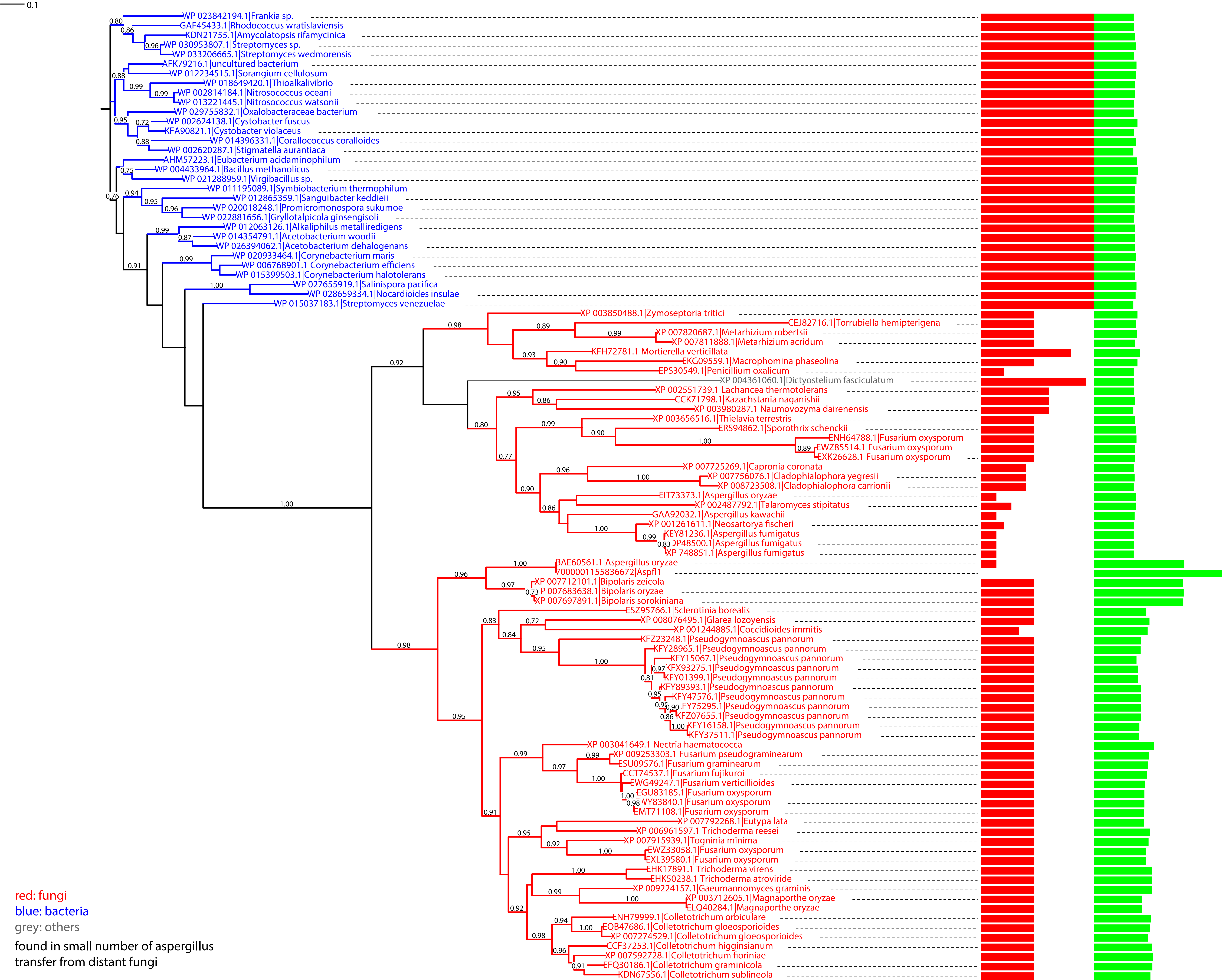

0.1
WP 023842194.1|Frankia sp.
GAF45433.1|Rhodococcus wratislaviensis
KDN21755.1|Amycolatopsis rifamycinica
WP 030953807.1|Streptomyces sp.
WP 033206665.1|Streptomyces wedmorensis
AFK79216.1|uncultured bacterium
WP 012234515.1|Sorangium cellulosum
WP 018649420.1|Thioalkalivibrio
WP 002814184.1|Nitrosococcus oceani
WP 013221445.1|Nitrosococcus watsonii
WP 029755832.1|Oxalobacteraceae bacterium
WP 002624138.1|Cystobacter fuscus
KFA90821.1|Cystobacter violaceus
WP 014396331.1|Corallococcus coralloides
WP 002620287.1|Stigmatella aurantiaca
AHM57223.1|Eubacterium acidaminophilum
WP 004433964.1|Bacillus methanolicus
WP 021288959.1|Virgibacillus sp.
WP 011195089.1|Symbiobacterium thermophilum
WP 012865359.1|Sanguibacter keddieii
WP 020018248.1|Promicromonospora sukumoe
WP 022881656.1|Gryllotalpicola ginsengisoli
WP 012063126.1|Alkaliphilus metalliredigens
WP 014354791.1|Acetobacterium woodii
WP 026394062.1|Acetobacterium dehalogenans
WP 020933464.1|Corynebacterium maris
WP 006768901.1|Corynebacterium efficiens
WP 015399503.1|Corynebacterium halotolerans
WP 027655919.1|Salinispora pacifica
WP 028659334.1|Nocardioides insulae
WP 015037183.1|Streptomyces venezuelae
XP 003850488.1|Zymoseptoria tritici
CEJ82716.1|Torrubiella hemipterigena
XP 007820687.1|Metarhizium robertsii
XP 007811888.1|Metarhizium acridum
KFH72781.1|Mortierella verticillata
EKG09559.1|Macrophomina phaseolina
EPS30549.1|Penicillium oxalicum
XP 004361060.1|Dictyostelium fasciculatum
XP 002551739.1|Lachancea thermotolerans
CCK71798.1|Kazachstania naganishii
XP 003980287.1|Naumovozyma dairenensis
XP 003656516.1|Thielavia terrestris
ERS94862.1|Sporothrix schenckii
ENH64788.1|Fusarium oxysporum
EWZ85514.1|Fusarium oxysporum
EXK26628.1|Fusarium oxysporum
XP 007725269.1|Capronia coronata
XP 007756076.1|Cladophialophora yegresii
XP 008723508.1|Cladophialophora carrionii
EIT73373.1|Aspergillus oryzae
XP 002487792.1|Talaromyces stipitatus
GAA92032.1|Aspergillus kawachii
XP 001261611.1|Neosartorya fischeri
KEY81236.1|Aspergillus fumigatus
P48500.1|Aspergillus fumigatus
XP 748851.1|Aspergillus fumigatus
BAE60561.1|Aspergillus oryzae
7000001155836672|Aspfl1
XP 007712101.1|Bipolaris zeicola
P 007683638.1|Bipolaris oryzae
XP 007697891.1|Bipolaris sorokiniana
ESZ95766.1|Sclerotinia borealis
XP 008076495.1|Glarea lozoyensis
XP 001244885.1|Coccidioides immitis
KFZ23248.1|Pseudogymnoascus pannorum
KFY28965.1|Pseudogymnoascus pannorum
KFY15067.1|Pseudogymnoascus pannorum
KFX93275.1|Pseudogymnoascus pannorum
KFY01399.1|Pseudogymnoascus pannorum
KFY89393.1|Pseudogymnoascus pannorum
KFY47576.1|Pseudogymnoascus pannorum
FY75295.1|Pseudogymnoascus pannorum
KFZ07655.1|Pseudogymnoascus pannorum
KFY16158.1|Pseudogymnoascus pannorum
KFY37511.1|Pseudogymnoascus pannorum
XP 003041649.1|Nectria haematococca
XP 009253303.1|Fusarium pseudograminearum
ESU09576.1|Fusarium graminearum
CCT74537.1|Fusarium fujikuroi
EWG49247.1|Fusarium verticillioides
EGU83185.1|Fusarium oxysporum
WY83840.1|Fusarium oxysporum
EMT71108.1|Fusarium oxysporum
XP 007792268.1|Eutypa lata
XP 006961597.1|Trichoderma reesei
XP 007915939.1|Togninia minima
EWZ33058.1|Fusarium oxysporum
EXL39580.1|Fusarium oxysporum
EHK17891.1|Trichoderma virens
EHK50238.1|Trichoderma atroviride
XP 009224157.1|Gaeumannomyces graminis
XP 003712605.1|Magnaporthe oryzae
ELQ40284.1|Magnaporthe oryzae
ENH79999.1|Colletotrichum orbiculare
EQB47686.1|Colletotrichum gloeosporioides
XP 007274529.1|Colletotrichum gloeosporioides
CCF37253.1|Colletotrichum higginsianum
XP 007592728.1|Colletotrichum fioriniae
EFQ30186.1|Colletotrichum graminicola
KDN67556.1|Colletotrichum sublineola
red: fungi
blue: bacteria
grey: others
found in small number of aspergillus
transfer from distant fungi

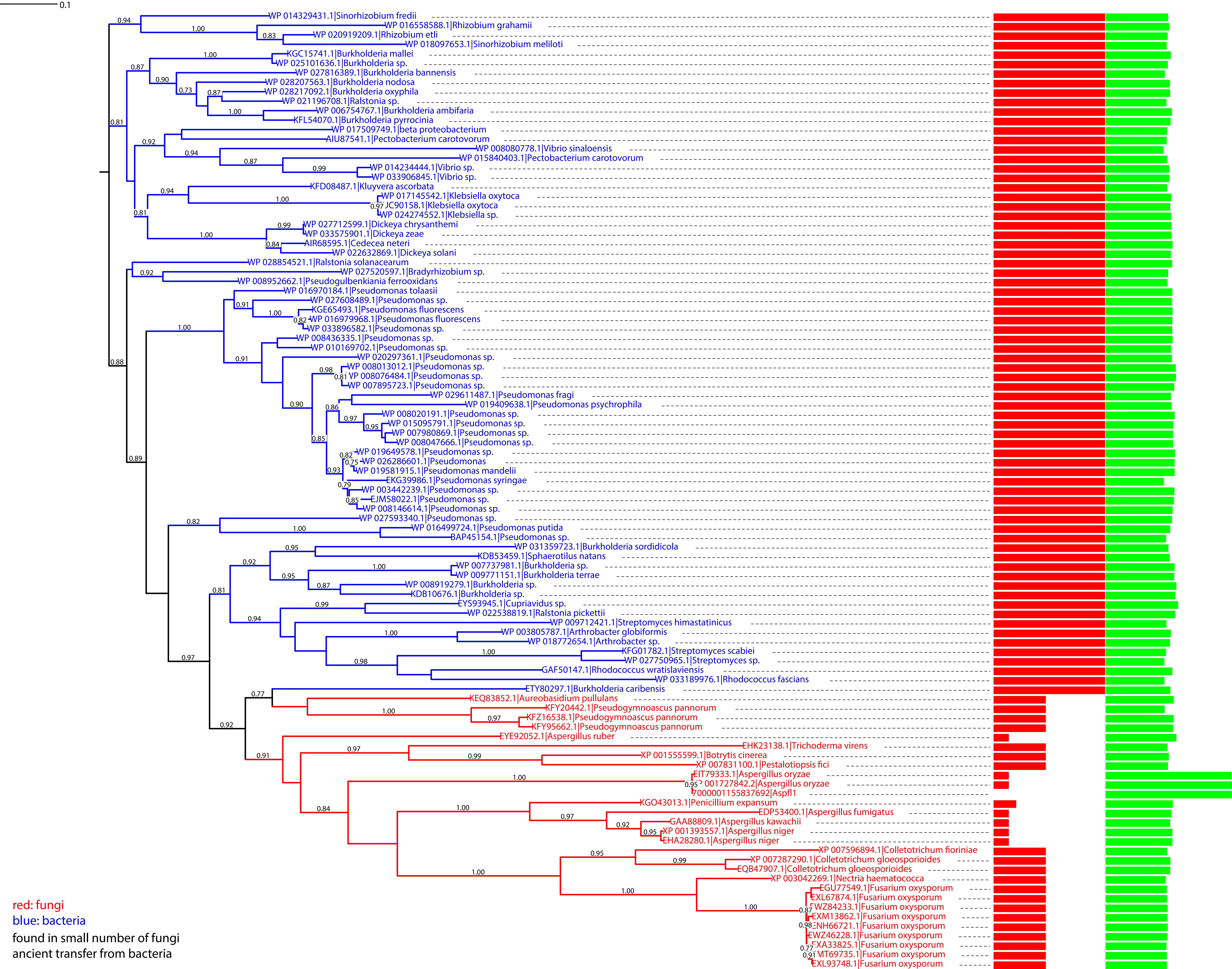
0.1
WP 014329431.1|Sinorhizobium fredii
WP 016558588.1|Rhizobium grahamii
WP 020919209.1|Rhizobium etli
WP 018097653.1|Sinorhizobium meliloti
KGC15741.1|Burkholderia mallei
WP 025101636.1|Burkholderia sp.
WP 027816389.1|Burkholderia bannensis
WP 028207563.1|Burkholderia nodosa
WP 028217092.1|Burkholderia oxyphila
WP 021196708.1|Ralstonia sp.
WP 006754767.1|Burkholderia ambifaria
KFL54070.1|Burkholderia pyrrocinia
WP 017509749.1|beta proteobacterium
AIU87541.1|Pectobacterium carotovorum
WP 008080778.1|Vibrio sinaloensis
WP 015840403.1|Pectobacterium carotovorum
WP 014234444.1|Vibrio sp.
WP 033906845.1|Vibrio sp.
KFD08487.1|Kluyvera ascorbata
WP 017145542.1|Klebsiella oxytoca
JC90158.1|Klebsiella oxytoca
WP 024274552.1|Klebsiella sp.
WP 027712599.1|Dickeya chrysanthemi
WP 033575901.1|Dickeya zeae
AIR68595.1|Cedecea neteri
WP 022632869.1|Dickeya solani
WP 028854521.1|Ralstonia solanacearum
WP 027520597.1|Bradyrhizobium sp.
WP 008952662.1|Pseudogulbenkiania ferrooxidans
WP 016970184.1|Pseudomonas tolaasii
WP 027608489.1|Pseudomonas sp.
KGE65493.1|Pseudomonas fluorescens
WP 016979968.1|Pseudomonas fluorescens
WP 033896582.1|Pseudomonas sp.
WP 008436335.1|Pseudomonas sp.
WP 010169702.1|Pseudomonas sp.
WP 020297361.1|Pseudomonas sp.
WP 008013012.1|Pseudomonas sp.
VP 008076484.1|Pseudomonas sp.
WP 007895723.1|Pseudomonas sp.
WP 029611487.1|Pseudomonas fragi
WP 019409638.1|Pseudomonas psychrophila
WP 008020191.1|Pseudomonas sp.
WP 015095791.1|Pseudomonas sp.
WP 007980869.1|Pseudomonas sp.
WP 008047666.1|Pseudomonas sp.
WP 019649578.1|Pseudomonas sp.
WP 026286601.1|Pseudomonas
WP 019581915.1|Pseudomonas mandelii
EKG39986.1|Pseudomonas syringae
WP 003442239.1|Pseudomonas sp.
EJM58022.1|Pseudomonas sp.
WP 008146614.1|Pseudomonas sp.
WP 027593340.1|Pseudomonas sp.
WP 016499724.1|Pseudomonas putida
BAP45154.1|Pseudomonas sp.
WP 031359723.1|Burkholderia sordidicola
KDB53459.1|Sphaerotilus natans
WP 007737981.1|Burkholderia sp.
WP 009771151.1|Burkholderia terrae
WP 008919279.1|Burkholderia sp.
KDB10676.1|Burkholderia sp.
EYS93945.1|Cupriavidus sp.
WP 022538819.1|Ralstonia pickettii
WP 009712421.1|Streptomyces himastatinicus
WP 003805787.1|Arthrobacter globiformis
WP 018772654.1|Arthrobacter sp.
KFG01782.1|Streptomyces scabiei
WP 027750965.1|Streptomyces sp.
GAF50147.1|Rhodococcus wratislaviensis
WP 033189976.1|Rhodococcus fascians
ETY80297.1|Burkholderia caribensis
KEQ83852.1|Aureobasidium pullulans
KFY20442.1|Pseudogymnoascus pannorum
KFZ16538.1|Pseudogymnoascus pannorum
KFY95662.1|Pseudogymnoascus pannorum
EYE92052.1|Aspergillus ruber
EHK23138.1|Trichoderma virens
XP 001555599.1|Botrytis cinerea
XP 007831100.1|Pestalotiopsis fici
EIT79333.1|Aspergillus oryzae
001727842.2|Aspergillus oryzae
7000001155837692|Aspfl1
KGO43013.1|Penicillium expansum
EDP53400.1|Aspergillus fumigatus
GAA88809.1|Aspergillus kawachii
XP 001393557.1|Aspergillus niger
EHA28280.1|Aspergillus niger
XP 007596894.1|Colletotrichum fioriniae
XP 007287290.1|Colletotrichum gloeosporioides
EQB47907.1|Colletotrichum gloeosporioides
XP 003042269.1|Nectria haematococca
EGU77549.1|Fusarium oxysporum
EXL67874.1|Fusarium oxysporum
WZ84233.1|Fusarium oxysporum
EXM13862.1|Fusarium oxysporum
NH66721.1|Fusarium oxysporum
EWZ46228.1|Fusarium oxysporum
XA33825.1|Fusarium oxysporum
MT69735.1|Fusarium oxysporum
EXL93748.1|Fusarium oxysporum
red: fungi
blue: bacteria
found in small number of fungi
ancient transfer from bacteria

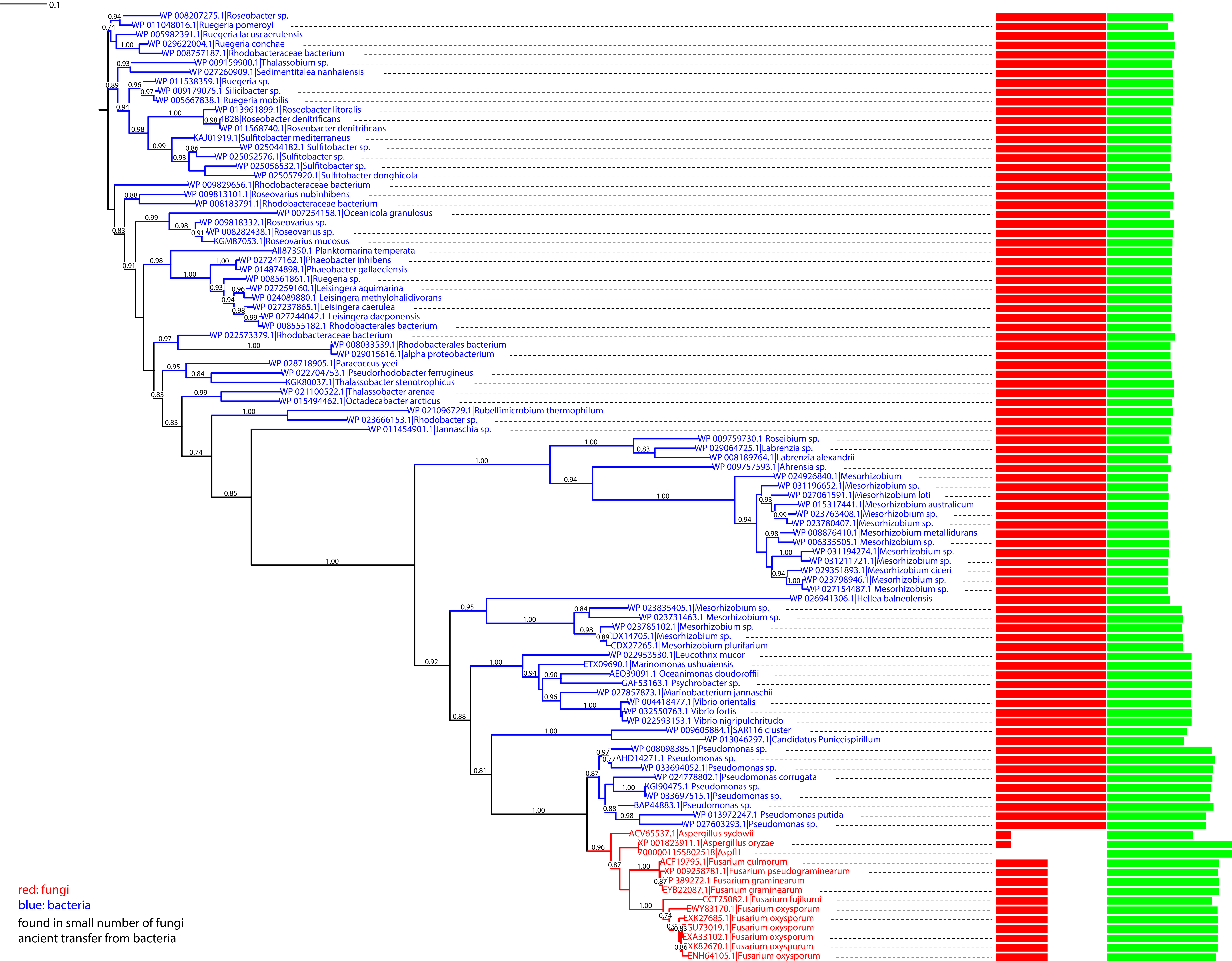
0.1
WP 008207275.1|Roseobacter sp.
WP 011048016.1|Ruegeria pomeroyi
WP 005982391.1|Ruegeria lacuscaerulensis
WP 029622004.1|Ruegeria conchae
WP 008757187.1|Rhodobacteraceae bacterium
WP 009159900.1|Thalassobium sp.
WP 027260909.1|Sedimentitalea nanhaiensis
WP 011538359.1|Ruegeria sp.
WP 009179075.1|Silicibacter sp.
WP 005667838.1|Ruegeria mobilis
WP 013961899.1|Roseobacter litoralis
4B28|Roseobacter denitrificans
WP 011568740.1|Roseobacter denitrificans
KAJ01919.1|Sulfitobacter mediterraneus
WP 025044182.1|Sulfitobacter sp.
WP 025052576.1|Sulfitobacter sp.
WP 025056532.1|Sulfitobacter sp.
WP 025057920.1|Sulfitobacter donghicola
WP 009829656.1|Rhodobacteraceae bacterium
WP 009813101.1|Roseovarius nubinhibens
WP 008183791.1|Rhodobacteraceae bacterium
WP 007254158.1|Oceanicola granulosus
WP 009818332.1|Roseovarius sp.
WP 008282438.1|Roseovarius sp.
KGM87053.1|Roseovarius mucosus
AII87350.1|Planktomarina temperata
WP 027247162.1|Phaeobacter inhibens
WP 014874898.1|Phaeobacter gallaeciensis
WP 008561861.1|Ruegeria sp.
WP 027259160.1|Leisingera aquimarina
WP 024089880.1|Leisingera methylohalidivorans
WP 027237865.1|Leisingera caerulea
WP 027244042.1|Leisingera daeponensis
WP 008555182.1|Rhodobacterales bacterium
WP 022573379.1|Rhodobacteraceae bacterium
WP 008033539.1|Rhodobacterales bacterium
WP 029015616.1|alpha proteobacterium
WP 028718905.1|Paracoccus yeei
WP 022704753.1|Pseudorhodobacter ferrugineus
KGK80037.1|Thalassobacter stenotrophicus
WP 021100522.1|Thalassobacter arenae
WP 015494462.1|Octadecabacter arcticus
WP 021096729.1|Rubellimicrobium thermophilum
WP 023666153.1|Rhodobacter sp.
WP 011454901.1|Jannaschia sp.
WP 009759730.1|Roseibium sp.
WP 029064725.1|Labrenzia sp.
WP 008189764.1|Labrenzia alexandrii
WP 009757593.1|Ahrensia sp.
WP 024926840.1|Mesorhizobium
WP 031196652.1|Mesorhizobium sp.
WP 027061591.1|Mesorhizobium loti
WP 015317441.1|Mesorhizobium australicum
WP 023763408.1|Mesorhizobium sp.
WP 023780407.1|Mesorhizobium sp.
WP 008876410.1|Mesorhizobium metallidurans
WP 006335505.1|Mesorhizobium sp.
WP 031194274.1|Mesorhizobium sp.
WP 031211721.1|Mesorhizobium sp.
WP 029351893.1|Mesorhizobium ciceri
WP 023798946.1|Mesorhizobium sp.
WP 027154487.1|Mesorhizobium sp.
WP 026941306.1|Hellea balneolensis
WP 023835405.1|Mesorhizobium sp.
WP 023731463.1|Mesorhizobium sp.
WP 023785102.1|Mesorhizobium sp.
DX14705.1|Mesorhizobium sp.
CDX27265.1|Mesorhizobium plurifarium
WP 022953530.1|Leucothrix mucor
ETX09690.1|Marinomonas ushuaiensis
AEQ39091.1|Oceanimonas doudoroffii
GAF53163.1|Psychrobacter sp.
WP 027857873.1|Marinobacterium jannaschii
WP 004418477.1|Vibrio orientalis
WP 032550763.1|Vibrio fortis
WP 022593153.1|Vibrio nigripulchritudo
WP 009605884.1|SAR116 cluster
WP 013046297.1|Candidatus Puniceispirillum
WP 008098385.1|Pseudomonas sp.
AHD14271.1|Pseudomonas sp.
WP 033694052.1|Pseudomonas sp.
WP 024778802.1|Pseudomonas corrugata
KGI90475.1|Pseudomonas sp.
WP 033697515.1|Pseudomonas sp.
BAP44883.1|Pseudomonas sp.
WP 013972247.1|Pseudomonas putida
WP 027603293.1|Pseudomonas sp.
ACV65537.1|Aspergillus sydowii
XP 001823911.1|Aspergillus oryzae
7000001155802518|Aspfl1
ACF19795.1|Fusarium culmorum
XP 009258781.1|Fusarium pseudograminearum
P 389272.1|Fusarium graminearum
EYB22087.1|Fusarium graminearum
CCT75082.1|Fusarium fujikuroi
EWY83170.1|Fusarium oxysporum
EXK27685.1|Fusarium oxysporum
GU73019.1|Fusarium oxysporum
EXA33102.1|Fusarium oxysporum
EXK82670.1|Fusarium oxysporum
ENH64105.1|Fusarium oxysporum
red: fungi
blue: bacteria
found in small number of fungi
ancient transfer from bacteria

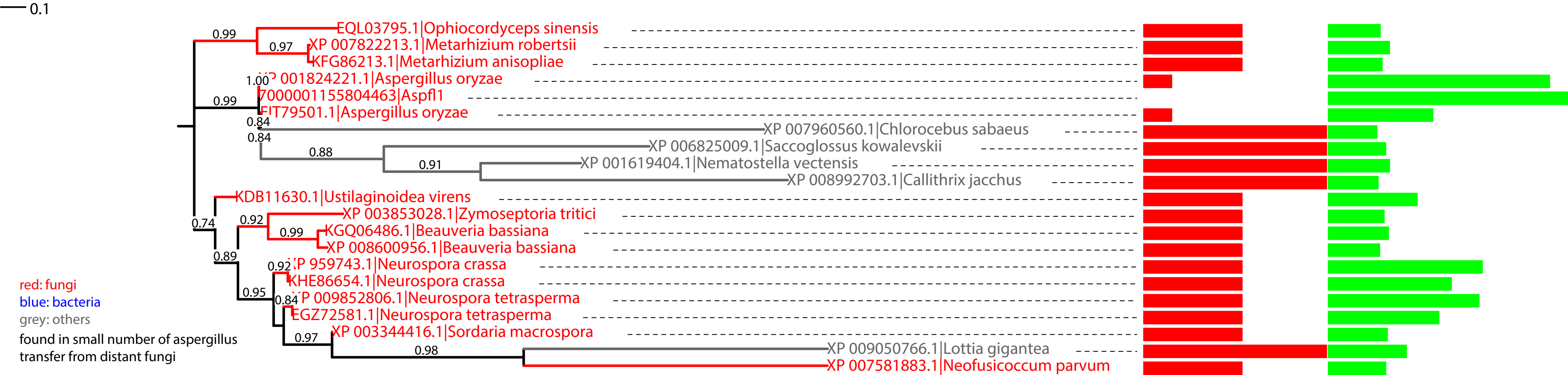
0.1
EQL03795.1|Ophiocordyceps sinensis
XP 007822213.1|Metarhizium robertsii
KFG86213.1|Metarhizium anisopliae
XP 001824221.1|Aspergillus oryzae
7000001155804463|Aspfl1
EIT79501.1|Aspergillus oryzae
XP 007960560.1|Chlorocebus sabaeus
XP 006825009.1|Saccoglossus kowalevskii
XP 001619404.1|Nematostella vectensis
XP 008992703.1|Callithrix jacchus
KDB11630.1|Ustilaginoidea virens
XP 003853028.1|Zymoseptoria tritici
KGQ06486.1|Beauveria bassiana
XP 008600956.1|Beauveria bassiana
XP 959743.1|Neurospora crassa
KHE86654.1|Neurospora crassa
XP 009852806.1|Neurospora tetrasperma
EGZ72581.1|Neurospora tetrasperma
XP 003344416.1|Sordaria macrospora
XP 009050766.1|Lottia gigantea
XP 007581883.1|Neofusicoccum parvum
0.99
0.97
1.00
0.99
0.84
0.84
0.88
0.91
0.74
0.92
0.99
0.89
0.92
0.95
0.84
0.97
0.98
red: fungi
blue: bacteria
grey: others
found in small number of aspergillus
transfer from distant fungi

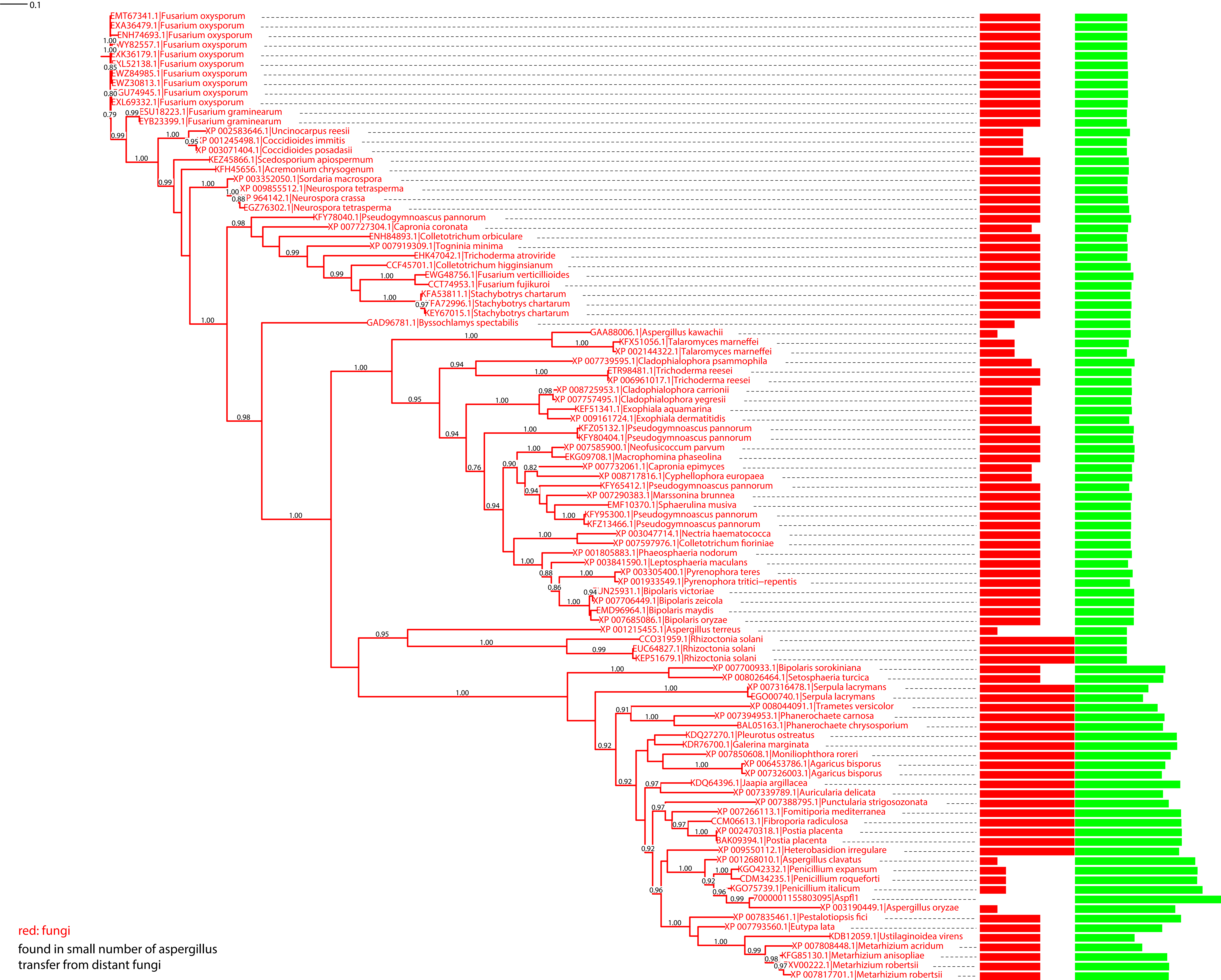

red: fungi

found in small number of aspergillus
transfer from distant fungi

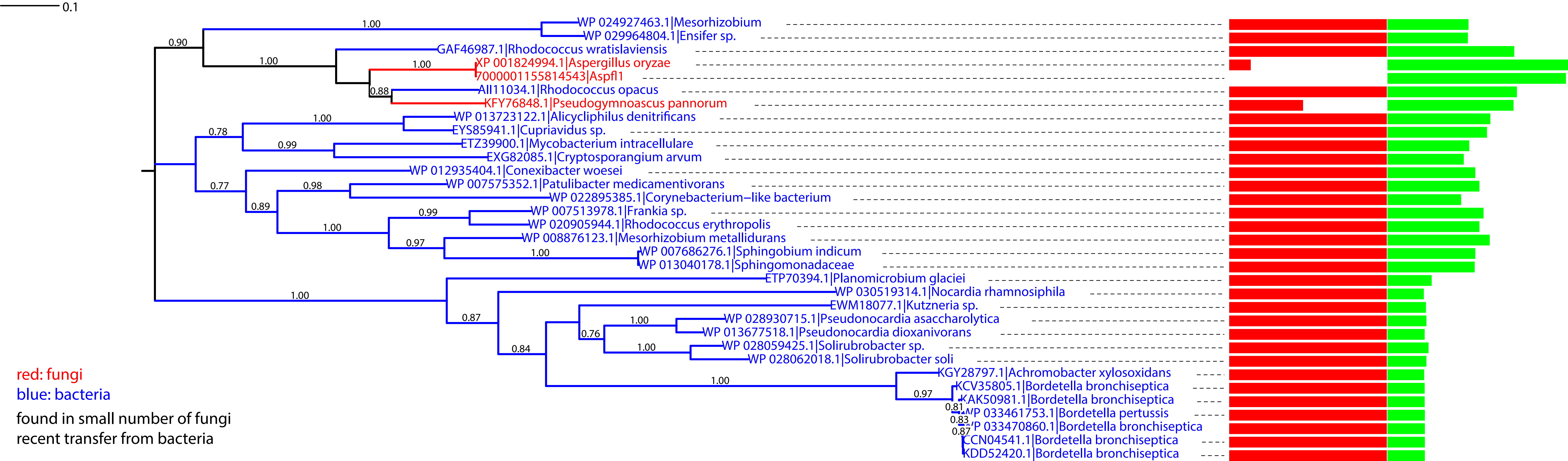
0.1
WP 024927463.1|Mesorhizobium
WP 029964804.1|Ensifer sp.
GAF46987.1|Rhodococcus wratislaviensis
XP 001824994.1|Aspergillus oryzae
7000001155814543|Aspfl1
AII11034.1|Rhodococcus opacus
KFY76848.1|Pseudogymnoascus pannorum
WP 013723122.1|Alicycliphilus denitrificans
EYS85941.1|Cupriavidus sp.
ETZ39900.1|Mycobacterium intracellulare
EXG82085.1|Cryptosporangium arvum
WP 012935404.1|Conexibacter woesei
WP 007575352.1|Patulibacter medicamentivorans
WP 022895385.1|Corynebacterium–like bacterium
WP 007513978.1|Frankia sp.
WP 020905944.1|Rhodococcus erythropolis
WP 008876123.1|Mesorhizobium metallidurans
WP 007686276.1|Sphingobium indicum
WP 013040178.1|Sphingomonadaceae
ETP70394.1|Planomicrobium glaciei
WP 030519314.1|Nocardia rhamnosiphila
EWM18077.1|Kutzneria sp.
WP 028930715.1|Pseudonocardia asaccharolytica
WP 013677518.1|Pseudonocardia dioxanivorans
WP 028059425.1|Solirubrobacter sp.
WP 028062018.1|Solirubrobacter soli
KGY28797.1|Achromobacter xylosoxidans
KCV35805.1|Bordetella bronchiseptica
KAK50981.1|Bordetella bronchiseptica
WP 033461753.1|Bordetella pertussis
P 033470860.1|Bordetella bronchiseptica
CCN04541.1|Bordetella bronchiseptica
KDD52420.1|Bordetella bronchiseptica
red: fungi
blue: bacteria
found in small number of fungi
recent transfer from bacteria

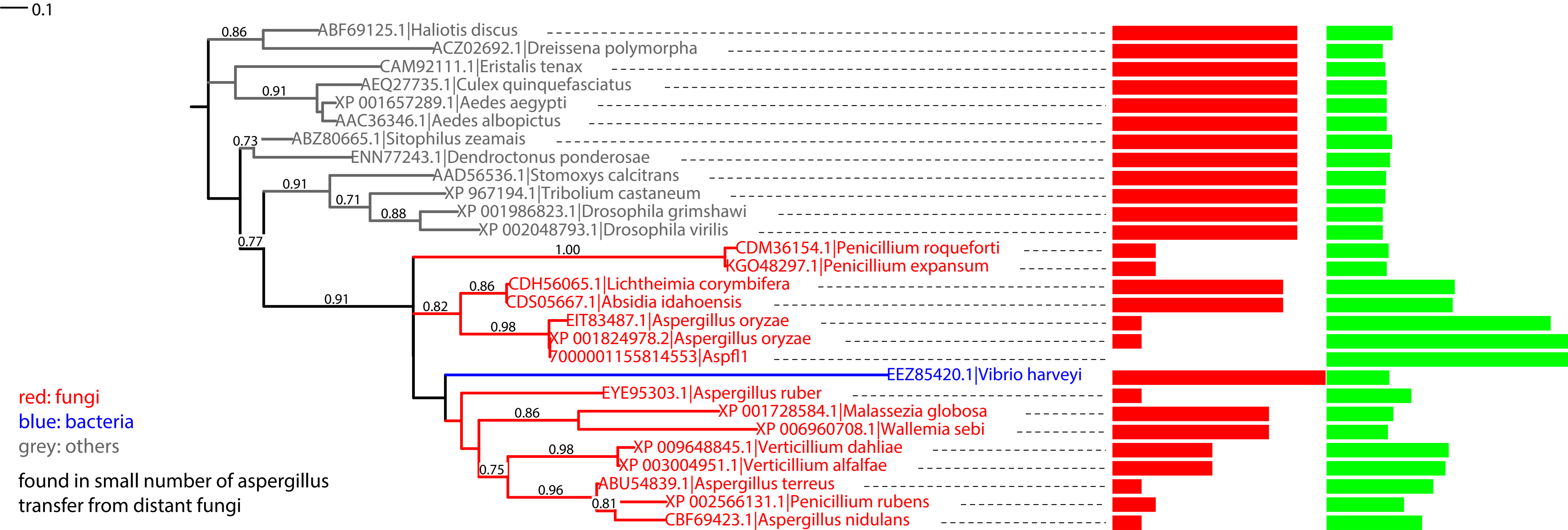

0.1
ABF69125.1|Haliotis discus
ACZ02692.1|Dreissena polymorpha
CAM92111.1|Eristalis tenax
AEQ27735.1|Culex quinquefasciatus
XP 001657289.1|Aedes aegypti
AAC36346.1|Aedes albopictus
ABZ80665.1|Sitophilus zeamais
ENN77243.1|Dendroctonus ponderosae
AAD56536.1|Stomoxys calcitrans
XP 967194.1|Tribolium castaneum
XP 001986823.1|Drosophila grimshawi
XP 002048793.1|Drosophila virilis
CDM36154.1|Penicillium roqueforti
KGO48297.1|Penicillium expansum
CDH56065.1|Lichtheimia corymbifera
CDS05667.1|Absidia idahoensis
EIT83487.1|Aspergillus oryzae
XP 001824978.2|Aspergillus oryzae
7000001155814553|Aspfl1
EEZ85420.1|Vibrio harveyi
EYE95303.1|Aspergillus ruber
XP 001728584.1|Malassezia globosa
XP 006960708.1|Wallemia sebi
XP 009648845.1|Verticillium dahliae
XP 003004951.1|Verticillium alfalfae
ABU54839.1|Aspergillus terreus
XP 002566131.1|Penicillium rubens
CBF69423.1|Aspergillus nidulans
0.86
0.91
0.73
0.91
0.71
0.88
0.77
1.00
0.86
0.91
0.82
0.98
0.86
0.98
0.75
0.96
0.81
red: fungi
blue: bacteria
grey: others
found in small number of aspergillus
transfer from distant fungi

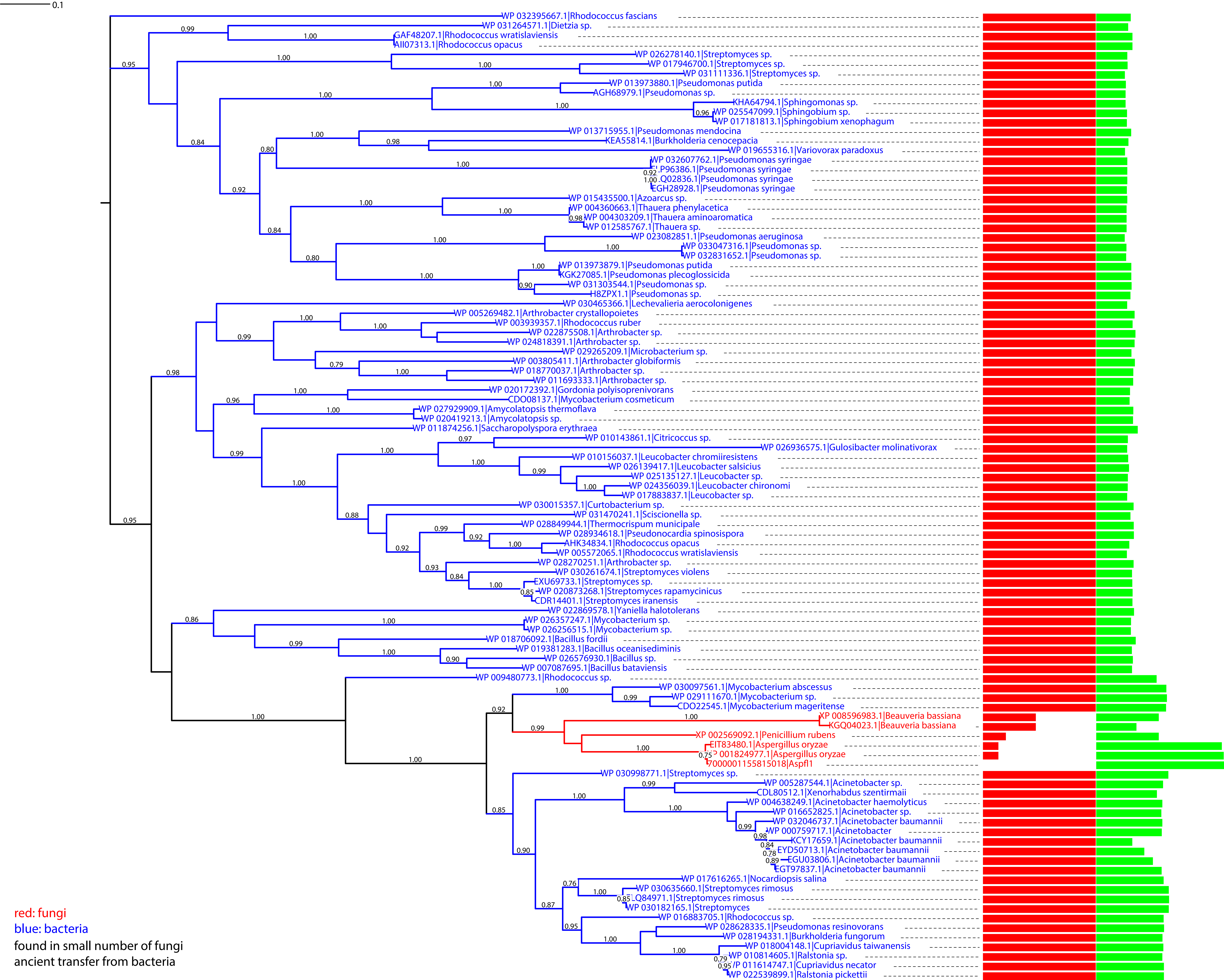

0.1
WP 032395667.1|Rhodococcus fascians
WP 031264571.1|Dietzia sp.
GAF48207.1|Rhodococcus wratislaviensis
AII07313.1|Rhodococcus opacus
WP 026278140.1|Streptomyces sp.
WP 017946700.1|Streptomyces sp.
WP 031111336.1|Streptomyces sp.
WP 013973880.1|Pseudomonas putida
AGH68979.1|Pseudomonas sp.
KHA64794.1|Sphingomonas sp.
WP 025547099.1|Sphingobium sp.
WP 017181813.1|Sphingobium xenophagum
WP 013715955.1|Pseudomonas mendocina
KEA55814.1|Burkholderia cenocepacia
WP 019655316.1|Variovorax paradoxus
WP 032607762.1|Pseudomonas syringae
P96386.1|Pseudomonas syringae
Q02836.1|Pseudomonas syringae
EGH28928.1|Pseudomonas syringae
WP 015435500.1|Azoarcus sp.
WP 004360663.1|Thauera phenylacetica
WP 004303209.1|Thauera aminoaromatica
WP 012585767.1|Thauera sp.
WP 023082851.1|Pseudomonas aeruginosa
WP 033047316.1|Pseudomonas sp.
WP 032831652.1|Pseudomonas sp.
WP 013973879.1|Pseudomonas putida
KGK27085.1|Pseudomonas plecoglossicida
WP 031303544.1|Pseudomonas sp.
H8ZPX1.1|Pseudomonas sp.
WP 030465366.1|Lechevalieria aerocolonigenes
WP 005269482.1|Arthrobacter crystallopoietes
WP 003939357.1|Rhodococcus ruber
WP 022875508.1|Arthrobacter sp.
WP 024818391.1|Arthrobacter sp.
WP 029265209.1|Microbacterium sp.
WP 003805411.1|Arthrobacter globiformis
WP 018770037.1|Arthrobacter sp.
WP 011693333.1|Arthrobacter sp.
WP 020172392.1|Gordonia polyisoprenivorans
CDO08137.1|Mycobacterium cosmeticum
WP 027929909.1|Amycolatopsis thermoflava
WP 020419213.1|Amycolatopsis sp.
WP 011874256.1|Saccharopolyspora erythraea
WP 010143861.1|Citricoccus sp.
WP 026936575.1|Gulosibacter molinativorax
WP 010156037.1|Leucobacter chromiiresistens
WP 026139417.1|Leucobacter salsicius
WP 025135127.1|Leucobacter sp.
WP 024356039.1|Leucobacter chironomi
WP 017883837.1|Leucobacter sp.
WP 030015357.1|Curtobacterium sp.
WP 031470241.1|Sciscionella sp.
WP 028849944.1|Thermocrispum municipale
WP 028934618.1|Pseudonocardia spinosispora
AHK34834.1|Rhodococcus opacus
WP 005572065.1|Rhodococcus wratislaviensis
WP 028270251.1|Arthrobacter sp.
WP 030261674.1|Streptomyces violens
EXU69733.1|Streptomyces sp.
WP 020873268.1|Streptomyces rapamycinicus
CDR14401.1|Streptomyces iranensis
WP 022869578.1|Yaniella halotolerans
WP 026357247.1|Mycobacterium sp.
WP 026256515.1|Mycobacterium sp.
WP 018706092.1|Bacillus fordii
WP 019381283.1|Bacillus oceanisediminis
WP 026576930.1|Bacillus sp.
WP 007087695.1|Bacillus bataviensis
WP 009480773.1|Rhodococcus sp.
WP 030097561.1|Mycobacterium abscessus
WP 029111670.1|Mycobacterium sp.
CDO22545.1|Mycobacterium mageritense
XP 008596983.1|Beauveria bassiana
KGQ04023.1|Beauveria bassiana
XP 002569092.1|Penicillium rubens
EIT83480.1|Aspergillus oryzae
001824977.1|Aspergillus oryzae
7000001155815018|Aspfl1
WP 030998771.1|Streptomyces sp.
WP 005287544.1|Acinetobacter sp.
CDL80512.1|Xenorhabdus szentirmaii
WP 004638249.1|Acinetobacter haemolyticus
WP 016652825.1|Acinetobacter sp.
WP 032046737.1|Acinetobacter baumannii
WP 000759717.1|Acinetobacter
KCY17659.1|Acinetobacter baumannii
EYD50713.1|Acinetobacter baumannii
EGU03806.1|Acinetobacter baumannii
EGT97837.1|Acinetobacter baumannii
WP 017616265.1|Nocardiopsis salina
WP 030635660.1|Streptomyces rimosus
Q84971.1|Streptomyces rimosus
WP 030182165.1|Streptomyces
WP 016883705.1|Rhodococcus sp.
WP 028628335.1|Pseudomonas resinovorans
WP 028194331.1|Burkholderia fungorum
WP 018004148.1|Cupriavidus taiwanensis
WP 010814605.1|Ralstonia sp.
P 011614747.1|Cupriavidus necator
WP 022539899.1|Ralstonia pickettii
red: fungi
blue: bacteria
found in small number of fungi
ancient transfer from bacteria

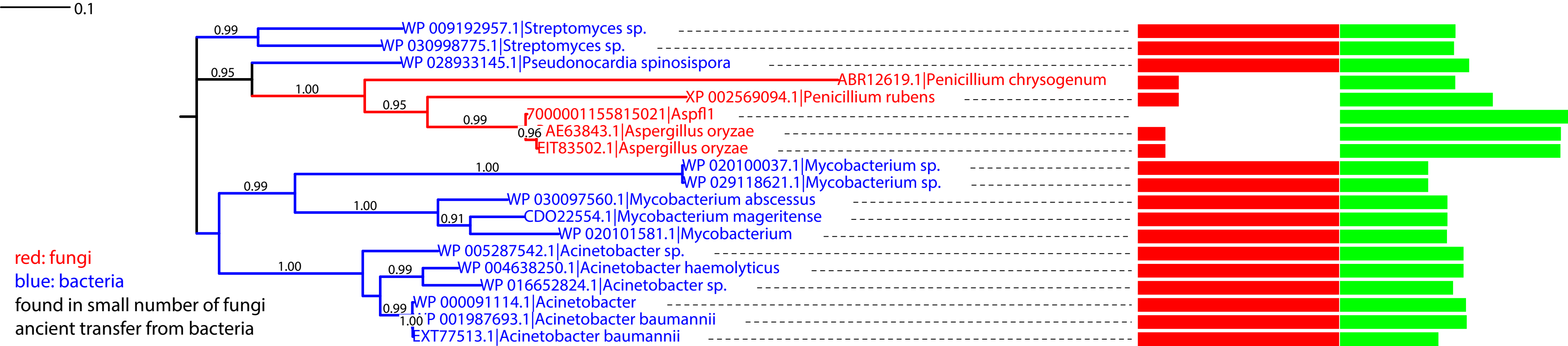

0.1
0.99
0.95
1.00
0.95
0.99
0.96
1.00
0.99
1.00
0.91
1.00
0.99
0.99
1.00
WP 009192957.1|Streptomyces sp.
WP 030998775.1|Streptomyces sp.
WP 028933145.1|Pseudonocardia spinosispora
ABR12619.1|Penicillium chrysogenum
XP 002569094.1|Penicillium rubens
7000001155815021|Aspfl1
AE63843.1|Aspergillus oryzae
EIT83502.1|Aspergillus oryzae
WP 020100037.1|Mycobacterium sp.
WP 029118621.1|Mycobacterium sp.
WP 030097560.1|Mycobacterium abscessus
CDO22554.1|Mycobacterium mageritense
WP 020101581.1|Mycobacterium
WP 005287542.1|Acinetobacter sp.
WP 004638250.1|Acinetobacter haemolyticus
WP 016652824.1|Acinetobacter sp.
WP 000091114.1|Acinetobacter
P 001987693.1|Acinetobacter baumannii
EXT77513.1|Acinetobacter baumannii
red: fungi
blue: bacteria
found in small number of fungi
ancient transfer from bacteria

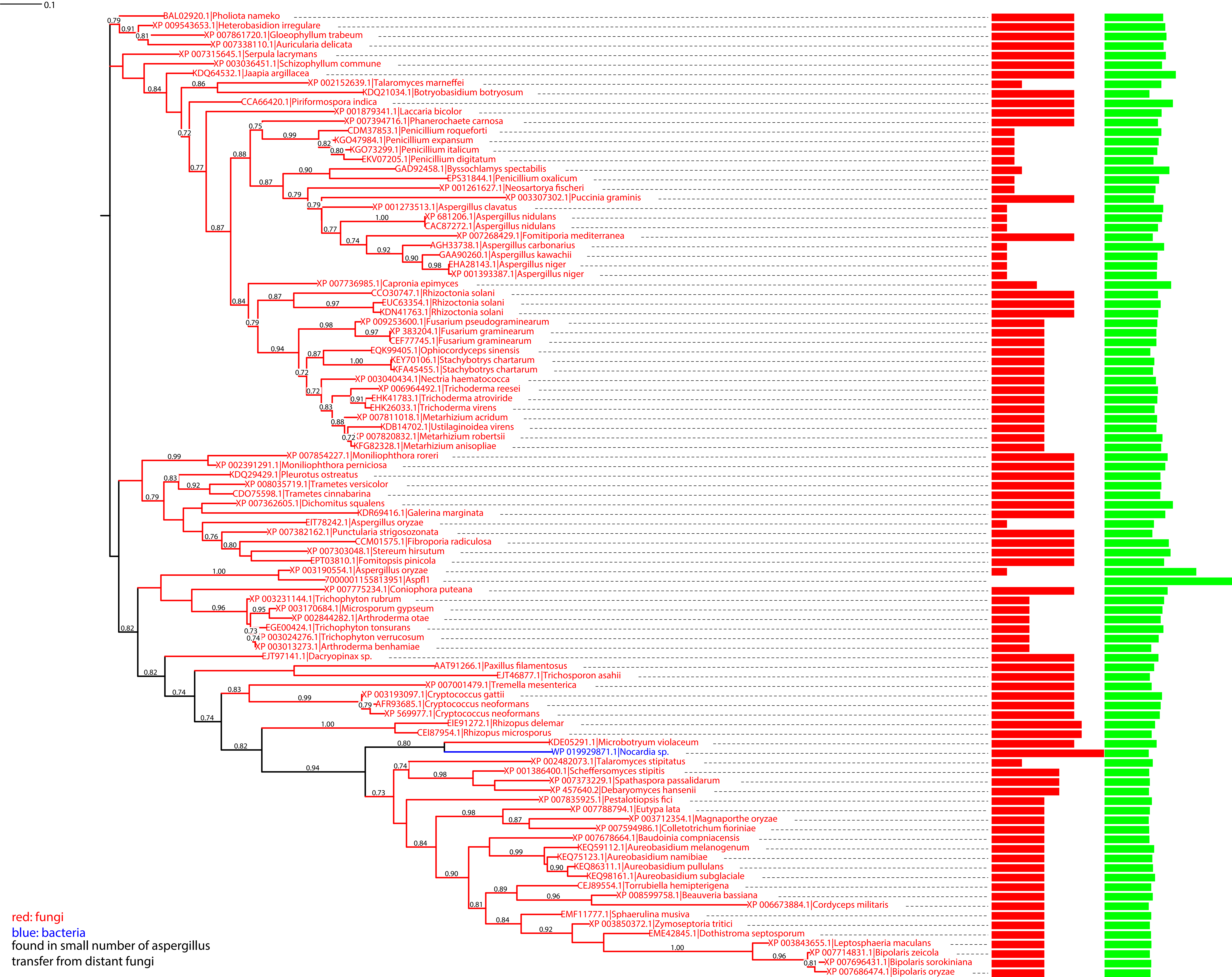
0.1
BAL02920.1|Pholiota nameko
XP 009543653.1|Heterobasidion irregulare
XP 007861720.1|Gloeophyllum trabeum
XP 007338110.1|Auricularia delicata
XP 007315645.1|Serpula lacrymans
XP 003036451.1|Schizophyllum commune
KDQ64532.1|Jaapia argillacea
XP 002152639.1|Talaromyces marneffei
KDQ21034.1|Botryobasidium botryosum
CCA66420.1|Piriformospora indica
XP 001879341.1|Laccaria bicolor
XP 007394716.1|Phanerochaete carnosa
CDM37853.1|Penicillium roqueforti
KGO47984.1|Penicillium expansum
KGO73299.1|Penicillium italicum
EKV07205.1|Penicillium digitatum
GAD92458.1|Byssochlamys spectabilis
EPS31844.1|Penicillium oxalicum
XP 001261627.1|Neosartorya fischeri
XP 003307302.1|Puccinia graminis
XP 001273513.1|Aspergillus clavatus
XP 681206.1|Aspergillus nidulans
CAC87272.1|Aspergillus nidulans
XP 007268429.1|Fomitiporia mediterranea
AGH33738.1|Aspergillus carbonarius
GAA90260.1|Aspergillus kawachii
EHA28143.1|Aspergillus niger
XP 001393387.1|Aspergillus niger
XP 007736985.1|Capronia epimyces
CCO30747.1|Rhizoctonia solani
EUC63354.1|Rhizoctonia solani
KDN41763.1|Rhizoctonia solani
XP 009253600.1|Fusarium pseudograminearum
XP 383204.1|Fusarium graminearum
CEF77745.1|Fusarium graminearum
EQK99405.1|Ophiocordyceps sinensis
KEY70106.1|Stachybotrys chartarum
KFA45455.1|Stachybotrys chartarum
XP 003040434.1|Nectria haematococca
XP 006964492.1|Trichoderma reesei
EHK41783.1|Trichoderma atroviride
EHK26033.1|Trichoderma virens
XP 007811018.1|Metarhizium acridum
KDB14702.1|Ustilaginoidea virens
XP 007820832.1|Metarhizium robertsii
KFG82328.1|Metarhizium anisopliae
XP 007854227.1|Moniliophthora roreri
XP 002391291.1|Moniliophthora perniciosa
KDQ29429.1|Pleurotus ostreatus
XP 008035719.1|Trametes versicolor
CDO75598.1|Trametes cinnabarina
XP 007362605.1|Dichomitus squalens
KDR69416.1|Galerina marginata
EIT78242.1|Aspergillus oryzae
XP 007382162.1|Punctularia strigosozonata
CCM01575.1|Fibroporia radiculosa
XP 007303048.1|Stereum hirsutum
EPT03810.1|Fomitopsis pinicola
XP 003190554.1|Aspergillus oryzae
7000001155813951|Aspfl1
XP 007775234.1|Coniophora puteana
XP 003231144.1|Trichophyton rubrum
XP 003170684.1|Microsporum gypseum
XP 002844282.1|Arthroderma otae
EGE00424.1|Trichophyton tonsurans
XP 003024276.1|Trichophyton verrucosum
XP 003013273.1|Arthroderma benhamiae
EJT97141.1|Dacryopinax sp.
AAT91266.1|Paxillus filamentosus
EJT46877.1|Trichosporon asahii
XP 007001479.1|Tremella mesenterica
XP 003193097.1|Cryptococcus gattii
AFR93685.1|Cryptococcus neoformans
XP 569977.1|Cryptococcus neoformans
EIE91272.1|Rhizopus delemar
CEI87954.1|Rhizopus microsporus
KDE05291.1|Microbotryum violaceum
WP 019929871.1|Nocardia sp.
XP 002482073.1|Talaromyces stipitatus
XP 001386400.1|Scheffersomyces stipitis
XP 007373229.1|Spathaspora passalidarum
XP 457640.2|Debaryomyces hansenii
XP 007835925.1|Pestalotiopsis fici
XP 007788794.1|Eutypa lata
XP 003712354.1|Magnaporthe oryzae
XP 007594986.1|Colletotrichum fioriniae
XP 007678664.1|Baudoinia compniacensis
KEQ59112.1|Aureobasidium melanogenum
KEQ75123.1|Aureobasidium namibiae
KEQ86311.1|Aureobasidium pullulans
KEQ98161.1|Aureobasidium subglaciale
CEJ89554.1|Torrubiella hemipterigena
XP 008599758.1|Beauveria bassiana
XP 006673884.1|Cordyceps militaris
EMF11777.1|Sphaerulina musiva
XP 003850372.1|Zymoseptoria tritici
EME42845.1|Dothistroma septosporum
XP 003843655.1|Leptosphaeria maculans
XP 007714831.1|Bipolaris zeicola
XP 007696431.1|Bipolaris sorokiniana
XP 007686474.1|Bipolaris oryzae
red: fungi
blue: bacteria
found in small number of aspergillus
transfer from distant fungi

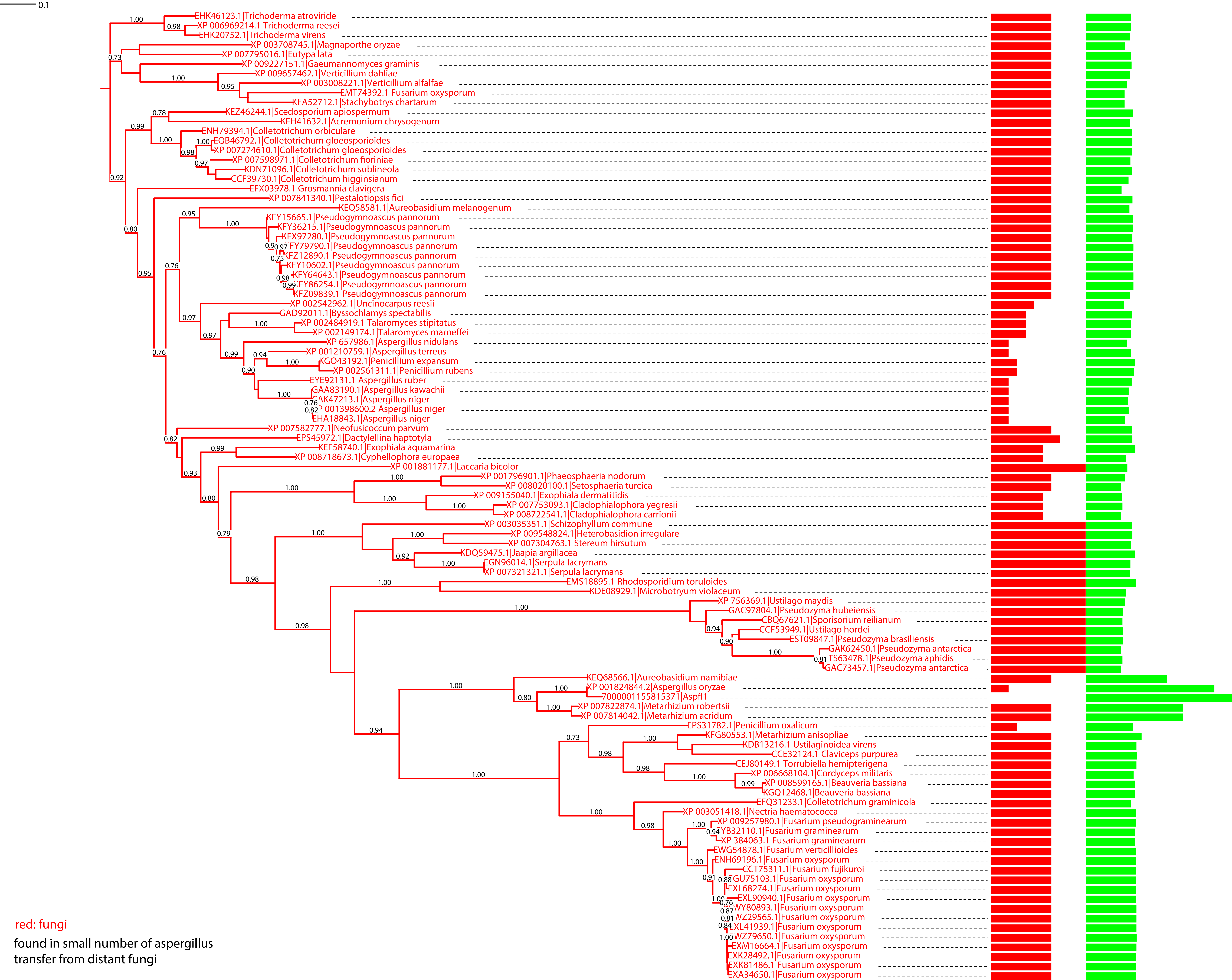
0.1
EHK46123.1|Trichoderma atroviride
XP 006969214.1|Trichoderma reesei
EHK20752.1|Trichoderma virens
XP 003708745.1|Magnaporthe oryzae
XP 007795016.1|Eutypa lata
XP 009227151.1|Gaeumannomyces graminis
XP 009657462.1|Verticillium dahliae
XP 003008221.1|Verticillium alfalfae
EMT74392.1|Fusarium oxysporum
KFA52712.1|Stachybotrys chartarum
KEZ46244.1|Scedosporium apiospermum
KFH41632.1|Acremonium chrysogenum
ENH79394.1|Colletotrichum orbiculare
EQB46792.1|Colletotrichum gloeosporioides
XP 007274610.1|Colletotrichum gloeosporioides
XP 007598971.1|Colletotrichum fioriniae
KDN71096.1|Colletotrichum sublineola
CCF39730.1|Colletotrichum higginsianum
EFX03978.1|Grosmannia clavigera
XP 007841340.1|Pestalotiopsis fici
KEQ58581.1|Aureobasidium melanogenum
KFY15665.1|Pseudogymnoascus pannorum
KFY36215.1|Pseudogymnoascus pannorum
KFX97280.1|Pseudogymnoascus pannorum
FY79790.1|Pseudogymnoascus pannorum
KFZ12890.1|Pseudogymnoascus pannorum
KFY10602.1|Pseudogymnoascus pannorum
KFY64643.1|Pseudogymnoascus pannorum
FY86254.1|Pseudogymnoascus pannorum
KFZ09839.1|Pseudogymnoascus pannorum
XP 002542962.1|Uncinocarpus reesii
GAD92011.1|Byssochlamys spectabilis
XP 002484919.1|Talaromyces stipitatus
XP 002149174.1|Talaromyces marneffei
XP 657986.1|Aspergillus nidulans
XP 001210759.1|Aspergillus terreus
KGO43192.1|Penicillium expansum
XP 002561311.1|Penicillium rubens
EYE92131.1|Aspergillus ruber
GAA83190.1|Aspergillus kawachii
AK47213.1|Aspergillus niger
001398600.2|Aspergillus niger
EHA18843.1|Aspergillus niger
XP 007582777.1|Neofusicoccum parvum
EPS45972.1|Dactylellina haptotyla
KEF58740.1|Exophiala aquamarina
XP 008718673.1|Cyphellophora europaea
XP 001881177.1|Laccaria bicolor
XP 001796901.1|Phaeosphaeria nodorum
XP 008020100.1|Setosphaeria turcica
XP 009155040.1|Exophiala dermatitidis
XP 007753093.1|Cladophialophora yegresii
XP 008722541.1|Cladophialophora carrionii
XP 003035351.1|Schizophyllum commune
XP 009548824.1|Heterobasidion irregulare
XP 007304763.1|Stereum hirsutum
KDQ59475.1|Jaapia argillacea
EGN96014.1|Serpula lacrymans
XP 007321321.1|Serpula lacrymans
EMS18895.1|Rhodosporidium toruloides
KDE08929.1|Microbotryum violaceum
XP 756369.1|Ustilago maydis
GAC97804.1|Pseudozyma hubeiensis
CBQ67621.1|Sporisorium reilianum
CCF53949.1|Ustilago hordei
EST09847.1|Pseudozyma brasiliensis
GAK62450.1|Pseudozyma antarctica
TS63478.1|Pseudozyma aphidis
GAC73457.1|Pseudozyma antarctica
KEQ68566.1|Aureobasidium namibiae
XP 001824844.2|Aspergillus oryzae
7000001155815371|Aspfl1
XP 007822874.1|Metarhizium robertsii
XP 007814042.1|Metarhizium acridum
EPS31782.1|Penicillium oxalicum
KFG80553.1|Metarhizium anisopliae
KDB13216.1|Ustilaginoidea virens
CCE32124.1|Claviceps purpurea
CEJ80149.1|Torrubiella hemipterigena
XP 006668104.1|Cordyceps militaris
XP 008599165.1|Beauveria bassiana
KGQ12468.1|Beauveria bassiana
EFQ31233.1|Colletotrichum graminicola
XP 003051418.1|Nectria haematococca
XP 009257980.1|Fusarium pseudograminearum
YB32110.1|Fusarium graminearum
XP 384063.1|Fusarium graminearum
EWG54878.1|Fusarium verticillioides
ENH69196.1|Fusarium oxysporum
CCT75311.1|Fusarium fujikuroi
GU75103.1|Fusarium oxysporum
EXL68274.1|Fusarium oxysporum
EXL90940.1|Fusarium oxysporum
WY80893.1|Fusarium oxysporum
NZ29565.1|Fusarium oxysporum
XL41939.1|Fusarium oxysporum
WZ79650.1|Fusarium oxysporum
EXM16664.1|Fusarium oxysporum
EXK28492.1|Fusarium oxysporum
EXK81486.1|Fusarium oxysporum
EXA34650.1|Fusarium oxysporum
red: fungi
found in small number of aspergillus
transfer from distant fungi

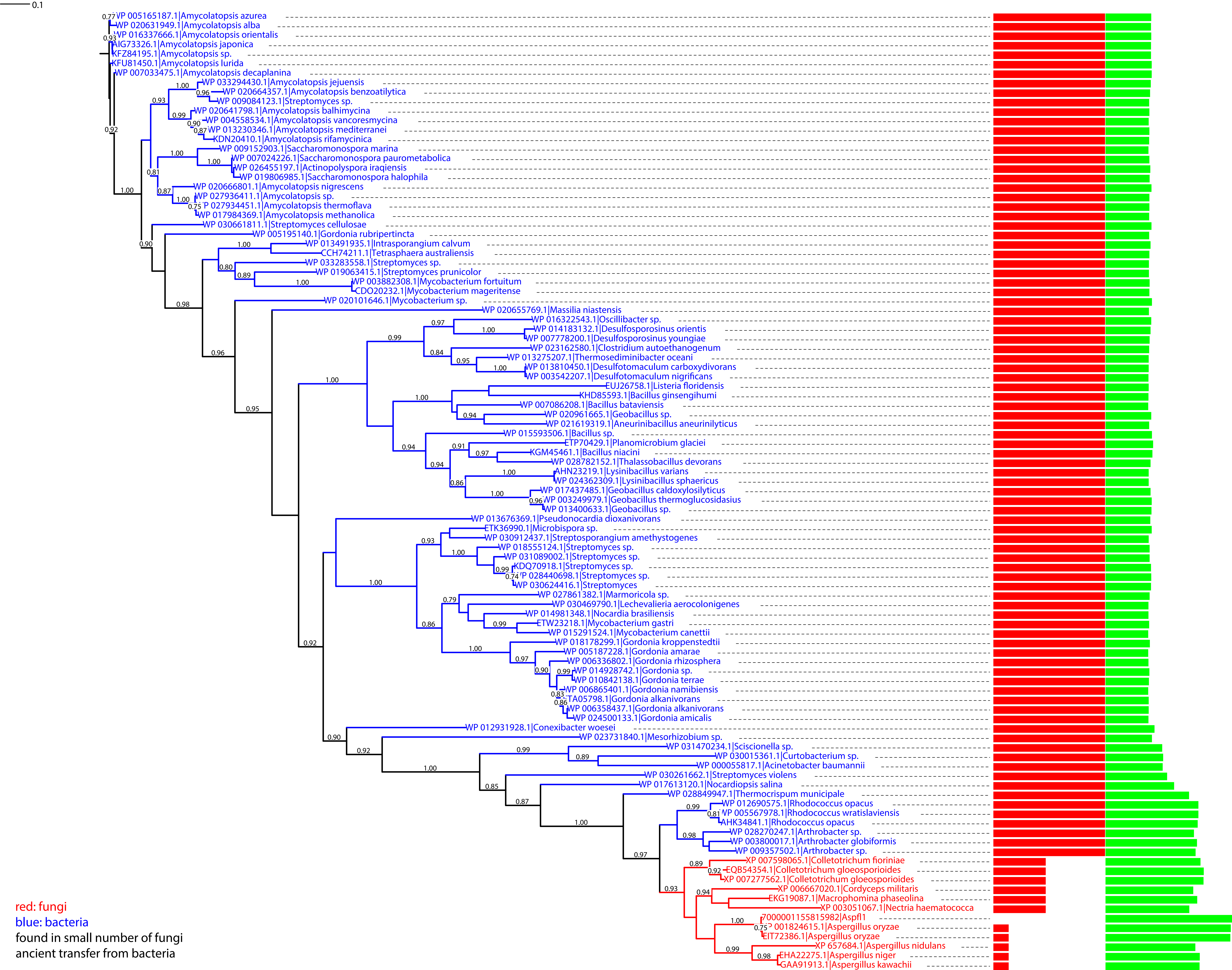
0.1
WP 005165187.1|Amycolatopsis azurea
WP 020631949.1|Amycolatopsis alba
WP 016337666.1|Amycolatopsis orientalis
AIG73326.1|Amycolatopsis japonica
KFZ84195.1|Amycolatopsis sp.
KFU81450.1|Amycolatopsis lurida
WP 007033475.1|Amycolatopsis decaplanina
WP 033294430.1|Amycolatopsis jejuensis
WP 020664357.1|Amycolatopsis benzoatilytica
WP 009084123.1|Streptomyces sp.
WP 020641798.1|Amycolatopsis balhimycina
WP 004558534.1|Amycolatopsis vancoresmycina
WP 013230346.1|Amycolatopsis mediterranei
KDN20410.1|Amycolatopsis rifamycinica
WP 009152903.1|Saccharomonospora marina
WP 007024226.1|Saccharomonospora paurometabolica
WP 026455197.1|Actinopolyspora iraqiensis
WP 019806985.1|Saccharomonospora halophila
WP 020666801.1|Amycolatopsis nigrescens
WP 027936411.1|Amycolatopsis sp.
WP 027934451.1|Amycolatopsis thermoflava
WP 017984369.1|Amycolatopsis methanolica
WP 030661811.1|Streptomyces cellulosae
WP 005195140.1|Gordonia rubripertincta
WP 013491935.1|Intrasporangium calvum
CCH74211.1|Tetrasphaera australiensis
WP 033283558.1|Streptomyces sp.
WP 019063415.1|Streptomyces prunicolor
WP 003882308.1|Mycobacterium fortuitum
CDO20232.1|Mycobacterium mageritense
WP 020101646.1|Mycobacterium sp.
WP 020655769.1|Massilia niastensis
WP 016322543.1|Oscillibacter sp.
WP 014183132.1|Desulfosporosinus orientis
WP 007778200.1|Desulfosporosinus youngiae
WP 023162580.1|Clostridium autoethanogenum
WP 013275207.1|Thermosediminibacter oceani
WP 013810450.1|Desulfotomaculum carboxydivorans
WP 003542207.1|Desulfotomaculum nigrificans
EUJ26758.1|Listeria floridensis
KHD85593.1|Bacillus ginsengihumi
WP 007086208.1|Bacillus bataviensis
WP 020961665.1|Geobacillus sp.
WP 021619319.1|Aneurinibacillus aneurinilyticus
WP 015593506.1|Bacillus sp.
ETP70429.1|Planomicrobium glaciei
KGM45461.1|Bacillus niacini
WP 028782152.1|Thalassobacillus devorans
AHN23219.1|Lysinibacillus varians
WP 024362309.1|Lysinibacillus sphaericus
WP 017437485.1|Geobacillus caldoxylosilyticus
WP 003249979.1|Geobacillus thermoglucosidasius
WP 013400633.1|Geobacillus sp.
WP 013676369.1|Pseudonocardia dioxanivorans
ETK36990.1|Microbispora sp.
WP 030912437.1|Streptosporangium amethystogenes
WP 018555124.1|Streptomyces sp.
WP 031089002.1|Streptomyces sp.
KDQ70918.1|Streptomyces sp.
WP 028440698.1|Streptomyces sp.
WP 030624416.1|Streptomyces
WP 027861382.1|Marmoricola sp.
WP 030469790.1|Lechevalieria aerocolonigenes
WP 014981348.1|Nocardia brasiliensis
ETW23218.1|Mycobacterium gastri
WP 015291524.1|Mycobacterium canettii
WP 018178299.1|Gordonia kroppenstedtii
WP 005187228.1|Gordonia amarae
WP 006336802.1|Gordonia rhizosphera
WP 014928742.1|Gordonia sp.
WP 010842138.1|Gordonia terrae
WP 006865401.1|Gordonia namibiensis
TA05798.1|Gordonia alkanivorans
WP 006358437.1|Gordonia alkanivorans
WP 024500133.1|Gordonia amicalis
WP 012931928.1|Conexibacter woesei
WP 023731840.1|Mesorhizobium sp.
WP 031470234.1|Sciscionella sp.
WP 030015361.1|Curtobacterium sp.
WP 000055817.1|Acinetobacter baumannii
WP 030261662.1|Streptomyces violens
WP 017613120.1|Nocardiopsis salina
WP 028849947.1|Thermocrispum municipale
WP 012690575.1|Rhodococcus opacus
WP 005567978.1|Rhodococcus wratislaviensis
AHK34841.1|Rhodococcus opacus
WP 028270247.1|Arthrobacter sp.
WP 003800017.1|Arthrobacter globiformis
WP 009357502.1|Arthrobacter sp.
XP 007598065.1|Colletotrichum fioriniae
EQB54354.1|Colletotrichum gloeosporioides
XP 007277562.1|Colletotrichum gloeosporioides
XP 006667020.1|Cordyceps militaris
EKG19087.1|Macrophomina phaseolina
XP 003051067.1|Nectria haematococca
7000001155815982|Aspfl1
001824615.1|Aspergillus oryzae
EIT72386.1|Aspergillus oryzae
XP 657684.1|Aspergillus nidulans
EHA22275.1|Aspergillus niger
GAA91913.1|Aspergillus kawachii
red: fungi
blue: bacteria
found in small number of fungi
ancient transfer from bacteria

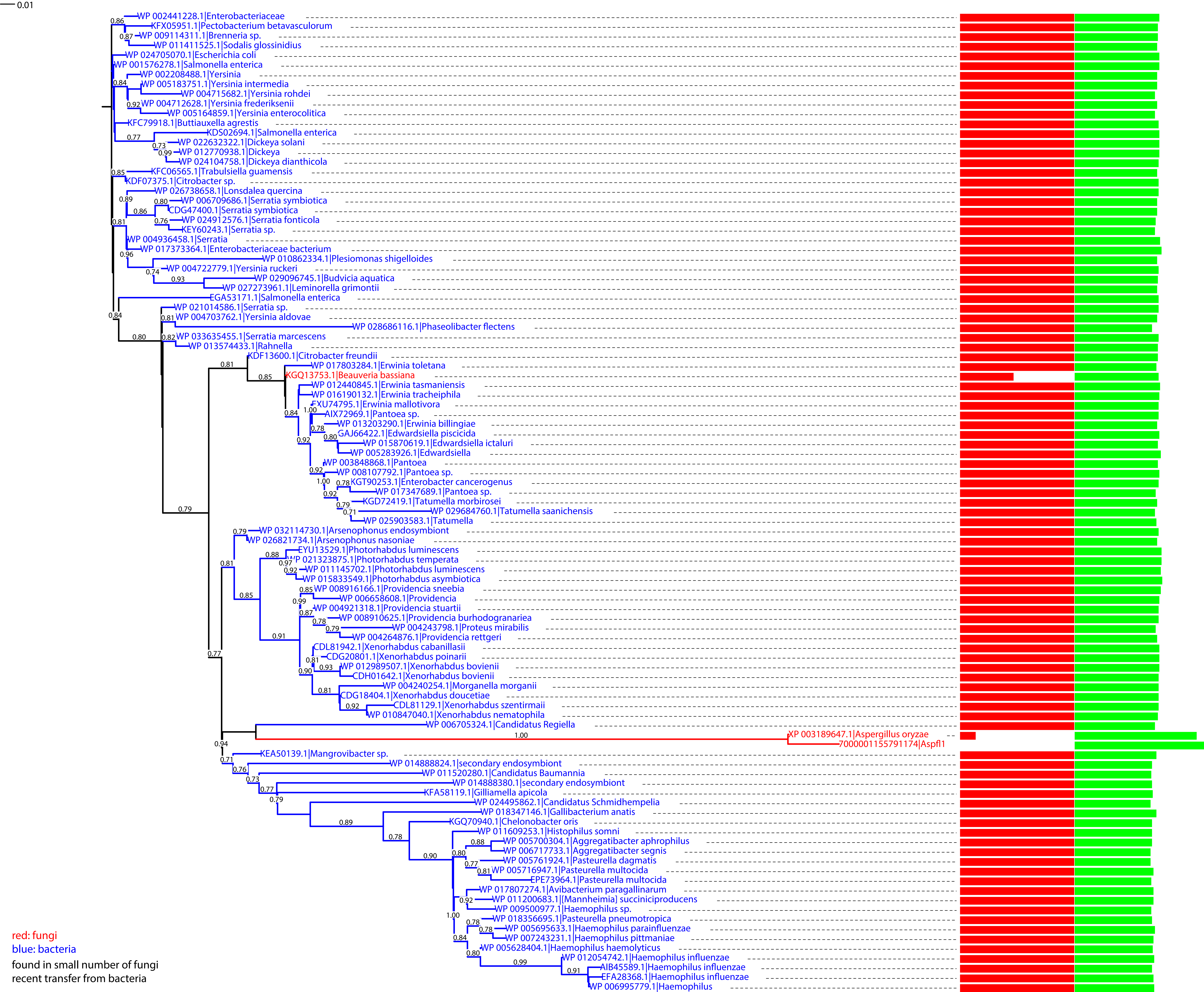
0.01
WP 002441228.1|Enterobacteriaceae
KFX05951.1|Pectobacterium betavasculorum
WP 009114311.1|Brenneria sp.
WP 011411525.1|Sodalis glossinidius
WP 024705070.1|Escherichia coli
WP 001576278.1|Salmonella enterica
WP 002208488.1|Yersinia
WP 005183751.1|Yersinia intermedia
WP 004715682.1|Yersinia rohdei
WP 004712628.1|Yersinia frederiksenii
WP 005164859.1|Yersinia enterocolitica
KFC79918.1|Buttiauxella agrestis
KDS02694.1|Salmonella enterica
WP 022632322.1|Dickeya solani
WP 012770938.1|Dickeya
WP 024104758.1|Dickeya dianthicola
KFC06565.1|Trabulsiella guamensis
KDF07375.1|Citrobacter sp.
WP 026738658.1|Lonsdalea quercina
WP 006709686.1|Serratia symbiotica
CDG47400.1|Serratia symbiotica
WP 024912576.1|Serratia fonticola
KEY60243.1|Serratia sp.
WP 004936458.1|Serratia
WP 017373364.1|Enterobacteriaceae bacterium
WP 010862334.1|Plesiomonas shigelloides
WP 004722779.1|Yersinia ruckeri
WP 029096745.1|Budvicia aquatica
WP 027273961.1|Leminorella grimontii
EGA53171.1|Salmonella enterica
WP 021014586.1|Serratia sp.
WP 004703762.1|Yersinia aldovae
WP 028686116.1|Phaseolibacter flectens
WP 033635455.1|Serratia marcescens
WP 013574433.1|Rahnella
KDF13600.1|Citrobacter freundii
WP 017803284.1|Erwinia toletana
KGQ13753.1|Beauveria bassiana
WP 012440845.1|Erwinia tasmaniensis
WP 016190132.1|Erwinia tracheiphila
EXU74795.1|Erwinia mallotivora
AIX72969.1|Pantoea sp.
WP 013203290.1|Erwinia billingiae
GAJ66422.1|Edwardsiella piscicida
WP 015870619.1|Edwardsiella ictaluri
WP 005283926.1|Edwardsiella
WP 003848868.1|Pantoea
WP 008107792.1|Pantoea sp.
KGT90253.1|Enterobacter cancerogenus
WP 017347689.1|Pantoea sp.
KGD72419.1|Tatumella morbirosei
WP 029684760.1|Tatumella saanichensis
WP 025903583.1|Tatumella
WP 032114730.1|Arsenophonus endosymbiont
WP 026821734.1|Arsenophonus nasoniae
EYU13529.1|Photorhabdus luminescens
WP 021323875.1|Photorhabdus temperata
WP 011145702.1|Photorhabdus luminescens
WP 015833549.1|Photorhabdus asymbiotica
WP 008916166.1|Providencia sneebia
WP 006658608.1|Providencia
WP 004921318.1|Providencia stuartii
WP 008910625.1|Providencia burhodogranariea
WP 004243798.1|Proteus mirabilis
WP 004264876.1|Providencia rettgeri
CDL81942.1|Xenorhabdus cabanillasii
CDG20801.1|Xenorhabdus poinarii
WP 012989507.1|Xenorhabdus bovienii
CDH01642.1|Xenorhabdus bovienii
WP 004240254.1|Morganella morganii
CDG18404.1|Xenorhabdus doucetiae
CDL81129.1|Xenorhabdus szentirmaii
WP 010847040.1|Xenorhabdus nematophila
WP 006705324.1|Candidatus Regiella
XP 003189647.1|Aspergillus oryzae
7000001155791174|Aspfl1
KEA50139.1|Mangrovibacter sp.
WP 014888824.1|secondary endosymbiont
WP 011520280.1|Candidatus Baumannia
WP 014888380.1|secondary endosymbiont
KFA58119.1|Gilliamella apicola
WP 024495862.1|Candidatus Schmidhempelia
WP 018347146.1|Gallibacterium anatis
KGQ70940.1|Chelonobacter oris
WP 011609253.1|Histophilus somni
WP 005700304.1|Aggregatibacter aphrophilus
WP 006717733.1|Aggregatibacter segnis
WP 005761924.1|Pasteurella dagmatis
WP 005716947.1|Pasteurella multocida
EPE73964.1|Pasteurella multocida
WP 017807274.1|Avibacterium paragallinarum
WP 011200683.1|[Mannheimia] succiniciproducens
WP 009500977.1|Haemophilus sp.
WP 018356695.1|Pasteurella pneumotropica
WP 005695633.1|Haemophilus parainfluenzae
WP 007243231.1|Haemophilus pittmaniae
WP 005628404.1|Haemophilus haemolyticus
WP 012054742.1|Haemophilus influenzae
AIB45589.1|Haemophilus influenzae
EFA28368.1|Haemophilus influenzae
WP 006995779.1|Haemophilus
red: fungi
blue: bacteria
found in small number of fungi
recent transfer from bacteria

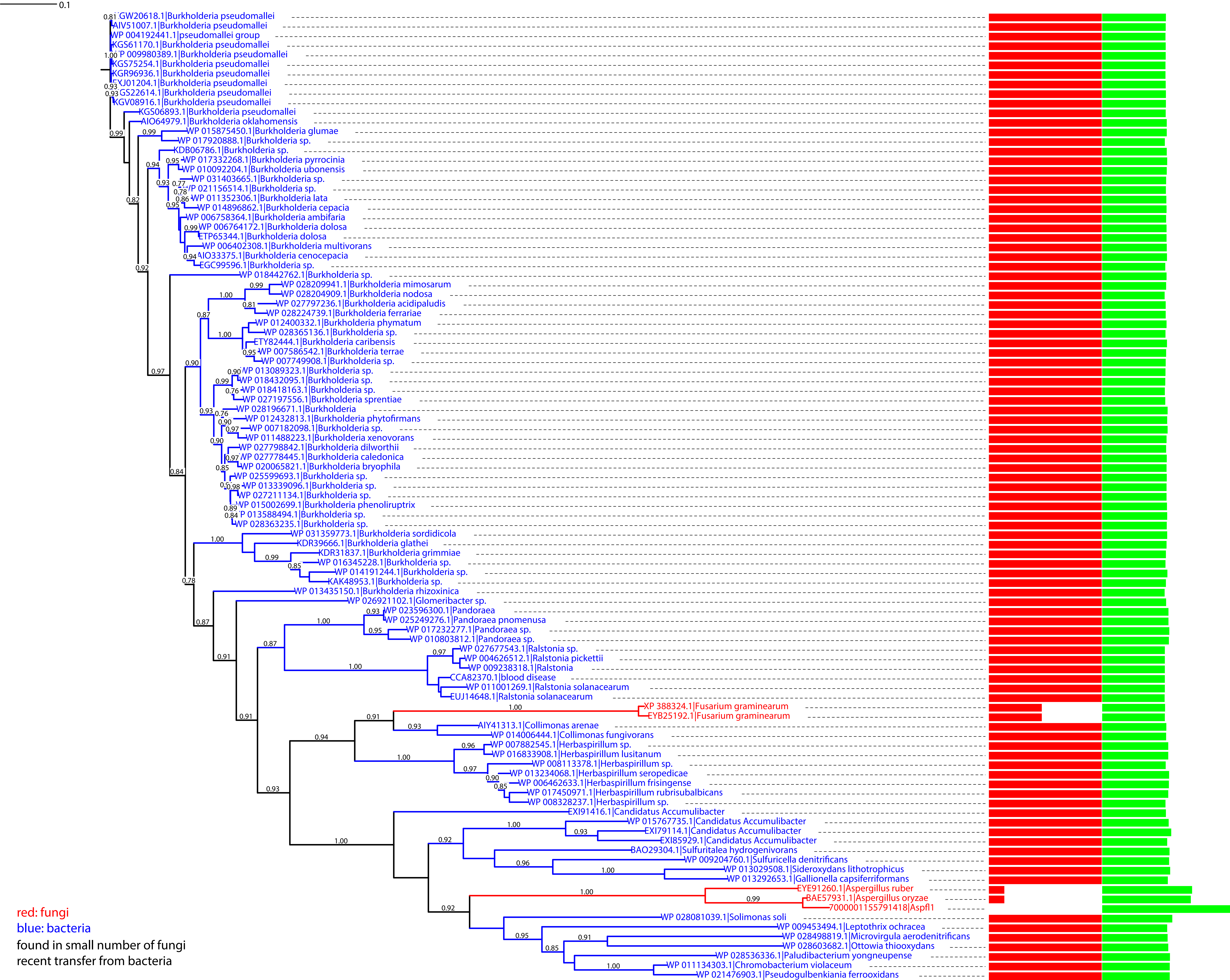
0.1
GW20618.1|Burkholderia pseudomallei
AIV51007.1|Burkholderia pseudomallei
WP 004192441.1|pseudomallei group
KGS61170.1|Burkholderia pseudomallei
P 009980389.1|Burkholderia pseudomallei
KGS75254.1|Burkholderia pseudomallei
KGR96936.1|Burkholderia pseudomallei
XJ01204.1|Burkholderia pseudomallei
GS22614.1|Burkholderia pseudomallei
KGV08916.1|Burkholderia pseudomallei
KGS06893.1|Burkholderia pseudomallei
AIO64979.1|Burkholderia oklahomensis
WP 015875450.1|Burkholderia glumae
WP 017920888.1|Burkholderia sp.
KDB06786.1|Burkholderia sp.
WP 017332268.1|Burkholderia pyrrocinia
WP 010092204.1|Burkholderia ubonensis
WP 031403665.1|Burkholderia sp.
P 021156514.1|Burkholderia sp.
WP 011352306.1|Burkholderia lata
WP 014896862.1|Burkholderia cepacia
WP 006758364.1|Burkholderia ambifaria
WP 006764172.1|Burkholderia dolosa
ETP65344.1|Burkholderia dolosa
WP 006402308.1|Burkholderia multivorans
AIO33375.1|Burkholderia cenocepacia
EGC99596.1|Burkholderia sp.
WP 018442762.1|Burkholderia sp.
WP 028209941.1|Burkholderia mimosarum
WP 028204909.1|Burkholderia nodosa
WP 027797236.1|Burkholderia acidipaludis
WP 028224739.1|Burkholderia ferrariae
WP 012400332.1|Burkholderia phymatum
WP 028365136.1|Burkholderia sp.
ETY82444.1|Burkholderia caribensis
WP 007586542.1|Burkholderia terrae
WP 007749908.1|Burkholderia sp.
WP 013089323.1|Burkholderia sp.
WP 018432095.1|Burkholderia sp.
WP 018418163.1|Burkholderia sp.
WP 027197556.1|Burkholderia sprentiae
WP 028196671.1|Burkholderia
WP 012432813.1|Burkholderia phytofirmans
WP 007182098.1|Burkholderia sp.
WP 011488223.1|Burkholderia xenovorans
WP 027798842.1|Burkholderia dilworthii
WP 027778445.1|Burkholderia caledonica
WP 020065821.1|Burkholderia bryophila
WP 025599693.1|Burkholderia sp.
WP 013339096.1|Burkholderia sp.
WP 027211134.1|Burkholderia sp.
WP 015002699.1|Burkholderia phenoliruptrix
P 013588494.1|Burkholderia sp.
WP 028363235.1|Burkholderia sp.
WP 031359773.1|Burkholderia sordidicola
KDR39666.1|Burkholderia glathei
KDR31837.1|Burkholderia grimmiae
WP 016345228.1|Burkholderia sp.
WP 014191244.1|Burkholderia sp.
KAK48953.1|Burkholderia sp.
WP 013435150.1|Burkholderia rhizoxinica
WP 026921102.1|Glomeribacter sp.
WP 023596300.1|Pandoraea
WP 025249276.1|Pandoraea pnomenusa
WP 017232277.1|Pandoraea sp.
WP 010803812.1|Pandoraea sp.
WP 027677543.1|Ralstonia sp.
WP 004626512.1|Ralstonia pickettii
WP 009238318.1|Ralstonia
CCA82370.1|blood disease
WP 011001269.1|Ralstonia solanacearum
EUJ14648.1|Ralstonia solanacearum
XP 388324.1|Fusarium graminearum
EYB25192.1|Fusarium graminearum
AIY41313.1|Collimonas arenae
WP 014006444.1|Collimonas fungivorans
WP 007882545.1|Herbaspirillum sp.
WP 016833908.1|Herbaspirillum lusitanum
WP 008113378.1|Herbaspirillum sp.
WP 013234068.1|Herbaspirillum seropedicae
WP 006462633.1|Herbaspirillum frisingense
WP 017450971.1|Herbaspirillum rubrisubalbicans
WP 008328237.1|Herbaspirillum sp.
EXI91416.1|Candidatus Accumulibacter
WP 015767735.1|Candidatus Accumulibacter
EXI79114.1|Candidatus Accumulibacter
EXI85929.1|Candidatus Accumulibacter
BAO29304.1|Sulfuritalea hydrogenivorans
WP 009204760.1|Sulfuricella denitrificans
WP 013029508.1|Sideroxydans lithotrophicus
WP 013292653.1|Gallionella capsiferriformans
EYE91260.1|Aspergillus ruber
BAE57931.1|Aspergillus oryzae
7000001155791418|Aspfl1
WP 028081039.1|Solimonas soli
WP 009453494.1|Leptothrix ochracea
WP 028498819.1|Microvirgula aerodenitrificans
WP 028603682.1|Ottowia thiooxydans
WP 028536336.1|Paludibacterium yongneupense
WP 011134303.1|Chromobacterium violaceum
WP 021476903.1|Pseudogulbenkiania ferrooxidans
red: fungi
blue: bacteria
found in small number of fungi
recent transfer from bacteria

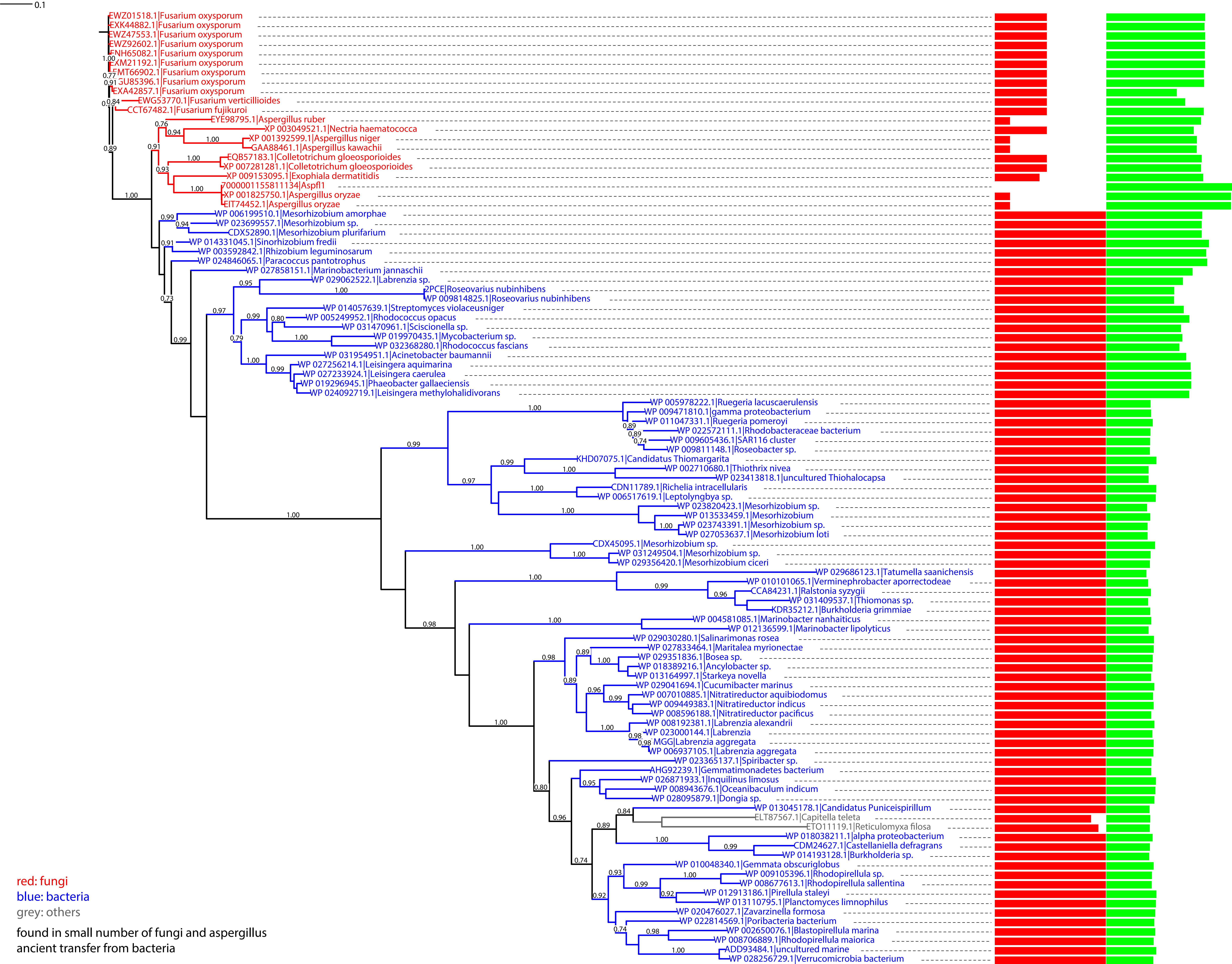
0.1
EWZ01518.1|Fusarium oxysporum
EXK44882.1|Fusarium oxysporum
EWZ47553.1|Fusarium oxysporum
EWZ92602.1|Fusarium oxysporum
ENH65082.1|Fusarium oxysporum
EXM21192.1|Fusarium oxysporum
EMT66902.1|Fusarium oxysporum
EGU85396.1|Fusarium oxysporum
EXA42857.1|Fusarium oxysporum
EWG53770.1|Fusarium verticillioides
CCT67482.1|Fusarium fujikuroi
EYE98795.1|Aspergillus ruber
XP 003049521.1|Nectria haematococca
XP 001392599.1|Aspergillus niger
GAA88461.1|Aspergillus kawachii
EQB57183.1|Colletotrichum gloeosporioides
XP 007281281.1|Colletotrichum gloeosporioides
XP 009153095.1|Exophiala dermatitidis
7000001155811134|Aspfl1
XP 001825750.1|Aspergillus oryzae
EIT74452.1|Aspergillus oryzae
WP 006199510.1|Mesorhizobium amorphae
WP 023699557.1|Mesorhizobium sp.
CDX52890.1|Mesorhizobium plurifarium
WP 014331045.1|Sinorhizobium fredii
WP 003592842.1|Rhizobium leguminosarum
WP 024846065.1|Paracoccus pantotrophus
WP 027858151.1|Marinobacterium jannaschii
WP 029062522.1|Labrenzia sp.
2PCE|Roseovarius nubinhibens
WP 009814825.1|Roseovarius nubinhibens
WP 014057639.1|Streptomyces violaceusniger
WP 005249952.1|Rhodococcus opacus
WP 031470961.1|Sciscionella sp.
WP 019970435.1|Mycobacterium sp.
WP 032368280.1|Rhodococcus fascians
WP 031954951.1|Acinetobacter baumannii
WP 027256214.1|Leisingera aquimarina
WP 027233924.1|Leisingera caerulea
WP 019296945.1|Phaeobacter gallaeciensis
WP 024092719.1|Leisingera methylohalidivorans
WP 005978222.1|Ruegeria lacuscaerulensis
WP 009471810.1|gamma proteobacterium
WP 011047331.1|Ruegeria pomeroyi
WP 022572111.1|Rhodobacteraceae bacterium
WP 009605436.1|SAR116 cluster
WP 009811148.1|Roseobacter sp.
KHD07075.1|Candidatus Thiomargarita
WP 002710680.1|Thiothrix nivea
WP 023413818.1|uncultured Thiohalocapsa
CDN11789.1|Richelia intracellularis
WP 006517619.1|Leptolyngbya sp.
WP 023820423.1|Mesorhizobium sp.
WP 013533459.1|Mesorhizobium
WP 023743391.1|Mesorhizobium sp.
WP 027053637.1|Mesorhizobium loti
CDX45095.1|Mesorhizobium sp.
WP 031249504.1|Mesorhizobium sp.
WP 029356420.1|Mesorhizobium ciceri
WP 029686123.1|Tatumella saanichensis
WP 010101065.1|Verminephrobacter aporrectodeae
CCA84231.1|Ralstonia syzygii
WP 031409537.1|Thiomonas sp.
KDR35212.1|Burkholderia grimmiae
WP 004581085.1|Marinobacter nanhaiticus
WP 012136599.1|Marinobacter lipolyticus
WP 029030280.1|Salinarimonas rosea
WP 027833464.1|Maritalea myrionectae
WP 029351836.1|Bosea sp.
WP 018389216.1|Ancylobacter sp.
WP 013164997.1|Starkeya novella
WP 029041694.1|Cucumibacter marinus
WP 007010885.1|Nitratireductor aquibiodomus
WP 009449383.1|Nitratireductor indicus
WP 008596188.1|Nitratireductor pacificus
WP 008192381.1|Labrenzia alexandrii
WP 023000144.1|Labrenzia
MGG|Labrenzia aggregata
WP 006937105.1|Labrenzia aggregata
WP 023365137.1|Spiribacter sp.
AHG92239.1|Gemmatimonadetes bacterium
WP 026871933.1|Inquilinus limosus
WP 008943676.1|Oceanibaculum indicum
WP 028095879.1|Dongia sp.
WP 013045178.1|Candidatus Puniceispirillum
ELT87567.1|Capitella teleta
ETO11119.1|Reticulomyxa filosa
WP 018038211.1|alpha proteobacterium
CDM24627.1|Castellaniella defragrans
WP 014193128.1|Burkholderia sp.
WP 010048340.1|Gemmata obscuriglobus
WP 009105396.1|Rhodopirellula sp.
WP 008677613.1|Rhodopirellula sallentina
WP 012913186.1|Pirellula staleyi
WP 013110795.1|Planctomyces limnophilus
WP 020476027.1|Zavarzinella formosa
WP 022814569.1|Poribacteria bacterium
WP 002650076.1|Blastopirellula marina
WP 008706889.1|Rhodopirellula maiorica
ADD93484.1|uncultured marine
WP 028256729.1|Verrucomicrobia bacterium
red: fungi
blue: bacteria
grey: others
found in small number of fungi and aspergillus
ancient transfer from bacteria

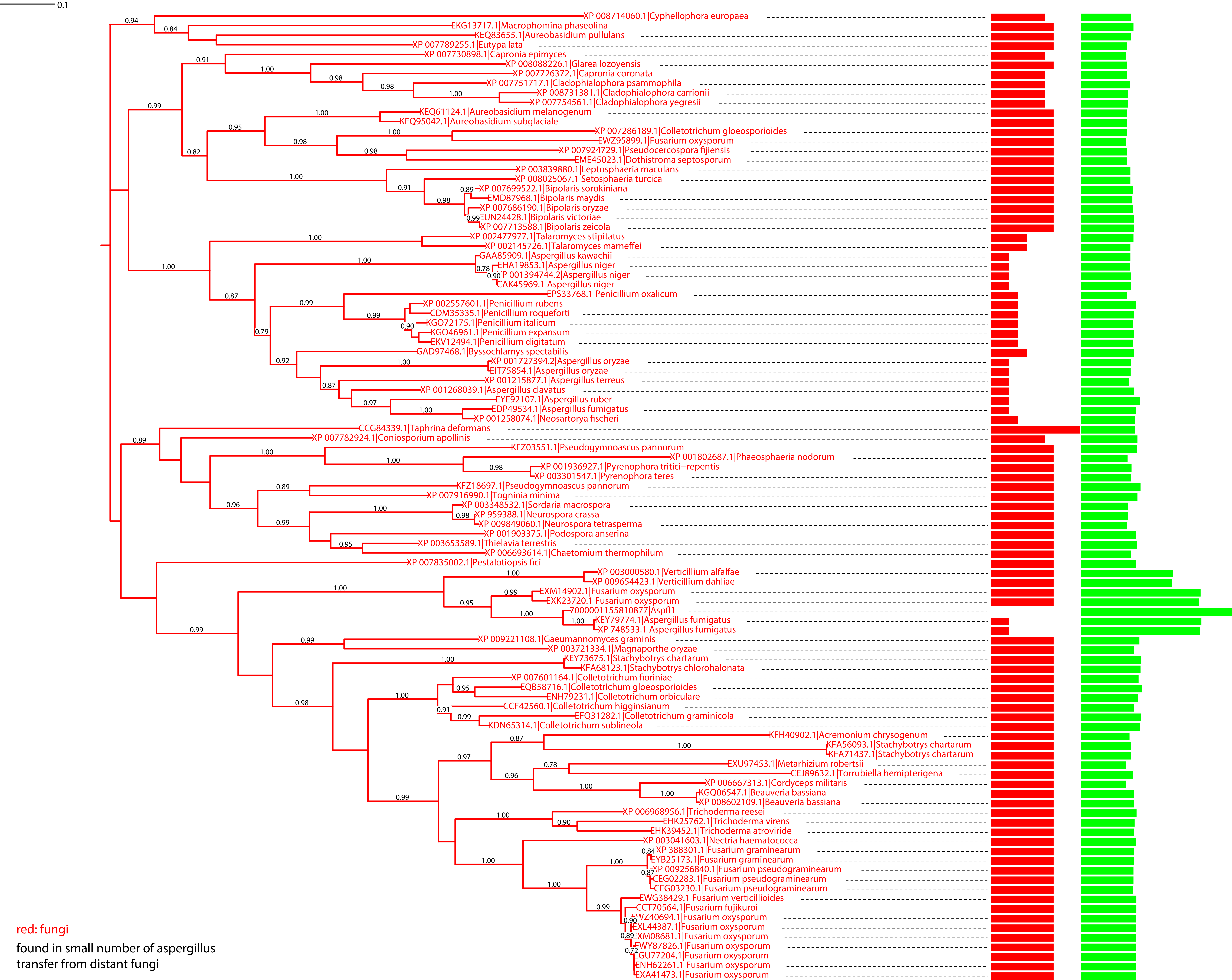
0.1
XP 008714060.1|Cyphellophora europaea
EKG13717.1|Macrophomina phaseolina
KEQ83655.1|Aureobasidium pullulans
XP 007789255.1|Eutypa lata
XP 007730898.1|Capronia epimyces
XP 008088226.1|Glarea lozoyensis
XP 007726372.1|Capronia coronata
XP 007751717.1|Cladophialophora psammophila
XP 008731381.1|Cladophialophora carrionii
XP 007754561.1|Cladophialophora yegresii
KEQ61124.1|Aureobasidium melanogenum
KEQ95042.1|Aureobasidium subglaciale
XP 007286189.1|Colletotrichum gloeosporioides
EWZ95899.1|Fusarium oxysporum
XP 007924729.1|Pseudocercospora fijiensis
EME45023.1|Dothistroma septosporum
XP 003839880.1|Leptosphaeria maculans
XP 008025067.1|Setosphaeria turcica
XP 007699522.1|Bipolaris sorokiniana
EMD87968.1|Bipolaris maydis
XP 007686190.1|Bipolaris oryzae
UN24428.1|Bipolaris victoriae
XP 007713588.1|Bipolaris zeicola
XP 002477977.1|Talaromyces stipitatus
XP 002145726.1|Talaromyces marneffei
GAA85909.1|Aspergillus kawachii
EHA19853.1|Aspergillus niger
P 001394744.2|Aspergillus niger
CAK45969.1|Aspergillus niger
EPS33768.1|Penicillium oxalicum
XP 002557601.1|Penicillium rubens
CDM35335.1|Penicillium roqueforti
KGO72175.1|Penicillium italicum
KGO46961.1|Penicillium expansum
EKV12494.1|Penicillium digitatum
GAD97468.1|Byssochlamys spectabilis
XP 001727394.2|Aspergillus oryzae
EIT75854.1|Aspergillus oryzae
XP 001215877.1|Aspergillus terreus
XP 001268039.1|Aspergillus clavatus
EYE92107.1|Aspergillus ruber
EDP49534.1|Aspergillus fumigatus
XP 001258074.1|Neosartorya fischeri
CCG84339.1|Taphrina deformans
XP 007782924.1|Coniosporium apollinis
KFZ03551.1|Pseudogymnoascus pannorum
XP 001802687.1|Phaeosphaeria nodorum
XP 001936927.1|Pyrenophora tritici–repentis
XP 003301547.1|Pyrenophora teres
KFZ18697.1|Pseudogymnoascus pannorum
XP 007916990.1|Togninia minima
XP 003348532.1|Sordaria macrospora
XP 959388.1|Neurospora crassa
XP 009849060.1|Neurospora tetrasperma
XP 001903375.1|Podospora anserina
XP 003653589.1|Thielavia terrestris
XP 006693614.1|Chaetomium thermophilum
XP 007835002.1|Pestalotiopsis fici
XP 003000580.1|Verticillium alfalfae
XP 009654423.1|Verticillium dahliae
EXM14902.1|Fusarium oxysporum
EXK23720.1|Fusarium oxysporum
7000001155810877|Aspfl1
KEY79774.1|Aspergillus fumigatus
XP 748533.1|Aspergillus fumigatus
XP 009221108.1|Gaeumannomyces graminis
XP 003721334.1|Magnaporthe oryzae
KEY73675.1|Stachybotrys chartarum
KFA68123.1|Stachybotrys chlorohalonata
XP 007601164.1|Colletotrichum fioriniae
EQB58716.1|Colletotrichum gloeosporioides
ENH79231.1|Colletotrichum orbiculare
CCF42560.1|Colletotrichum higginsianum
EFQ31282.1|Colletotrichum graminicola
KDN65314.1|Colletotrichum sublineola
KFH40902.1|Acremonium chrysogenum
KFA56093.1|Stachybotrys chartarum
KFA71437.1|Stachybotrys chartarum
EXU97453.1|Metarhizium robertsii
CEJ89632.1|Torrubiella hemipterigena
XP 006667313.1|Cordyceps militaris
KGQ06547.1|Beauveria bassiana
XP 008602109.1|Beauveria bassiana
XP 006968956.1|Trichoderma reesei
EHK25762.1|Trichoderma virens
EHK39452.1|Trichoderma atroviride
XP 003041603.1|Nectria haematococca
XP 388301.1|Fusarium graminearum
EYB25173.1|Fusarium graminearum
XP 009256840.1|Fusarium pseudograminearum
CEG02283.1|Fusarium pseudograminearum
CEG03230.1|Fusarium pseudograminearum
EWG38429.1|Fusarium verticillioides
CCT70564.1|Fusarium fujikuroi
WZ40694.1|Fusarium oxysporum
EXL44387.1|Fusarium oxysporum
XM08681.1|Fusarium oxysporum
EWY87826.1|Fusarium oxysporum
EGU77204.1|Fusarium oxysporum
ENH62261.1|Fusarium oxysporum
EXA41473.1|Fusarium oxysporum
red: fungi
found in small number of aspergillus
transfer from distant fungi

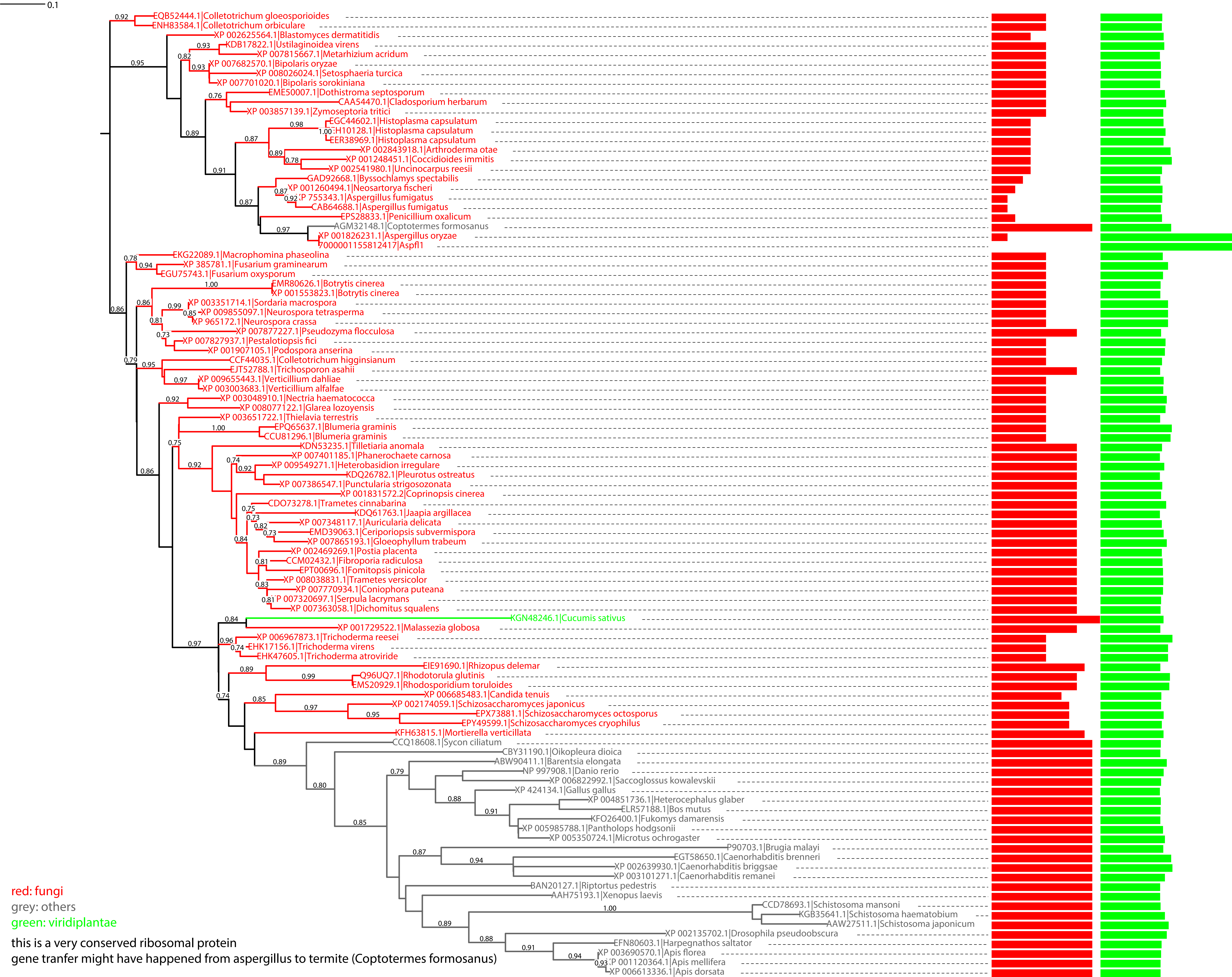

this is a very conserved ribosomal protein
gene tranfer might have happened from aspergillus to termite (Coptotermes formosanus)

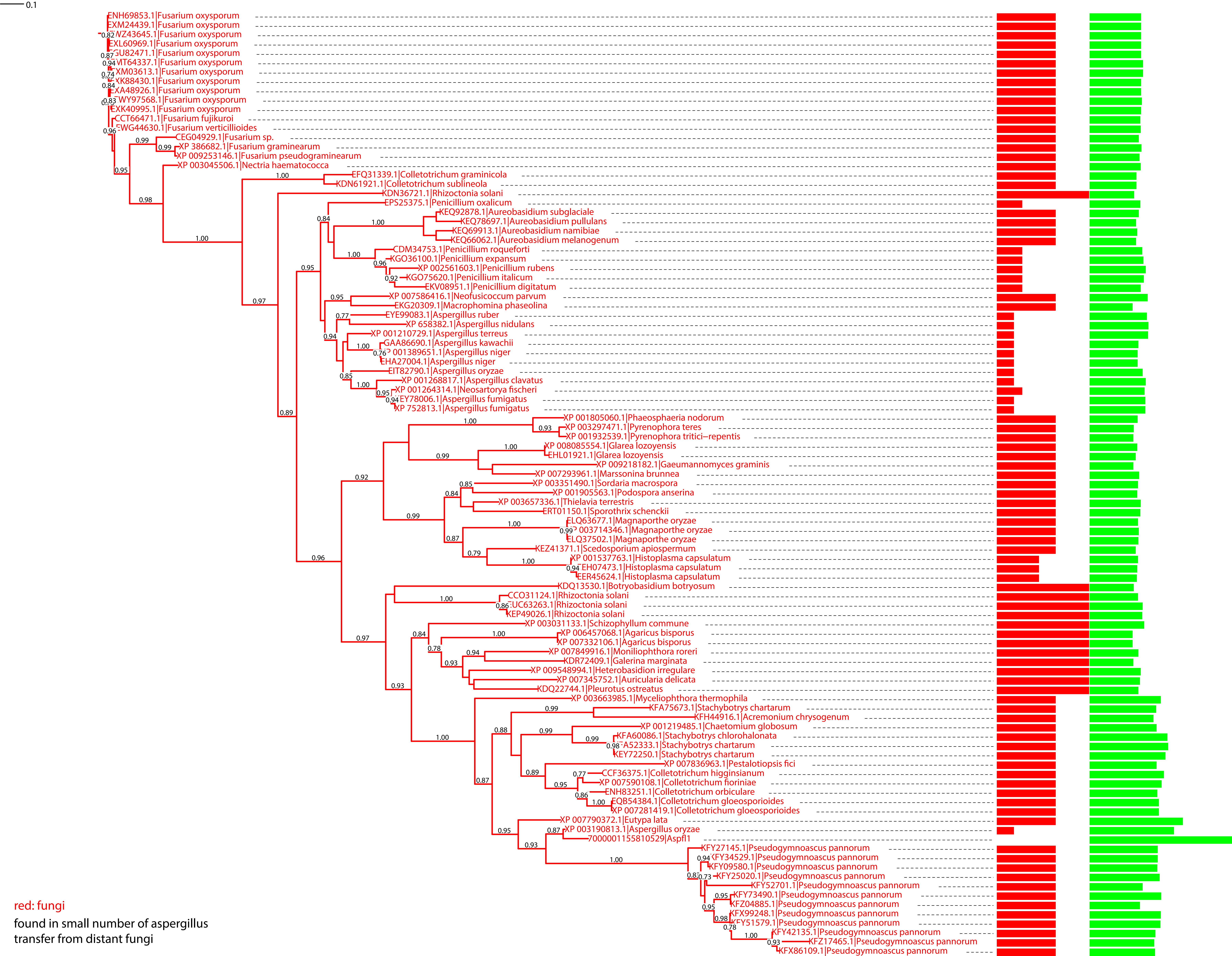

0.1
ENH69853.1|Fusarium oxysporum
EXM24439.1|Fusarium oxysporum
NZ43645.1|Fusarium oxysporum
EXL60969.1|Fusarium oxysporum
GU82471.1|Fusarium oxysporum
VIT64337.1|Fusarium oxysporum
XM03613.1|Fusarium oxysporum
XK88430.1|Fusarium oxysporum
EXA48926.1|Fusarium oxysporum
WY97568.1|Fusarium oxysporum
EXK40995.1|Fusarium oxysporum
CCT66471.1|Fusarium fujikuroi
WG44630.1|Fusarium verticillioides
CEG04929.1|Fusarium sp.
XP 386682.1|Fusarium graminearum
XP 009253146.1|Fusarium pseudograminearum
XP 003045506.1|Nectria haematococca
EFQ31339.1|Colletotrichum graminicola
KDN61921.1|Colletotrichum sublineola
KDN36721.1|Rhizoctonia solani
EPS25375.1|Penicillium oxalicum
KEQ92878.1|Aureobasidium subglaciale
KEQ78697.1|Aureobasidium pullulans
KEQ69913.1|Aureobasidium namibiae
KEQ66062.1|Aureobasidium melanogenum
CDM34753.1|Penicillium roqueforti
KGO36100.1|Penicillium expansum
XP 002561603.1|Penicillium rubens
KGO75620.1|Penicillium italicum
EKV08951.1|Penicillium digitatum
XP 007586416.1|Neofusicoccum parvum
EKG20309.1|Macrophomina phaseolina
EYE99083.1|Aspergillus ruber
XP 658382.1|Aspergillus nidulans
XP 001210729.1|Aspergillus terreus
GAA86690.1|Aspergillus kawachii
001389651.1|Aspergillus niger
EHA27004.1|Aspergillus niger
EIT82790.1|Aspergillus oryzae
XP 001268817.1|Aspergillus clavatus
XP 001264314.1|Neosartorya fischeri
EY78006.1|Aspergillus fumigatus
XP 752813.1|Aspergillus fumigatus
XP 001805060.1|Phaeosphaeria nodorum
XP 003297471.1|Pyrenophora teres
XP 001932539.1|Pyrenophora tritici−repentis
XP 008085554.1|Glarea lozoyensis
EHL01921.1|Glarea lozoyensis
XP 009218182.1|Gaeumannomyces graminis
XP 007293961.1|Marssonina brunnea
XP 003351490.1|Sordaria macrospora
XP 001905563.1|Podospora anserina
XP 003657336.1|Thielavia terrestris
ERT01150.1|Sporothrix schenckii
ELQ63677.1|Magnaporthe oryzae
003714346.1|Magnaporthe oryzae
ELQ37502.1|Magnaporthe oryzae
KEZ41371.1|Scedosporium apiospermum
XP 001537763.1|Histoplasma capsulatum
EH07473.1|Histoplasma capsulatum
EER45624.1|Histoplasma capsulatum
KDQ13530.1|Botryobasidium botryosum
CCO31124.1|Rhizoctonia solani
UC63263.1|Rhizoctonia solani
KEP49026.1|Rhizoctonia solani
XP 003031133.1|Schizophyllum commune
XP 006457068.1|Agaricus bisporus
XP 007332106.1|Agaricus bisporus
XP 007849916.1|Moniliophthora roreri
KDR72409.1|Galerina marginata
XP 009548994.1|Heterobasidion irregulare
XP 007345752.1|Auricularia delicata
KDQ22744.1|Pleurotus ostreatus
XP 003663985.1|Myceliophthora thermophila
KFA75673.1|Stachybotrys chartarum
KFH44916.1|Acremonium chrysogenum
XP 001219485.1|Chaetomium globosum
KFA60086.1|Stachybotrys chlorohalonata
A52333.1|Stachybotrys chartarum
KEY72250.1|Stachybotrys chartarum
XP 007836963.1|Pestalotiopsis fici
CCF36375.1|Colletotrichum higginsianum
XP 007590108.1|Colletotrichum fioriniae
ENH83251.1|Colletotrichum orbiculare
EQB54384.1|Colletotrichum gloeosporioides
XP 007281419.1|Colletotrichum gloeosporioides
XP 007790372.1|Eutypa lata
XP 003190813.1|Aspergillus oryzae
7000001155810529|Aspfl1
KFY27145.1|Pseudogymnoascus pannorum
KFY34529.1|Pseudogymnoascus pannorum
KFY09580.1|Pseudogymnoascus pannorum
KFY25020.1|Pseudogymnoascus pannorum
KFY52701.1|Pseudogymnoascus pannorum
KFY73490.1|Pseudogymnoascus pannorum
KFZ04885.1|Pseudogymnoascus pannorum
KFX99248.1|Pseudogymnoascus pannorum
KFY51579.1|Pseudogymnoascus pannorum
KFY42135.1|Pseudogymnoascus pannorum
KFZ17465.1|Pseudogymnoascus pannorum
KFX86109.1|Pseudogymnoascus pannorum
red: fungi
found in small number of aspergillus
transfer from distant fungi

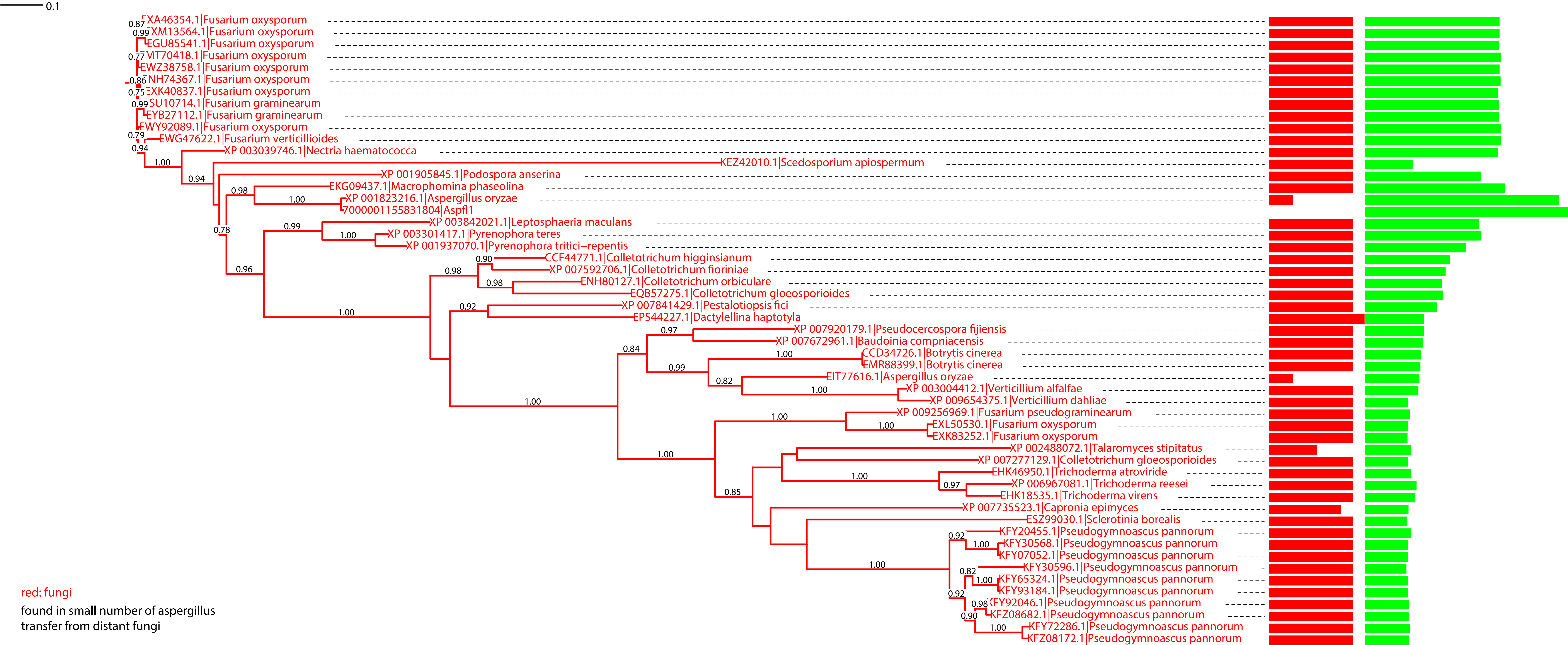
0.1
EXA46354.1|Fusarium oxysporum
XM13564.1|Fusarium oxysporum
EGU85541.1|Fusarium oxysporum
VIT70418.1|Fusarium oxysporum
EWZ38758.1|Fusarium oxysporum
NH74367.1|Fusarium oxysporum
EXK40837.1|Fusarium oxysporum
SU10714.1|Fusarium graminearum
EYB27112.1|Fusarium graminearum
EWY92089.1|Fusarium oxysporum
EWG47622.1|Fusarium verticillioides
XP 003039746.1|Nectria haematococca
KEZ42010.1|Scedosporium apiospermum
XP 001905845.1|Podospora anserina
EKG09437.1|Macrophomina phaseolina
XP 001823216.1|Aspergillus oryzae
7000001155831804|Aspfl1
XP 003842021.1|Leptosphaeria maculans
XP 003301417.1|Pyrenophora teres
XP 001937070.1|Pyrenophora tritici–repentis
CCF44771.1|Colletotrichum higginsianum
XP 007592706.1|Colletotrichum fioriniae
ENH80127.1|Colletotrichum orbiculare
EQB57275.1|Colletotrichum gloeosporioides
XP 007841429.1|Pestalotiopsis fici
EPS44227.1|Dactylellina haptotyla
XP 007920179.1|Pseudocercospora fijiensis
XP 007672961.1|Baudoinia compniacensis
CCD34726.1|Botrytis cinerea
EMR88399.1|Botrytis cinerea
EIT77616.1|Aspergillus oryzae
XP 003004412.1|Verticillium alfalfae
XP 009654375.1|Verticillium dahliae
XP 009256969.1|Fusarium pseudograminearum
EXL50530.1|Fusarium oxysporum
EXK83252.1|Fusarium oxysporum
XP 002488072.1|Talaromyces stipitatus
XP 007277129.1|Colletotrichum gloeosporioides
EHK46950.1|Trichoderma atroviride
XP 006967081.1|Trichoderma reesei
EHK18535.1|Trichoderma virens
XP 007735523.1|Capronia epimyces
ESZ99030.1|Sclerotinia borealis
KFY20455.1|Pseudogymnoascus pannorum
KFY30568.1|Pseudogymnoascus pannorum
KFY07052.1|Pseudogymnoascus pannorum
KFY30596.1|Pseudogymnoascus pannorum
KFY65324.1|Pseudogymnoascus pannorum
KFY93184.1|Pseudogymnoascus pannorum
KFY92046.1|Pseudogymnoascus pannorum
KFZ08682.1|Pseudogymnoascus pannorum
KFY72286.1|Pseudogymnoascus pannorum
KFZ08172.1|Pseudogymnoascus pannorum
red: fungi
found in small number of aspergillus
transfer from distant fungi

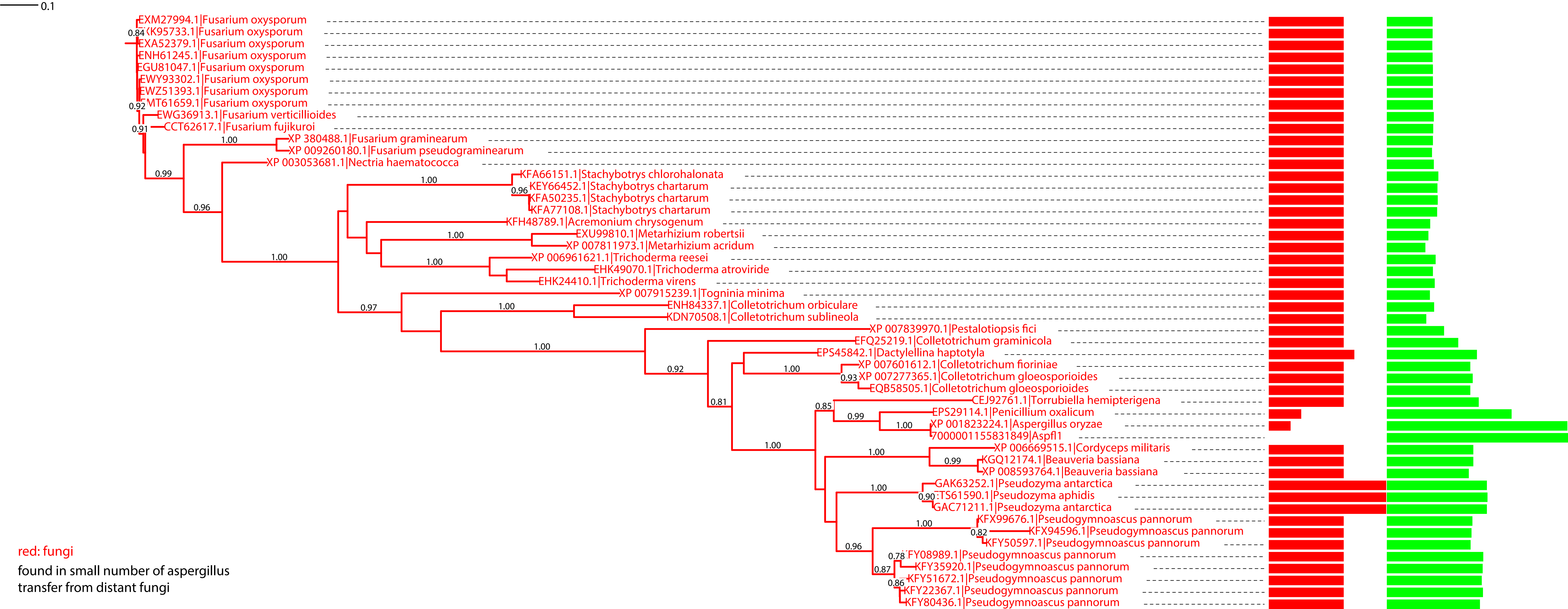
0.1
EXM27994.1|Fusarium oxysporum
0.84
K95733.1|Fusarium oxysporum
EXA52379.1|Fusarium oxysporum
ENH61245.1|Fusarium oxysporum
EGU81047.1|Fusarium oxysporum
EWY93302.1|Fusarium oxysporum
EWZ51393.1|Fusarium oxysporum
MT61659.1|Fusarium oxysporum
0.92
EWG36913.1|Fusarium verticillioides
0.91
CCT62617.1|Fusarium fujikuroi
1.00
XP 380488.1|Fusarium graminearum
XP 009260180.1|Fusarium pseudograminearum
0.99
XP 003053681.1|Nectria haematococca
0.96
1.00
KFA66151.1|Stachybotrys chlorohalonata
0.96
KEY66452.1|Stachybotrys chartarum
KFA50235.1|Stachybotrys chartarum
KFA77108.1|Stachybotrys chartarum
KFH48789.1|Acremonium chrysogenum
1.00
1.00
EXU99810.1|Metarhizium robertsii
XP 007811973.1|Metarhizium acridum
1.00
XP 006961621.1|Trichoderma reesei
EHK49070.1|Trichoderma atroviride
EHK24410.1|Trichoderma virens
0.97
XP 007915239.1|Togninia minima
1.00
ENH84337.1|Colletotrichum orbiculare
KDN70508.1|Colletotrichum sublineola
1.00
XP 007839970.1|Pestalotiopsis fici
EFQ25219.1|Colletotrichum graminicola
0.92
EPS45842.1|Dactylellina haptotyla
1.00
XP 007601612.1|Colletotrichum fioriniae
0.93
XP 007277365.1|Colletotrichum gloeosporioides
EQB58505.1|Colletotrichum gloeosporioides
0.81
0.85
CEJ92761.1|Torrubiella hemipterigena
0.99
EPS29114.1|Penicillium oxalicum
1.00
XP 001823224.1|Aspergillus oryzae
7000001155831849|Aspfl1
1.00
1.00
XP 006669515.1|Cordyceps militaris
0.99
KGQ12174.1|Beauveria bassiana
XP 008593764.1|Beauveria bassiana
1.00
GAK63252.1|Pseudozyma antarctica
0.90
TS61590.1|Pseudozyma aphidis
GAC71211.1|Pseudozyma antarctica
1.00
KFX99676.1|Pseudogymnoascus pannorum
0.82
KFX94596.1|Pseudogymnoascus pannorum
KFY50597.1|Pseudogymnoascus pannorum
0.96
0.78
FY08989.1|Pseudogymnoascus pannorum
0.87
KFY35920.1|Pseudogymnoascus pannorum
KFY51672.1|Pseudogymnoascus pannorum
0.86
KFY22367.1|Pseudogymnoascus pannorum
KFY80436.1|Pseudogymnoascus pannorum
red: fungi
found in small number of aspergillus
transfer from distant fungi

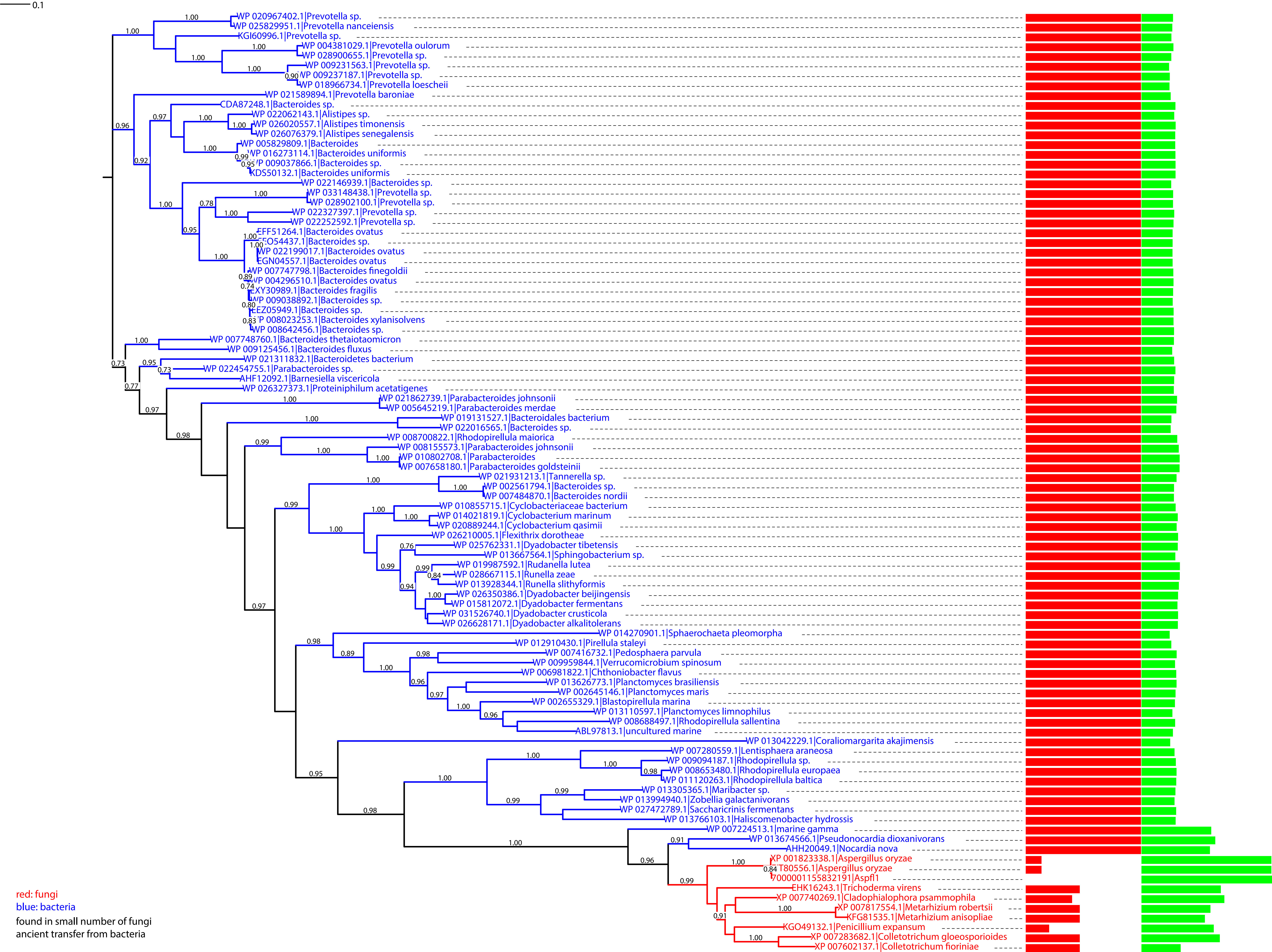
0.1
WP 020967402.1|Prevotella sp.
WP 025829951.1|Prevotella nanceiensis
KGI60996.1|Prevotella sp.
WP 004381029.1|Prevotella oulorum
WP 028900655.1|Prevotella sp.
WP 009231563.1|Prevotella sp.
WP 009237187.1|Prevotella sp.
WP 018966734.1|Prevotella loescheii
WP 021589894.1|Prevotella baroniae
CDA87248.1|Bacteroides sp.
WP 022062143.1|Alistipes sp.
WP 026020557.1|Alistipes timonensis
WP 026076379.1|Alistipes senegalensis
WP 005829809.1|Bacteroides
WP 016273114.1|Bacteroides uniformis
P 009037866.1|Bacteroides sp.
KDS50132.1|Bacteroides uniformis
WP 022146939.1|Bacteroides sp.
WP 033148438.1|Prevotella sp.
WP 028902100.1|Prevotella sp.
WP 022327397.1|Prevotella sp.
WP 022252592.1|Prevotella sp.
EFF51264.1|Bacteroides ovatus
O54437.1|Bacteroides sp.
WP 022199017.1|Bacteroides ovatus
EGN04557.1|Bacteroides ovatus
WP 007747798.1|Bacteroides finegoldii
P 004296510.1|Bacteroides ovatus
EXY30989.1|Bacteroides fragilis
WP 009038892.1|Bacteroides sp.
EEZ05949.1|Bacteroides sp.
P 008023253.1|Bacteroides xylanisolvens
WP 008642456.1|Bacteroides sp.
WP 007748760.1|Bacteroides thetaiotaomicron
WP 009125456.1|Bacteroides fluxus
WP 021311832.1|Bacteroidetes bacterium
WP 022454755.1|Parabacteroides sp.
AHF12092.1|Barnesiella viscericola
WP 026327373.1|Proteiniphilum acetatigenes
WP 021862739.1|Parabacteroides johnsonii
WP 005645219.1|Parabacteroides merdae
WP 019131527.1|Bacteroidales bacterium
WP 022016565.1|Bacteroides sp.
WP 008700822.1|Rhodopirellula maiorica
WP 008155573.1|Parabacteroides johnsonii
WP 010802708.1|Parabacteroides
WP 007658180.1|Parabacteroides goldsteinii
WP 021931213.1|Tannerella sp.
WP 002561794.1|Bacteroides sp.
WP 007484870.1|Bacteroides nordii
WP 010855715.1|Cyclobacteriaceae bacterium
WP 014021819.1|Cyclobacterium marinum
WP 020889244.1|Cyclobacterium qasimii
WP 026210005.1|Flexithrix dorotheae
WP 025762331.1|Dyadobacter tibetensis
WP 013667564.1|Sphingobacterium sp.
WP 019987592.1|Rudanella lutea
WP 028667115.1|Runella zeae
WP 013928344.1|Runella slithyformis
WP 026350386.1|Dyadobacter beijingensis
WP 015812072.1|Dyadobacter fermentans
WP 031526740.1|Dyadobacter crusticola
WP 026628171.1|Dyadobacter alkalitolerans
WP 014270901.1|Sphaerochaeta pleomorpha
WP 012910430.1|Pirellula staleyi
WP 007416732.1|Pedosphaera parvula
WP 009959844.1|Verrucomicrobium spinosum
WP 006981822.1|Chthoniobacter flavus
WP 013626773.1|Planctomyces brasiliensis
WP 002645146.1|Planctomyces maris
WP 002655329.1|Blastopirellula marina
WP 013110597.1|Planctomyces limnophilus
WP 008688497.1|Rhodopirellula sallentina
ABL97813.1|uncultured marine
WP 013042229.1|Coraliomargarita akajimensis
WP 007280559.1|Lentisphaera araneosa
WP 009094187.1|Rhodopirellula sp.
WP 008653480.1|Rhodopirellula europaea
WP 011120263.1|Rhodopirellula baltica
WP 013305365.1|Maribacter sp.
WP 013994940.1|Zobellia galactanivorans
WP 027472789.1|Saccharicrinis fermentans
WP 013766103.1|Haliscomenobacter hydrossis
WP 007224513.1|marine gamma
WP 013674566.1|Pseudonocardia dioxanivorans
AHH20049.1|Nocardia nova
XP 001823338.1|Aspergillus oryzae
T80556.1|Aspergillus oryzae
7000001155832191|Aspfl1
EHK16243.1|Trichoderma virens
XP 007740269.1|Cladophialophora psammophila
XP 007817554.1|Metarhizium robertsii
KFG81535.1|Metarhizium anisopliae
KGO49132.1|Penicillium expansum
XP 007283682.1|Colletotrichum gloeosporioides
XP 007602137.1|Colletotrichum fioriniae
red: fungi
blue: bacteria
found in small number of fungi
ancient transfer from bacteria

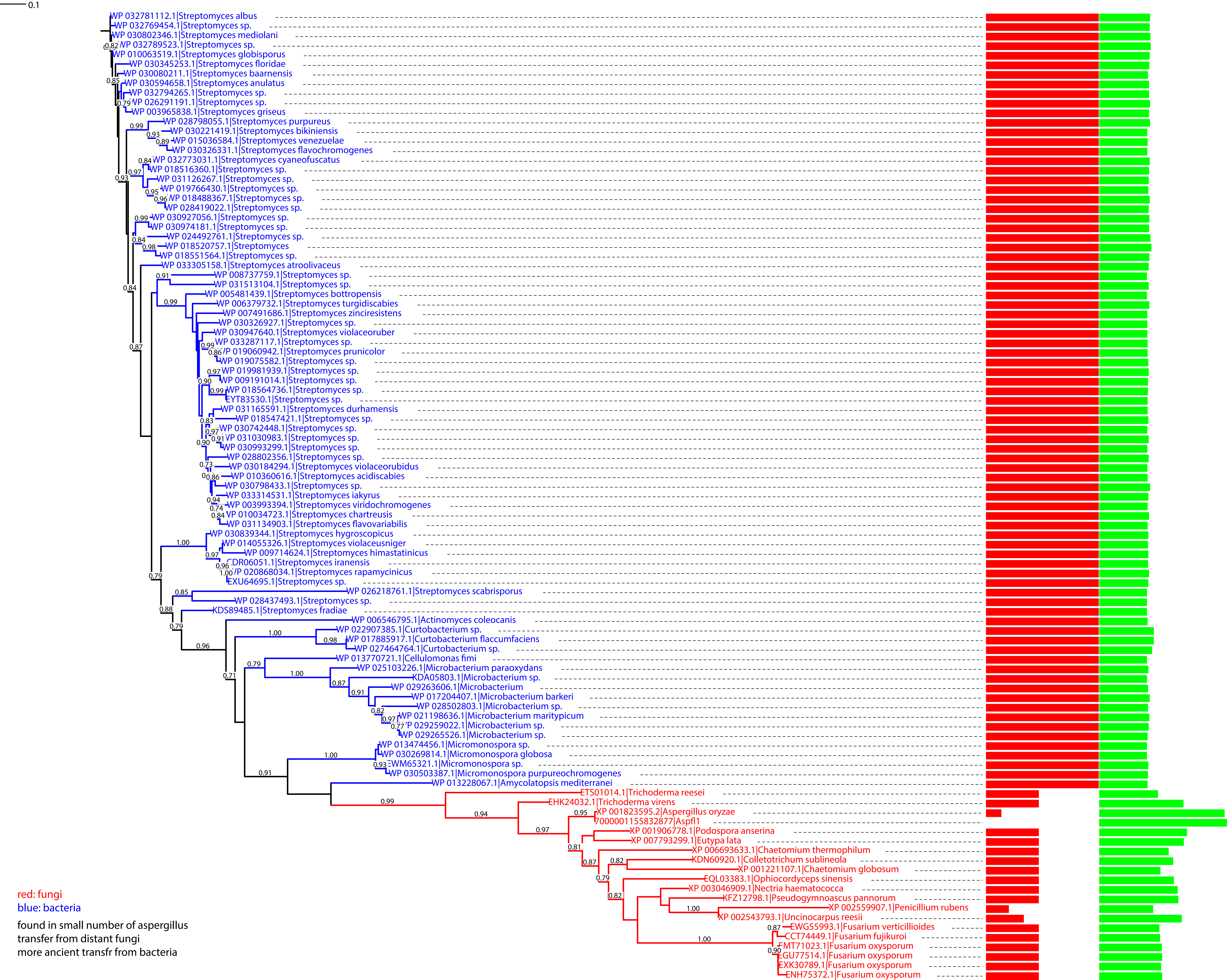

0.1
WP 032781112.1|Streptomyces albus
WP 032769454.1|Streptomyces sp.
WP 030802346.1|Streptomyces mediolani
WP 032789523.1|Streptomyces sp.
WP 010063519.1|Streptomyces globisporus
WP 030345253.1|Streptomyces floridae
WP 030080211.1|Streptomyces baarnensis
WP 030594658.1|Streptomyces anulatus
WP 032794265.1|Streptomyces sp.
WP 026291191.1|Streptomyces sp.
WP 003965838.1|Streptomyces griseus
WP 028798055.1|Streptomyces purpureus
WP 030221419.1|Streptomyces bikiniensis
WP 015036584.1|Streptomyces venezuelae
WP 030326331.1|Streptomyces flavochromogenes
WP 032773031.1|Streptomyces cyaneofuscatus
WP 018516360.1|Streptomyces sp.
WP 031126267.1|Streptomyces sp.
WP 019766430.1|Streptomyces sp.
WP 018488367.1|Streptomyces sp.
WP 028419022.1|Streptomyces sp.
WP 030927056.1|Streptomyces sp.
WP 030974181.1|Streptomyces sp.
WP 024492761.1|Streptomyces sp.
WP 018520757.1|Streptomyces
WP 018551564.1|Streptomyces sp.
WP 033305158.1|Streptomyces atroolivaceus
WP 008737759.1|Streptomyces sp.
WP 031513104.1|Streptomyces sp.
WP 005481439.1|Streptomyces bottropensis
WP 006379732.1|Streptomyces turgidiscabies
WP 007491686.1|Streptomyces zincire sistens
WP 030326927.1|Streptomyces sp.
WP 030947640.1|Streptomyces violaceoruber
WP 033287117.1|Streptomyces sp.
WP 019060942.1|Streptomyces prunicolor
WP 019075582.1|Streptomyces sp.
WP 019981939.1|Streptomyces sp.
WP 009191014.1|Streptomyces sp.
WP 018564736.1|Streptomyces sp.
EYT83530.1|Streptomyces sp.
WP 031165591.1|Streptomyces durhamensis
WP 018547421.1|Streptomyces sp.
WP 030742448.1|Streptomyces sp.
WP 031030983.1|Streptomyces sp.
WP 030993299.1|Streptomyces sp.
WP 028802356.1|Streptomyces sp.
WP 030184294.1|Streptomyces violaceorubidus
WP 010360616.1|Streptomyces acidiscabies
WP 030798433.1|Streptomyces sp.
WP 033314531.1|Streptomyces iakyrus
WP 003993394.1|Streptomyces viridochromogenes
WP 010034723.1|Streptomyces chartreusis
WP 031134903.1|Streptomyces flavovariabilis
WP 030839344.1|Streptomyces hygroscopicus
WP 014055326.1|Streptomyces violaceusniger
WP 009714624.1|Streptomyces himastatinicus
CDR06051.1|Streptomyces iranensis
WP 020868034.1|Streptomyces rapamycinicus
EXU64695.1|Streptomyces sp.
WP 026218761.1|Streptomyces scabrisporus
WP 028437493.1|Streptomyces sp.
KDS89485.1|Streptomyces fradiae
WP 006546795.1|Actinomyces coleocanis
WP 022907385.1|Curtobacterium sp.
WP 017885917.1|Curtobacterium flaccumfaciens
WP 027464764.1|Curtobacterium sp.
WP 013770721.1|Cellulomonas fimi
WP 025103226.1|Microbacterium paraoxydans
KDA05803.1|Microbacterium sp.
WP 029263606.1|Microbacterium
WP 017204407.1|Microbacterium barkeri
WP 028502803.1|Microbacterium sp.
WP 021198636.1|Microbacterium maritypicum
WP 029259022.1|Microbacterium sp.
WP 029265526.1|Microbacterium sp.
WP 013474456.1|Micromonospora sp.
WP 030269814.1|Micromonospora globosa
EWM65321.1|Micromonospora sp.
WP 030503387.1|Micromonospora purpureochromogenes
WP 013228067.1|Amycolatopsis mediterranei
ETS01014.1|Trichoderma reesei
EHK24032.1|Trichoderma virens
XP 001823595.2|Aspergillus oryzae
7000001155832877|Aspfl1
XP 001906778.1|Podospora anserina
XP 007793299.1|Eutypa lata
XP 006693633.1|Chaetomium thermophilum
KDN60920.1|Colletotrichum sublineola
XP 001221107.1|Chaetomium globosum
EQL03383.1|Ophiocordyceps sinensis
XP 003046909.1|Nectria haematococca
KFZ12798.1|Pseudogymnoascus pannorum
XP 002559907.1|Penicillium rubens
XP 002543793.1|Uncinocarpus reesii
EWG55993.1|Fusarium verticillioides
CCT74449.1|Fusarium fujikuroi
EMT71023.1|Fusarium oxysporum
EGU77514.1|Fusarium oxysporum
EXK30789.1|Fusarium oxysporum
ENH75372.1|Fusarium oxysporum
red: fungi
blue: bacteria
found in small number of aspergillus
transfer from distant fungi
more ancient transfr from bacteria

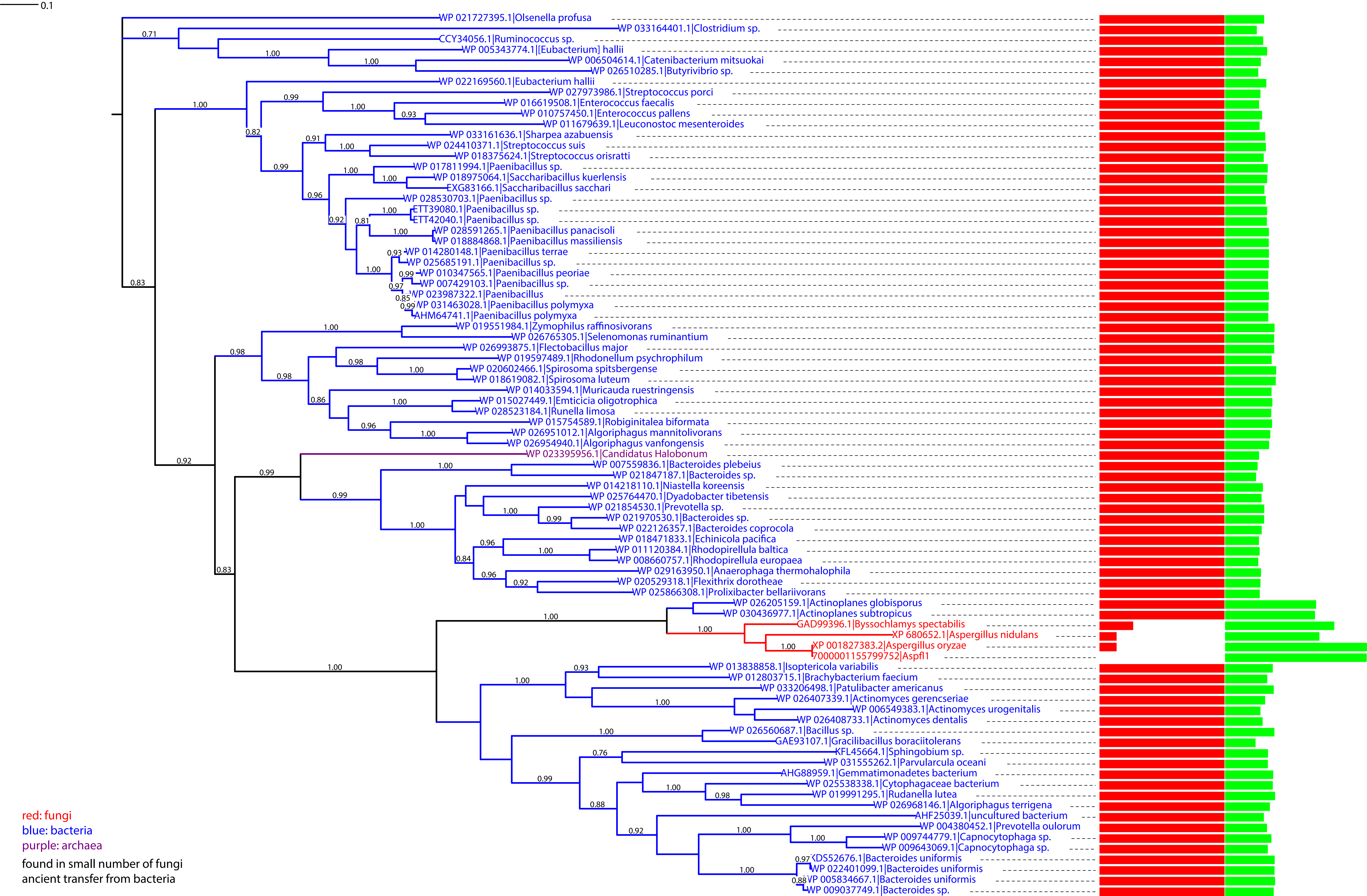
0.1
WP 021727395.1|Olsenella profusa
WP 033164401.1|Clostridium sp.
CCY34056.1|Ruminococcus sp.
WP 005343774.1|[Eubacterium] hallii
WP 006504614.1|Catenibacterium mitsuokai
WP 026510285.1|Butyrivibrio sp.
WP 022169560.1|Eubacterium hallii
WP 027973986.1|Streptococcus porci
WP 016619508.1|Enterococcus faecalis
WP 010757450.1|Enterococcus pallens
WP 011679639.1|Leuconostoc mesenteroides
WP 033161636.1|Sharpea azabuensis
WP 024410371.1|Streptococcus suis
WP 018375624.1|Streptococcus orisratti
WP 017811994.1|Paenibacillus sp.
WP 018975064.1|Saccharibacillus kuerlensis
EXG83166.1|Saccharibacillus sacchari
WP 028530703.1|Paenibacillus sp.
ETT39080.1|Paenibacillus sp.
ETT42040.1|Paenibacillus sp.
WP 028591265.1|Paenibacillus panacisoli
WP 018884868.1|Paenibacillus massiliensis
WP 014280148.1|Paenibacillus terrae
WP 025685191.1|Paenibacillus sp.
WP 010347565.1|Paenibacillus peoriae
WP 007429103.1|Paenibacillus sp.
WP 023987322.1|Paenibacillus
WP 031463028.1|Paenibacillus polymyxa
AHM64741.1|Paenibacillus polymyxa
WP 019551984.1|Zymophilus raffinosivorans
WP 026765305.1|Selenomonas ruminantium
WP 026993875.1|Flectobacillus major
WP 019597489.1|Rhodonellum psychrophilum
WP 020602466.1|Spirosoma spitsbergense
WP 018619082.1|Spirosoma luteum
WP 014033594.1|Muricauda ruestringensis
WP 015027449.1|Emticicia oligotrophica
WP 028523184.1|Runella limosa
WP 015754589.1|Robiginitalea biformata
WP 026951012.1|Algoriphagus mannitolivorans
WP 026954940.1|Algoriphagus vanfongensis
WP 023395956.1|Candidatus Halobonum
WP 007559836.1|Bacteroides plebeius
WP 021847187.1|Bacteroides sp.
WP 014218110.1|Niastella koreensis
WP 025764470.1|Dyadobacter tibetensis
WP 021854530.1|Prevotella sp.
WP 021970530.1|Bacteroides sp.
WP 022126357.1|Bacteroides coprocola
WP 018471833.1|Echinicola pacifica
WP 011120384.1|Rhodopirellula baltica
WP 008660757.1|Rhodopirellula europaea
WP 029163950.1|Anaerophaga thermohalophila
WP 020529318.1|Flexithrix dorotheae
WP 025866308.1|Prolixibacter bellariivorans
WP 026205159.1|Actinoplanes globisporus
WP 030436977.1|Actinoplanes subtropicus
GAD99396.1|Byssochlamys spectabilis
XP 680652.1|Aspergillus nidulans
XP 001827383.2|Aspergillus oryzae
7000001155799752|Aspfl1
WP 013838858.1|Isoptericola variabilis
WP 012803715.1|Brachybacterium faecium
WP 033206498.1|Patulibacter americanus
WP 026407339.1|Actinomyces gerencseriae
WP 006549383.1|Actinomyces urogenitalis
WP 026408733.1|Actinomyces dentalis
WP 026560687.1|Bacillus sp.
GAE93107.1|Gracilibacillus boraciitolerans
KFL45664.1|Sphingobium sp.
WP 031555262.1|Parvularcula oceani
AHG88959.1|Gemmatimonadetes bacterium
WP 025538338.1|Cytophagaceae bacterium
WP 019991295.1|Rudanella lutea
WP 026968146.1|Algoriphagus terrigena
AHF25039.1|uncultured bacterium
WP 004380452.1|Prevotella oulorum
WP 009744779.1|Capnocytophaga sp.
WP 009643069.1|Capnocytophaga sp.
KDS52676.1|Bacteroides uniformis
WP 022401099.1|Bacteroides uniformis
WP 005834667.1|Bacteroides uniformis
WP 009037749.1|Bacteroides sp.
red: fungi
blue: bacteria
purple: archaea
found in small number of fungi
ancient transfer from bacteria

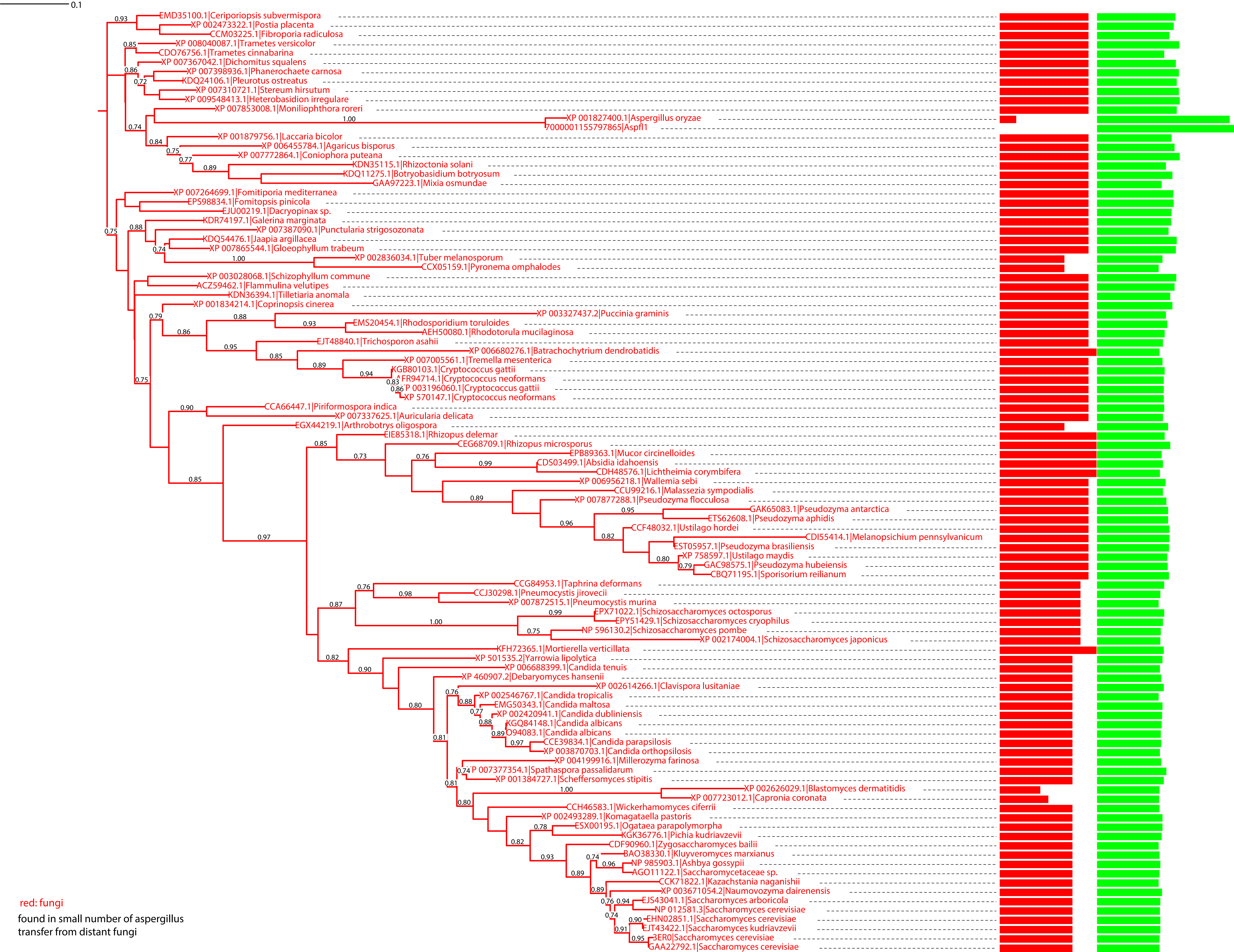
0.1
EMD35100.1|Ceriporiopsis subvermispora
XP 002473322.1|Postia placenta
CCM03225.1|Fibroporia radiculosa
XP 008040087.1|Trametes versicolor
CDO76756.1|Trametes cinnabarina
XP 007367042.1|Dichomitus squalens
XP 007398936.1|Phanerochaete carnosa
KDQ24106.1|Pleurotus ostreatus
XP 007310721.1|Stereum hirsutum
XP 009548413.1|Heterobasidion irregulare
XP 007853008.1|Moniliophthora roreri
XP 001827400.1|Aspergillus oryzae
7000001155797865|Aspfl1
XP 001879756.1|Laccaria bicolor
XP 006455784.1|Agaricus bisporus
XP 007772864.1|Coniophora puteana
KDN35115.1|Rhizoctonia solani
KDQ11275.1|Botryobasidium botryosum
GAA97223.1|Mixia osmundae
XP 007264699.1|Fomitiporia mediterranea
EPS98834.1|Fomitopsis pinicola
EJU00219.1|Dacryopinax sp.
KDR74197.1|Galerina marginata
XP 007387090.1|Punctularia strigosozonata
KDQ54476.1|Jaapia argillacea
XP 007865544.1|Gloeophyllum trabeum
XP 002836034.1|Tuber melanosporum
CCX05159.1|Pyronema omphalodes
XP 003028068.1|Schizophyllum commune
ACZ59462.1|Flammulina velutipes
KDN36394.1|Tilletiaria anomala
XP 001834214.1|Coprinopsis cinerea
XP 003327437.2|Puccinia graminis
EMS20454.1|Rhodosporidium toruloides
AEH50080.1|Rhodotorula mucilaginosa
EJT48840.1|Trichosporon asahii
XP 006680276.1|Batrachochytrium dendrobatidis
XP 007005561.1|Tremella mesenterica
KGB80103.1|Cryptococcus gattii
AFR94714.1|Cryptococcus neoformans
XP 003196060.1|Cryptococcus gattii
XP 570147.1|Cryptococcus neoformans
CCA66447.1|Piriformospora indica
XP 007337625.1|Auricularia delicata
EGX44219.1|Arthrobotrys oligospora
EIE85318.1|Rhizopus delemar
CEG68709.1|Rhizopus microsporus
EPB89363.1|Mucor circinelloides
CDS03499.1|Absidia idahoensis
CDH48576.1|Lichtheimia corymbifera
XP 006956218.1|Wallemia sebi
CCU99216.1|Malassezia sympodialis
XP 007877288.1|Pseudozyma flocculosa
GAK65083.1|Pseudozyma antarctica
ETS62608.1|Pseudozyma aphidis
CCF48032.1|Ustilago hordei
CDI55414.1|Melanopsichium pennsylvanicum
EST05957.1|Pseudozyma brasiliensis
XP 758597.1|Ustilago maydis
GAC98575.1|Pseudozyma hubeiensis
CBQ71195.1|Sporisorium reilianum
CCG84953.1|Taphrina deformans
CCJ30298.1|Pneumocystis jirovecii
XP 007872515.1|Pneumocystis murina
EPX71022.1|Schizosaccharomyces octosporus
EPY51429.1|Schizosaccharomyces cryophilus
NP 596130.2|Schizosaccharomyces pombe
XP 002174004.1|Schizosaccharomyces japonicus
KFH72365.1|Mortierella verticillata
XP 501535.2|Yarrowia lipolytica
XP 006688399.1|Candida tenuis
XP 460907.2|Debaryomyces hansenii
XP 002614266.1|Clavispora lusitaniae
XP 002546767.1|Candida tropicalis
EMG50343.1|Candida maltosa
XP 002420941.1|Candida dubliniensis
KGQ84148.1|Candida albicans
O94083.1|Candida albicans
CCE39834.1|Candida parapsilosis
XP 003870703.1|Candida orthopsilosis
XP 004199916.1|Millerozyma farinosa
XP 007377354.1|Spathaspora passalidarum
XP 001384727.1|Scheffersomyces stipitis
XP 002626029.1|Blastomyces dermatitidis
XP 007723012.1|Capronia coronata
CCH46583.1|Wickerhamomyces ciferrii
XP 002493289.1|Komagataella pastoris
ESX00195.1|Ogataea parapolymorpha
KGK36776.1|Pichia kudriavzevii
CDF90960.1|Zygosaccharomyces bailii
BAO38330.1|Kluyveromyces marxianus
NP 985903.1|Ashbya gossypii
AGO11122.1|Saccharomycetaceae sp.
CCK71822.1|Kazachstania naganishii
XP 003671054.2|Naumovozyma dairenensis
EJS43041.1|Saccharomyces arboricola
NP 012581.3|Saccharomyces cerevisiae
EHN02851.1|Saccharomyces cerevisiae
EJT43422.1|Saccharomyces kudriavzevii
3ER0|Saccharomyces cerevisiae
GAA22792.1|Saccharomyces cerevisiae
red: fungi
found in small number of aspergillus
transfer from distant fungi

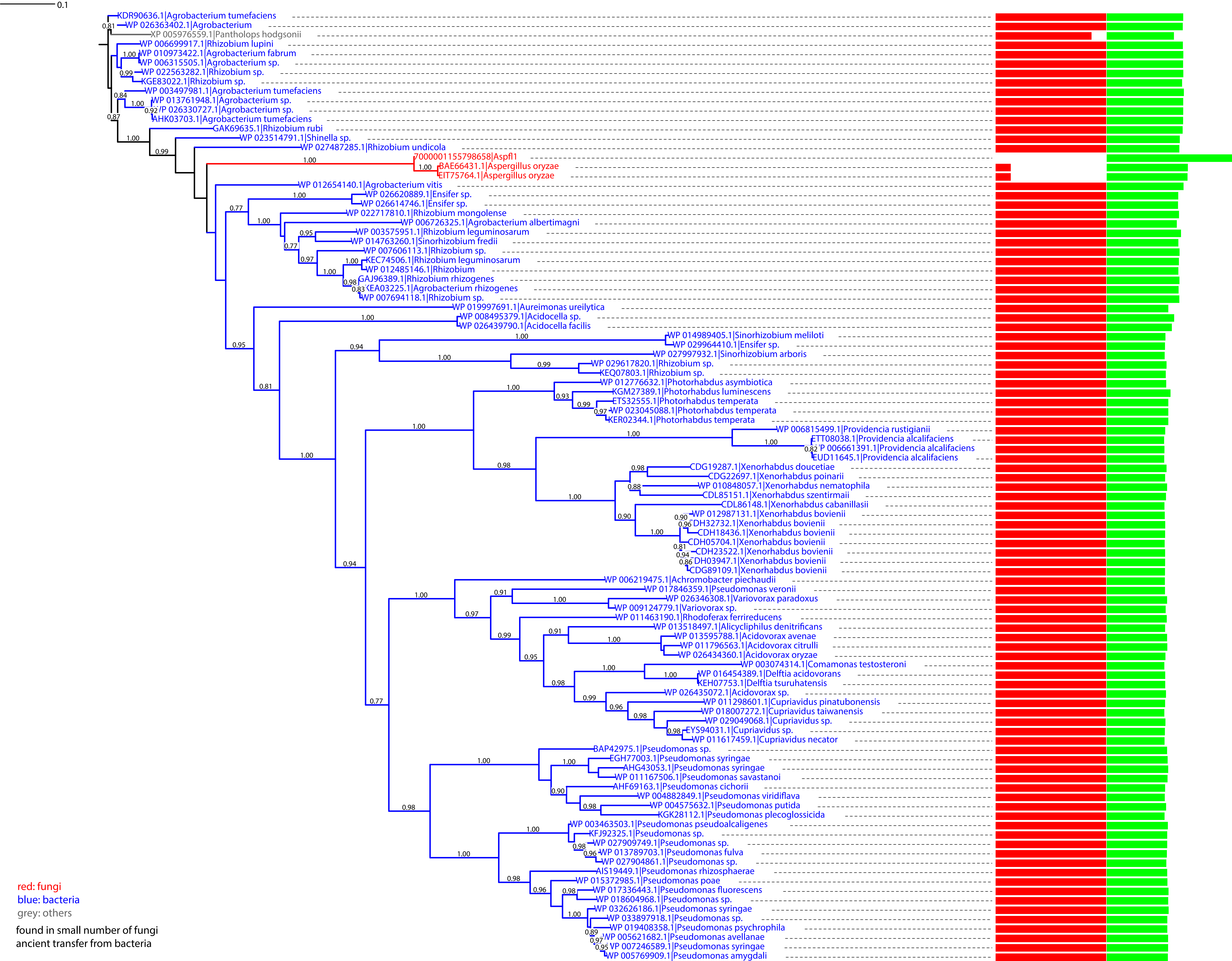

0.1
KDR90636.1|Agrobacterium tumefaciens
WP 026363402.1|Agrobacterium
XP 005976559.1|Pantholops hodgsonii
WP 006699917.1|Rhizobium lupini
WP 010973422.1|Agrobacterium fabrum
WP 006315505.1|Agrobacterium sp.
WP 022563282.1|Rhizobium sp.
KGE83022.1|Rhizobium sp.
WP 003497981.1|Agrobacterium tumefaciens
WP 013761948.1|Agrobacterium sp.
P 026330727.1|Agrobacterium sp.
AHK03703.1|Agrobacterium tumefaciens
GAK69635.1|Rhizobium rubi
WP 023514791.1|Shinella sp.
WP 027487285.1|Rhizobium undicola
7000001155798658|Aspfl1
BAE66431.1|Aspergillus oryzae
EIT75764.1|Aspergillus oryzae
WP 012654140.1|Agrobacterium vitis
WP 026620889.1|Ensifer sp.
WP 026614746.1|Ensifer sp.
WP 022717810.1|Rhizobium mongolense
WP 006726325.1|Agrobacterium albertimagni
WP 003575951.1|Rhizobium leguminosarum
WP 014763260.1|Sinorhizobium fredii
WP 007606113.1|Rhizobium sp.
KEC74506.1|Rhizobium leguminosarum
WP 012485146.1|Rhizobium
GAJ96389.1|Rhizobium rhizogenes
KEA03225.1|Agrobacterium rhizogenes
WP 007694118.1|Rhizobium sp.
WP 019997691.1|Aureimonas ureilytica
WP 008495379.1|Acidocella sp.
WP 026439790.1|Acidocella facilis
WP 014989405.1|Sinorhizobium meliloti
WP 029964410.1|Ensifer sp.
WP 027997932.1|Sinorhizobium arboris
WP 029617820.1|Rhizobium sp.
KEQ07803.1|Rhizobium sp.
WP 012776632.1|Photorhabdus asymbiotica
KGM27389.1|Photorhabdus luminescens
ETS32555.1|Photorhabdus temperata
WP 023045088.1|Photorhabdus temperata
KER02344.1|Photorhabdus temperata
WP 006815499.1|Providencia rustigianii
ETT08038.1|Providencia alcalifaciens
P 006661391.1|Providencia alcalifaciens
EUD11645.1|Providencia alcalifaciens
CDG19287.1|Xenorhabdus doucetiae
CDG22697.1|Xenorhabdus poinarii
WP 010848057.1|Xenorhabdus nematophila
CDL85151.1|Xenorhabdus szentirmaii
CDL86148.1|Xenorhabdus cabanillasii
WP 012987131.1|Xenorhabdus bovienii
DH32732.1|Xenorhabdus bovienii
CDH18436.1|Xenorhabdus bovienii
CDH05704.1|Xenorhabdus bovienii
CDH23522.1|Xenorhabdus bovienii
DH03947.1|Xenorhabdus bovienii
CDG89109.1|Xenorhabdus bovienii
WP 006219475.1|Achromobacter piechaudii
WP 017846359.1|Pseudomonas veronii
WP 026346308.1|Variovorax paradoxus
WP 009124779.1|Variovorax sp.
WP 011463190.1|Rhodoferax ferrireducens
WP 013518497.1|Alicycliphilus denitrificans
WP 013595788.1|Acidovorax avenae
WP 011796563.1|Acidovorax citrulli
WP 026434360.1|Acidovorax oryzae
WP 003074314.1|Comamonas testosteroni
WP 016454389.1|Delftia acidovorans
KEH07753.1|Delftia tsuruhatensis
WP 026435072.1|Acidovorax sp.
WP 011298601.1|Cupriavidus pinatubonensis
WP 018007272.1|Cupriavidus taiwanensis
WP 029049068.1|Cupriavidus sp.
EYS94031.1|Cupriavidus sp.
WP 011617459.1|Cupriavidus necator
BAP42975.1|Pseudomonas sp.
EGH77003.1|Pseudomonas syringae
AHG43053.1|Pseudomonas syringae
WP 011167506.1|Pseudomonas savastanoi
AHF69163.1|Pseudomonas cichorii
WP 004882849.1|Pseudomonas viridiflava
WP 004575632.1|Pseudomonas putida
KGK28112.1|Pseudomonas plecoglossicida
WP 003463503.1|Pseudomonas pseudoalcaligenes
KFJ92325.1|Pseudomonas sp.
WP 027909749.1|Pseudomonas sp.
WP 013789703.1|Pseudomonas fulva
WP 027904861.1|Pseudomonas sp.
AIS19449.1|Pseudomonas rhizosphaerae
WP 015372985.1|Pseudomonas poae
WP 017336443.1|Pseudomonas fluorescens
WP 018604968.1|Pseudomonas sp.
WP 032626186.1|Pseudomonas syringae
WP 033897918.1|Pseudomonas sp.
WP 019408358.1|Pseudomonas psychrophila
WP 005621682.1|Pseudomonas avellanae
WP 007246589.1|Pseudomonas syringae
WP 005769909.1|Pseudomonas amygdali
red: fungi
blue: bacteria
grey: others
found in small number of fungi
ancient transfer from bacteria

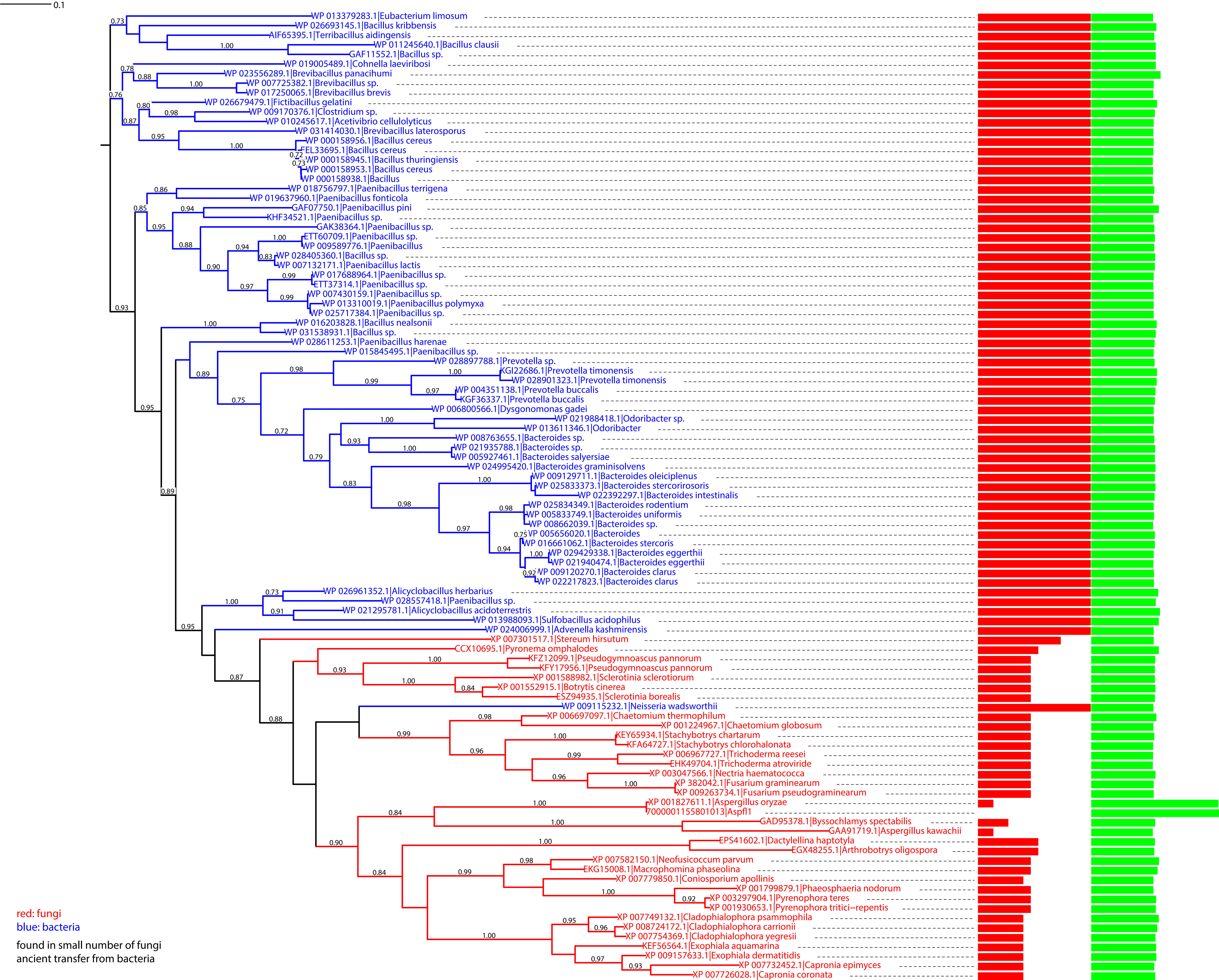
0.1
WP 013379283.1|Eubacterium limosum
WP 026693145.1|Bacillus kribbensis
AIF65395.1|Terribacillus aidingensis
WP 011245640.1|Bacillus clausii
GAF11552.1|Bacillus sp.
WP 019005489.1|Cohnella laeviribosi
WP 023556289.1|Brevibacillus panacihumi
WP 007725382.1|Brevibacillus sp.
WP 017250065.1|Brevibacillus brevis
WP 026679479.1|Fictibacillus gelatini
WP 009170376.1|Clostridium sp.
WP 010245617.1|Acetivibrio cellulolyticus
WP 031414030.1|Brevibacillus laterosporus
WP 000158956.1|Bacillus cereus
EEL33695.1|Bacillus cereus
WP 000158945.1|Bacillus thuringiensis
WP 000158953.1|Bacillus cereus
WP 000158938.1|Bacillus
WP 018756797.1|Paenibacillus terrigena
WP 019637960.1|Paenibacillus fonticola
GAF07750.1|Paenibacillus pini
KHF34521.1|Paenibacillus sp.
GAK38364.1|Paenibacillus sp.
ETT60709.1|Paenibacillus sp.
WP 009589776.1|Paenibacillus
WP 028405360.1|Bacillus sp.
WP 007132171.1|Paenibacillus lactis
WP 017688964.1|Paenibacillus sp.
ETT37314.1|Paenibacillus sp.
WP 007430159.1|Paenibacillus sp.
WP 013310019.1|Paenibacillus polymyxa
WP 025717384.1|Paenibacillus sp.
WP 016203828.1|Bacillus nealsonii
WP 031538931.1|Bacillus sp.
WP 028611253.1|Paenibacillus harenae
WP 015845495.1|Paenibacillus sp.
WP 028897788.1|Prevotella sp.
KGI22686.1|Prevotella timonensis
WP 028901323.1|Prevotella timonensis
WP 004351138.1|Prevotella buccalis
KGF36337.1|Prevotella buccalis
WP 006800566.1|Dysgonomonas gadei
WP 021988418.1|Odoribacter sp.
WP 013611346.1|Odoribacter
WP 008763655.1|Bacteroides sp.
WP 021935788.1|Bacteroides sp.
WP 005927461.1|Bacteroides salyersiae
WP 024995420.1|Bacteroides graminisolvens
WP 009129711.1|Bacteroides oleiciplenus
WP 025833373.1|Bacteroides stercorirosoris
WP 022392297.1|Bacteroides intestinalis
WP 025834349.1|Bacteroides rodentium
WP 005833749.1|Bacteroides uniformis
WP 008662039.1|Bacteroides sp.
WP 005656020.1|Bacteroides
WP 016661062.1|Bacteroides stercoris
WP 029429338.1|Bacteroides eggerthii
WP 021940474.1|Bacteroides eggerthii
WP 009120270.1|Bacteroides clarus
WP 022217823.1|Bacteroides clarus
WP 026961352.1|Alicyclobacillus herbarius
WP 028557418.1|Paenibacillus sp.
WP 021295781.1|Alicyclobacillus acidoterrestris
WP 013988093.1|Sulfobacillus acidophilus
WP 024006999.1|Advenella kashmirensis
XP 007301517.1|Stereum hirsutum
CCX10695.1|Pyronema omphalodes
KFZ12099.1|Pseudogymnoascus pannorum
KFY17956.1|Pseudogymnoascus pannorum
XP 001588982.1|Sclerotinia sclerotiorum
XP 001552915.1|Botrytis cinerea
ESZ94935.1|Sclerotinia borealis
WP 009115232.1|Neisseria wadsworthii
XP 006697097.1|Chaetomium thermophilum
XP 001224967.1|Chaetomium globosum
KEY65934.1|Stachybotrys chartarum
KFA64727.1|Stachybotrys chlorohalonata
XP 006967727.1|Trichoderma reesei
EHK49704.1|Trichoderma atroviride
XP 003047566.1|Nectria haematococca
XP 382042.1|Fusarium graminearum
XP 009263734.1|Fusarium pseudograminearum
XP 001827611.1|Aspergillus oryzae
7000001155801013|Aspfl1
GAD95378.1|Byssochlamys spectabilis
GAA91719.1|Aspergillus kawachii
EPS41602.1|Dactylellina haptotyla
EGX48255.1|Arthrobotrys oligospora
XP 007582150.1|Neofusicoccum parvum
EKG15008.1|Macrophomina phaseolina
XP 007779850.1|Coniosporium apollinis
XP 001799879.1|Phaeosphaeria nodorum
XP 003297904.1|Pyrenophora teres
XP 001930653.1|Pyrenophora tritici−repentis
XP 007749132.1|Cladophialophora psammophila
XP 008724172.1|Cladophialophora carrionii
XP 007754369.1|Cladophialophora yegresii
KEF56564.1|Exophiala aquamarina
XP 009157633.1|Exophiala dermatitidis
XP 007732452.1|Capronia epimyces
XP 007726028.1|Capronia coronata
red: fungi
blue: bacteria
found in small number of fungi
ancient transfer from bacteria

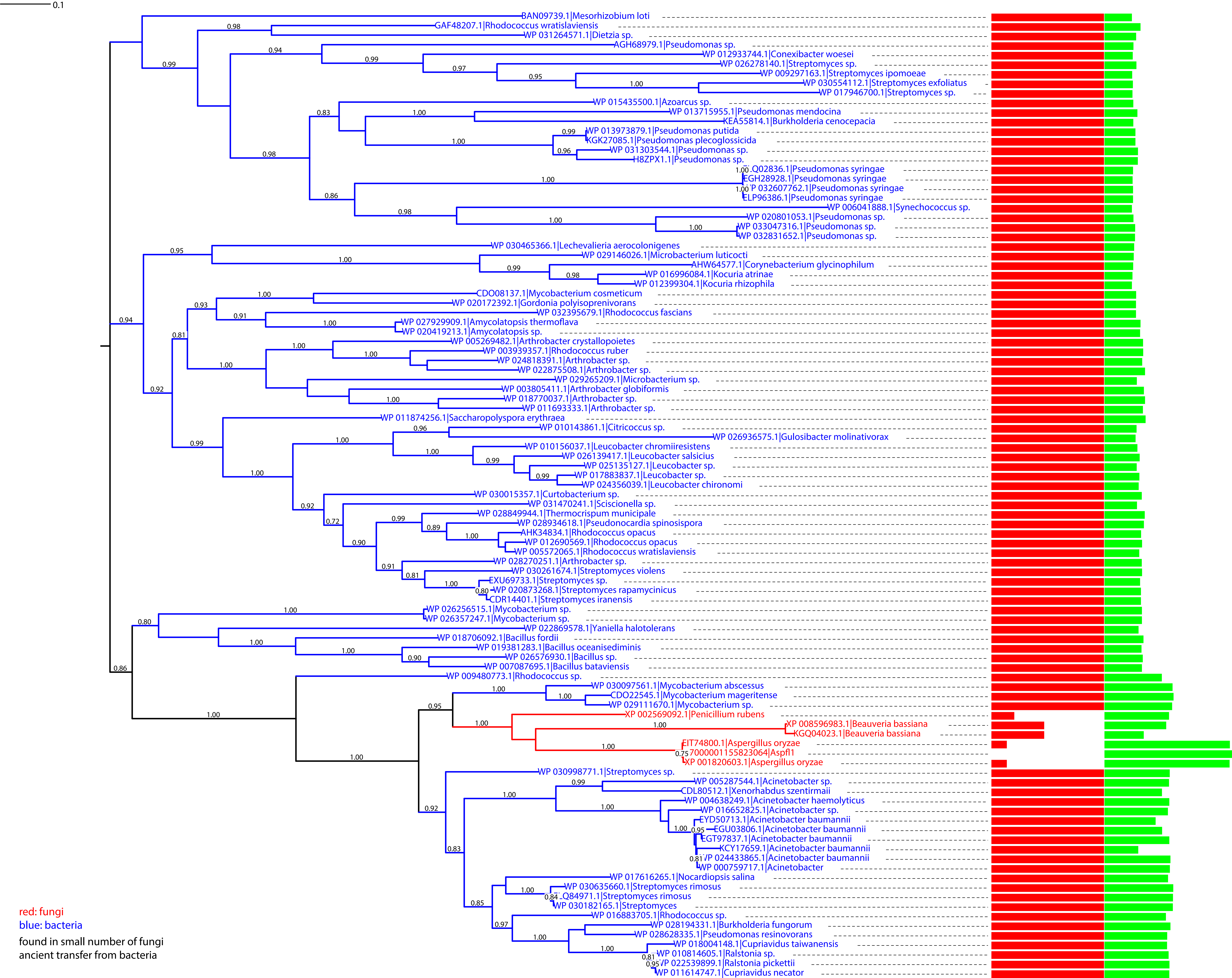
0.1
BAN09739.1|Mesorhizobium loti
GAF48207.1|Rhodococcus wratislaviensis
WP 031264571.1|Dietzia sp.
AGH68979.1|Pseudomonas sp.
WP 012933744.1|Conexibacter woesei
WP 026278140.1|Streptomyces sp.
WP 009297163.1|Streptomyces ipomoeae
WP 030554112.1|Streptomyces exfoliatus
WP 017946700.1|Streptomyces sp.
WP 015435500.1|Azoarcus sp.
WP 013715955.1|Pseudomonas mendocina
KEA55814.1|Burkholderia cenocepacia
WP 013973879.1|Pseudomonas putida
KGK27085.1|Pseudomonas plecoglossicida
WP 031303544.1|Pseudomonas sp.
H8ZPX1.1|Pseudomonas sp.
Q02836.1|Pseudomonas syringae
EGH28928.1|Pseudomonas syringae
P 032607762.1|Pseudomonas syringae
ELP96386.1|Pseudomonas syringae
WP 006041888.1|Synechococcus sp.
WP 020801053.1|Pseudomonas sp.
WP 033047316.1|Pseudomonas sp.
WP 032831652.1|Pseudomonas sp.
WP 030465366.1|Lechevalieria aerocolonigenes
WP 029146026.1|Microbacterium luticocti
AHW64577.1|Corynebacterium glycinophilum
WP 016996084.1|Kocuria atrinae
WP 012399304.1|Kocuria rhizophila
CDO08137.1|Mycobacterium cosmeticum
WP 020172392.1|Gordonia polyisoprenivorans
WP 032395679.1|Rhodococcus fascians
WP 027929909.1|Amycolatopsis thermoflava
WP 020419213.1|Amycolatopsis sp.
WP 005269482.1|Arthrobacter crystallopoietes
WP 003939357.1|Rhodococcus ruber
WP 024818391.1|Arthrobacter sp.
WP 022875508.1|Arthrobacter sp.
WP 029265209.1|Microbacterium sp.
WP 003805411.1|Arthrobacter globiformis
WP 018770037.1|Arthrobacter sp.
WP 011693333.1|Arthrobacter sp.
WP 011874256.1|Saccharopolyspora erythraea
WP 010143861.1|Citricoccus sp.
WP 026936575.1|Gulosibacter molinativorax
WP 010156037.1|Leucobacter chromiiresistens
WP 026139417.1|Leucobacter salsicius
WP 025135127.1|Leucobacter sp.
WP 017883837.1|Leucobacter sp.
WP 024356039.1|Leucobacter chironomi
WP 030015357.1|Curtobacterium sp.
WP 031470241.1|Sciscionella sp.
WP 028849944.1|Thermocrispum municipale
WP 028934618.1|Pseudonocardia spinosispora
AHK34834.1|Rhodococcus opacus
WP 012690569.1|Rhodococcus opacus
WP 005572065.1|Rhodococcus wratislaviensis
WP 028270251.1|Arthrobacter sp.
WP 030261674.1|Streptomyces violens
EXU69733.1|Streptomyces sp.
WP 020873268.1|Streptomyces rapamycinicus
CDR14401.1|Streptomyces iranensis
WP 026256515.1|Mycobacterium sp.
WP 026357247.1|Mycobacterium sp.
WP 022869578.1|Yaniella halotolerans
WP 018706092.1|Bacillus fordii
WP 019381283.1|Bacillus oceanisediminis
WP 026576930.1|Bacillus sp.
WP 007087695.1|Bacillus bataviensis
WP 009480773.1|Rhodococcus sp.
WP 030097561.1|Mycobacterium abscessus
CDO22545.1|Mycobacterium mageritense
WP 029111670.1|Mycobacterium sp.
XP 002569092.1|Penicillium rubens
XP 008596983.1|Beauveria bassiana
KGQ04023.1|Beauveria bassiana
EIT74800.1|Aspergillus oryzae
7000001155823064|Aspfl1
XP 001820603.1|Aspergillus oryzae
WP 030998771.1|Streptomyces sp.
WP 005287544.1|Acinetobacter sp.
CDL80512.1|Xenorhabdus szentirmaii
WP 004638249.1|Acinetobacter haemolyticus
WP 016652825.1|Acinetobacter sp.
EYD50713.1|Acinetobacter baumannii
EGU03806.1|Acinetobacter baumannii
EGT97837.1|Acinetobacter baumannii
KCY17659.1|Acinetobacter baumannii
VP 024433865.1|Acinetobacter baumannii
WP 000759717.1|Acinetobacter
WP 017616265.1|Nocardiopsis salina
WP 030635660.1|Streptomyces rimosus
Q84971.1|Streptomyces rimosus
WP 030182165.1|Streptomyces
WP 016883705.1|Rhodococcus sp.
WP 028194331.1|Burkholderia fungorum
WP 028628335.1|Pseudomonas resinovorans
WP 018004148.1|Cupriavidus taiwanensis
WP 010814605.1|Ralstonia sp.
VP 022539899.1|Ralstonia pickettii
WP 011614747.1|Cupriavidus necator
red: fungi
blue: bacteria
found in small number of fungi
ancient transfer from bacteria

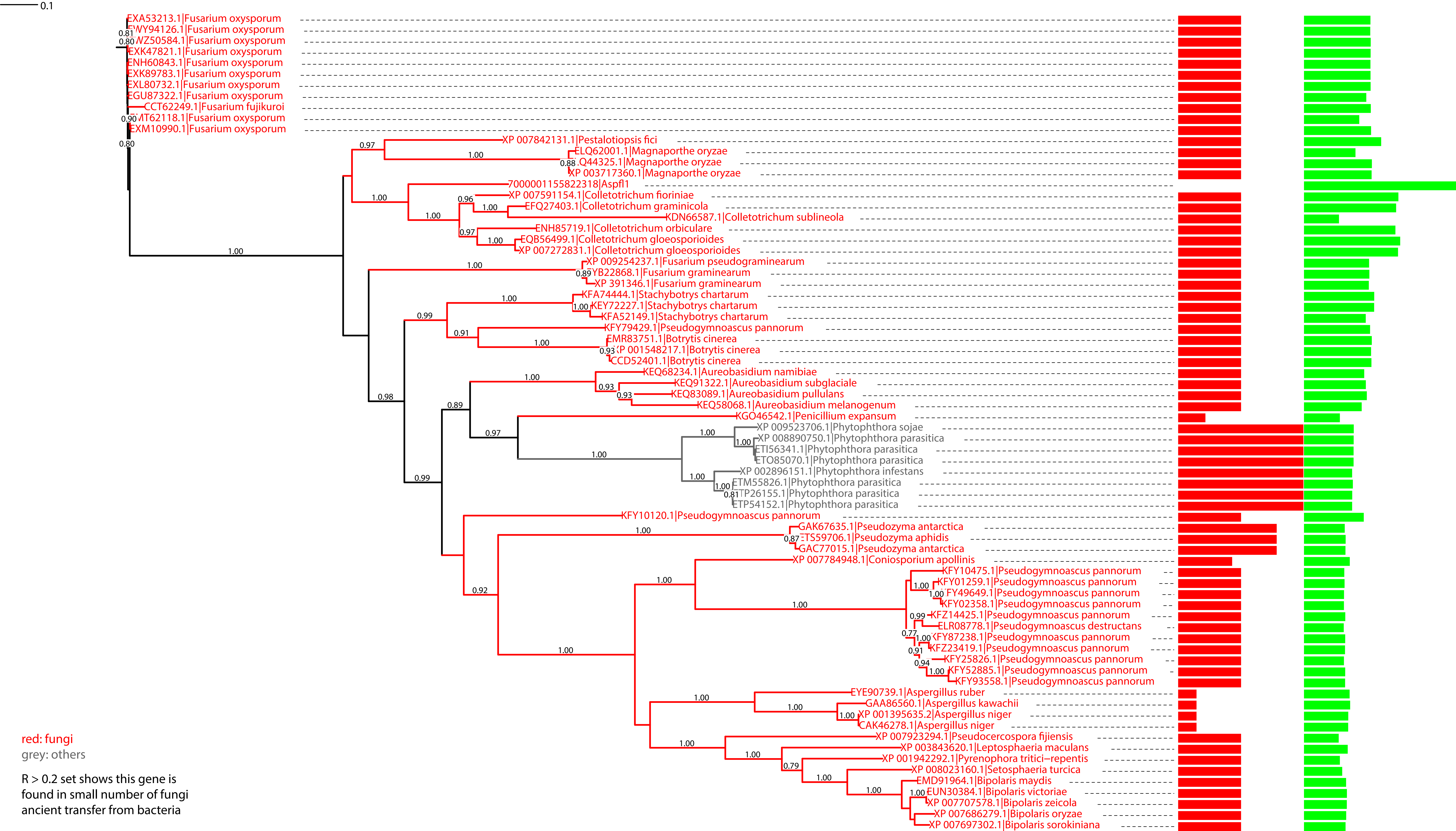
0.1
EXA53213.1|Fusarium oxysporum
WY94126.1|Fusarium oxysporum
NZ50584.1|Fusarium oxysporum
EXK47821.1|Fusarium oxysporum
ENH60843.1|Fusarium oxysporum
EXK89783.1|Fusarium oxysporum
EXL80732.1|Fusarium oxysporum
EGU87322.1|Fusarium oxysporum
CCT62249.1|Fusarium fujikuroi
MT62118.1|Fusarium oxysporum
EXM10990.1|Fusarium oxysporum
XP 007842131.1|Pestalotiopsis fici
ELQ62001.1|Magnaporthe oryzae
Q44325.1|Magnaporthe oryzae
XP 003717360.1|Magnaporthe oryzae
7000001155822318|Aspfl1
XP 007591154.1|Colletotrichum fioriniae
EFQ27403.1|Colletotrichum graminicola
KDN66587.1|Colletotrichum sublineola
ENH85719.1|Colletotrichum orbiculare
EQB56499.1|Colletotrichum gloeosporioides
XP 007272831.1|Colletotrichum gloeosporioides
XP 009254237.1|Fusarium pseudograminearum
YB22868.1|Fusarium graminearum
XP 391346.1|Fusarium graminearum
KFA74444.1|Stachybotrys chartarum
KEY72227.1|Stachybotrys chartarum
KFA52149.1|Stachybotrys chartarum
KFY79429.1|Pseudogymnoascus pannorum
EMR83751.1|Botrytis cinerea
P 001548217.1|Botrytis cinerea
CCD52401.1|Botrytis cinerea
KEQ68234.1|Aureobasidium namibiae
KEQ91322.1|Aureobasidium subglaciale
KEQ83089.1|Aureobasidium pullulans
KEQ58068.1|Aureobasidium melanogenum
KGO46542.1|Penicillium expansum
XP 009523706.1|Phytophthora sojae
XP 008890750.1|Phytophthora parasitica
ETI56341.1|Phytophthora parasitica
ETO85070.1|Phytophthora parasitica
XP 002896151.1|Phytophthora infestans
ETM55826.1|Phytophthora parasitica
TP26155.1|Phytophthora parasitica
ETP54152.1|Phytophthora parasitica
KFY10120.1|Pseudogymnoascus pannorum
GAK67635.1|Pseudozyma antarctica
TS59706.1|Pseudozyma aphidis
GAC77015.1|Pseudozyma antarctica
XP 007784948.1|Coniosporium apollinis
KFY10475.1|Pseudogymnoascus pannorum
KFY01259.1|Pseudogymnoascus pannorum
FY49649.1|Pseudogymnoascus pannorum
KFY02358.1|Pseudogymnoascus pannorum
KFZ14425.1|Pseudogymnoascus pannorum
ELR08778.1|Pseudogymnoascus destructans
KFY87238.1|Pseudogymnoascus pannorum
KFZ23419.1|Pseudogymnoascus pannorum
KFY25826.1|Pseudogymnoascus pannorum
KFY52885.1|Pseudogymnoascus pannorum
KFY93558.1|Pseudogymnoascus pannorum
EYE90739.1|Aspergillus ruber
GAA86560.1|Aspergillus kawachii
XP 001395635.2|Aspergillus niger
CAK46278.1|Aspergillus niger
XP 007923294.1|Pseudocercospora fijiensis
XP 003843620.1|Leptosphaeria maculans
XP 001942292.1|Pyrenophora tritici−repentis
XP 008023160.1|Setosphaeria turcica
EMD91964.1|Bipolaris maydis
EUN30384.1|Bipolaris victoriae
XP 007707578.1|Bipolaris zeicola
XP 007686279.1|Bipolaris oryzae
XP 007697302.1|Bipolaris sorokiniana
red: fungi
grey: others
R > 0.2 set shows this gene is
found in small number of fungi
ancient transfer from bacteria

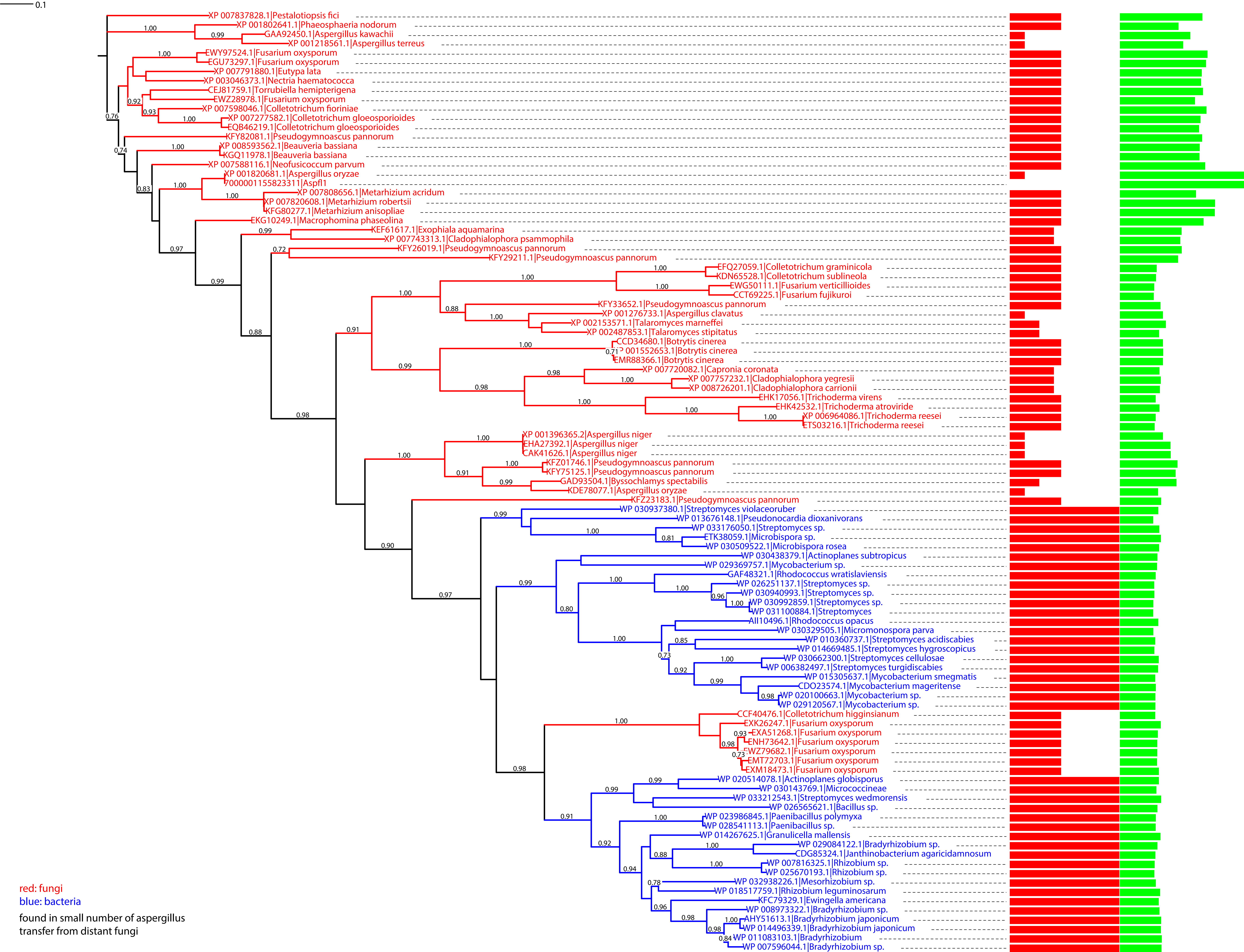
0.1
XP 007837828.1|Pestalotiopsis fici
XP 001802641.1|Phaeosphaeria nodorum
GAA92450.1|Aspergillus kawachii
XP 001218561.1|Aspergillus terreus
EWY97524.1|Fusarium oxysporum
EGU73297.1|Fusarium oxysporum
XP 007791880.1|Eutypa lata
XP 003046373.1|Nectria haematococca
CEJ81759.1|Torrubiella hemipterigena
EWZ28978.1|Fusarium oxysporum
XP 007598046.1|Colletotrichum fioriniae
XP 007277582.1|Colletotrichum gloeosporioides
EQB46219.1|Colletotrichum gloeosporioides
KFY82081.1|Pseudogymnoascus pannorum
XP 008593562.1|Beauveria bassiana
KGQ11978.1|Beauveria bassiana
XP 007588116.1|Neofusicoccum parvum
XP 001820681.1|Aspergillus oryzae
7000001155823311|Aspfl1
XP 007808656.1|Metarhizium acridum
XP 007820608.1|Metarhizium robertsii
KFG80277.1|Metarhizium anisopliae
EKG10249.1|Macrophomina phaseolina
KEF61617.1|Exophiala aquamarina
XP 007743313.1|Cladophialophora psammophila
KFY26019.1|Pseudogymnoascus pannorum
KFY29211.1|Pseudogymnoascus pannorum
EFQ27059.1|Colletotrichum graminicola
KDN65528.1|Colletotrichum sublineola
EWG50111.1|Fusarium verticillioides
CCT69225.1|Fusarium fujikuroi
KFY33652.1|Pseudogymnoascus pannorum
XP 001276733.1|Aspergillus clavatus
XP 002153571.1|Talaromyces marneffei
XP 002487853.1|Talaromyces stipitatus
CCD34680.1|Botrytis cinerea
001552653.1|Botrytis cinerea
EMR88366.1|Botrytis cinerea
XP 007720082.1|Capronia coronata
XP 007757232.1|Cladophialophora yegresii
XP 008726201.1|Cladophialophora carrionii
EHK17056.1|Trichoderma virens
EHK42532.1|Trichoderma atroviride
XP 006964086.1|Trichoderma reesei
ETS03216.1|Trichoderma reesei
XP 001396365.2|Aspergillus niger
EHA27392.1|Aspergillus niger
CAK41626.1|Aspergillus niger
KFZ01746.1|Pseudogymnoascus pannorum
KFY75125.1|Pseudogymnoascus pannorum
GAD93504.1|Byssochlamys spectabilis
KDE78077.1|Aspergillus oryzae
KFZ23183.1|Pseudogymnoascus pannorum
WP 030937380.1|Streptomyces violaceoruber
WP 013676148.1|Pseudonocardia dioxanivorans
WP 033176050.1|Streptomyces sp.
ETK38059.1|Microbispora sp.
WP 030509522.1|Microbispora rosea
WP 030438379.1|Actinoplanes subtropicus
WP 029369757.1|Mycobacterium sp.
GAF48321.1|Rhodococcus wratislaviensis
WP 026251137.1|Streptomyces sp.
WP 030940993.1|Streptomyces sp.
WP 030992859.1|Streptomyces sp.
WP 031100884.1|Streptomyces
AII10496.1|Rhodococcus opacus
WP 030329505.1|Micromonospora parva
WP 010360737.1|Streptomyces acidiscabies
WP 014669485.1|Streptomyces hygroscopicus
WP 030662300.1|Streptomyces cellulosae
WP 006382497.1|Streptomyces turgidiscabies
WP 015305637.1|Mycobacterium smegmatis
CDO23574.1|Mycobacterium mageritense
WP 020100663.1|Mycobacterium sp.
WP 029120567.1|Mycobacterium sp.
CCF40476.1|Colletotrichum higginsianum
EXK26247.1|Fusarium oxysporum
EXA51268.1|Fusarium oxysporum
ENH73642.1|Fusarium oxysporum
FWZ79682.1|Fusarium oxysporum
EMT72703.1|Fusarium oxysporum
EXM18473.1|Fusarium oxysporum
WP 020514078.1|Actinoplanes globisporus
WP 030143769.1|Micrococcineae
WP 033212543.1|Streptomyces wedmorensis
WP 026565621.1|Bacillus sp.
WP 023986845.1|Paenibacillus polymyxa
WP 028541113.1|Paenibacillus sp.
WP 014267625.1|Granulicella mallensis
WP 029084122.1|Bradyrhizobium sp.
CDG85324.1|Janthinobacterium agaricidamnosum
WP 007816325.1|Rhizobium sp.
WP 025670193.1|Rhizobium sp.
WP 032938226.1|Mesorhizobium sp.
WP 018517759.1|Rhizobium leguminosarum
KFC79329.1|Ewingella americana
WP 008973322.1|Bradyrhizobium sp.
AHY51613.1|Bradyrhizobium japonicum
WP 014496339.1|Bradyrhizobium japonicum
WP 011083103.1|Bradyrhizobium
WP 007596044.1|Bradyrhizobium sp.
red: fungi
blue: bacteria
found in small number of aspergillus
transfer from distant fungi

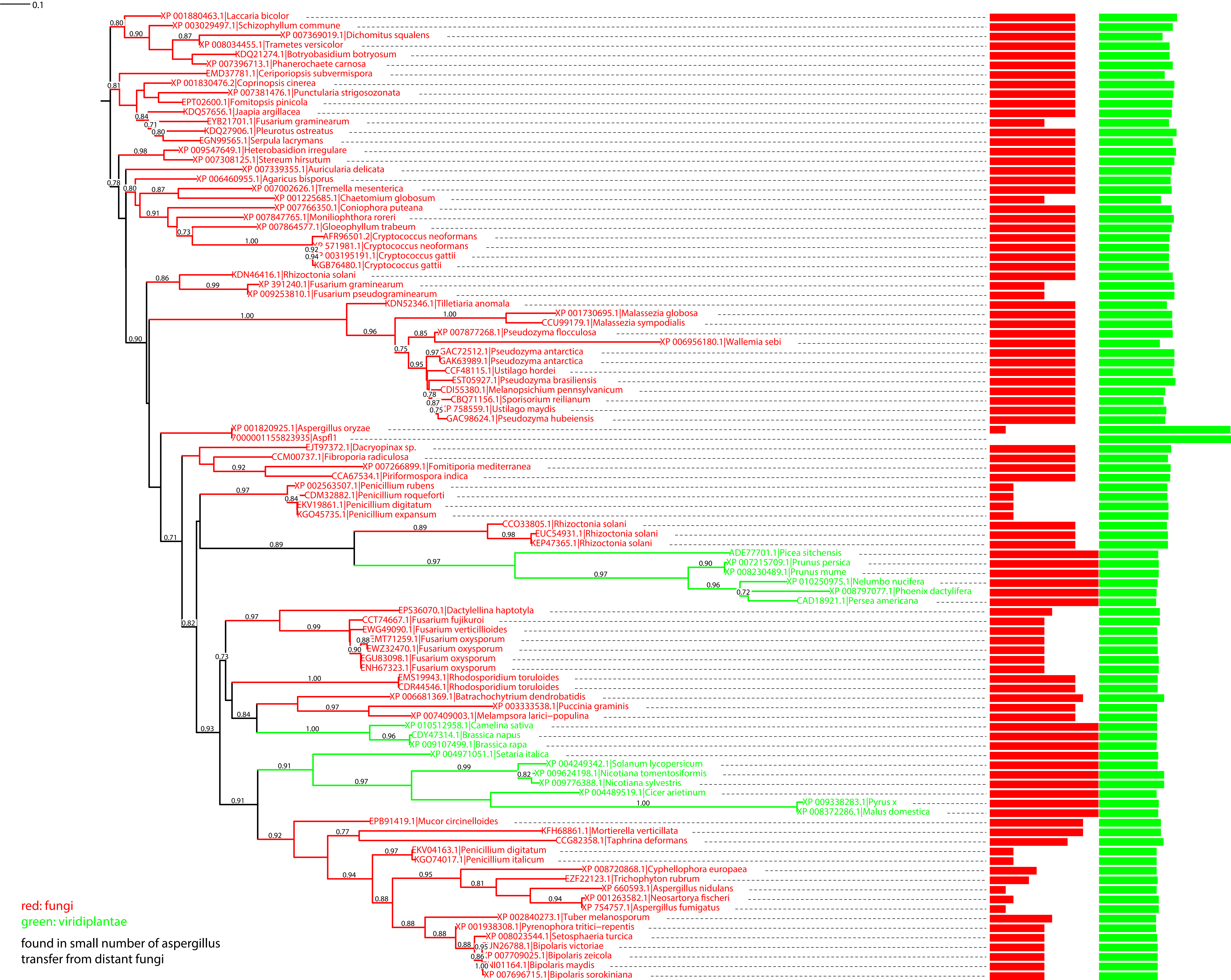

0.1
XP 001880463.1|Laccaria bicolor
XP 003029497.1|Schizophyllum commune
XP 007369019.1|Dichomitus squalens
XP 008034455.1|Trametes versicolor
KDQ21274.1|Botryobasidium botryosum
XP 007396713.1|Phanerochaete carnosa
EMD37781.1|Ceriporiopsis subvermispora
XP 001830476.2|Coprinopsis cinerea
XP 007381476.1|Punctularia strigosozonata
EPT02600.1|Fomitopsis pinicola
KDQ57656.1|Jaapia argillacea
EYB21701.1|Fusarium graminearum
KDQ27906.1|Pleurotus ostreatus
EGN99565.1|Serpula lacrymans
XP 009547649.1|Heterobasidion irregulare
XP 007308125.1|Stereum hirsutum
XP 007339355.1|Auricularia delicata
XP 006460955.1|Agaricus bisporus
XP 007002626.1|Tremella mesenterica
XP 001225685.1|Chaetomium globosum
XP 007766350.1|Coniophora puteana
XP 007847765.1|Moniliophthora roreri
XP 007864577.1|Gloeophyllum trabeum
AFR96501.2|Cryptococcus neoformans
571981.1|Cryptococcus neoformans
003195191.1|Cryptococcus gattii
KGB76480.1|Cryptococcus gattii
KDN46416.1|Rhizoctonia solani
XP 391240.1|Fusarium graminearum
XP 009253810.1|Fusarium pseudograminearum
KDN52346.1|Tilletiaria anomala
XP 001730695.1|Malassezia globosa
CCU99179.1|Malassezia sympodialis
XP 007877268.1|Pseudozyma flocculosa
XP 006956180.1|Wallemia sebi
GAC72512.1|Pseudozyma antarctica
GAK63989.1|Pseudozyma antarctica
CCF48115.1|Ustilago hordei
EST05927.1|Pseudozyma brasiliensis
CDI55380.1|Melanopsichium pennsylvanicum
CBQ71156.1|Sporisorium reilianum
P 758559.1|Ustilago maydis
GAC98624.1|Pseudozyma hubeiensis
XP 001820925.1|Aspergillus oryzae
7000001155823935|Aspfl1
EJT97372.1|Dacryopinax sp.
CCM00737.1|Fibroporia radiculosa
XP 007266899.1|Fomitiporia mediterranea
CCA67534.1|Piriformospora indica
XP 002563507.1|Penicillium rubens
CDM32882.1|Penicillium roqueforti
EKV19861.1|Penicillium digitatum
KGO45735.1|Penicillium expansum
CCO33805.1|Rhizoctonia solani
EUC54931.1|Rhizoctonia solani
KEP47365.1|Rhizoctonia solani
ADE77701.1|Picea sitchensis
XP 007215709.1|Prunus persica
XP 008230489.1|Prunus mume
XP 010250975.1|Nelumbo nucifera
XP 008797077.1|Phoenix dactylifera
CAD18921.1|Persea americana
EPS36070.1|Dactylellina haptotyla
CCT74667.1|Fusarium fujikuroi
EWG49090.1|Fusarium verticillioides
EMT71259.1|Fusarium oxysporum
EWZ32470.1|Fusarium oxysporum
EGU83098.1|Fusarium oxysporum
ENH67323.1|Fusarium oxysporum
EMS19943.1|Rhodosporidium toruloides
CDR44546.1|Rhodosporidium toruloides
XP 006681369.1|Batrachochytrium dendrobatidis
XP 003333538.1|Puccinia graminis
XP 007409003.1|Melampsora larici–populina
XP 010512958.1|Camelina sativa
CDY47314.1|Brassica napus
XP 009107499.1|Brassica rapa
XP 004971051.1|Setaria italica
XP 004249342.1|Solanum lycopersicum
XP 009624198.1|Nicotiana tomentosiformis
XP 009776388.1|Nicotiana sylvestris
XP 004489519.1|Cicer arietinum
XP 009338283.1|Pyrus x
XP 008372286.1|Malus domestica
EPB91419.1|Mucor circinelloides
KFH68861.1|Mortierella verticillata
CCG82358.1|Taphrina deformans
EKV04163.1|Penicillium digitatum
KGO74017.1|Penicillium italicum
XP 008720868.1|Cyphellophora europaea
EZF22123.1|Trichophyton rubrum
XP 660593.1|Aspergillus nidulans
XP 001263582.1|Neosartorya fischeri
XP 754757.1|Aspergillus fumigatus
XP 002840273.1|Tuber melanosporum
XP 001938308.1|Pyrenophora tritici–repentis
XP 008023544.1|Setosphaeria turcica
JN26788.1|Bipolaris victoriae
XP 007709025.1|Bipolaris zeicola
NI01164.1|Bipolaris maydis
XP 007696715.1|Bipolaris sorokiniana
red: fungi
green: viridiplantae
found in small number of aspergillus
transfer from distant fungi

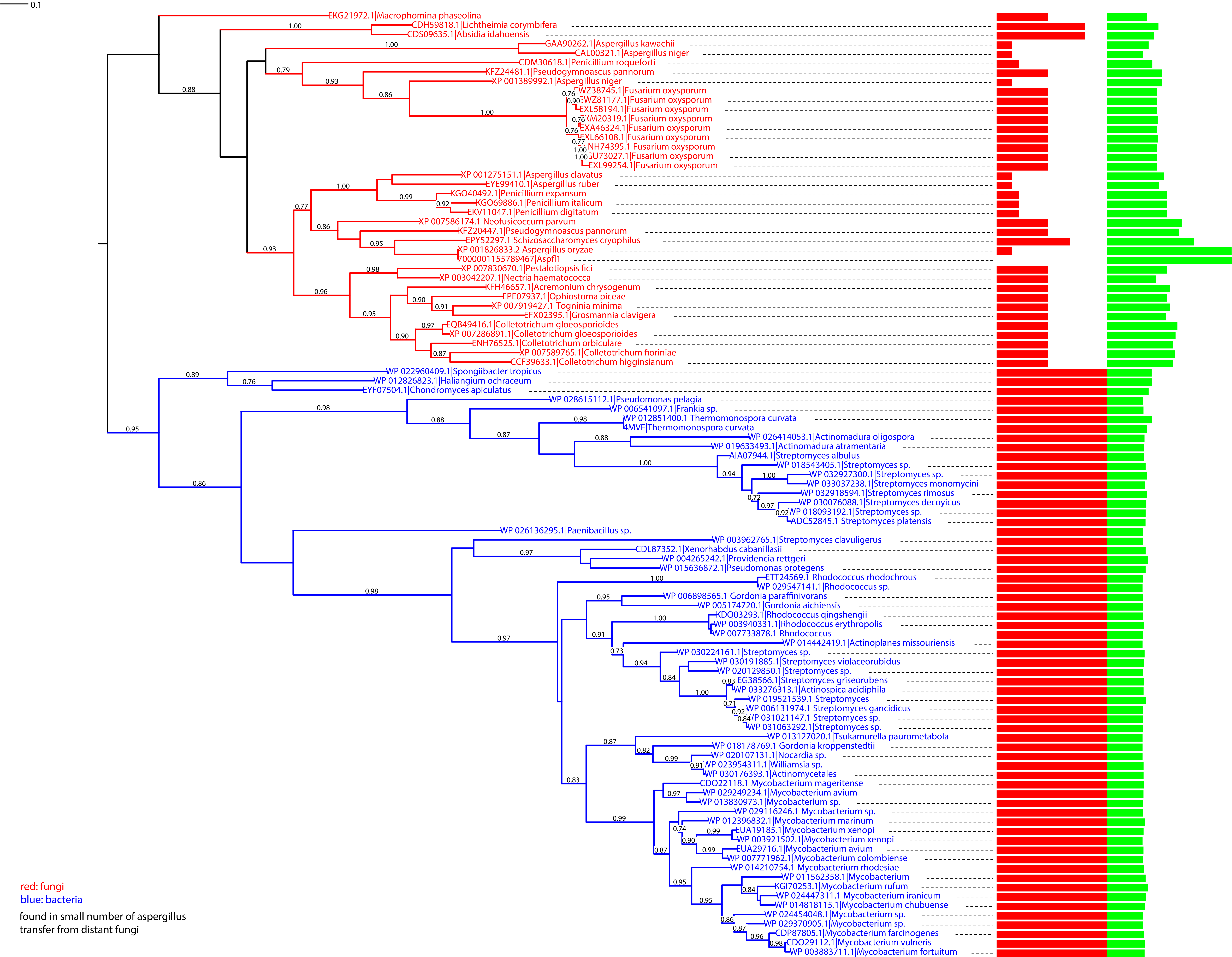
0.1
EKG21972.1|Macrophomina phaseolina
CDH59818.1|Lichtheimia corymbifera
CDS09635.1|Absidia idahoensis
GAA90262.1|Aspergillus kawachii
CAL00321.1|Aspergillus niger
CDM30618.1|Penicillium roqueforti
KFZ24481.1|Pseudogymnoascus pannorum
XP 001389992.1|Aspergillus niger
EWZ38745.1|Fusarium oxysporum
WZ81177.1|Fusarium oxysporum
EXL58194.1|Fusarium oxysporum
KM20319.1|Fusarium oxysporum
EXA46324.1|Fusarium oxysporum
EXL66108.1|Fusarium oxysporum
NH74395.1|Fusarium oxysporum
GU73027.1|Fusarium oxysporum
EXL99254.1|Fusarium oxysporum
XP 001275151.1|Aspergillus clavatus
EYE99410.1|Aspergillus ruber
KGO40492.1|Penicillium expansum
KGO69886.1|Penicillium italicum
EKV11047.1|Penicillium digitatum
XP 007586174.1|Neofusicoccum parvum
KFZ20447.1|Pseudogymnoascus pannorum
EPY52297.1|Schizosaccharomyces cryophilus
XP 001826833.2|Aspergillus oryzae
7000001155789467|Aspfl1
XP 007830670.1|Pestalotiopsis fici
XP 003042207.1|Nectria haematococca
KFH46657.1|Acremonium chrysogenum
EPE07937.1|Ophiostoma piceae
XP 007919427.1|Togninia minima
EFX02395.1|Grosmannia clavigera
EQB49416.1|Colletotrichum gloeosporioides
XP 007286891.1|Colletotrichum gloeosporioides
ENH76525.1|Colletotrichum orbiculare
XP 007589765.1|Colletotrichum fioriniae
CCF39633.1|Colletotrichum higginsianum
WP 022960409.1|Spongiibacter tropicus
WP 012826823.1|Haliangium ochraceum
EYF07504.1|Chondromyces apiculatus
WP 028615112.1|Pseudomonas pelagia
WP 006541097.1|Frankia sp.
WP 012851400.1|Thermomonospora curvata
4MVE|Thermomonospora curvata
WP 026414053.1|Actinomadura oligospora
WP 019633493.1|Actinomadura atramentaria
AIA07944.1|Streptomyces albulus
WP 018543405.1|Streptomyces sp.
WP 032927300.1|Streptomyces sp.
WP 033037238.1|Streptomyces monomycini
WP 032918594.1|Streptomyces rimosus
WP 030076088.1|Streptomyces decoyicus
WP 018093192.1|Streptomyces sp.
ADC52845.1|Streptomyces platensis
WP 026136295.1|Paenibacillus sp.
WP 003962765.1|Streptomyces clavuligerus
CDL87352.1|Xenorhabdus cabanillasii
WP 004265242.1|Providencia rettgeri
WP 015636872.1|Pseudomonas protegens
ETT24569.1|Rhodococcus rhodochrous
WP 029547141.1|Rhodococcus sp.
WP 006898565.1|Gordonia paraffinivorans
WP 005174720.1|Gordonia aichiensis
KDQ03293.1|Rhodococcus qingshengii
WP 003940331.1|Rhodococcus erythropolis
WP 007733878.1|Rhodococcus
WP 014442419.1|Actinoplanes missouriensis
WP 030224161.1|Streptomyces sp.
WP 030191885.1|Streptomyces violaceorubidus
WP 020129850.1|Streptomyces sp.
EG38566.1|Streptomyces griseorubens
WP 033276313.1|Actinospica acidiphila
WP 019521539.1|Streptomyces
WP 006131974.1|Streptomyces gancidicus
P 031021147.1|Streptomyces sp.
WP 031063292.1|Streptomyces sp.
WP 013127020.1|Tsukamurella paurometabola
WP 018178769.1|Gordonia kroppenstedtii
WP 020107131.1|Nocardia sp.
WP 023954311.1|Williamsia sp.
WP 030176393.1|Actinomycetales
CDO22118.1|Mycobacterium mageritense
WP 029249234.1|Mycobacterium avium
WP 013830973.1|Mycobacterium sp.
WP 029116246.1|Mycobacterium sp.
WP 012396832.1|Mycobacterium marinum
EUA19185.1|Mycobacterium xenopi
WP 003921502.1|Mycobacterium xenopi
EUA29716.1|Mycobacterium avium
WP 007771962.1|Mycobacterium colombiense
WP 014210754.1|Mycobacterium rhodesiae
WP 011562358.1|Mycobacterium
KGI70253.1|Mycobacterium rufum
WP 024447311.1|Mycobacterium iranicum
WP 014818115.1|Mycobacterium chubuense
WP 024454048.1|Mycobacterium sp.
WP 029370905.1|Mycobacterium sp.
CDP87805.1|Mycobacterium farcinogenes
CDO29112.1|Mycobacterium vulneris
WP 003883711.1|Mycobacterium fortuitum
red: fungi
blue: bacteria
found in small number of aspergillus
transfer from distant fungi

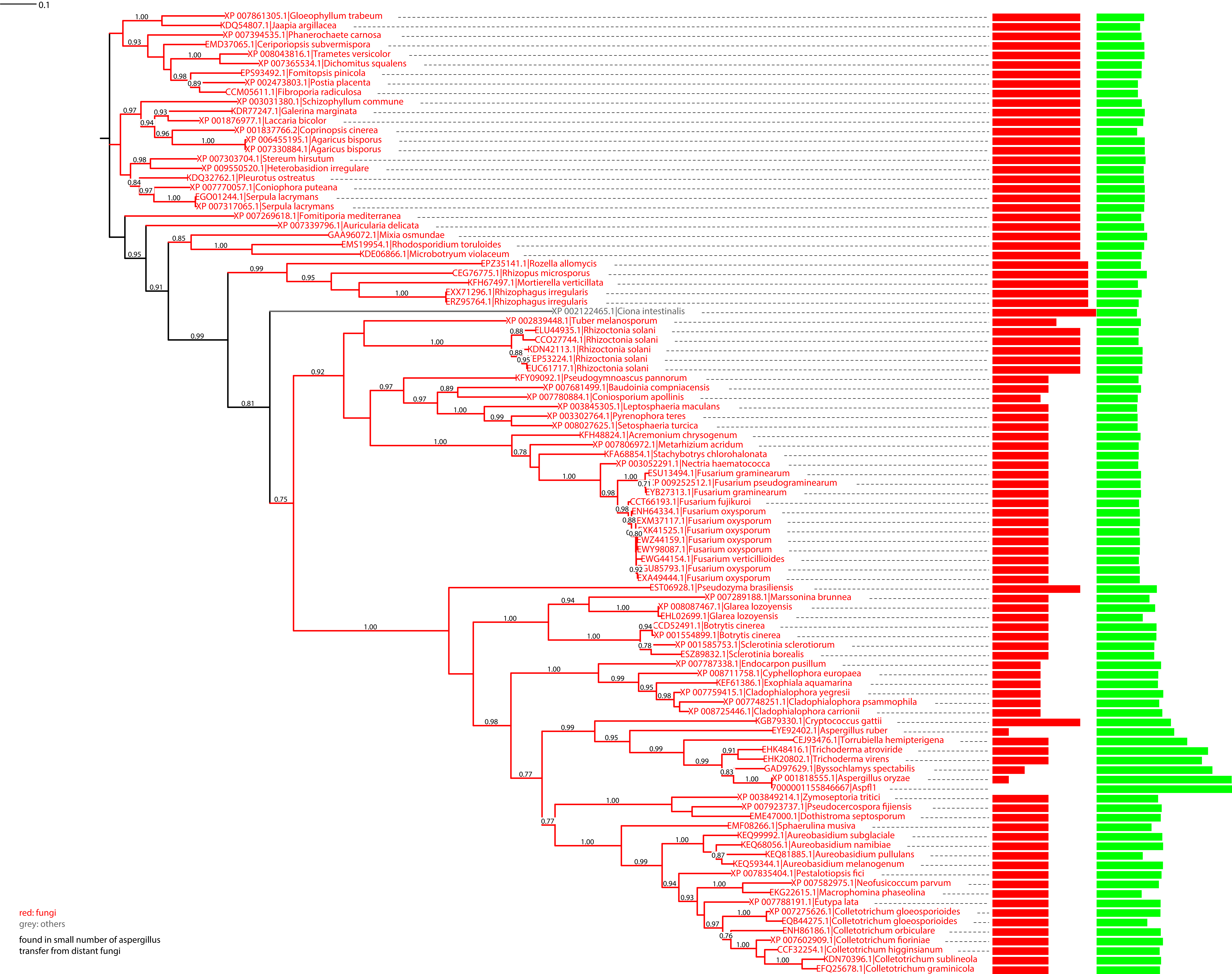

0.1
XP 007861305.1|Gloeophyllum trabeum
KDQ54807.1|Jaapia argillacea
XP 007394535.1|Phanerochaete carnosa
EMD37065.1|Ceriporiopsis subvermispora
XP 008043816.1|Trametes versicolor
XP 007365534.1|Dichomitus squalens
EPS93492.1|Fomitopsis pinicola
XP 002473803.1|Postia placenta
CCM05611.1|Fibroporia radiculosa
XP 003031380.1|Schizophyllum commune
KDR77247.1|Galerina marginata
XP 001876977.1|Laccaria bicolor
XP 001837766.2|Coprinopsis cinerea
XP 006455195.1|Agaricus bisporus
XP 007330884.1|Agaricus bisporus
XP 007303704.1|Stereum hirsutum
XP 009550520.1|Heterobasidion irregulare
KDQ32762.1|Pleurotus ostreatus
XP 007770057.1|Coniophora puteana
EGO01244.1|Serpula lacrymans
XP 007317065.1|Serpula lacrymans
XP 007269618.1|Fomitiporia mediterranea
XP 007339796.1|Auricularia delicata
GAA96072.1|Mixia osmundae
EMS19954.1|Rhodosporidium toruloides
KDE06866.1|Microbotryum violaceum
EPZ35141.1|Rozella allomycis
CEG76775.1|Rhizopus microsporus
KFH67497.1|Mortierella verticillata
EXX71296.1|Rhizophagus irregularis
ERZ95764.1|Rhizophagus irregularis
XP 002122465.1|Ciona intestinalis
XP 002839448.1|Tuber melanosporum
ELU44935.1|Rhizoctonia solani
CCO27744.1|Rhizoctonia solani
KDN42113.1|Rhizoctonia solani
EP53224.1|Rhizoctonia solani
EUC61717.1|Rhizoctonia solani
KFY09092.1|Pseudogymnoascus pannorum
XP 007681499.1|Baudoinia compniacensis
XP 007780884.1|Coniosporium apollinis
XP 003845305.1|Leptosphaeria maculans
XP 003302764.1|Pyrenophora teres
XP 008027625.1|Setosphaeria turcica
KFH48824.1|Acremonium chrysogenum
XP 007806972.1|Metarhizium acridum
KFA68854.1|Stachybotrys chlorohalonata
XP 003052291.1|Nectria haematococca
ESU13494.1|Fusarium graminearum
P 009252512.1|Fusarium pseudograminearum
EYB27313.1|Fusarium graminearum
CCT66193.1|Fusarium fujikuroi
ENH64334.1|Fusarium oxysporum
EXM37117.1|Fusarium oxysporum
XK41525.1|Fusarium oxysporum
EWZ44159.1|Fusarium oxysporum
EWY98087.1|Fusarium oxysporum
EWG44154.1|Fusarium verticillioides
GU85793.1|Fusarium oxysporum
EXA49444.1|Fusarium oxysporum
EST06928.1|Pseudozyma brasiliensis
XP 007289188.1|Marssonina brunnea
XP 008087467.1|Glarea lozoyensis
EHL02699.1|Glarea lozoyensis
CCD52491.1|Botrytis cinerea
XP 001554899.1|Botrytis cinerea
XP 001585753.1|Sclerotinia sclerotiorum
ESZ89832.1|Sclerotinia borealis
XP 007787338.1|Endocarpon pusillum
XP 008711758.1|Cyphellophora europaea
KEF61386.1|Exophiala aquamarina
XP 007759415.1|Cladophialophora yegresii
XP 007748251.1|Cladophialophora psammophila
XP 008725446.1|Cladophialophora carrionii
KGB79330.1|Cryptococcus gattii
EYE92402.1|Aspergillus ruber
CEJ93476.1|Torrubiella hemipterigena
EHK48416.1|Trichoderma atroviride
EHK20802.1|Trichoderma virens
GAD97629.1|Byssochlamys spectabilis
XP 001818555.1|Aspergillus oryzae
7000001155846667|Aspfl1
XP 003849214.1|Zymoseptoria tritici
XP 007923737.1|Pseudocercospora fijiensis
EME47000.1|Dothistroma septosporum
EMF08266.1|Sphaerulina musiva
KEQ99992.1|Aureobasidium subglaciale
KEQ68056.1|Aureobasidium namibiae
KEQ81885.1|Aureobasidium pullulans
KEQ59344.1|Aureobasidium melanogenum
XP 007835404.1|Pestalotiopsis fici
XP 007582975.1|Neofusicoccum parvum
EKG22615.1|Macrophomina phaseolina
XP 007788191.1|Eutypa lata
XP 007275626.1|Colletotrichum gloeosporioides
EQB44275.1|Colletotrichum gloeosporioides
ENH86186.1|Colletotrichum orbiculare
XP 007602909.1|Colletotrichum fioriniae
CCF32254.1|Colletotrichum higginsianum
KDN70396.1|Colletotrichum sublineola
EFQ25678.1|Colletotrichum graminicola
red: fungi
grey: others
found in small number of aspergillus
transfer from distant fungi

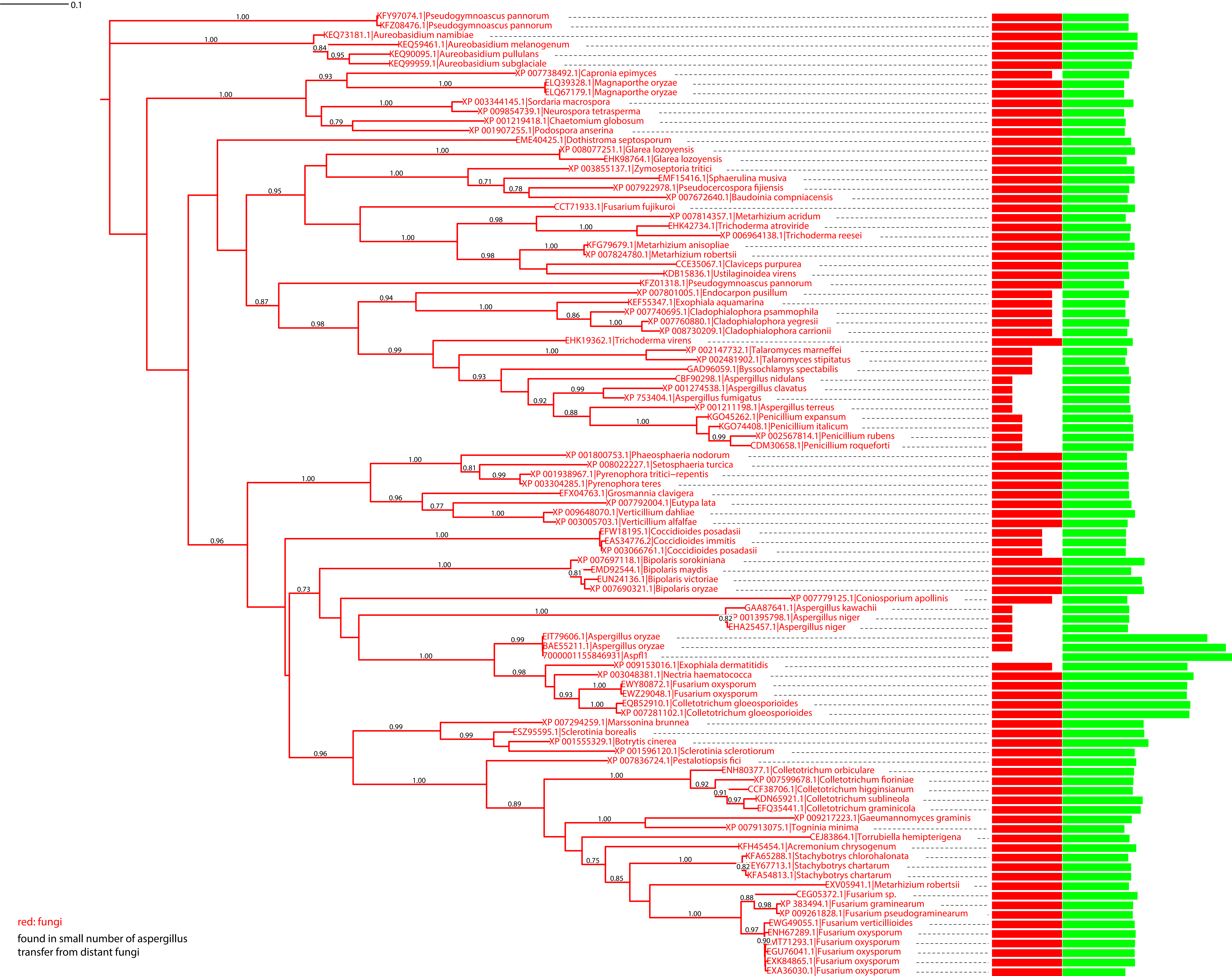
0.1
KFY97074.1|Pseudogymnoascus pannorum
KFZ08476.1|Pseudogymnoascus pannorum
KEQ73181.1|Aureobasidium namibiae
KEQ59461.1|Aureobasidium melanogenum
KEQ90095.1|Aureobasidium pullulans
KEQ99959.1|Aureobasidium subglaciale
XP 007738492.1|Capronia epimyces
ELQ39328.1|Magnaporthe oryzae
ELQ67179.1|Magnaporthe oryzae
XP 003344145.1|Sordaria macrospora
XP 009854739.1|Neurospora tetrasperma
XP 001219418.1|Chaetomium globosum
XP 001907255.1|Podospora anserina
EME40425.1|Dothistroma septosporum
XP 008077251.1|Glarea lozoyensis
EHK98764.1|Glarea lozoyensis
XP 003855137.1|Zymoseptoria tritici
EMF15416.1|Sphaerulina musiva
XP 007922978.1|Pseudocercospora fijiensis
XP 007672640.1|Baudoinia compniacensis
CCT71933.1|Fusarium fujikuroi
XP 007814357.1|Metarhizium acridum
EHK42734.1|Trichoderma atroviride
XP 006964138.1|Trichoderma reesei
KFG79679.1|Metarhizium anisopliae
XP 007824780.1|Metarhizium robertsii
CCE35067.1|Claviceps purpurea
KDB15836.1|Ustilaginoidea virens
KFZ01318.1|Pseudogymnoascus pannorum
XP 007801005.1|Endocarpon pusillum
KEF55347.1|Exophiala aquamarina
XP 007740695.1|Cladophialophora psammophila
XP 007760880.1|Cladophialophora yegresii
XP 008730209.1|Cladophialophora carrionii
EHK19362.1|Trichoderma virens
XP 002147732.1|Talaromyces marneffei
XP 002481902.1|Talaromyces stipitatus
GAD96059.1|Byssochlamys spectabilis
CBF90298.1|Aspergillus nidulans
XP 001274538.1|Aspergillus clavatus
XP 753404.1|Aspergillus fumigatus
XP 001211198.1|Aspergillus terreus
KGO45262.1|Penicillium expansum
KGO74408.1|Penicillium italicum
XP 002567814.1|Penicillium rubens
CDM30658.1|Penicillium roqueforti
XP 001800753.1|Phaeosphaeria nodorum
XP 008022227.1|Setosphaeria turcica
XP 001938967.1|Pyrenophora tritici−repentis
XP 003304285.1|Pyrenophora teres
EFX04763.1|Grosmannia clavigera
XP 007792004.1|Eutypa lata
XP 009648070.1|Verticillium dahliae
XP 003005703.1|Verticillium alfalfae
EFW18195.1|Coccidioides posadasii
EAS34776.2|Coccidioides immitis
XP 003066761.1|Coccidioides posadasii
XP 007697118.1|Bipolaris sorokiniana
EMD92544.1|Bipolaris maydis
EUN24136.1|Bipolaris victoriae
XP 007690321.1|Bipolaris oryzae
XP 007779125.1|Coniosporium apollinis
GAA87641.1|Aspergillus kawachii
P 001395798.1|Aspergillus niger
EHA25457.1|Aspergillus niger
EIT79606.1|Aspergillus oryzae
BAE55211.1|Aspergillus oryzae
7000001155846931|Aspfl1
XP 009153016.1|Exophiala dermatitidis
XP 003048381.1|Nectria haematococca
EWY80872.1|Fusarium oxysporum
EWZ29048.1|Fusarium oxysporum
EQB52910.1|Colletotrichum gloeosporioides
XP 007281102.1|Colletotrichum gloeosporioides
XP 007294259.1|Marssonina brunnea
ESZ95595.1|Sclerotinia borealis
XP 001555329.1|Botrytis cinerea
XP 001596120.1|Sclerotinia sclerotiorum
XP 007836724.1|Pestalotiopsis fici
ENH80377.1|Colletotrichum orbiculare
XP 007599678.1|Colletotrichum fioriniae
CCF38706.1|Colletotrichum higginsianum
KDN65921.1|Colletotrichum sublineola
EFQ35441.1|Colletotrichum graminicola
XP 009217223.1|Gaeumannomyces graminis
XP 007913075.1|Togninia minima
CEJ83864.1|Torrubiella hemipterigena
KFH45454.1|Acremonium chrysogenum
KFA65288.1|Stachybotrys chlorohalonata
EY67713.1|Stachybotrys chartarum
KFA54813.1|Stachybotrys chartarum
EXV05941.1|Metarhizium robertsii
CEG05372.1|Fusarium sp.
XP 383494.1|Fusarium graminearum
XP 009261828.1|Fusarium pseudograminearum
EWG49055.1|Fusarium verticillioides
ENH67289.1|Fusarium oxysporum
MT71293.1|Fusarium oxysporum
EGU76041.1|Fusarium oxysporum
EXK84865.1|Fusarium oxysporum
EXA36030.1|Fusarium oxysporum
red: fungi
found in small number of aspergillus
transfer from distant fungi

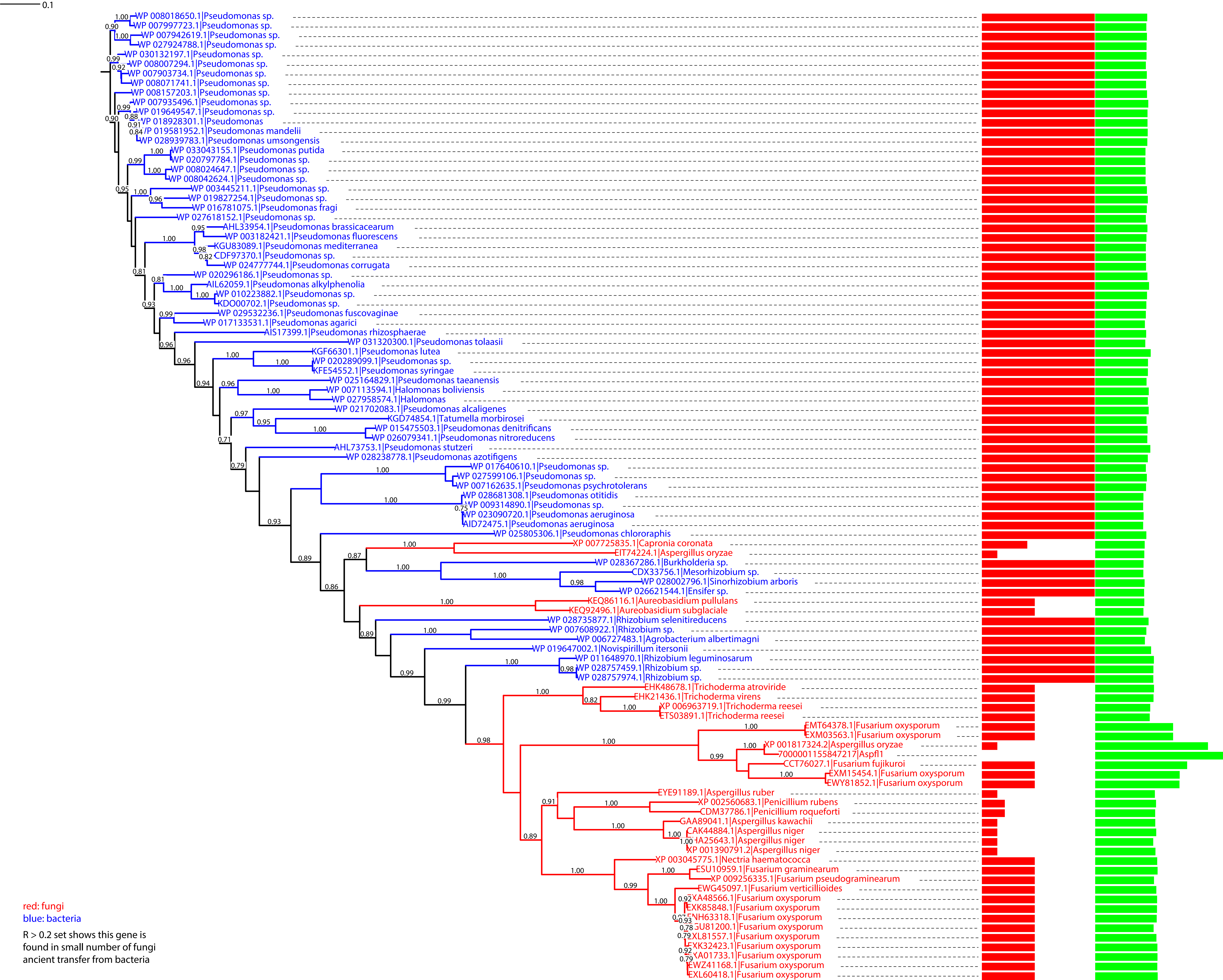

0.1
WP 008018650.1|Pseudomonas sp.
WP 007997723.1|Pseudomonas sp.
WP 007942619.1|Pseudomonas sp.
WP 027924788.1|Pseudomonas sp.
WP 030132197.1|Pseudomonas sp.
WP 008007294.1|Pseudomonas sp.
WP 007903734.1|Pseudomonas sp.
WP 008071741.1|Pseudomonas sp.
WP 008157203.1|Pseudomonas sp.
WP 007935496.1|Pseudomonas sp.
WP 019649547.1|Pseudomonas sp.
WP 018928301.1|Pseudomonas
WP 019581952.1|Pseudomonas mandelii
WP 028939783.1|Pseudomonas umsongensis
WP 033043155.1|Pseudomonas putida
WP 020797784.1|Pseudomonas sp.
WP 008024647.1|Pseudomonas sp.
WP 008042624.1|Pseudomonas sp.
WP 003445211.1|Pseudomonas sp.
WP 019827254.1|Pseudomonas sp.
WP 016781075.1|Pseudomonas fragi
WP 027618152.1|Pseudomonas sp.
AHL33954.1|Pseudomonas brassicacearum
WP 003182421.1|Pseudomonas fluorescens
KGU83089.1|Pseudomonas mediterranea
CDF97370.1|Pseudomonas sp.
WP 024777744.1|Pseudomonas corrugata
WP 020296186.1|Pseudomonas sp.
AIL62059.1|Pseudomonas alkylphenolia
WP 010223882.1|Pseudomonas sp.
KDO00702.1|Pseudomonas sp.
WP 029532236.1|Pseudomonas fuscovaginae
WP 017133531.1|Pseudomonas agarici
AIS17399.1|Pseudomonas rhizosphaerae
WP 031320300.1|Pseudomonas tolaasii
KGF66301.1|Pseudomonas lutea
WP 020289099.1|Pseudomonas sp.
KFE54552.1|Pseudomonas syringae
WP 025164829.1|Pseudomonas taeanensis
WP 007113594.1|Halomonas boliviensis
WP 027958574.1|Halomonas
WP 021702083.1|Pseudomonas alcaligenes
KGD74854.1|Tatumella morbirosei
WP 015475503.1|Pseudomonas denitrificans
WP 026079341.1|Pseudomonas nitroreducens
AHL73753.1|Pseudomonas stutzeri
WP 028238778.1|Pseudomonas azotifigens
WP 017640610.1|Pseudomonas sp.
WP 027599106.1|Pseudomonas sp.
WP 007162635.1|Pseudomonas psychrotolerans
WP 028681308.1|Pseudomonas otitidis
WP 009314890.1|Pseudomonas sp.
WP 023090720.1|Pseudomonas aeruginosa
AID72475.1|Pseudomonas aeruginosa
WP 025805306.1|Pseudomonas chlororaphis
XP 007725835.1|Capronia coronata
EIT74224.1|Aspergillus oryzae
WP 028367286.1|Burkholderia sp.
CDX33756.1|Mesorhizobium sp.
WP 028002796.1|Sinorhizobium arboris
WP 026621544.1|Ensifer sp.
KEQ86116.1|Aureobasidium pullulans
KEQ92496.1|Aureobasidium subglaciale
WP 028735877.1|Rhizobium selenitireducens
WP 007608922.1|Rhizobium sp.
WP 006727483.1|Agrobacterium albertimagni
WP 019647002.1|Novispirillum itersonii
WP 011648970.1|Rhizobium leguminosarum
WP 028757459.1|Rhizobium sp.
WP 028757974.1|Rhizobium sp.
EHK48678.1|Trichoderma atroviride
EHK21436.1|Trichoderma virens
XP 006963719.1|Trichoderma reesei
ETS03891.1|Trichoderma reesei
EMT64378.1|Fusarium oxysporum
EXM03563.1|Fusarium oxysporum
XP 001817324.2|Aspergillus oryzae
7000001155847217|Aspfl1
CCT76027.1|Fusarium fujikuroi
EXM15454.1|Fusarium oxysporum
EWY81852.1|Fusarium oxysporum
EYE91189.1|Aspergillus ruber
XP 002560683.1|Penicillium rubens
CDM37786.1|Penicillium roqueforti
GAA89041.1|Aspergillus kawachii
CAK44884.1|Aspergillus niger
EHA25643.1|Aspergillus niger
XP 001390791.2|Aspergillus niger
XP 003045775.1|Nectria haematococca
ESU10959.1|Fusarium graminearum
XP 009256335.1|Fusarium pseudograminearum
EWG45097.1|Fusarium verticillioides
EXA48566.1|Fusarium oxysporum
EXK85848.1|Fusarium oxysporum
ENH63318.1|Fusarium oxysporum
EGU81200.1|Fusarium oxysporum
EXL81557.1|Fusarium oxysporum
EXK32423.1|Fusarium oxysporum
EXA01733.1|Fusarium oxysporum
EWZ41168.1|Fusarium oxysporum
EXL60418.1|Fusarium oxysporum
red: fungi
blue: bacteria
R > 0.2 set shows this gene is
found in small number of fungi
ancient transfer from bacteria

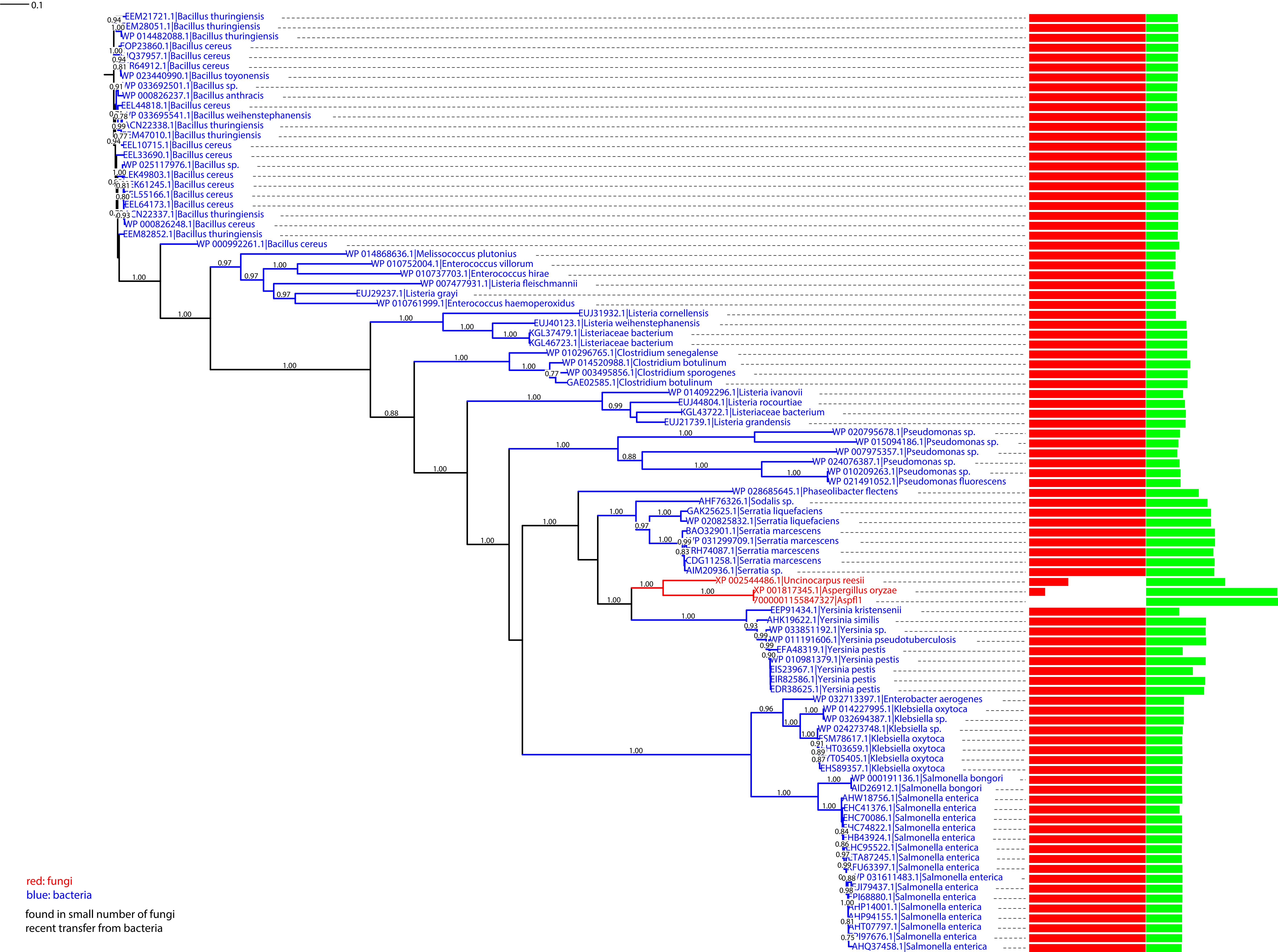
0.1
EEM21721.1|Bacillus thuringiensis
EM28051.1|Bacillus thuringiensis
WP 014482088.1|Bacillus thuringiensis
EOP23860.1|Bacillus cereus
IQ37957.1|Bacillus cereus
R64912.1|Bacillus cereus
WP 023440990.1|Bacillus toyonensis
WP 033692501.1|Bacillus sp.
WP 000826237.1|Bacillus anthracis
EEL44818.1|Bacillus cereus
P 033695541.1|Bacillus weihenstephanensis
CN22338.1|Bacillus thuringiensis
M47010.1|Bacillus thuringiensis
EEL10715.1|Bacillus cereus
EEL33690.1|Bacillus cereus
WP 025117976.1|Bacillus sp.
EK49803.1|Bacillus cereus
K61245.1|Bacillus cereus
L55166.1|Bacillus cereus
EEL64173.1|Bacillus cereus
N22337.1|Bacillus thuringiensis
WP 000826248.1|Bacillus cereus
EEM82852.1|Bacillus thuringiensis
WP 000992261.1|Bacillus cereus
WP 014868636.1|Melissococcus plutonius
WP 010752004.1|Enterococcus villorum
WP 010737703.1|Enterococcus hirae
WP 007477931.1|Listeria fleischmannii
EUJ29237.1|Listeria grayi
WP 010761999.1|Enterococcus haemoperoxidus
EUJ31932.1|Listeria cornellensis
EUJ40123.1|Listeria weihenstephanensis
KGL37479.1|Listeriaceae bacterium
KGL46723.1|Listeriaceae bacterium
WP 010296765.1|Clostridium senegalense
WP 014520988.1|Clostridium botulinum
WP 003495856.1|Clostridium sporogenes
GAE02585.1|Clostridium botulinum
WP 014092296.1|Listeria ivanovii
EUJ44804.1|Listeria rocourtiae
KGL43722.1|Listeriaceae bacterium
EUJ21739.1|Listeria grandensis
WP 020795678.1|Pseudomonas sp.
WP 015094186.1|Pseudomonas sp.
WP 007975357.1|Pseudomonas sp.
WP 024076387.1|Pseudomonas sp.
WP 010209263.1|Pseudomonas sp.
WP 021491052.1|Pseudomonas fluorescens
WP 028685645.1|Phaseolibacter flectens
AHF76326.1|Sodalis sp.
GAK25625.1|Serratia liquefaciens
WP 020825832.1|Serratia liquefaciens
BAO32901.1|Serratia marcescens
P 031299709.1|Serratia marcescens
RH74087.1|Serratia marcescens
CDG11258.1|Serratia marcescens
AIM20936.1|Serratia sp.
XP 002544486.1|Uncinocarpus reesii
XP 001817345.1|Aspergillus oryzae
7000001155847327|Aspfl1
EEP91434.1|Yersinia kristensenii
AHK19622.1|Yersinia similis
WP 033851192.1|Yersinia sp.
WP 011191606.1|Yersinia pseudotuberculosis
EFA48319.1|Yersinia pestis
WP 010981379.1|Yersinia pestis
EIS23967.1|Yersinia pestis
EIR82586.1|Yersinia pestis
EDR38625.1|Yersinia pestis
WP 032713397.1|Enterobacter aerogenes
WP 014227995.1|Klebsiella oxytoca
WP 032694387.1|Klebsiella sp.
WP 024273748.1|Klebsiella sp.
SM78617.1|Klebsiella oxytoca
HT03659.1|Klebsiella oxytoca
T05405.1|Klebsiella oxytoca
EHS89357.1|Klebsiella oxytoca
WP 000191136.1|Salmonella bongori
AID26912.1|Salmonella bongori
AHW18756.1|Salmonella enterica
EHC41376.1|Salmonella enterica
EHC70086.1|Salmonella enterica
HC74822.1|Salmonella enterica
HB43924.1|Salmonella enterica
HC95522.1|Salmonella enterica
TA87245.1|Salmonella enterica
FU63397.1|Salmonella enterica
P 031611483.1|Salmonella enterica
JI79437.1|Salmonella enterica
PI68880.1|Salmonella enterica
HP14001.1|Salmonella enterica
HP94155.1|Salmonella enterica
HT07797.1|Salmonella enterica
I97676.1|Salmonella enterica
AHQ37458.1|Salmonella enterica
red: fungi
blue: bacteria
found in small number of fungi
recent transfer from bacteria

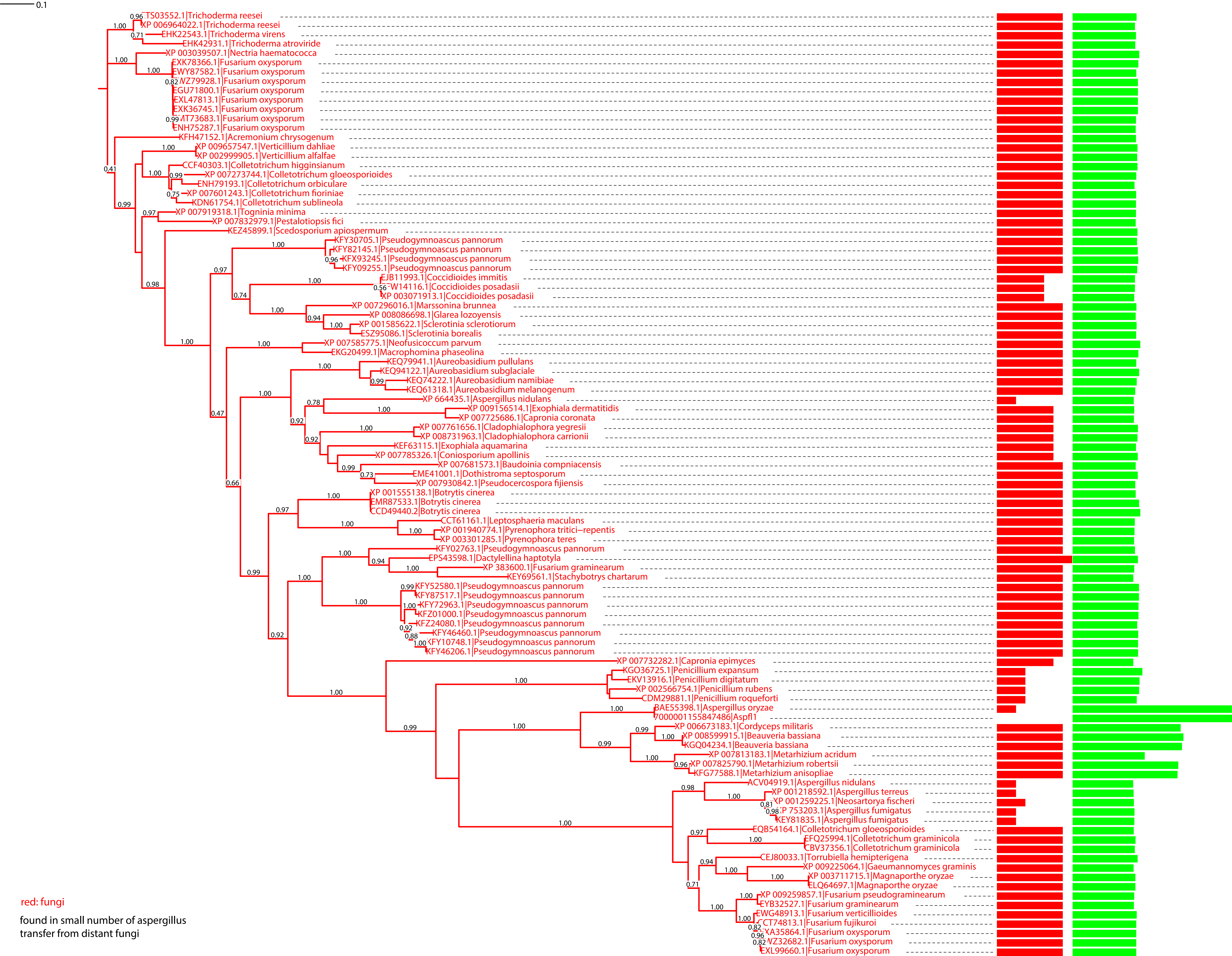
0.1
TS03552.1|Trichoderma reesei
XP 006964022.1|Trichoderma reesei
EHK22543.1|Trichoderma virens
EHK42931.1|Trichoderma atroviride
XP 003039507.1|Nectria haematococca
EXK78366.1|Fusarium oxysporum
EWY87582.1|Fusarium oxysporum
WZ79928.1|Fusarium oxysporum
EGU71800.1|Fusarium oxysporum
EXL47813.1|Fusarium oxysporum
EXK36745.1|Fusarium oxysporum
MT73683.1|Fusarium oxysporum
ENH75287.1|Fusarium oxysporum
KFH47152.1|Acremonium chrysogenum
XP 009657547.1|Verticillium dahliae
XP 002999905.1|Verticillium alfalfae
CCF40303.1|Colletotrichum higginsianum
XP 007273744.1|Colletotrichum gloeosporioides
ENH79193.1|Colletotrichum orbiculare
XP 007601243.1|Colletotrichum fioriniae
KDN61754.1|Colletotrichum sublineola
XP 007919318.1|Togninia minima
XP 007832979.1|Pestalotiopsis fici
KEZ45899.1|Scedosporium apiospermum
KFY30705.1|Pseudogymnoascus pannorum
KFY82145.1|Pseudogymnoascus pannorum
KFX93245.1|Pseudogymnoascus pannorum
KFY09255.1|Pseudogymnoascus pannorum
EJB11993.1|Coccidioides immitis
W14116.1|Coccidioides posadasii
XP 003071913.1|Coccidioides posadasii
XP 007296016.1|Marssonina brunnea
XP 008086698.1|Glarea lozoyensis
XP 001585622.1|Sclerotinia sclerotiorum
ESZ95086.1|Sclerotinia borealis
XP 007585775.1|Neofusicoccum parvum
EKG20499.1|Macrophomina phaseolina
KEQ79941.1|Aureobasidium pullulans
KEQ94122.1|Aureobasidium subglaciale
KEQ74222.1|Aureobasidium namibiae
KEQ61318.1|Aureobasidium melanogenum
XP 664435.1|Aspergillus nidulans
XP 009156514.1|Exophiala dermatitidis
XP 007725686.1|Capronia coronata
XP 007761656.1|Cladophialophora yegresii
XP 008731963.1|Cladophialophora carrionii
KEF63115.1|Exophiala aquamarina
XP 007785326.1|Coniosporium apollinis
XP 007681573.1|Baudoinia compniacensis
EME41001.1|Dothistroma septosporum
XP 007930842.1|Pseudocercospora fijiensis
XP 001555138.1|Botrytis cinerea
EMR87533.1|Botrytis cinerea
CCD49440.2|Botrytis cinerea
CCT61161.1|Leptosphaeria maculans
XP 001940774.1|Pyrenophora tritici–repentis
XP 003301285.1|Pyrenophora teres
KFY02763.1|Pseudogymnoascus pannorum
EPS43598.1|Dactylellina haptotyla
XP 383600.1|Fusarium graminearum
KEY69561.1|Stachybotrys chartarum
KFY52580.1|Pseudogymnoascus pannorum
KFY87517.1|Pseudogymnoascus pannorum
KFY72963.1|Pseudogymnoascus pannorum
KFZ01000.1|Pseudogymnoascus pannorum
KFZ24080.1|Pseudogymnoascus pannorum
KFY46460.1|Pseudogymnoascus pannorum
KFY10748.1|Pseudogymnoascus pannorum
KFY46206.1|Pseudogymnoascus pannorum
XP 007732282.1|Capronia epimyces
KGO36725.1|Penicillium expansum
EKV13916.1|Penicillium digitatum
XP 002566754.1|Penicillium rubens
CDM29881.1|Penicillium roqueforti
BAE55398.1|Aspergillus oryzae
70000001155847486|Aspfl1
XP 006673183.1|Cordyceps militaris
XP 008599915.1|Beauveria bassiana
KGQ04234.1|Beauveria bassiana
XP 007813183.1|Metarhizium acridum
XP 007825790.1|Metarhizium robertsii
KFG77588.1|Metarhizium anisopliae
ACV04919.1|Aspergillus nidulans
XP 001218592.1|Aspergillus terreus
XP 001259225.1|Neosartorya fischeri
P 753203.1|Aspergillus fumigatus
KEY81835.1|Aspergillus fumigatus
EQB54164.1|Colletotrichum gloeosporioides
EFQ25994.1|Colletotrichum graminicola
CBV37356.1|Colletotrichum graminicola
CEJ80033.1|Torrubiella hemipterigena
XP 009225064.1|Gaeumannomyces graminis
XP 003711715.1|Magnaporthe oryzae
ELQ64697.1|Magnaporthe oryzae
XP 009259857.1|Fusarium pseudograminearum
EYB32527.1|Fusarium graminearum
EWG48913.1|Fusarium verticillioides
CCT74813.1|Fusarium fujikuroi
XA35864.1|Fusarium oxysporum
WZ32682.1|Fusarium oxysporum
EXL99660.1|Fusarium oxysporum
red: fungi
found in small number of aspergillus
transfer from distant fungi

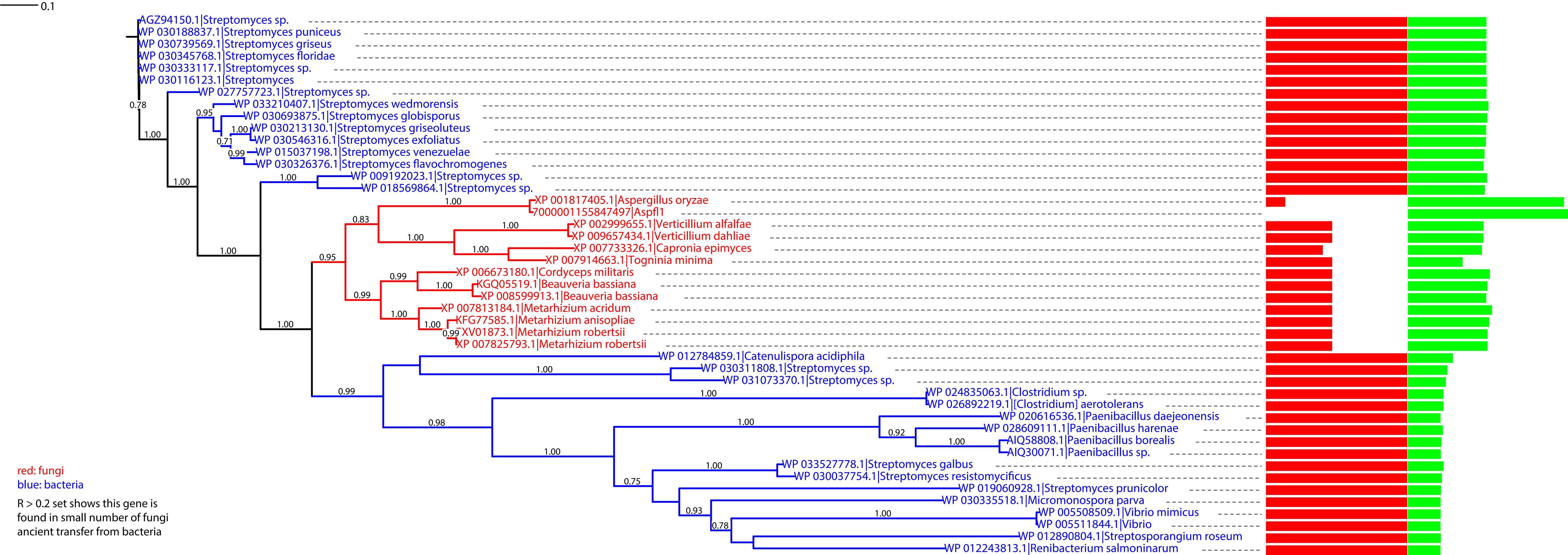
0.1
AGZ94150.1|Streptomyces sp.
WP 030188837.1|Streptomyces puniceus
WP 030739569.1|Streptomyces griseus
WP 030345768.1|Streptomyces floridae
WP 030333117.1|Streptomyces sp.
WP 030116123.1|Streptomyces
WP 027757723.1|Streptomyces sp.
WP 033210407.1|Streptomyces wedmorensis
WP 030693875.1|Streptomyces globisporus
WP 030213130.1|Streptomyces griseoluteus
WP 030546316.1|Streptomyces exfoliatus
WP 015037198.1|Streptomyces venezuelae
WP 030326376.1|Streptomyces flavochromogenes
WP 009192023.1|Streptomyces sp.
WP 018569864.1|Streptomyces sp.
XP 001817405.1|Aspergillus oryzae
7000001155847497|Aspfl1
XP 002999655.1|Verticillium alfalfae
XP 009657434.1|Verticillium dahliae
XP 007733326.1|Capronia epimyces
XP 007914663.1|Togninia minima
XP 006673180.1|Cordyceps militaris
KGQ05519.1|Beauveria bassiana
XP 008599913.1|Beauveria bassiana
XP 007813184.1|Metarhizium acridum
KFG77585.1|Metarhizium anisopliae
XV01873.1|Metarhizium robertsii
XP 007825793.1|Metarhizium robertsii
WP 012784859.1|Catenulispora acidiphila
WP 030311808.1|Streptomyces sp.
WP 031073370.1|Streptomyces sp.
WP 024835063.1|Clostridium sp.
WP 026892219.1|[Clostridium] aerotolerans
WP 020616536.1|Paenibacillus daejeonensis
WP 028609111.1|Paenibacillus harenae
AIQ58808.1|Paenibacillus borealis
AIQ30071.1|Paenibacillus sp.
WP 033527778.1|Streptomyces galbus
WP 030037754.1|Streptomyces resistomyicificus
WP 019060928.1|Streptomyces prunicolor
WP 030335518.1|Micromonospora parva
WP 005508509.1|Vibrio mimicus
WP 005511844.1|Vibrio
WP 012890804.1|Streptosporangium roseum
WP 012243813.1|Renibacterium salmoninarum
0.78
1.00
0.95
1.00
0.71
0.99
1.00
1.00
1.00
0.83
1.00
1.00
1.00
0.95
0.99
1.00
0.99
1.00
1.00
0.99
1.00
0.99
1.00
0.98
1.00
1.00
0.92
1.00
1.00
0.75
0.93
0.78
1.00
red: fungi
blue: bacteria
R > 0.2 set shows this gene is
found in small number of fungi
ancient transfer from bacteria

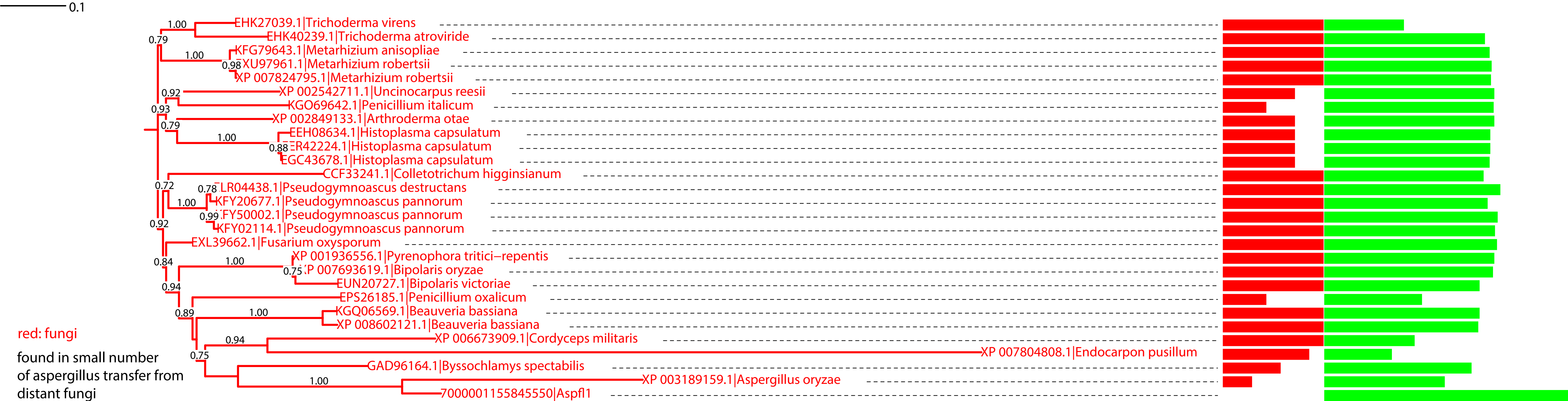
0.1
EHK27039.1|Trichoderma virens
EHK40239.1|Trichoderma atroviride
KFG79643.1|Metarhizium anisopliae
XU97961.1|Metarhizium robertsii
XP 007824795.1|Metarhizium robertsii
XP 002542711.1|Uncinocarpus reesii
KGO69642.1|Penicillium italicum
XP 002849133.1|Arthroderma otae
EEH08634.1|Histoplasma capsulatum
R42224.1|Histoplasma capsulatum
EGC43678.1|Histoplasma capsulatum
CCF33241.1|Colletotrichum higginsianum
LR04438.1|Pseudogymnoascus destructans
KFY20677.1|Pseudogymnoascus pannorum
FY50002.1|Pseudogymnoascus pannorum
KFY02114.1|Pseudogymnoascus pannorum
EXL39662.1|Fusarium oxysporum
XP 001936556.1|Pyrenophora tritici−repentis
P 007693619.1|Bipolaris oryzae
EUN20727.1|Bipolaris victoriae
EPS26185.1|Penicillium oxalicum
KGQ06569.1|Beauveria bassiana
XP 008602121.1|Beauveria bassiana
XP 006673909.1|Cordyceps militaris
XP 007804808.1|Endocarpon pusillum
GAD96164.1|Byssochlamys spectabilis
XP 003189159.1|Aspergillus oryzae
7000001155845550|Aspfl1
1.00
0.79
1.00
0.98
0.92
0.93
0.79
1.00
0.88
0.72
0.78
1.00
0.99
0.92
0.84
1.00
0.75
0.94
0.89
1.00
0.94
0.75
1.00
red: fungi
found in small number
of aspergillus transfer from
distant fungi

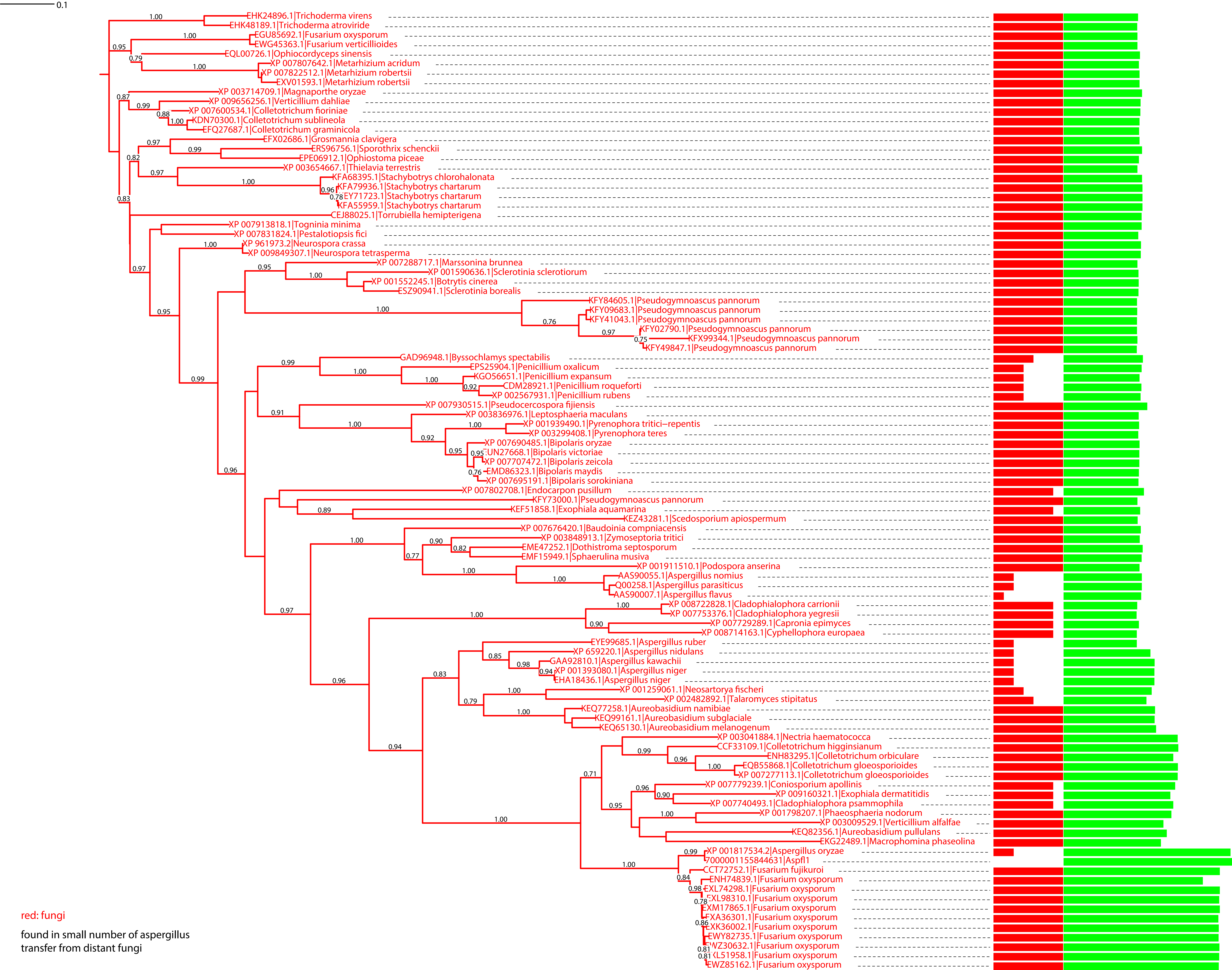
0.1
EHK24896.1|Trichoderma virens
EHK48189.1|Trichoderma atroviride
EGU85692.1|Fusarium oxysporum
EWG45363.1|Fusarium verticillioides
EQL00726.1|Ophiocordyceps sinensis
XP 007807642.1|Metarhizium acridum
XP 007822512.1|Metarhizium robertsii
EXV01593.1|Metarhizium robertsii
XP 003714709.1|Magnaporthe oryzae
XP 009656256.1|Verticillium dahliae
XP 007600534.1|Colletotrichum fioriniae
KDN70300.1|Colletotrichum sublineola
EFQ27687.1|Colletotrichum graminicola
EFX02686.1|Grosmannia clavigera
ERS96756.1|Sporothrix schenckii
EPE06912.1|Ophiostoma piceae
XP 003654667.1|Thielavia terrestris
KFA68395.1|Stachybotrys chlorohalonata
KFA79936.1|Stachybotrys chartarum
EY71723.1|Stachybotrys chartarum
KFA55959.1|Stachybotrys chartarum
CEJ88025.1|Torrubiella hemipterigena
XP 007913818.1|Togninia minima
XP 007831824.1|Pestalotiopsis fici
XP 961973.2|Neurospora crassa
XP 009849307.1|Neurospora tetrasperma
XP 007288717.1|Marssonina brunnea
XP 001590636.1|Sclerotinia sclerotiorum
XP 001552245.1|Botrytis cinerea
ESZ90941.1|Sclerotinia borealis
KFY84605.1|Pseudogymnoascus pannorum
KFY09683.1|Pseudogymnoascus pannorum
KFY41043.1|Pseudogymnoascus pannorum
KFY02790.1|Pseudogymnoascus pannorum
KFX99344.1|Pseudogymnoascus pannorum
KFY49847.1|Pseudogymnoascus pannorum
GAD96948.1|Byssochlamys spectabilis
EPS25904.1|Penicillium oxalicum
KGO56651.1|Penicillium expansum
CDM28921.1|Penicillium roqueforti
XP 002567931.1|Penicillium rubens
XP 007930515.1|Pseudocercospora fijiensis
XP 003836976.1|Leptosphaeria maculans
XP 001939490.1|Pyrenophora tritici–repentis
XP 003299408.1|Pyrenophora teres
XP 007690485.1|Bipolaris oryzae
UN27668.1|Bipolaris victoriae
XP 007707472.1|Bipolaris zeicola
EMD86323.1|Bipolaris maydis
XP 007695191.1|Bipolaris sorokiniana
XP 007802708.1|Endocarpon pusillum
KFY73000.1|Pseudogymnoascus pannorum
KEF51858.1|Exophiala aquamarina
KEZ43281.1|Scedosporium apiospermum
XP 007676420.1|Baudoinia compniacensis
XP 003848913.1|Zymoseptoria tritici
EME47252.1|Dothistroma septosporum
EMF15949.1|Sphaerulina musiva
XP 001911510.1|Podospora anserina
AAS90055.1|Aspergillus nomius
Q00258.1|Aspergillus parasiticus
AAS90007.1|Aspergillus flavus
XP 008722828.1|Cladophialophora carrionii
XP 007753376.1|Cladophialophora yegresii
XP 007729289.1|Capronia epimyces
XP 008714163.1|Cyphellophora europaea
EYE99685.1|Aspergillus ruber
XP 659220.1|Aspergillus nidulans
GAA92810.1|Aspergillus kawachii
XP 001393080.1|Aspergillus niger
EHA18436.1|Aspergillus niger
XP 001259061.1|Neosartorya fischeri
XP 002482892.1|Talaromyces stipitatus
KEQ77258.1|Aureobasidium namibiae
KEQ99161.1|Aureobasidium subglaciale
KEQ65130.1|Aureobasidium melanogenum
XP 003041884.1|Nectria haematococca
CCF33109.1|Colletotrichum higginsianum
ENH83295.1|Colletotrichum orbiculare
EQB55868.1|Colletotrichum gloeosporioides
XP 007277113.1|Colletotrichum gloeosporioides
XP 007779239.1|Coniosporium apollinis
XP 009160321.1|Exophiala dermatitidis
XP 007740493.1|Cladophialophora psammophila
XP 001798207.1|Phaeosphaeria nodorum
XP 003009529.1|Verticillium alfalfae
KEQ82356.1|Aureobasidium pullulans
EKG22489.1|Macrophomina phaseolina
XP 001817534.2|Aspergillus oryzae
7000001155844631|Aspfl1
CCT72752.1|Fusarium fujikuroi
ENH74839.1|Fusarium oxysporum
EXL74298.1|Fusarium oxysporum
EXL98310.1|Fusarium oxysporum
EXM17865.1|Fusarium oxysporum
EXA36301.1|Fusarium oxysporum
EXK36002.1|Fusarium oxysporum
EWY82735.1|Fusarium oxysporum
WZ30632.1|Fusarium oxysporum
KL51958.1|Fusarium oxysporum
EWZ85162.1|Fusarium oxysporum
red: fungi
found in small number of aspergillus
transfer from distant fungi

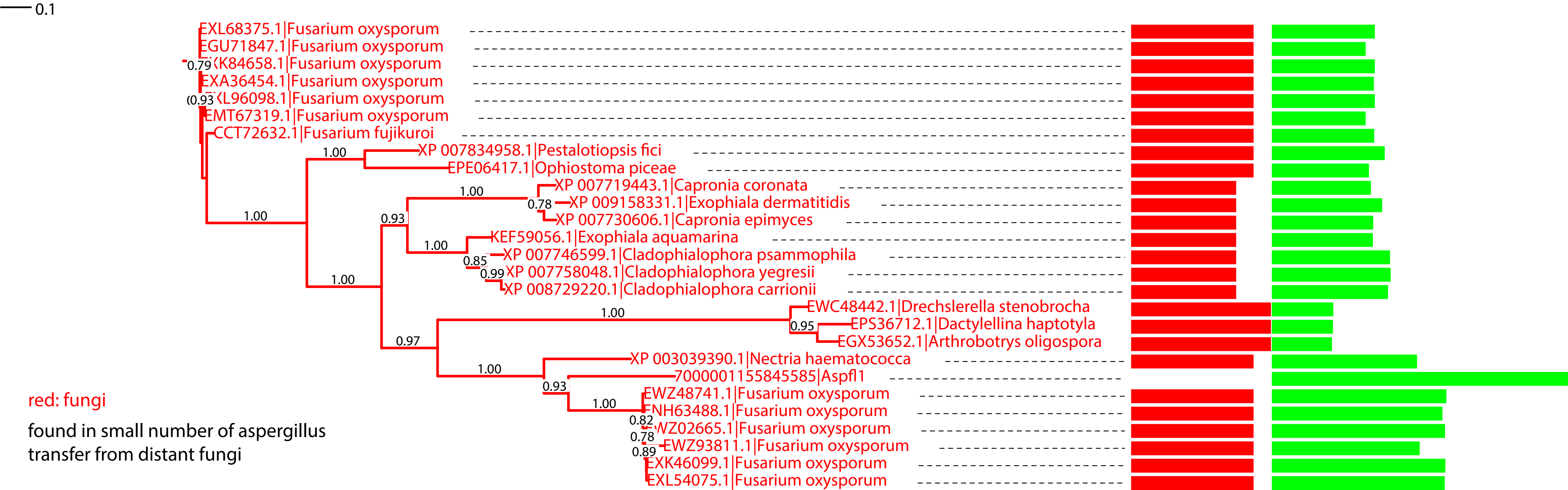

0.1
EXL68375.1|Fusarium oxysporum
EGU71847.1|Fusarium oxysporum
K84658.1|Fusarium oxysporum
EXA36454.1|Fusarium oxysporum
KL96098.1|Fusarium oxysporum
EMT67319.1|Fusarium oxysporum
CCT72632.1|Fusarium fujikuroi
XP 007834958.1|Pestalotiopsis fici
EPE06417.1|Ophiostoma piceae
XP 007719443.1|Capronia coronata
XP 009158331.1|Exophiala dermatitidis
XP 007730606.1|Capronia epimyces
KEF59056.1|Exophiala aquamarina
XP 007746599.1|Cladophialophora psammophila
XP 007758048.1|Cladophialophora yegresii
XP 008729220.1|Cladophialophora carrionii
EWC48442.1|Drechslerella stenobrocha
EPS36712.1|Dactylellina haptotyla
EGX53652.1|Arthrobotrys oligospora
XP 003039390.1|Nectria haematococca
7000001155845585|Aspfl1
EWZ48741.1|Fusarium oxysporum
NH63488.1|Fusarium oxysporum
WZ02665.1|Fusarium oxysporum
EWZ93811.1|Fusarium oxysporum
EXK46099.1|Fusarium oxysporum
EXL54075.1|Fusarium oxysporum
0.79
0.93
1.00
1.00
1.00
0.78
0.93
1.00
0.85
0.99
1.00
1.00
0.95
0.97
1.00
0.93
1.00
0.82
0.78
0.89
red: fungi
found in small number of aspergillus
transfer from distant fungi

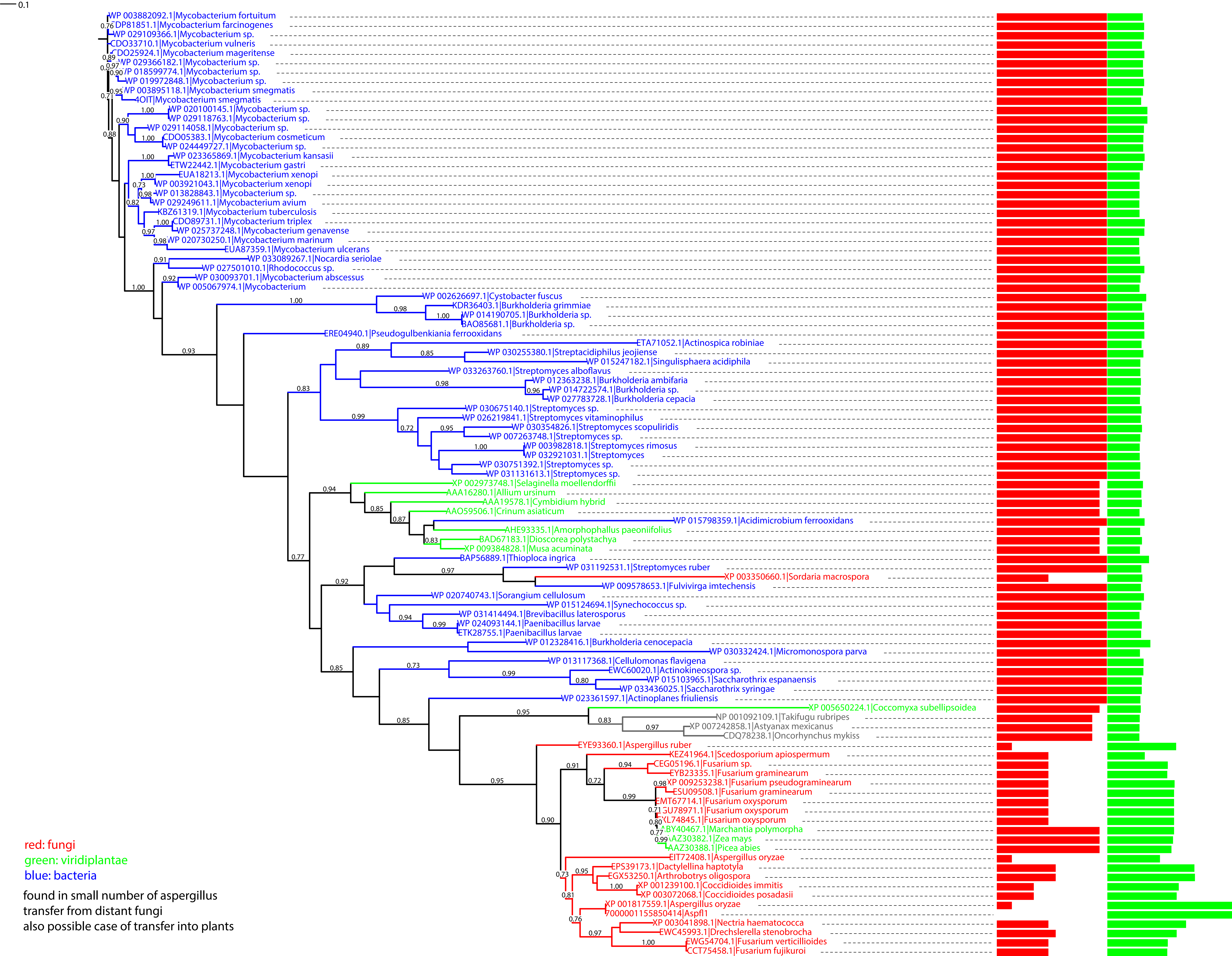
0.1
WP 003882092.1|Mycobacterium fortuitum
DP81851.1|Mycobacterium farcinogenes
WP 029109366.1|Mycobacterium sp.
CDO33710.1|Mycobacterium vulneris
CDO25924.1|Mycobacterium mageritense
WP 029366182.1|Mycobacterium sp.
P 018599774.1|Mycobacterium sp.
WP 019972848.1|Mycobacterium sp.
WP 003895118.1|Mycobacterium smegmatis
4OIT|Mycobacterium smegmatis
WP 020100145.1|Mycobacterium sp.
WP 029118763.1|Mycobacterium sp.
WP 029114058.1|Mycobacterium sp.
CDO05383.1|Mycobacterium cosmeticum
WP 024449727.1|Mycobacterium sp.
WP 023365869.1|Mycobacterium kansasii
ETW22442.1|Mycobacterium gastri
EUA18213.1|Mycobacterium xenopi
WP 003921043.1|Mycobacterium xenopi
WP 013828843.1|Mycobacterium sp.
WP 029249611.1|Mycobacterium avium
KBZ61319.1|Mycobacterium tuberculosis
CDO89731.1|Mycobacterium triplex
WP 025737248.1|Mycobacterium genavense
WP 020730250.1|Mycobacterium marinum
EUA87359.1|Mycobacterium ulcerans
WP 033089267.1|Nocardia seriolae
WP 027501010.1|Rhodococcus sp.
WP 030093701.1|Mycobacterium abscessus
WP 005067974.1|Mycobacterium
WP 002626697.1|Cystobacter fuscus
KDR36403.1|Burkholderia grimmiae
WP 014190705.1|Burkholderia sp.
BAO85681.1|Burkholderia sp.
ERE04940.1|Pseudogulbenkiania ferrooxidans
ETA71052.1|Actinospica robiniae
WP 030255380.1|Streptacidiphilus jeojiense
WP 015247182.1|Singulisphaera acidiphila
WP 033263760.1|Streptomyces alboflavus
WP 012363238.1|Burkholderia ambifaria
WP 014722574.1|Burkholderia sp.
WP 027783728.1|Burkholderia cepacia
WP 030675140.1|Streptomyces sp.
WP 026219841.1|Streptomyces vitaminophilus
WP 030354826.1|Streptomyces scopuliridis
WP 007263748.1|Streptomyces sp.
WP 003982818.1|Streptomyces rimosus
WP 032921031.1|Streptomyces
WP 030751392.1|Streptomyces sp.
WP 031131613.1|Streptomyces sp.
XP 002973748.1|Selaginella moellendorffii
AAA16280.1|Allium ursinum
AAA19578.1|Cymbidium hybrid
AAO59506.1|Crinum asiaticum
WP 015798359.1|Acidimicrobium ferrooxidans
AHE93335.1|Amorphophallus paeoniifolius
BAD67183.1|Dioscorea polystachya
XP 009384828.1|Musa acuminata
BAP56889.1|Thioploca ingrica
WP 031192531.1|Streptomyces ruber
XP 003350660.1|Sordaria macrospora
WP 009578653.1|Fulvivirga imtechensis
WP 020740743.1|Sorangium cellulosum
WP 015124694.1|Synechococcus sp.
WP 031414494.1|Brevibacillus laterosporus
WP 024093144.1|Paenibacillus larvae
ETK28755.1|Paenibacillus larvae
WP 012328416.1|Burkholderia cenocepacia
WP 030332424.1|Micromonospora parva
WP 013117368.1|Cellulomonas flavigena
EWC60020.1|Actinokineospora sp.
WP 015103965.1|Saccharothrix espanaensis
WP 033436025.1|Saccharothrix syringae
WP 023361597.1|Actinoplanes friuliensis
XP 005650224.1|Coccomyxa subellipsoidea
NP 001092109.1|Takifugu rubripes
XP 007242858.1|Astyanax mexicanus
CDQ78238.1|Oncorhynchus mykiss
EYE93360.1|Aspergillus ruber
KEZ41964.1|Scedosporium apiospermum
CEG05196.1|Fusarium sp.
EYB23335.1|Fusarium graminearum
XP 009253238.1|Fusarium pseudograminearum
ESU09508.1|Fusarium graminearum
EMT67714.1|Fusarium oxysporum
GU78971.1|Fusarium oxysporum
XL74845.1|Fusarium oxysporum
BY40467.1|Marchantia polymorpha
AZ30382.1|Zea mays
AAZ30388.1|Picea abies
EIT72408.1|Aspergillus oryzae
EPS39173.1|Dactylellina haptotyla
EGX53250.1|Arthrobotrys oligospora
XP 001239100.1|Coccidioides immitis
XP 003072068.1|Coccidioides posadasii
XP 001817559.1|Aspergillus oryzae
7000001155850414|Aspfl1
XP 003041898.1|Nectria haematococca
EWC45993.1|Drechslerella stenobrocha
EWG54704.1|Fusarium verticillioides
CCT75458.1|Fusarium fujikuroi
red: fungi
green: viridiplantae
blue: bacteria
found in small number of aspergillus
transfer from distant fungi
also possible case of transfer into plants

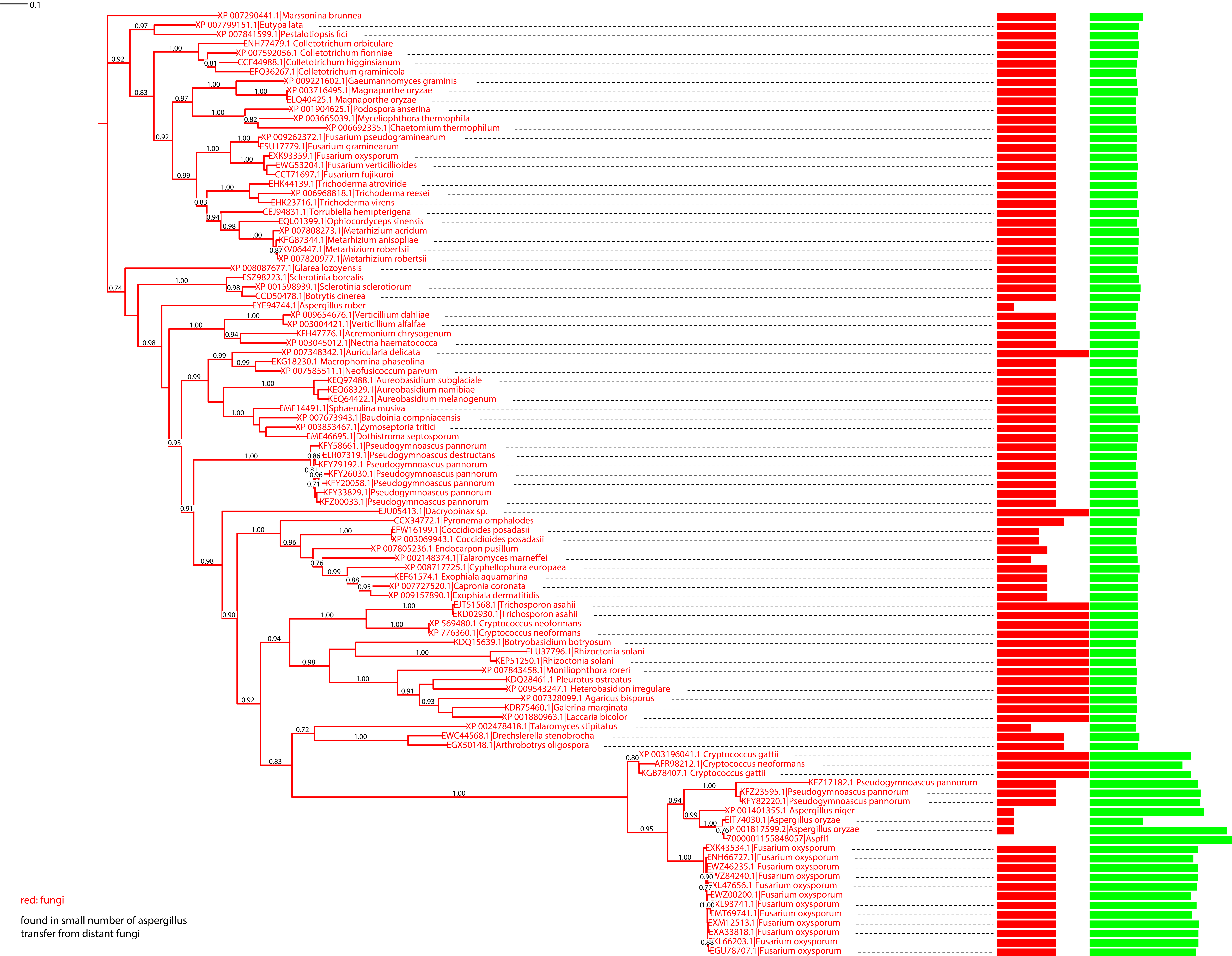
0.1
XP 007290441.1|Marssonina brunnea
XP 007799151.1|Eutypa lata
XP 007841599.1|Pestalotiopsis fici
ENH77479.1|Colletotrichum orbiculare
XP 007592056.1|Colletotrichum fioriniae
CCF44988.1|Colletotrichum higginsianum
EFQ36267.1|Colletotrichum graminicola
XP 009221602.1|Gaeumannomyces graminis
XP 003716495.1|Magnaporthe oryzae
ELQ40425.1|Magnaporthe oryzae
XP 001904625.1|Podospora anserina
XP 003665039.1|Myceliophthora thermophila
XP 006692335.1|Chaetomium thermophilum
XP 009262372.1|Fusarium pseudograminearum
ESU17779.1|Fusarium graminearum
EXK93359.1|Fusarium oxysporum
EWG53204.1|Fusarium verticillioides
CCT71697.1|Fusarium fujikuroi
EHK44139.1|Trichoderma atroviride
XP 006968818.1|Trichoderma reesei
EHK23716.1|Trichoderma virens
CEJ94831.1|Torrubiella hemipterigena
EQL01399.1|Ophiocordyceps sinensis
XP 007808273.1|Metarhizium acridum
KFG87344.1|Metarhizium anisopliae
KV06447.1|Metarhizium robertsii
XP 007820977.1|Metarhizium robertsii
XP 008087677.1|Glarea lozoyensis
ESZ98223.1|Sclerotinia borealis
XP 001598939.1|Sclerotinia sclerotiorum
CCD50478.1|Botrytis cinerea
EYE94744.1|Aspergillus ruber
XP 009654676.1|Verticillium dahliae
XP 003004421.1|Verticillium alfalfae
KFH47776.1|Acremonium chrysogenum
XP 003045012.1|Nectria haematococca
XP 007348342.1|Auricularia delicata
EKG18230.1|Macrophomina phaseolina
XP 007585511.1|Neofusicoccum parvum
KEQ97488.1|Aureobasidium subglaciale
KEQ68329.1|Aureobasidium namibiae
KEQ64422.1|Aureobasidium melanogenum
EMF14491.1|Sphaerulina musiva
XP 007673943.1|Baudoinia compniacensis
XP 003853467.1|Zymoseptoria tritici
EME46695.1|Dothistroma septosporum
KFY58661.1|Pseudogymnoascus pannorum
ELR07319.1|Pseudogymnoascus destructans
KFY79192.1|Pseudogymnoascus pannorum
KFY26030.1|Pseudogymnoascus pannorum
KFY20058.1|Pseudogymnoascus pannorum
KFY33829.1|Pseudogymnoascus pannorum
KFZ00033.1|Pseudogymnoascus pannorum
EJU05413.1|Dacryopinax sp.
CCX34772.1|Pyronema omphalodes
EFW16199.1|Coccidioides posadasii
XP 003069943.1|Coccidioides posadasii
XP 007805236.1|Endocarpon pusillum
XP 002148374.1|Talaromyces marneffei
XP 008717725.1|Cyphellophora europaea
KEF61574.1|Exophiala aquamarina
XP 007727520.1|Capronia coronata
XP 009157890.1|Exophiala dermatitidis
EJT51568.1|Trichosporon asahii
EKD02930.1|Trichosporon asahii
XP 569480.1|Cryptococcus neoformans
XP 776360.1|Cryptococcus neoformans
KDQ15639.1|Botryobasidium botryosum
ELU37796.1|Rhizoctonia solani
KEP51250.1|Rhizoctonia solani
XP 007843458.1|Moniliophthora roreri
KDQ28461.1|Pleurotus ostreatus
XP 009543247.1|Heterobasidion irregulare
XP 007328099.1|Agaricus bisporus
KDR75460.1|Galerina marginata
XP 001880963.1|Laccaria bicolor
XP 002478418.1|Talaromyces stipitatus
EWC44568.1|Drechslerella stenobrocha
EGX50148.1|Arthrobotrys oligospora
XP 003196041.1|Cryptococcus gattii
AFR98212.1|Cryptococcus neoformans
KGB78407.1|Cryptococcus gattii
KFZ17182.1|Pseudogymnoascus pannorum
KFZ23595.1|Pseudogymnoascus pannorum
KFY82220.1|Pseudogymnoascus pannorum
XP 001401355.1|Aspergillus niger
EIT74030.1|Aspergillus oryzae
001817599.2|Aspergillus oryzae
7000001155848057|Aspfl1
EXK43534.1|Fusarium oxysporum
ENH66727.1|Fusarium oxysporum
EWZ46235.1|Fusarium oxysporum
NZ84240.1|Fusarium oxysporum
XL47656.1|Fusarium oxysporum
EWZ00200.1|Fusarium oxysporum
XL93741.1|Fusarium oxysporum
EMT69741.1|Fusarium oxysporum
EXM12513.1|Fusarium oxysporum
EXA33818.1|Fusarium oxysporum
KL66203.1|Fusarium oxysporum
EGU78707.1|Fusarium oxysporum
red: fungi
found in small number of aspergillus
transfer from distant fungi

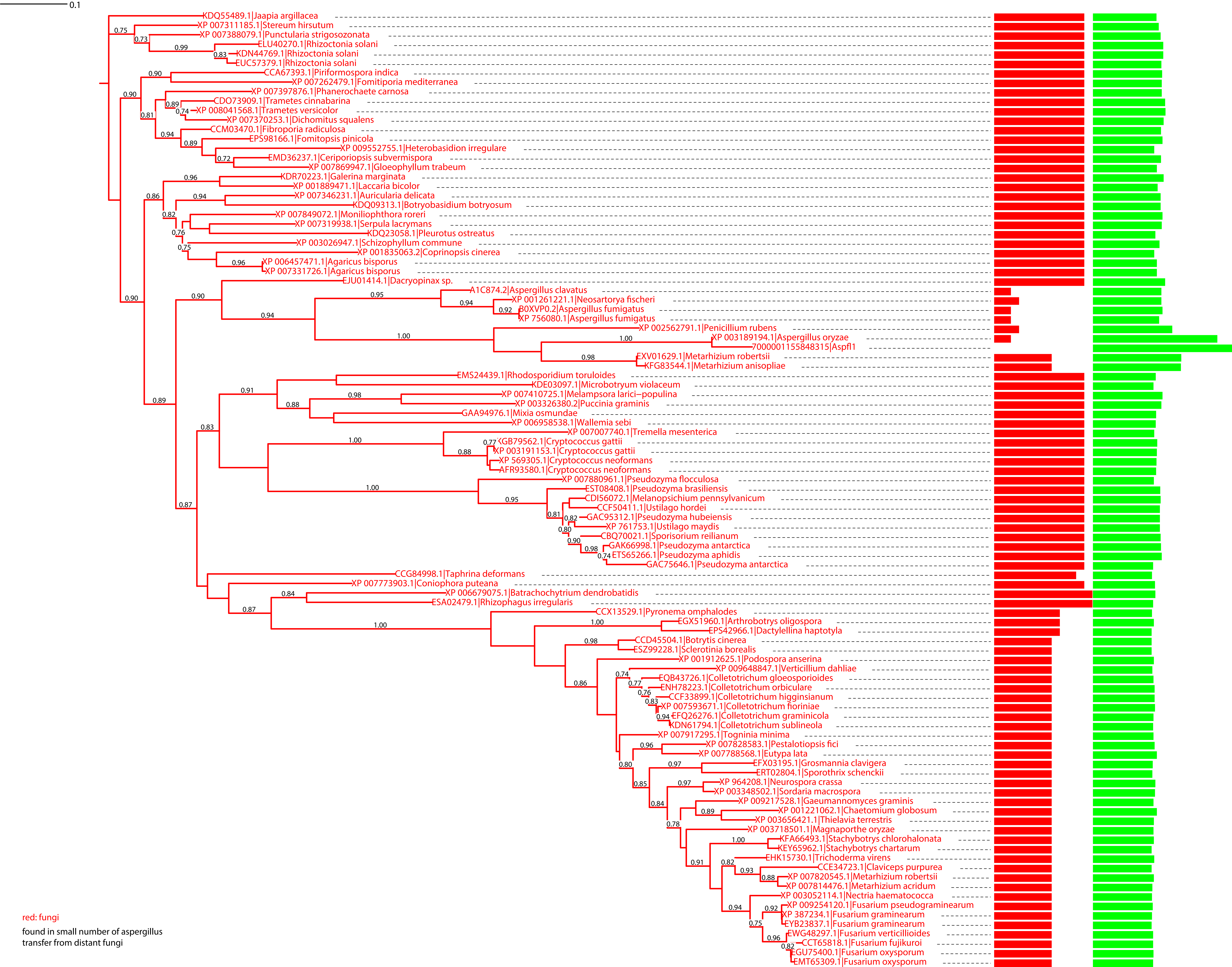
0.1
KDQ55489.1|Jaapia argillacea
XP 007311185.1|Stereum hirsutum
XP 007388079.1|Punctularia strigosozonata
ELU40270.1|Rhizoctonia solani
KDN44769.1|Rhizoctonia solani
EUC57379.1|Rhizoctonia solani
CCA67393.1|Piriformospora indica
XP 007262479.1|Fomitiporia mediterranea
XP 007397876.1|Phanerochaete carnosa
CDO73909.1|Trametes cinnabarina
XP 008041568.1|Trametes versicolor
XP 007370253.1|Dichomitus squalens
CCM03470.1|Fibroporia radiculosa
EPS98166.1|Fomitopsis pinicola
XP 009552755.1|Heterobasidion irregulare
EMD36237.1|Ceriporiopsis subvermispora
XP 007869947.1|Gloeophyllum trabeum
KDR70223.1|Galerina marginata
XP 001889471.1|Laccaria bicolor
XP 007346231.1|Auricularia delicata
KDQ09313.1|Botryobasidium botryosum
XP 007849072.1|Moniliophthora roreri
XP 007319938.1|Serpula lacrymans
KDQ23058.1|Pleurotus ostreatus
XP 003026947.1|Schizophyllum commune
XP 001835063.2|Coprinopsis cinerea
XP 006457471.1|Agaricus bisporus
XP 007331726.1|Agaricus bisporus
EJU01414.1|Dacryopinax sp.
A1C874.2|Aspergillus clavatus
XP 001261221.1|Neosartorya fischeri
B0XVP0.2|Aspergillus fumigatus
XP 756080.1|Aspergillus fumigatus
XP 002562791.1|Penicillium rubens
XP 003189194.1|Aspergillus oryzae
7000001155848315|Aspfl1
EXV01629.1|Metarhizium robertsii
KFG83544.1|Metarhizium anisopliae
EMS24439.1|Rhodosporidium toruloides
KDE03097.1|Microbotryum violaceum
XP 007410725.1|Melampsora larici-populina
XP 003326380.2|Puccinia graminis
GAA94976.1|Mixia osmundae
XP 006958538.1|Wallemia sebi
XP 007007740.1|Tremella mesenterica
KGB79562.1|Cryptococcus gattii
XP 003191153.1|Cryptococcus gattii
XP 569305.1|Cryptococcus neoformans
AFR93580.1|Cryptococcus neoformans
XP 007880961.1|Pseudozyma flocculosa
EST08408.1|Pseudozyma brasiliensis
CDI56072.1|Melanopsichium pennsylvanicum
CCF50411.1|Ustilago hordei
GAC95312.1|Pseudozyma hubeiensis
XP 761753.1|Ustilago maydis
CBQ70021.1|Sporisorium reilianum
GAK66998.1|Pseudozyma antarctica
ETS65266.1|Pseudozyma aphidis
GAC75646.1|Pseudozyma antarctica
CCG84998.1|Taphrina deformans
XP 007773903.1|Coniophora puteana
XP 006679075.1|Batrachochytrium dendrobatidis
ESA02479.1|Rhizophagus irregularis
CCX13529.1|Pyronema omphalodes
EGX51960.1|Arthrobotrys oligospora
EPS42966.1|Dactylellina haptotyla
CCD45504.1|Botrytis cinerea
ESZ99228.1|Sclerotinia borealis
XP 001912625.1|Podospora anserina
XP 009648847.1|Verticillium dahliae
EQB43726.1|Colletotrichum gloeosporioides
ENH78223.1|Colletotrichum orbiculare
CCF33899.1|Colletotrichum higginsianum
XP 007593671.1|Colletotrichum fioriniae
EFQ26276.1|Colletotrichum graminicola
KDN61794.1|Colletotrichum sublineola
XP 007917295.1|Togninia minima
XP 007828583.1|Pestalotiopsis fici
XP 007788568.1|Eutypa lata
EFX03195.1|Grosmannia clavigera
ERT02804.1|Sporothrix schenckii
XP 964208.1|Neurospora crassa
XP 003348502.1|Sordaria macrospora
XP 009217528.1|Gaeumannomyces graminis
XP 001221062.1|Chaetomium globosum
XP 003656421.1|Thielavia terrestris
XP 003718501.1|Magnaporthe oryzae
KFA66493.1|Stachybotrys chlorohalonata
KEY65962.1|Stachybotrys chartarum
EHK15730.1|Trichoderma virens
CCE34723.1|Claviceps purpurea
XP 007820545.1|Metarhizium robertsii
XP 007814476.1|Metarhizium acridum
XP 003052114.1|Nectria haematococca
XP 009254120.1|Fusarium pseudograminearum
XP 387234.1|Fusarium graminearum
EYB23837.1|Fusarium graminearum
EWG48297.1|Fusarium verticillioides
CCT65818.1|Fusarium fujikuroi
EGU75400.1|Fusarium oxysporum
EMT65309.1|Fusarium oxysporum
red: fungi
found in small number of aspergillus
transfer from distant fungi

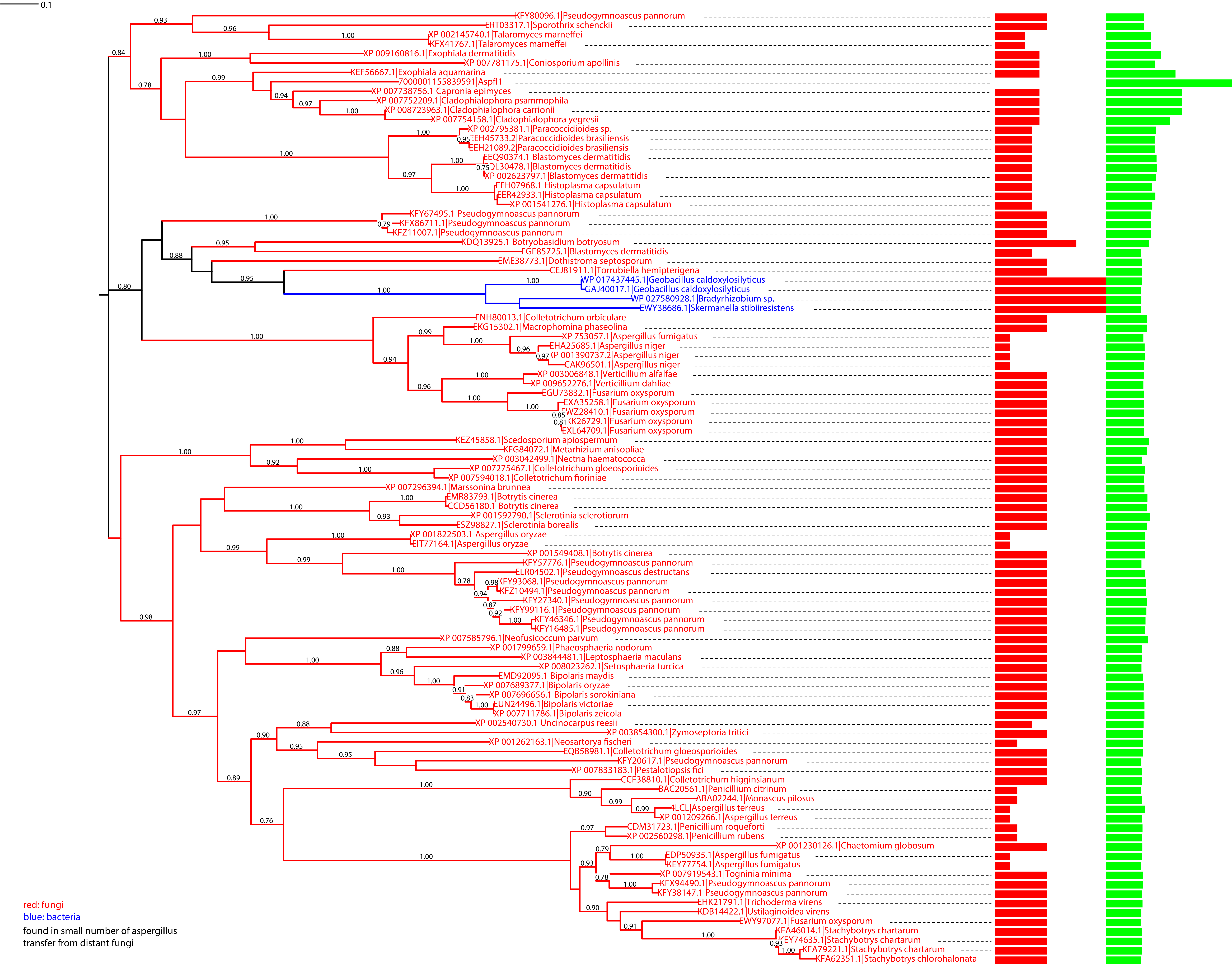

0.1
KFY80096.1|Pseudogymnoascus pannorum
ERT03317.1|Sporothrix schenckii
XP 002145740.1|Talaromyces marneffei
KFX41767.1|Talaromyces marneffei
XP 009160816.1|Exophiala dermatitidis
XP 007781175.1|Coniosporium apollinis
KEF56667.1|Exophiala aquamarina
7000001155839591|Aspfl1
XP 007738756.1|Capronia epimyces
XP 007752209.1|Cladophialophora psammophila
XP 008723963.1|Cladophialophora carrionii
XP 007754158.1|Cladophialophora yegresii
XP 002795381.1|Paracoccidioides sp.
EH45733.2|Paracoccidioides brasiliensis
EEH21089.2|Paracoccidioides brasiliensis
EEQ90374.1|Blastomyces dermatitidis
L30478.1|Blastomyces dermatitidis
XP 002623797.1|Blastomyces dermatitidis
EEH07968.1|Histoplasma capsulatum
EER42933.1|Histoplasma capsulatum
XP 001541276.1|Histoplasma capsulatum
KFY67495.1|Pseudogymnoascus pannorum
KFX86711.1|Pseudogymnoascus pannorum
KFZ11007.1|Pseudogymnoascus pannorum
KDQ13925.1|Botryobasidium botryosum
EGE85725.1|Blastomyces dermatitidis
EME38773.1|Dothistroma septosporum
CEJ81911.1|Torrubiella hemipterigena
WP 017437445.1|Geobacillus caldoxylosilyticus
GAJ40017.1|Geobacillus caldoxylosilyticus
WP 027580928.1|Bradyrhizobium sp.
EWY38686.1|Skermanella stibiiresistens
ENH80013.1|Colletotrichum orbiculare
EKG15302.1|Macrophomina phaseolina
XP 753057.1|Aspergillus fumigatus
EHA25685.1|Aspergillus niger
XP 001390737.2|Aspergillus niger
CAK96501.1|Aspergillus niger
XP 003006848.1|Verticillium alfalfae
XP 009652276.1|Verticillium dahliae
EGU73832.1|Fusarium oxysporum
EXA35258.1|Fusarium oxysporum
EWZ28410.1|Fusarium oxysporum
K26729.1|Fusarium oxysporum
EXL64709.1|Fusarium oxysporum
KEZ45858.1|Scedosporium apiospermum
KFG84072.1|Metarhizium anisopliae
XP 003042499.1|Nectria haematococca
XP 007275467.1|Colletotrichum gloeosporioides
XP 007594018.1|Colletotrichum fioriniae
XP 007296394.1|Marssonina brunnea
EMR83793.1|Botrytis cinerea
CCD56180.1|Botrytis cinerea
XP 001592790.1|Sclerotinia sclerotiorum
ESZ98827.1|Sclerotinia borealis
XP 001822503.1|Aspergillus oryzae
EIT77164.1|Aspergillus oryzae
XP 001549408.1|Botrytis cinerea
KFY57776.1|Pseudogymnoascus pannorum
ELR04502.1|Pseudogymnoascus destructans
KFY93068.1|Pseudogymnoascus pannorum
KFZ10494.1|Pseudogymnoascus pannorum
KFY27340.1|Pseudogymnoascus pannorum
KFY99116.1|Pseudogymnoascus pannorum
KFY46346.1|Pseudogymnoascus pannorum
KFY16485.1|Pseudogymnoascus pannorum
XP 007585796.1|Neofusicoccum parvum
XP 001799659.1|Phaeosphaeria nodorum
XP 003844481.1|Leptosphaeria maculans
XP 008023262.1|Setosphaeria turcica
EMD92095.1|Bipolaris maydis
XP 007689377.1|Bipolaris oryzae
XP 007696656.1|Bipolaris sorokiniana
EUN24496.1|Bipolaris victoriae
XP 007711786.1|Bipolaris zeicola
XP 002540730.1|Uncinocarpus reesii
XP 003854300.1|Zymoseptoria tritici
XP 001262163.1|Neosartorya fischeri
EQB58981.1|Colletotrichum gloeosporioides
KFY20617.1|Pseudogymnoascus pannorum
XP 007833183.1|Pestalotiopsis fici
CCF38810.1|Colletotrichum higginsianum
BAC20561.1|Penicillium citrinum
ABA02244.1|Monascus pilosus
4LCL|Aspergillus terreus
XP 001209266.1|Aspergillus terreus
CDM31723.1|Penicillium roqueforti
XP 002560298.1|Penicillium rubens
XP 001230126.1|Chaetomium globosum
EDP50935.1|Aspergillus fumigatus
KEY77754.1|Aspergillus fumigatus
XP 007919543.1|Togninia minima
KFX94490.1|Pseudogymnoascus pannorum
KFY38147.1|Pseudogymnoascus pannorum
EHK21791.1|Trichoderma virens
KDB14422.1|Ustilaginoidea virens
EWY97077.1|Fusarium oxysporum
KFA46014.1|Stachybotrys chartarum
KEY74635.1|Stachybotrys chartarum
KFA79221.1|Stachybotrys chartarum
KFA62351.1|Stachybotrys chlorohalonata
red: fungi
blue: bacteria
found in small number of aspergillus
transfer from distant fungi

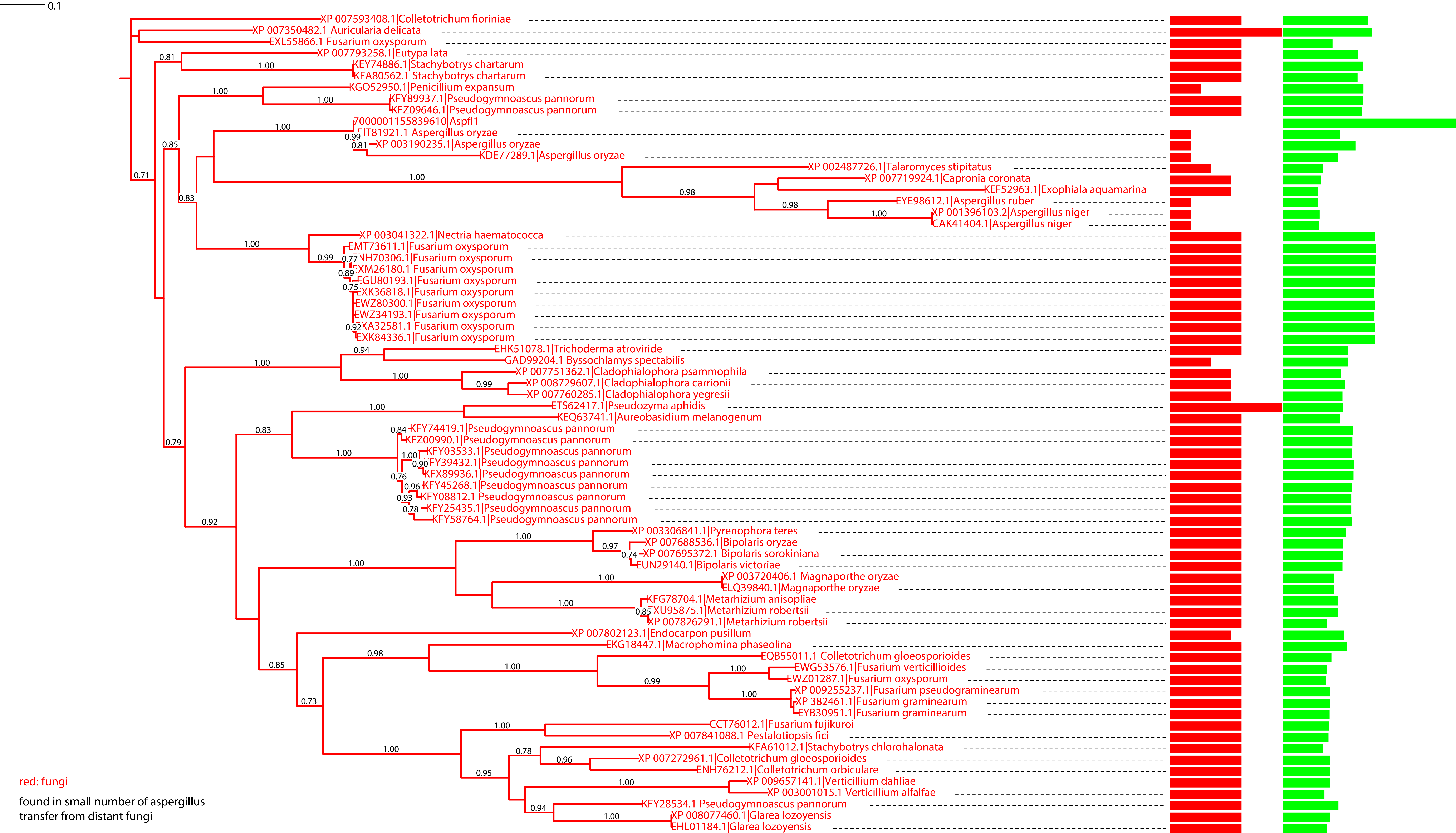
0.1
XP 007593408.1|Colletotrichum fioriniae
XP 007350482.1|Auricularia delicata
EXL55866.1|Fusarium oxysporum
XP 007793258.1|Eutypa lata
KEY74886.1|Stachybotrys chartarum
KFA80562.1|Stachybotrys chartarum
KGO52950.1|Penicillium expansum
KFY89937.1|Pseudogymnoascus pannorum
KFZ09646.1|Pseudogymnoascus pannorum
7000001155839610|Aspfl1
FIT81921.1|Aspergillus oryzae
XP 003190235.1|Aspergillus oryzae
KDE77289.1|Aspergillus oryzae
XP 002487726.1|Talaromyces stipitatus
XP 007719924.1|Capronia coronata
KEF52963.1|Exophiala aquamarina
EYE98612.1|Aspergillus ruber
XP 001396103.2|Aspergillus niger
CAK41404.1|Aspergillus niger
XP 003041322.1|Nectria haematococca
EMT73611.1|Fusarium oxysporum
VH70306.1|Fusarium oxysporum
XM26180.1|Fusarium oxysporum
FGU80193.1|Fusarium oxysporum
EXK36818.1|Fusarium oxysporum
EWZ80300.1|Fusarium oxysporum
EWZ34193.1|Fusarium oxysporum
XA32581.1|Fusarium oxysporum
EXK84336.1|Fusarium oxysporum
EHK51078.1|Trichoderma atroviride
GAD99204.1|Byssochlamys spectabilis
XP 007751362.1|Cladophialophora psammophila
XP 008729607.1|Cladophialophora carrionii
XP 007760285.1|Cladophialophora yegresii
ETS62417.1|Pseudozyma aphidis
KEQ63741.1|Aureobasidium melanogenum
KFY74419.1|Pseudogymnoascus pannorum
KFZ00990.1|Pseudogymnoascus pannorum
KFY03533.1|Pseudogymnoascus pannorum
FY39432.1|Pseudogymnoascus pannorum
KFX89936.1|Pseudogymnoascus pannorum
KFY45268.1|Pseudogymnoascus pannorum
KFY08812.1|Pseudogymnoascus pannorum
KFY25435.1|Pseudogymnoascus pannorum
KFY58764.1|Pseudogymnoascus pannorum
XP 003306841.1|Pyrenophora teres
XP 007688536.1|Bipolaris oryzae
XP 007695372.1|Bipolaris sorokiniana
EUN29140.1|Bipolaris victoriae
XP 003720406.1|Magnaporthe oryzae
ELQ39840.1|Magnaporthe oryzae
KFG78704.1|Metarhizium anisopliae
XU95875.1|Metarhizium robertsii
XP 007826291.1|Metarhizium robertsii
XP 007802123.1|Endocarpon pusillum
EKG18447.1|Macrophomina phaseolina
EQB55011.1|Colletotrichum gloeosporioides
EWG53576.1|Fusarium verticillioides
EWZ01287.1|Fusarium oxysporum
XP 009255237.1|Fusarium pseudograminearum
XP 382461.1|Fusarium graminearum
EYB30951.1|Fusarium graminearum
CCT76012.1|Fusarium fujikuroi
XP 007841088.1|Pestalotiopsis fici
KFA61012.1|Stachybotrys chlorohalonata
XP 007272961.1|Colletotrichum gloeosporioides
ENH76212.1|Colletotrichum orbiculare
XP 009657141.1|Verticillium dahliae
XP 003001015.1|Verticillium alfalfae
KFY28534.1|Pseudogymnoascus pannorum
XP 008077460.1|Glarea lozoyensis
EHL01184.1|Glarea lozoyensis
red: fungi
found in small number of aspergillus
transfer from distant fungi

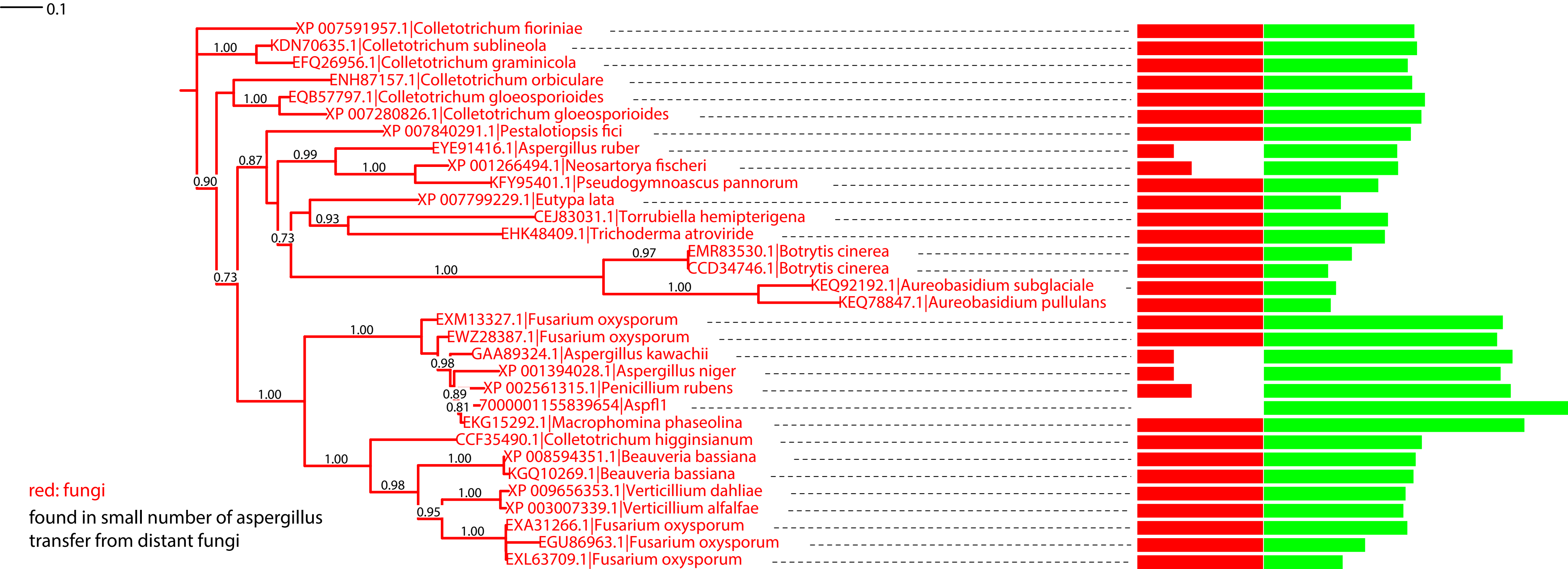

0.1
XP 007591957.1|Colletotrichum fioriniae
KDN70635.1|Colletotrichum sublineola
EFQ26956.1|Colletotrichum graminicola
ENH87157.1|Colletotrichum orbiculare
EQB57797.1|Colletotrichum gloeosporioides
XP 007280826.1|Colletotrichum gloeosporioides
XP 007840291.1|Pestalotiopsis fici
EYE91416.1|Aspergillus ruber
XP 001266494.1|Neosartorya fischeri
KFY95401.1|Pseudogymnoascus pannorum
XP 007799229.1|Eutypa lata
CEJ83031.1|Torrubiella hemipterigena
EHK48409.1|Trichoderma atroviride
EMR83530.1|Botrytis cinerea
CCD34746.1|Botrytis cinerea
KEQ92192.1|Aureobasidium subglaciale
KEQ78847.1|Aureobasidium pullulans
EXM13327.1|Fusarium oxysporum
EWZ28387.1|Fusarium oxysporum
GAA89324.1|Aspergillus kawachii
XP 001394028.1|Aspergillus niger
XP 002561315.1|Penicillium rubens
7000001155839654|Aspfl1
EKG15292.1|Macrophomina phaseolina
CCF35490.1|Colletotrichum higginsianum
XP 008594351.1|Beauveria bassiana
KGQ10269.1|Beauveria bassiana
XP 009656353.1|Verticillium dahliae
XP 003007339.1|Verticillium alfalfae
EXA31266.1|Fusarium oxysporum
EGU86963.1|Fusarium oxysporum
EXL63709.1|Fusarium oxysporum
1.00
1.00
0.90
0.87
0.99
1.00
0.93
0.73
0.73
0.97
1.00
1.00
1.00
0.98
0.89
0.81
1.00
1.00
1.00
0.98
1.00
0.95
1.00
red: fungi
found in small number of aspergillus
transfer from distant fungi

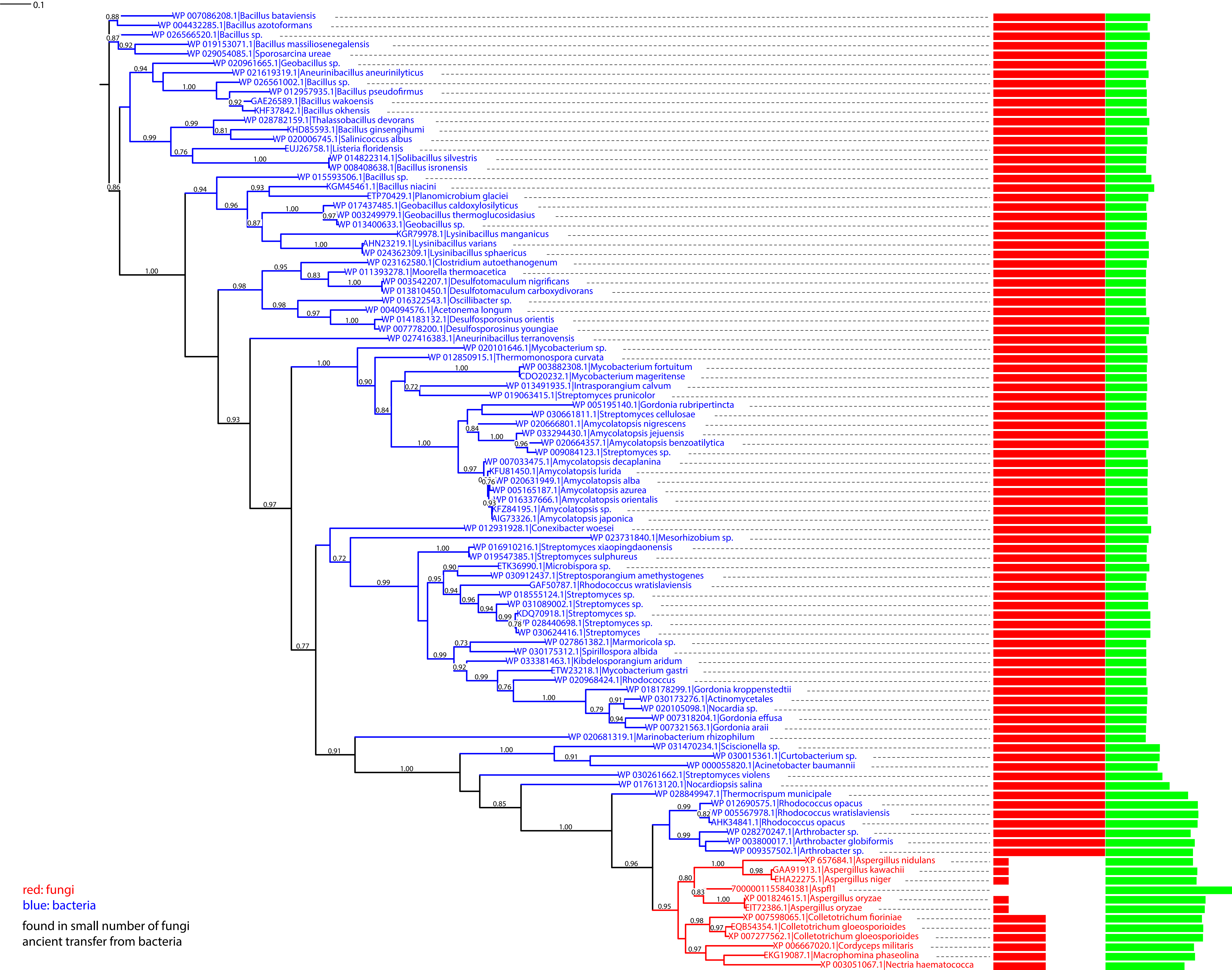
0.1
WP 007086208.1|Bacillus bataviensis
WP 004432285.1|Bacillus azotoformans
WP 026566520.1|Bacillus sp.
WP 019153071.1|Bacillus massiliosenegalensis
WP 029054085.1|Sporosarcina ureae
WP 020961665.1|Geobacillus sp.
WP 021619319.1|Aneurinibacillus aneurinilyticus
WP 026561002.1|Bacillus sp.
WP 012957935.1|Bacillus pseudofirmus
GAE26589.1|Bacillus wakoensis
KHF37842.1|Bacillus okhensis
WP 028782159.1|Thalassobacillus devorans
KHD85593.1|Bacillus ginsengihumi
WP 020006745.1|Salinicoccus albus
EUJ26758.1|Listeria floridensis
WP 014822314.1|Solibacillus silvestris
WP 008408638.1|Bacillus isronensis
WP 015593506.1|Bacillus sp.
KGM45461.1|Bacillus niacini
ETP70429.1|Planomicrobium glaciei
WP 017437485.1|Geobacillus caldoxylosilyticus
WP 003249979.1|Geobacillus thermoglucosidasius
WP 013400633.1|Geobacillus sp.
KGR79978.1|Lysinibacillus manganicus
AHN23219.1|Lysinibacillus varians
WP 024362309.1|Lysinibacillus sphaericus
WP 023162580.1|Clostridium autoethanogenum
WP 011393278.1|Moorella thermoacetica
WP 003542207.1|Desulfotomaculum nigrificans
WP 013810450.1|Desulfotomaculum carboxydivorans
WP 016322543.1|Oscillibacter sp.
WP 004094576.1|Acetonema longum
WP 014183132.1|Desulfosporosinus orientis
WP 007778200.1|Desulfosporosinus youngiae
WP 027416383.1|Aneurinibacillus terranovensis
WP 020101646.1|Mycobacterium sp.
WP 012850915.1|Thermomonospora curvata
WP 003882308.1|Mycobacterium fortuitum
CDO20232.1|Mycobacterium mageritense
WP 013491935.1|Intrasporangium calvum
WP 019063415.1|Streptomyces prunicolor
WP 005195140.1|Gordonia rubripertincta
WP 030661811.1|Streptomyces cellulosae
WP 020666801.1|Amycolatopsis nigrescens
WP 033294430.1|Amycolatopsis jejuensis
WP 020664357.1|Amycolatopsis benzoatilytica
WP 009084123.1|Streptomyces sp.
WP 007033475.1|Amycolatopsis decaplanina
KFU81450.1|Amycolatopsis lurida
WP 020631949.1|Amycolatopsis alba
WP 005165187.1|Amycolatopsis azurea
WP 016337666.1|Amycolatopsis orientalis
KFZ84195.1|Amycolatopsis sp.
AIG73326.1|Amycolatopsis japonica
WP 012931928.1|Conexibacter woesei
WP 023731840.1|Mesorhizobium sp.
WP 016910216.1|Streptomyces xiaopingdaonensis
WP 019547385.1|Streptomyces sulphureus
ETK36990.1|Microbispora sp.
WP 030912437.1|Streptosporangium amethystogenes
GAF50787.1|Rhodococcus wratislaviensis
WP 018555124.1|Streptomyces sp.
WP 031089002.1|Streptomyces sp.
KDQ70918.1|Streptomyces sp.
WP 028440698.1|Streptomyces sp.
WP 030624416.1|Streptomyces
WP 027861382.1|Marmoricola sp.
WP 030175312.1|Spirillospora albida
WP 033381463.1|Kibdelosporangium aridum
ETW23218.1|Mycobacterium gastri
WP 020968424.1|Rhodococcus
WP 018178299.1|Gordonia kroppenstedtii
WP 030173276.1|Actinomycetales
WP 020105098.1|Nocardia sp.
WP 007318204.1|Gordonia effusa
WP 007321563.1|Gordonia araii
WP 020681319.1|Marinobacterium rhizophilum
WP 031470234.1|Sciscionella sp.
WP 030015361.1|Curtobacterium sp.
WP 000055820.1|Acinetobacter baumannii
WP 030261662.1|Streptomyces violens
WP 017613120.1|Nocardiopsis salina
WP 028849947.1|Thermocrispum municipale
WP 012690575.1|Rhodococcus opacus
WP 005567978.1|Rhodococcus wratislaviensis
AHK34841.1|Rhodococcus opacus
WP 028270247.1|Arthrobacter sp.
WP 003800017.1|Arthrobacter globiformis
WP 009357502.1|Arthrobacter sp.
XP 657684.1|Aspergillus nidulans
GAA91913.1|Aspergillus kawachii
EHA22275.1|Aspergillus niger
7000001155840381|Aspfl1
XP 001824615.1|Aspergillus oryzae
EIT72386.1|Aspergillus oryzae
XP 007598065.1|Colletotrichum fioriniae
EQB54354.1|Colletotrichum gloeosporioides
XP 007277562.1|Colletotrichum gloeosporioides
XP 006667020.1|Cordyceps militaris
EKG19087.1|Macrophomina phaseolina
XP 003051067.1|Nectria haematococca
red: fungi
blue: bacteria
found in small number of fungi
ancient transfer from bacteria

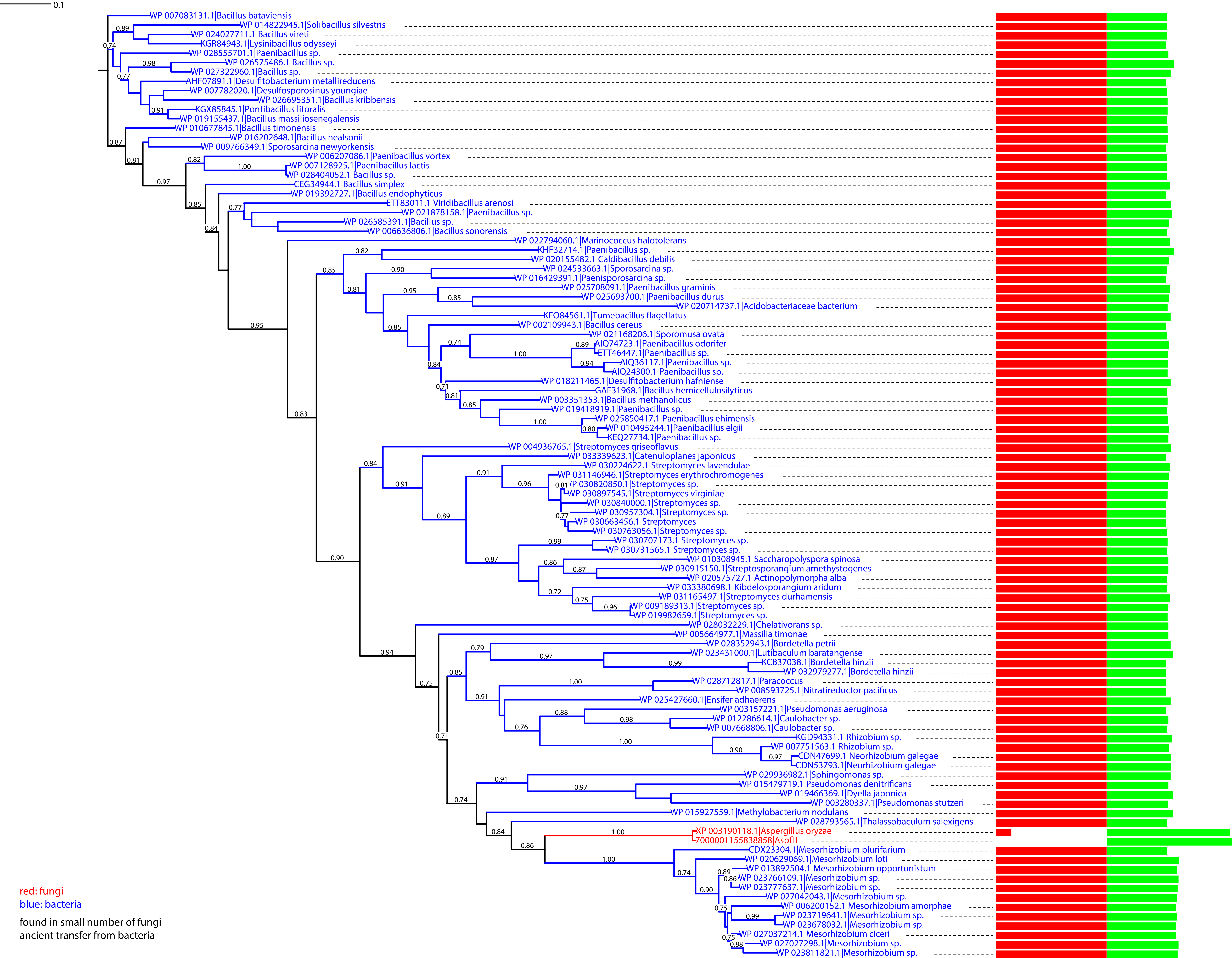
0.1
WP 007083131.1|Bacillus bataviensis
WP 014822945.1|Solibacillus silvestris
WP 024027711.1|Bacillus vireti
KGR84943.1|Lysinibacillus odysseyi
WP 028555701.1|Paenibacillus sp.
WP 026575486.1|Bacillus sp.
WP 027322960.1|Bacillus sp.
AHF07891.1|Desulfitobacterium metallireducens
WP 007782020.1|Desulfosporosinus youngiae
WP 026695351.1|Bacillus kribbensis
KGX85845.1|Pontibacillus litoralis
WP 019155437.1|Bacillus massiliosenegalensis
WP 010677845.1|Bacillus timonensis
WP 016202648.1|Bacillus nealsonii
WP 009766349.1|Sporosarcina newyorkensis
WP 006207086.1|Paenibacillus vortex
WP 007128925.1|Paenibacillus lactis
WP 028404052.1|Bacillus sp.
CEG34944.1|Bacillus simplex
WP 019392727.1|Bacillus endophyticus
ETT83011.1|Viridibacillus arenosi
WP 021878158.1|Paenibacillus sp.
WP 026585391.1|Bacillus sp.
WP 006636806.1|Bacillus sonorensis
WP 022794060.1|Marinococcus halotolerans
KHF32714.1|Paenibacillus sp.
WP 020155482.1|Caldibacillus debilis
WP 024533663.1|Sporosarcina sp.
WP 016429391.1|Paenisporosarcina sp.
WP 025708091.1|Paenibacillus graminis
WP 025693700.1|Paenibacillus durus
WP 020714737.1|Acidobacteriaceae bacterium
KEO84561.1|Tumebacillus flagellatus
WP 002109943.1|Bacillus cereus
WP 021168206.1|Sporomusa ovata
AIQ74723.1|Paenibacillus odorifer
ETT46447.1|Paenibacillus sp.
AIQ36117.1|Paenibacillus sp.
AIQ24300.1|Paenibacillus sp.
WP 018211465.1|Desulfitobacterium hafniense
GAE31968.1|Bacillus hemicellulosilyticus
WP 003351353.1|Bacillus methanolicus
WP 019418919.1|Paenibacillus sp.
WP 025850417.1|Paenibacillus ehimensis
WP 010495244.1|Paenibacillus elgii
KEQ27734.1|Paenibacillus sp.
WP 004936765.1|Streptomyces griseoflavus
WP 033339623.1|Catenuloplanes japonicus
WP 030224622.1|Streptomyces lavendulae
WP 031146946.1|Streptomyces erythrochromogenes
/P 030820850.1|Streptomyces sp.
WP 030897545.1|Streptomyces virginiae
WP 030840000.1|Streptomyces sp.
WP 030957304.1|Streptomyces sp.
WP 030663456.1|Streptomyces
WP 030763056.1|Streptomyces sp.
WP 030707173.1|Streptomyces sp.
WP 030731565.1|Streptomyces sp.
WP 010308945.1|Saccharopolyspora spinosa
WP 030915150.1|Streptosporangium amethystogenes
WP 020575727.1|Actinopolymorpha alba
WP 033380698.1|Kibdelosporangium aridum
WP 031165497.1|Streptomyces durhamensis
WP 009189313.1|Streptomyces sp.
WP 019982659.1|Streptomyces sp.
WP 028032229.1|Chelativorans sp.
WP 005664977.1|Massilia timonae
WP 028352943.1|Bordetella petrii
WP 023431000.1|Lutibaculum baratangense
KCB37038.1|Bordetella hinzii
WP 032979277.1|Bordetella hinzii
WP 028712817.1|Paracoccus
WP 008593725.1|Nitratireductor pacificus
WP 025427660.1|Ensifer adhaerens
WP 003157221.1|Pseudomonas aeruginosa
WP 012286614.1|Caulobacter sp.
WP 007668806.1|Caulobacter sp.
KGD94331.1|Rhizobium sp.
WP 007751563.1|Rhizobium sp.
CDN47699.1|Neorhizobium galegae
CDN53793.1|Neorhizobium galegae
WP 029936982.1|Sphingomonas sp.
WP 015479719.1|Pseudomonas denitrificans
WP 019466369.1|Dyella japonica
WP 003280337.1|Pseudomonas stutzeri
WP 015927559.1|Methylobacterium nodulans
WP 028793565.1|Thalassobaculum salexigens
XP 003190118.1|Aspergillus oryzae
7000001155838858|Aspfl1
CDX23304.1|Mesorhizobium plurifarium
WP 020629069.1|Mesorhizobium loti
WP 013892504.1|Mesorhizobium opportunistum
WP 023766109.1|Mesorhizobium sp.
WP 023777637.1|Mesorhizobium sp.
WP 027042043.1|Mesorhizobium sp.
WP 006200152.1|Mesorhizobium amorphae
WP 023719641.1|Mesorhizobium sp.
WP 023678032.1|Mesorhizobium sp.
WP 027037214.1|Mesorhizobium ciceri
WP 027027298.1|Mesorhizobium sp.
WP 023811821.1|Mesorhizobium sp.
red: fungi
blue: bacteria
found in small number of fungi
ancient transfer from bacteria

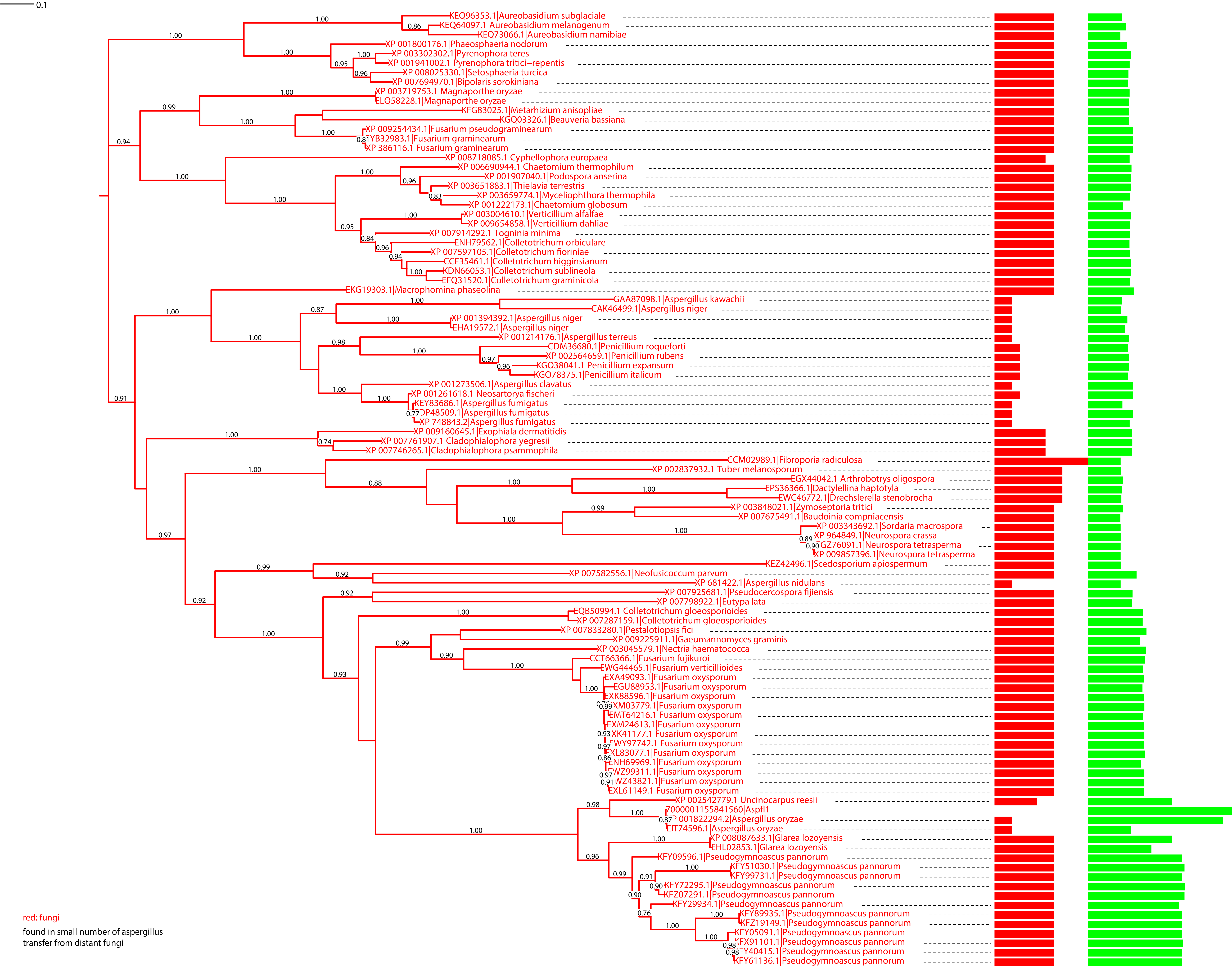
0.1
KEQ96353.1|Aureobasidium subglaciale
KEQ64097.1|Aureobasidium melanogenum
KEQ73066.1|Aureobasidium namibiae
XP 001800176.1|Phaeosphaeria nodorum
XP 003302302.1|Pyrenophora teres
XP 001941002.1|Pyrenophora tritici–repentis
XP 008025330.1|Setosphaeria turcica
XP 007694970.1|Bipolaris sorokiniana
XP 003719753.1|Magnaporthe oryzae
ELQ58228.1|Magnaporthe oryzae
KFG83025.1|Metarhizium anisopliae
KGQ03326.1|Beauveria bassiana
XP 009254434.1|Fusarium pseudograminearum
YB32983.1|Fusarium graminearum
XP 386116.1|Fusarium graminearum
XP 008718085.1|Cyphellophora europaea
XP 006690944.1|Chaetomium thermophilum
XP 001907040.1|Podospora anserina
XP 003651883.1|Thielavia terrestris
XP 003659774.1|Myceliophthora thermophila
XP 001222173.1|Chaetomium globosum
XP 003004610.1|Verticillium alfalfae
XP 009654858.1|Verticillium dahliae
XP 007914292.1|Togninia minima
ENH79562.1|Colletotrichum orbiculare
XP 007597105.1|Colletotrichum fioriniae
CCF35461.1|Colletotrichum higginsianum
KDN66053.1|Colletotrichum sublineola
EFQ31520.1|Colletotrichum graminicola
EKG19303.1|Macrophomina phaseolina
GAA87098.1|Aspergillus kawachii
CAK46499.1|Aspergillus niger
XP 001394392.1|Aspergillus niger
EHA19572.1|Aspergillus niger
XP 001214176.1|Aspergillus terreus
CDM36680.1|Penicillium roqueforti
XP 002564659.1|Penicillium rubens
KGO38041.1|Penicillium expansum
KGO78375.1|Penicillium italicum
XP 001273506.1|Aspergillus clavatus
XP 001261618.1|Neosartorya fischeri
KEY83686.1|Aspergillus fumigatus
P48509.1|Aspergillus fumigatus
XP 748843.2|Aspergillus fumigatus
XP 009160645.1|Exophiala dermatitidis
XP 007761907.1|Cladophialophora yegresii
XP 007746265.1|Cladophialophora psammophila
CCM02989.1|Fibroporia radiculosa
XP 002837932.1|Tuber melanosporum
EGX44042.1|Arthrobotrys oligospora
EPS36366.1|Dactylellina haptotyla
EWC46772.1|Drechslerella stenobrocha
XP 003848021.1|Zymoseptoria tritici
XP 007675491.1|Baudoinia compniacensis
XP 003343692.1|Sordaria macrospora
XP 964849.1|Neurospora crassa
GZ76091.1|Neurospora tetrasperma
XP 009857396.1|Neurospora tetrasperma
KEZ42496.1|Scedosporium apiospermum
XP 007582556.1|Neofusicoccum parvum
XP 681422.1|Aspergillus nidulans
XP 007925681.1|Pseudocercospora fijiensis
XP 007798922.1|Eutypa lata
EQB50994.1|Colletotrichum gloeosporioides
XP 007287159.1|Colletotrichum gloeosporioides
XP 007833280.1|Pestalotiopsis fici
XP 009225911.1|Gaeumannomyces graminis
XP 003045579.1|Nectria haematococca
CCT66366.1|Fusarium fujikuroi
EWG44465.1|Fusarium verticillioides
EXA49093.1|Fusarium oxysporum
EGU88953.1|Fusarium oxysporum
EXK88596.1|Fusarium oxysporum
XM03779.1|Fusarium oxysporum
EMT64216.1|Fusarium oxysporum
EXM24613.1|Fusarium oxysporum
XK41177.1|Fusarium oxysporum
WY97742.1|Fusarium oxysporum
XL83077.1|Fusarium oxysporum
ENH69969.1|Fusarium oxysporum
WZ99311.1|Fusarium oxysporum
WZ43821.1|Fusarium oxysporum
EXL61149.1|Fusarium oxysporum
XP 002542779.1|Uncinocarpus reesii
7000001155841560|Aspfl1
001822294.2|Aspergillus oryzae
EIT74596.1|Aspergillus oryzae
XP 008087633.1|Glarea lozoyensis
EHL02853.1|Glarea lozoyensis
KFY09596.1|Pseudogymnoascus pannorum
KFY51030.1|Pseudogymnoascus pannorum
KFY99731.1|Pseudogymnoascus pannorum
KFY72295.1|Pseudogymnoascus pannorum
KFZ07291.1|Pseudogymnoascus pannorum
KFY29934.1|Pseudogymnoascus pannorum
KFY89935.1|Pseudogymnoascus pannorum
KFZ19149.1|Pseudogymnoascus pannorum
KFY05091.1|Pseudogymnoascus pannorum
FX91101.1|Pseudogymnoascus pannorum
Y40415.1|Pseudogymnoascus pannorum
KFY61136.1|Pseudogymnoascus pannorum
red: fungi
found in small number of aspergillus
transfer from distant fungi

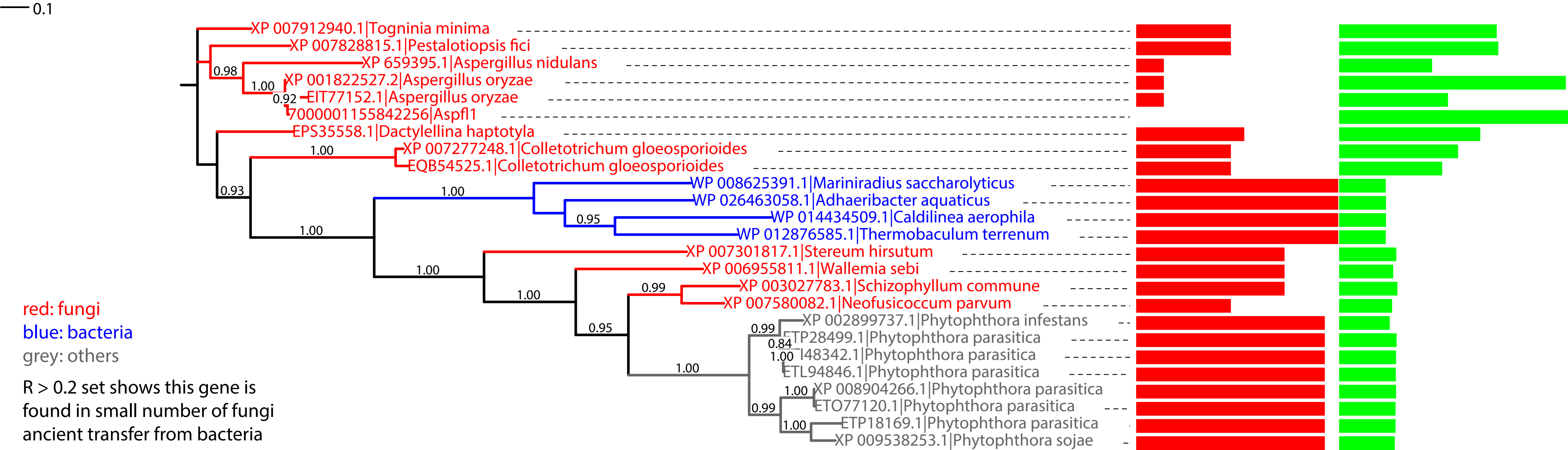
0.1
XP 007912940.1|Togninia minima
XP 007828815.1|Pestalotiopsis fici
XP 659395.1|Aspergillus nidulans
XP 001822527.2|Aspergillus oryzae
EIT77152.1|Aspergillus oryzae
7000001155842256|Aspfl1
EPS35558.1|Dactylellina haptotyla
XP 007277248.1|Colletotrichum gloeosporioides
EQB54525.1|Colletotrichum gloeosporioides
WP 008625391.1|Mariniradius saccharolyticus
WP 026463058.1|Adhaeribacter aquaticus
WP 014434509.1|Caldilinea aerophila
WP 012876585.1|Thermobaculum terrenum
XP 007301817.1|Stereum hirsutum
XP 006955811.1|Wallemia sebi
XP 003027783.1|Schizophyllum commune
XP 007580082.1|Neofusicoccum parvum
XP 002899737.1|Phytophthora infestans
ETP28499.1|Phytophthora parasitica
ETI48342.1|Phytophthora parasitica
ETL94846.1|Phytophthora parasitica
XP 008904266.1|Phytophthora parasitica
ETO77120.1|Phytophthora parasitica
ETP18169.1|Phytophthora parasitica
XP 009538253.1|Phytophthora sojae
0.98
1.00
0.92
1.00
0.93
1.00
0.95
1.00
1.00
1.00
0.99
0.95
1.00
0.99
0.84
1.00
1.00
0.99
1.00
red: fungi
blue: bacteria
grey: others
R > 0.2 set shows this gene is
found in small number of fungi
ancient transfer from bacteria

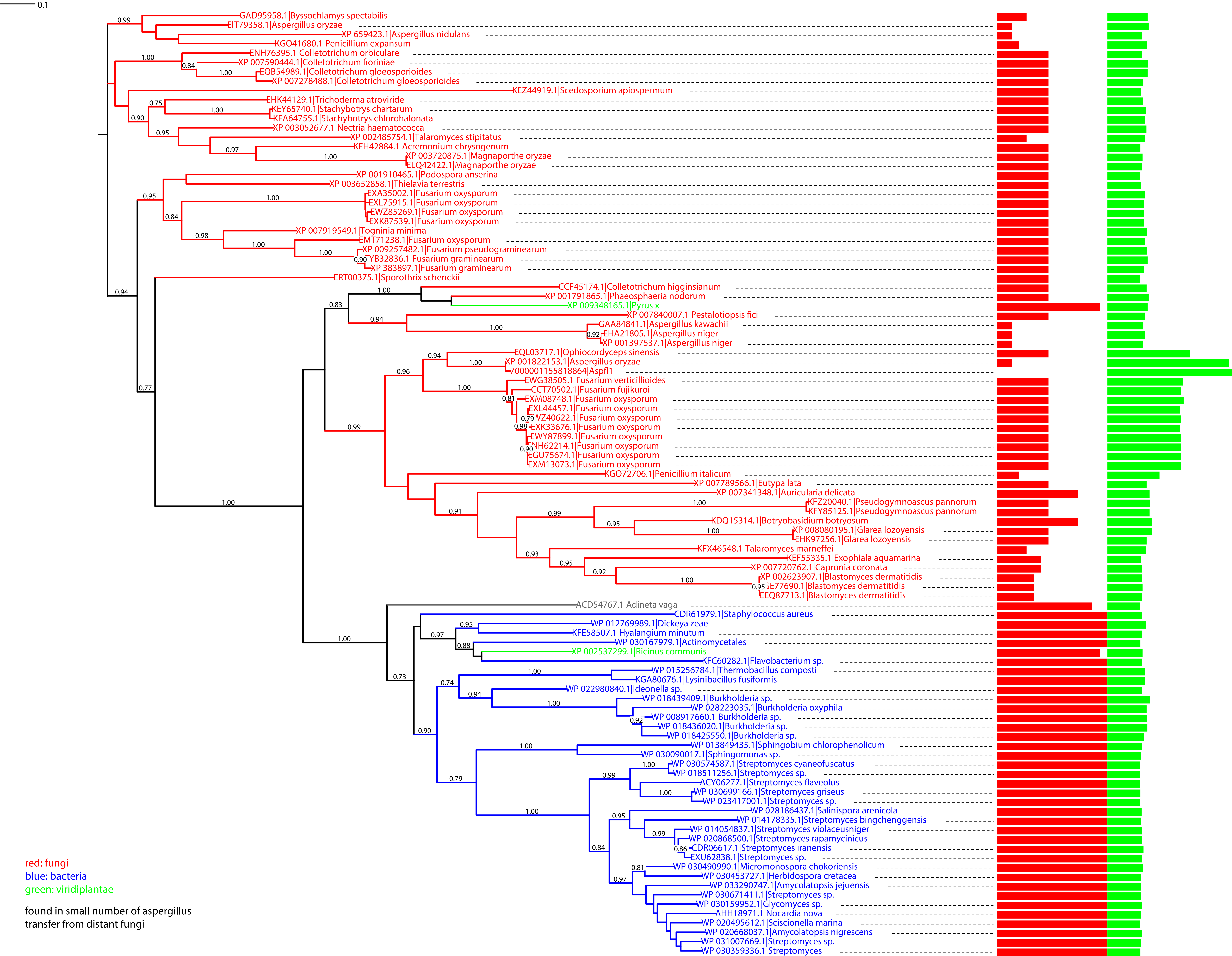
0.1
GAD95958.1|Byssochlamys spectabilis
EIT79358.1|Aspergillus oryzae
XP 659423.1|Aspergillus nidulans
KGO41680.1|Penicillium expansum
ENH76395.1|Colletotrichum orbiculare
XP 007590444.1|Colletotrichum fioriniae
EQB54989.1|Colletotrichum gloeosporioides
XP 007278488.1|Colletotrichum gloeosporioides
KEZ44919.1|Scedosporium apiospermum
EHK44129.1|Trichoderma atroviride
KEY65740.1|Stachybotrys chartarum
KFA64755.1|Stachybotrys chlorohalonata
XP 003052677.1|Nectria haematococca
XP 002485754.1|Talaromyces stipitatus
KFH42884.1|Acremonium chrysogenum
XP 003720875.1|Magnaporthe oryzae
ELQ42422.1|Magnaporthe oryzae
XP 001910465.1|Podospora anserina
XP 003652858.1|Thielavia terrestris
EXA35002.1|Fusarium oxysporum
EXL75915.1|Fusarium oxysporum
EWZ85269.1|Fusarium oxysporum
EXK87539.1|Fusarium oxysporum
XP 007919549.1|Togninia minima
EMT71238.1|Fusarium oxysporum
XP 009257482.1|Fusarium pseudograminearum
YB32836.1|Fusarium graminearum
XP 383897.1|Fusarium graminearum
ERT00375.1|Sporothrix schenckii
CCF45174.1|Colletotrichum higginsianum
XP 001791865.1|Phaeosphaeria nodorum
XP 009348165.1|Pyrus x
XP 007840007.1|Pestalotiopsis fici
GAA84841.1|Aspergillus kawachii
EHA21805.1|Aspergillus niger
XP 001397537.1|Aspergillus niger
EQL03717.1|Ophiocordyceps sinensis
XP 001822153.1|Aspergillus oryzae
7000001155818864|Aspfl1
EWG38505.1|Fusarium verticillioides
CCT70502.1|Fusarium fujikuroi
EXM08748.1|Fusarium oxysporum
EXL44457.1|Fusarium oxysporum
NZ40622.1|Fusarium oxysporum
EXK33676.1|Fusarium oxysporum
EWY87899.1|Fusarium oxysporum
NH62214.1|Fusarium oxysporum
EGU75674.1|Fusarium oxysporum
EXM13073.1|Fusarium oxysporum
KGO72706.1|Penicillium italicum
XP 007789566.1|Eutypa lata
XP 007341348.1|Auricularia delicata
KFZ20040.1|Pseudogymnoascus pannorum
KFY85125.1|Pseudogymnoascus pannorum
KDQ15314.1|Botryobasidium botryosum
XP 008080195.1|Glarea lozoyensis
EHK97256.1|Glarea lozoyensis
KFX46548.1|Talaromyces marneffei
KEF55335.1|Exophiala aquamarina
XP 007720762.1|Capronia coronata
XP 002623907.1|Blastomyces dermatitidis
E77690.1|Blastomyces dermatitidis
EEQ87713.1|Blastomyces dermatitidis
ACD54767.1|Adineta vaga
CDR61979.1|Staphylococcus aureus
WP 012769989.1|Dickeya zeae
KFE58507.1|Hyalangium minutum
WP 030167979.1|Actinomycetales
XP 002537299.1|Ricinus communis
KFC60282.1|Flavobacterium sp.
WP 015256784.1|Thermobacillus composti
KGA80676.1|Lysinibacillus fusiformis
WP 022980840.1|Ideonella sp.
WP 018439409.1|Burkholderia sp.
WP 028223035.1|Burkholderia oxyphila
WP 008917660.1|Burkholderia sp.
WP 018436020.1|Burkholderia sp.
WP 018425550.1|Burkholderia sp.
WP 013849435.1|Sphingobium chlorophenolicum
WP 030090017.1|Sphingomonas sp.
WP 030574587.1|Streptomyces cyaneofuscatus
WP 018511256.1|Streptomyces sp.
ACY06277.1|Streptomyces flaveolus
WP 030699166.1|Streptomyces griseus
WP 023417001.1|Streptomyces sp.
WP 028186437.1|Salinispora arenicola
WP 014178335.1|Streptomyces bingchenggensis
WP 014054837.1|Streptomyces violaceusniger
WP 020868500.1|Streptomyces rapamycinicus
CDR06617.1|Streptomyces iranensis
EXU62838.1|Streptomyces sp.
WP 030490990.1|Micromonospora chokoriensis
WP 030453727.1|Herbidospora cretacea
WP 033290747.1|Amycolatopsis jejuensis
WP 030671411.1|Streptomyces sp.
WP 030159952.1|Glycomyces sp.
AHH18971.1|Nocardia nova
WP 020495612.1|Sciscionella marina
WP 020668037.1|Amycolatopsis nigrescens
WP 031007669.1|Streptomyces sp.
WP 030359336.1|Streptomyces
red: fungi
blue: bacteria
green: viridiplantae
found in small number of aspergillus
transfer from distant fungi

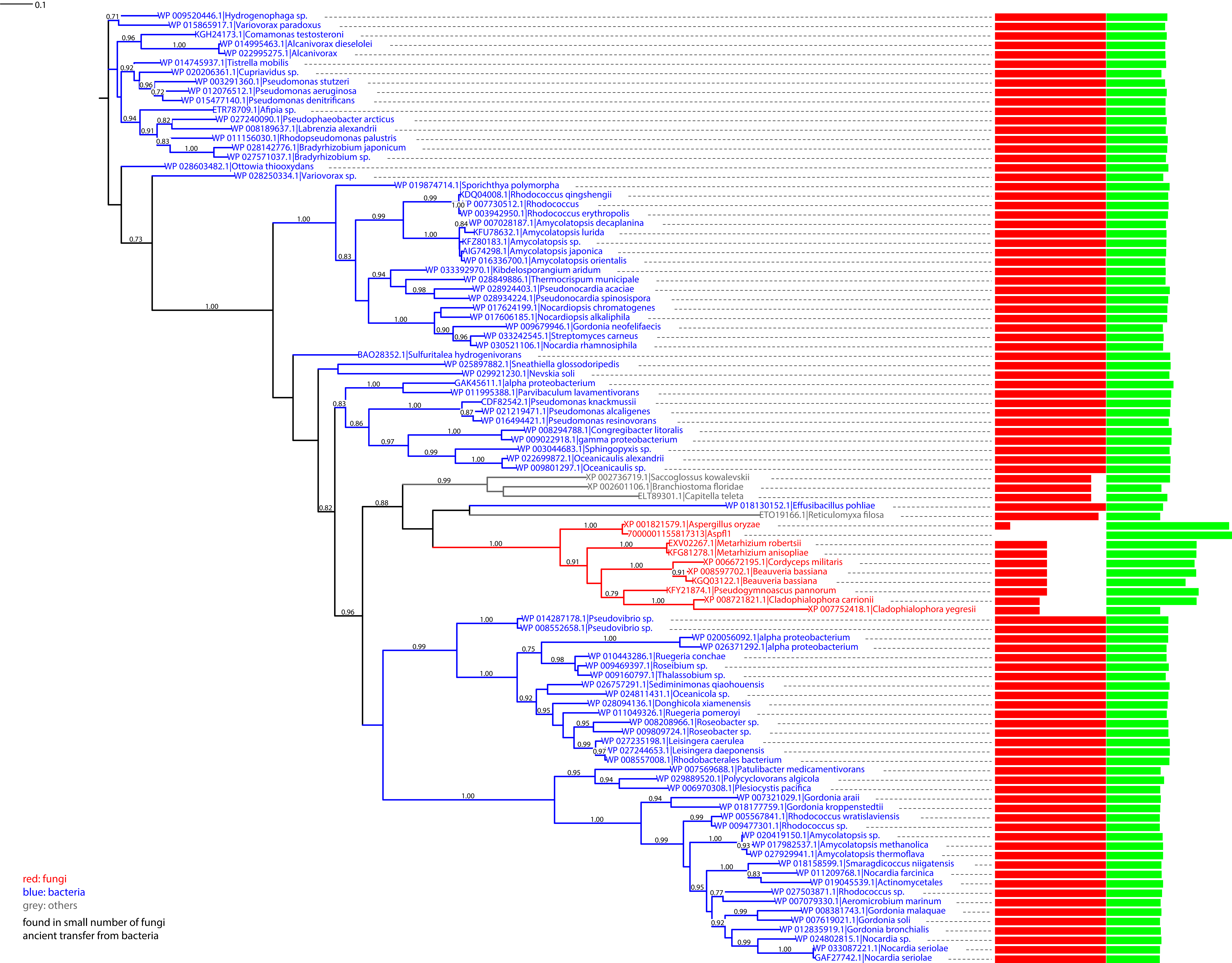
0.1
WP 009520446.1|Hydrogenophaga sp.
WP 015865917.1|Variovorax paradoxus
KGH24173.1|Comamonas testosteroni
WP 014995463.1|Alcanivorax dieselolei
WP 022995275.1|Alcanivorax
WP 014745937.1|Tistrella mobilis
WP 020206361.1|Cupriavidus sp.
WP 003291360.1|Pseudomonas stutzeri
WP 012076512.1|Pseudomonas aeruginosa
WP 015477140.1|Pseudomonas denitrificans
ETR78709.1|Afipia sp.
WP 027240090.1|Pseudophaeobacter arcticus
WP 008189637.1|Labrenzia alexandrii
WP 011156030.1|Rhodopseudomonas palustris
WP 028142776.1|Bradyrhizobium japonicum
WP 027571037.1|Bradyrhizobium sp.
WP 028603482.1|Ottowia thiooxydans
WP 028250334.1|Variovorax sp.
WP 019874714.1|Sporichthya polymorpha
KDQ04008.1|Rhodococcus qingshengii
P 007730512.1|Rhodococcus
WP 003942950.1|Rhodococcus erythropolis
WP 007028187.1|Amycolatopsis decaplanina
KFU78632.1|Amycolatopsis lurida
KFZ80183.1|Amycolatopsis sp.
AIG74298.1|Amycolatopsis japonica
WP 016336700.1|Amycolatopsis orientalis
WP 033392970.1|Kibdelosporangium aridum
WP 028849886.1|Thermocrispum municipale
WP 028924403.1|Pseudonocardia acaciae
WP 028934224.1|Pseudonocardia spinosispora
WP 017624199.1|Nocardiopsis chromatogenes
WP 017606185.1|Nocardiopsis alkaliphila
WP 009679946.1|Gordonia neofelifaecis
WP 033242545.1|Streptomyces carneus
WP 030521106.1|Nocardia rhamnosiphila
BAO28352.1|Sulfuritalea hydrogenivorans
WP 025897882.1|Sneathiella glossodoripedis
WP 029921230.1|Nevskia soli
GAK45611.1|alpha proteobacterium
WP 011995388.1|Parvibaculum lavamentivorans
CDF82542.1|Pseudomonas knackmussii
WP 021219471.1|Pseudomonas alcaligenes
WP 016494421.1|Pseudomonas resinovorans
WP 008294788.1|Congregibacter litoralis
WP 009022918.1|gamma proteobacterium
WP 003044683.1|Sphingopyxis sp.
WP 022699872.1|Oceanicaulis alexandrii
WP 009801297.1|Oceanicaulis sp.
XP 002736719.1|Saccoglossus kowalevskii
XP 002601106.1|Branchiostoma floridae
ELT89301.1|Capitella teleta
WP 018130152.1|Effusibacillus pohliae
ETO19166.1|Reticulomyxa filosa
XP 001821579.1|Aspergillus oryzae
7000001155817313|AspfI1
EXV02267.1|Metarhizium robertsii
KFG81278.1|Metarhizium anisopliae
XP 006672195.1|Cordyceps militaris
XP 008597702.1|Beauveria bassiana
KGQ03122.1|Beauveria bassiana
KFY21874.1|Pseudogymnoascus pannorum
XP 008721821.1|Cladophialophora carrionii
XP 007752418.1|Cladophialophora yegresii
WP 014287178.1|Pseudovibrio sp.
WP 008552658.1|Pseudovibrio sp.
WP 020056092.1|alpha proteobacterium
WP 026371292.1|alpha proteobacterium
WP 010443286.1|Ruegeria conchae
WP 009469397.1|Roseibium sp.
WP 009160797.1|Thalassobium sp.
WP 026757291.1|Sediminimonas qiaohouensis
WP 024811431.1|Oceanicola sp.
WP 028094136.1|Donghicola xiamenensis
WP 011049326.1|Ruegeria pomeroyi
WP 008208966.1|Roseobacter sp.
WP 009809724.1|Roseobacter sp.
WP 027235198.1|Leisingera caerulea
WP 027244653.1|Leisingera daeponensis
WP 008557008.1|Rhodobacterales bacterium
WP 007569688.1|Patulibacter medicamentivorans
WP 029889520.1|Polycyclovorans algicola
WP 006970308.1|Plesiocystis pacifica
WP 007321029.1|Gordonia araii
WP 018177759.1|Gordonia kroppenstedtii
WP 005567841.1|Rhodococcus wratislaviensis
WP 009477301.1|Rhodococcus sp.
WP 020419150.1|Amycolatopsis sp.
WP 017982537.1|Amycolatopsis methanolica
WP 027929941.1|Amycolatopsis thermoflava
WP 018158599.1|Smaragdicoccus niigatensis
WP 011209768.1|Nocardia farcinica
WP 019045539.1|Actinomycetales
WP 027503871.1|Rhodococcus sp.
WP 007079330.1|Aeromicrobium marinum
WP 008381743.1|Gordonia malaquae
WP 007619021.1|Gordonia soli
WP 012835919.1|Gordonia bronchialis
WP 024802815.1|Nocardia sp.
WP 033087221.1|Nocardia seriolae
GAF27742.1|Nocardia seriolae
red: fungi
blue: bacteria
grey: others
found in small number of fungi
ancient transfer from bacteria

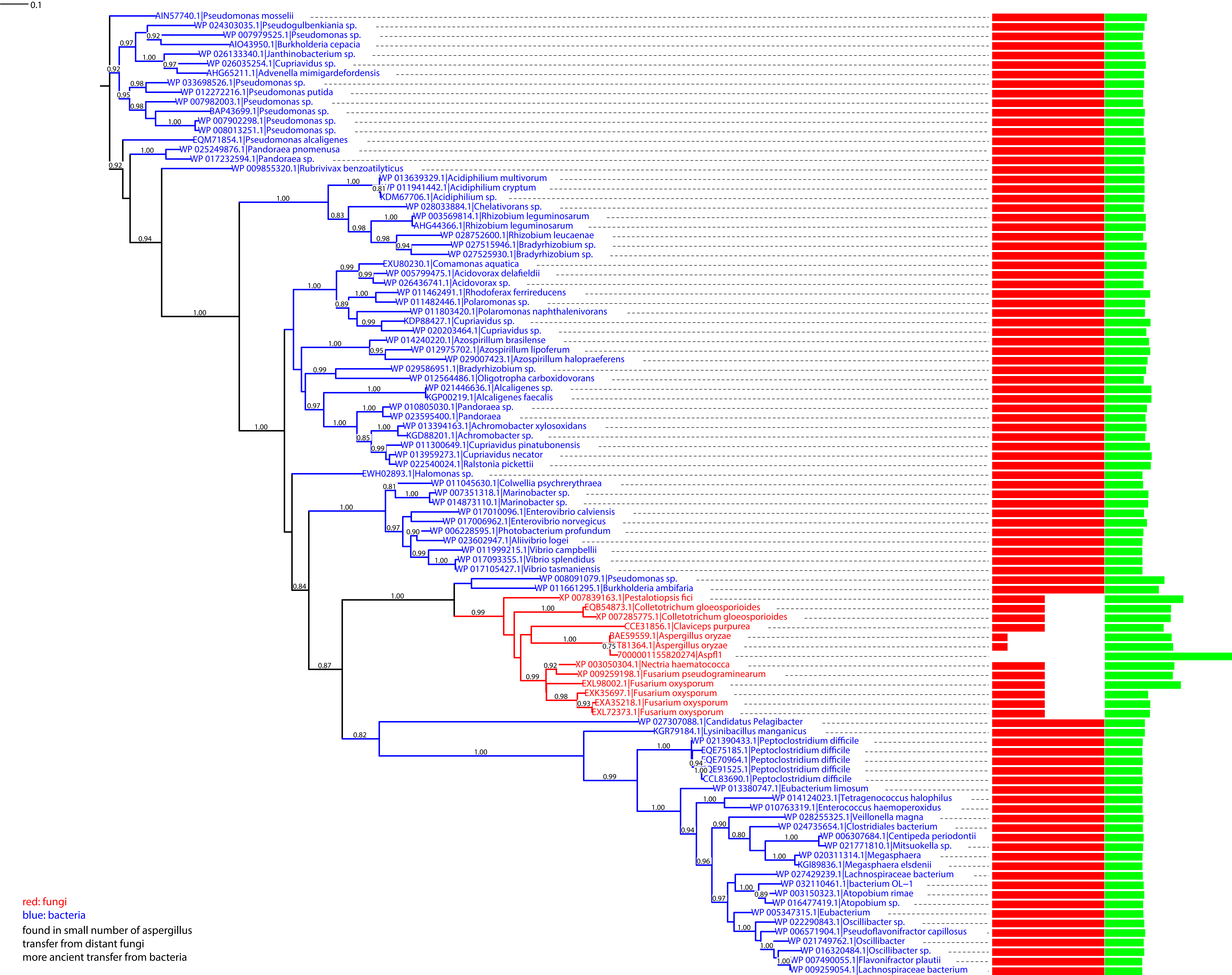

0.1
AIN57740.1|Pseudomonas mosselii
WP 024303035.1|Pseudogulbenkiania sp.
WP 007979525.1|Pseudomonas sp.
AIO43950.1|Burkholderia cepacia
WP 026133340.1|Janthinobacterium sp.
WP 026035254.1|Cupriavidus sp.
AHG65211.1|Advenella mimigardefordensis
WP 033698526.1|Pseudomonas sp.
WP 012272216.1|Pseudomonas putida
WP 007982003.1|Pseudomonas sp.
BAP43699.1|Pseudomonas sp.
WP 007902298.1|Pseudomonas sp.
WP 008013251.1|Pseudomonas sp.
EQM71854.1|Pseudomonas alcaligenes
WP 025249876.1|Pandoraea pnomenusa
WP 017232594.1|Pandoraea sp.
WP 009855320.1|Rubrivivax benzoatilyticus
WP 013639329.1|Acidiphilium multivorum
/P 011941442.1|Acidiphilium cryptum
KDM67706.1|Acidiphilium sp.
WP 028033884.1|Chelativorans sp.
WP 003569814.1|Rhizobium leguminosarum
AHG44366.1|Rhizobium leguminosarum
WP 028752600.1|Rhizobium leucaenae
WP 027515946.1|Bradyrhizobium sp.
WP 027525930.1|Bradyrhizobium sp.
EXU80230.1|Comamonas aquatica
WP 005799475.1|Acidovorax delafieldii
WP 026436741.1|Acidovorax sp.
WP 011462491.1|Rhodoferax ferrireducens
WP 011482446.1|Polaromonas sp.
WP 011803420.1|Polaromonas naphthalenivorans
KDP88427.1|Cupriavidus sp.
WP 020203464.1|Cupriavidus sp.
WP 014240220.1|Azospirillum brasilense
WP 012975702.1|Azospirillum lipoferum
WP 029007423.1|Azospirillum halopraeferens
WP 029586951.1|Bradyrhizobium sp.
WP 012564486.1|Oligotropha carboxidovorans
WP 021446636.1|Alcaligenes sp.
KGP00219.1|Alcaligenes faecalis
WP 010805030.1|Pandoraea sp.
WP 023595400.1|Pandoraea
WP 013394163.1|Achromobacter xylosoxidans
KGD88201.1|Achromobacter sp.
WP 011300649.1|Cupriavidus pinatubonensis
WP 013959273.1|Cupriavidus necator
WP 022540024.1|Ralstonia pickettii
EWH02893.1|Halomonas sp.
WP 011045630.1|Colwellia psychrerythraea
WP 007351318.1|Marinobacter sp.
WP 014873110.1|Marinobacter sp.
WP 017010096.1|Enterovibrio calviensis
WP 017006962.1|Enterovibrio norvegicus
WP 006228595.1|Photobacterium profundum
WP 023602947.1|Aliivibrio logei
WP 011999215.1|Vibrio campbellii
WP 017093355.1|Vibrio splendidus
WP 017105427.1|Vibrio tasmaniensis
WP 008091079.1|Pseudomonas sp.
WP 011661295.1|Burkholderia ambifaria
XP 007839163.1|Pestalotiopsis fici
EQB54873.1|Colletotrichum gloeosporioides
XP 007285775.1|Colletotrichum gloeosporioides
CCE31856.1|Claviceps purpurea
BAE59559.1|Aspergillus oryzae
T81364.1|Aspergillus oryzae
7000001155820274|Aspfl1
XP 003050304.1|Nectria haematococca
XP 009259198.1|Fusarium pseudograminearum
EXL98002.1|Fusarium oxysporum
EXK35697.1|Fusarium oxysporum
EXA35218.1|Fusarium oxysporum
EXL72373.1|Fusarium oxysporum
WP 027307088.1|Candidatus Pelagibacter
KGR79184.1|Lysinibacillus manganicus
WP 021390433.1|Peptoclostridium difficile
EQE75185.1|Peptoclostridium difficile
EQE70964.1|Peptoclostridium difficile
QE91525.1|Peptoclostridium difficile
CCL83690.1|Peptoclostridium difficile
WP 013380747.1|Eubacterium limosum
WP 014124023.1|Tetragenococcus halophilus
WP 010763319.1|Enterococcus haemoperoxidus
WP 028255325.1|Veillonella magna
WP 024735654.1|Clostridiales bacterium
WP 006307684.1|Centipeda periodontii
WP 021771810.1|Mitsuokella sp.
WP 020311314.1|Megasphaera
KGI89836.1|Megasphaera elsdenii
WP 027429239.1|Lachnospiraceae bacterium
WP 032110461.1|bacterium OL−1
WP 003150323.1|Atopobium rimae
WP 016477419.1|Atopobium sp.
WP 005347315.1|Eubacterium
WP 022290843.1|Oscillibacter sp.
WP 006571904.1|Pseudoflavonifractor capillosus
WP 021749762.1|Oscillibacter
WP 016320484.1|Oscillibacter sp.
WP 007490055.1|Flavonifractor plautii
WP 009259054.1|Lachnospiraceae bacterium
red: fungi
blue: bacteria
found in small number of aspergillus
transfer from distant fungi
more ancient transfer from bacteria

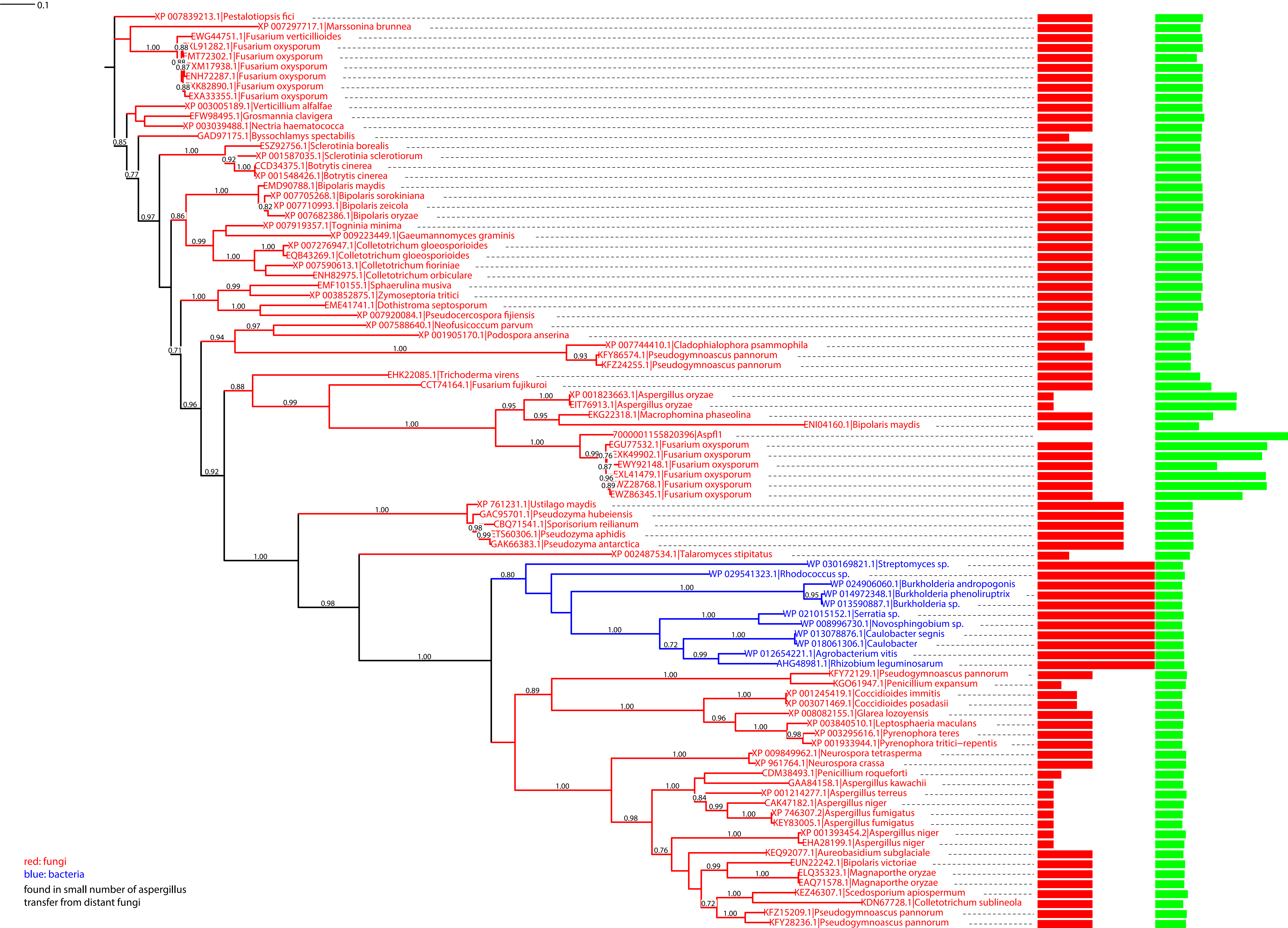
0.1
XP 007839213.1|Pestalotiopsis fici
XP 007297717.1|Marssonina brunnea
EWG44751.1|Fusarium verticillioides
KL91282.1|Fusarium oxysporum
FMT72302.1|Fusarium oxysporum
XM17938.1|Fusarium oxysporum
ENH72287.1|Fusarium oxysporum
XK82890.1|Fusarium oxysporum
EXA33355.1|Fusarium oxysporum
XP 003005189.1|Verticillium alfalfae
EFW98495.1|Grosmannia clavigera
XP 003039488.1|Nectria haematococca
GAD97175.1|Byssochlamys spectabilis
ESZ92756.1|Sclerotinia borealis
XP 001587035.1|Sclerotinia sclerotiorum
CCD34375.1|Botrytis cinerea
XP 001548426.1|Botrytis cinerea
EMD90788.1|Bipolaris maydis
XP 007705268.1|Bipolaris sorokiniana
XP 007710993.1|Bipolaris zeicola
XP 007682386.1|Bipolaris oryzae
XP 007919357.1|Togninia minima
XP 009223449.1|Gaeumannomyces graminis
XP 007276947.1|Colletotrichum gloeosporioides
EQB43269.1|Colletotrichum gloeosporioides
XP 007590613.1|Colletotrichum fioriniae
ENH82975.1|Colletotrichum orbiculare
EMF10155.1|Sphaerulina musiva
XP 003852875.1|Zymoseptoria tritici
EME41741.1|Dothistroma septosporum
XP 007920084.1|Pseudocercospora fijiensis
XP 007588640.1|Neofusicoccum parvum
XP 001905170.1|Podospora anserina
XP 007744410.1|Cladophialophora psammophila
KFY86574.1|Pseudogymnoascus pannorum
KFZ24255.1|Pseudogymnoascus pannorum
EHK22085.1|Trichoderma virens
CCT74164.1|Fusarium fujikuroi
XP 001823663.1|Aspergillus oryzae
EIT76913.1|Aspergillus oryzae
EKG22318.1|Macrophomina phaseolina
ENI04160.1|Bipolaris maydis
7000001155820396|Aspfl1
EGU77532.1|Fusarium oxysporum
XK49902.1|Fusarium oxysporum
EWY92148.1|Fusarium oxysporum
XL41479.1|Fusarium oxysporum
WZ28768.1|Fusarium oxysporum
EWZ86345.1|Fusarium oxysporum
XP 761231.1|Ustilago maydis
GAC95701.1|Pseudozyma hubeiensis
CBQ71541.1|Sporisorium reilianum
TS60306.1|Pseudozyma aphidis
GAK66383.1|Pseudozyma antarctica
XP 002487534.1|Talaromyces stipitatus
WP 030169821.1|Streptomyces sp.
WP 029541323.1|Rhodococcus sp.
WP 024906060.1|Burkholderia andropogonis
WP 014972348.1|Burkholderia phenoliruptrix
WP 013590887.1|Burkholderia sp.
WP 021015152.1|Serratia sp.
WP 008996730.1|Novosphingobium sp.
WP 013078876.1|Caulobacter segnis
WP 018061306.1|Caulobacter
WP 012654221.1|Agrobacterium vitis
AHG48981.1|Rhizobium leguminosarum
KFY72129.1|Pseudogymnoascus pannorum
KGO61947.1|Penicillium expansum
XP 001245419.1|Coccidioides immitis
XP 003071469.1|Coccidioides posadasii
XP 008082155.1|Glarea lozoyensis
XP 003840510.1|Leptosphaeria maculans
XP 003295616.1|Pyrenophora teres
XP 001933944.1|Pyrenophora tritici−repentis
XP 009849962.1|Neurospora tetrasperma
XP 961764.1|Neurospora crassa
CDM38493.1|Penicillium roqueforti
GAA84158.1|Aspergillus kawachii
XP 001214277.1|Aspergillus terreus
CAK47182.1|Aspergillus niger
XP 746307.2|Aspergillus fumigatus
KEY83005.1|Aspergillus fumigatus
XP 001393454.2|Aspergillus niger
EHA28199.1|Aspergillus niger
KEQ92077.1|Aureobasidium subglaciale
EUN22242.1|Bipolaris victoriae
ELQ35323.1|Magnaporthe oryzae
EAQ71578.1|Magnaporthe oryzae
KEZ46307.1|Scedosporium apiospermum
KDN67728.1|Colletotrichum sublineola
KFZ15209.1|Pseudogymnoascus pannorum
KFY28236.1|Pseudogymnoascus pannorum
red: fungi
blue: bacteria
found in small number of aspergillus
transfer from distant fungi

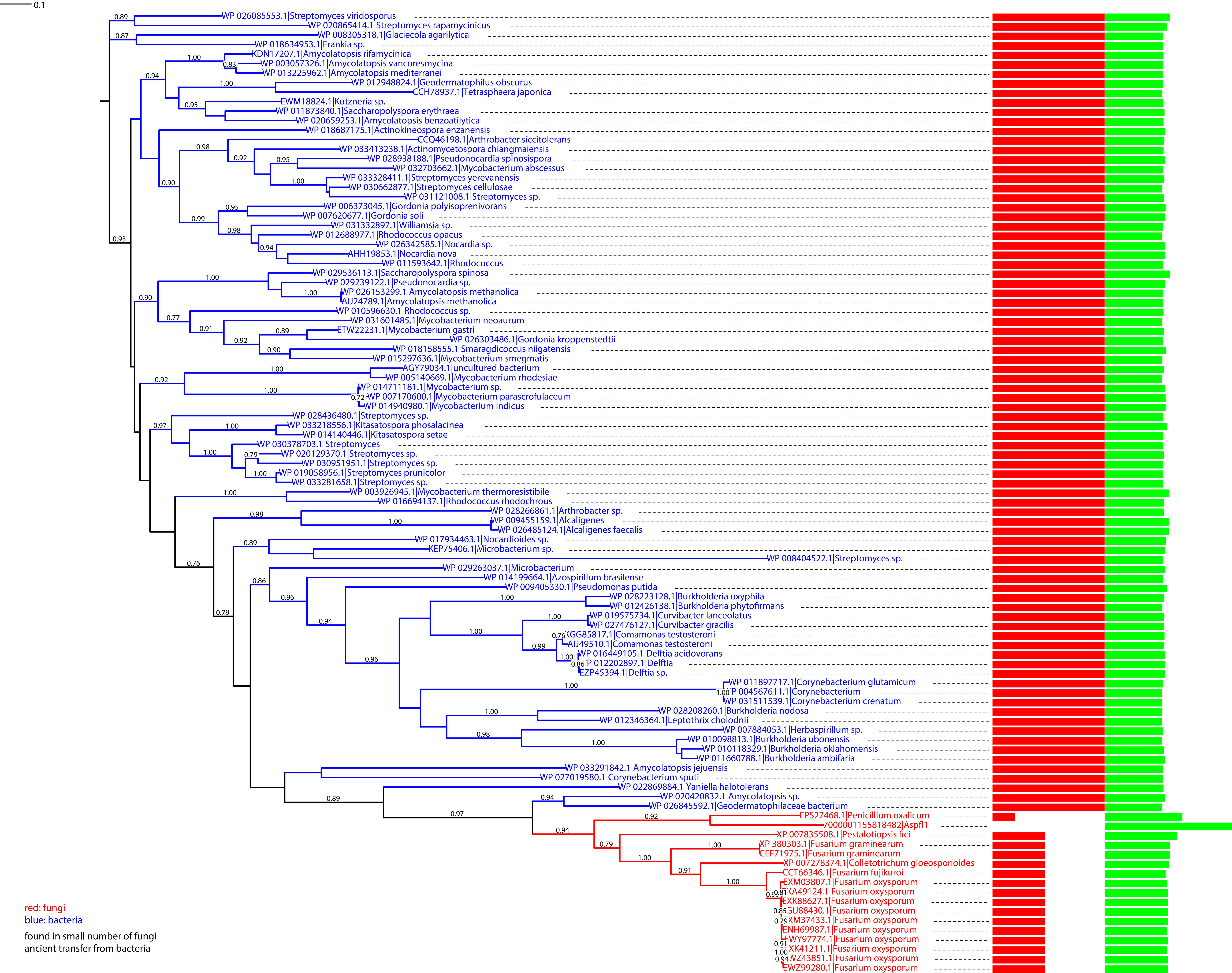

0.1
WP 026085553.1|Streptomyces viridosporus
WP 020865414.1|Streptomyces rapamycinicus
WP 008305318.1|Glaciecola agarilytica
WP 018634953.1|Frankia sp.
KDN17207.1|Amycolatopsis rifamycinica
WP 003057326.1|Amycolatopsis vancoresmycina
WP 013225962.1|Amycolatopsis mediterranei
WP 012948824.1|Geodermatophilus obscurus
CCH78937.1|Tetrasphaera japonica
EWM18824.1|Kutzneria sp.
WP 011873840.1|Saccharopolyspora erythraea
WP 020659253.1|Amycolatopsis benzoatilytica
WP 018687175.1|Actinokineospora enzanensis
CCQ46198.1|Arthrobacter siccitolerans
WP 033413238.1|Actinomycetospora chiangmaiensis
WP 028938188.1|Pseudonocardia spinosispora
WP 032703662.1|Mycobacterium abscessus
WP 033328411.1|Streptomyces yerevanensis
WP 030662877.1|Streptomyces cellulosae
WP 031121008.1|Streptomyces sp.
WP 006373045.1|Gordonia polyisoprenivorans
WP 007620677.1|Gordonia soli
WP 031332897.1|Williamsia sp.
WP 012688977.1|Rhodococcus opacus
WP 026342585.1|Nocardia sp.
AHH19853.1|Nocardia nova
WP 011593642.1|Rhodococcus
WP 029536113.1|Saccharopolyspora spinosa
WP 029239122.1|Pseudonocardia sp.
WP 026153299.1|Amycolatopsis methanolica
AIJ24789.1|Amycolatopsis methanolica
WP 010596630.1|Rhodococcus sp.
WP 031601485.1|Mycobacterium neoaurum
ETW22231.1|Mycobacterium gastri
WP 026303486.1|Gordonia kroppenstedtii
WP 018158555.1|Smaragdicoccus niigatensis
WP 015297636.1|Mycobacterium smegmatis
AGY79034.1|uncultured bacterium
WP 005140669.1|Mycobacterium rhodesiae
WP 014711181.1|Mycobacterium sp.
WP 007170600.1|Mycobacterium parascrofulaceum
WP 014940980.1|Mycobacterium indicus
WP 028436480.1|Streptomyces sp.
WP 033218556.1|Kitasatospora phosalacinea
WP 014140446.1|Kitasatospora setae
WP 030378703.1|Streptomyces
WP 020129370.1|Streptomyces sp.
WP 030951951.1|Streptomyces sp.
WP 019058956.1|Streptomyces prunicolor
WP 033281658.1|Streptomyces sp.
WP 003926945.1|Mycobacterium thermoresistibile
WP 016694137.1|Rhodococcus rhodochrous
WP 028266861.1|Arthrobacter sp.
WP 009455159.1|Alcaligenes
WP 026485124.1|Alcaligenes faecalis
WP 017934463.1|Nocardioides sp.
KEP75406.1|Microbacterium sp.
WP 008404522.1|Streptomyces sp.
WP 029263037.1|Microbacterium
WP 014199664.1|Azospirillum brasilense
WP 009405330.1|Pseudomonas putida
WP 028223128.1|Burkholderia oxyphila
WP 012426138.1|Burkholderia phytofirmans
WP 019575734.1|Curvibacter lanceolatus
WP 027476127.1|Curvibacter gracilis
GG85817.1|Comamonas testosteroni
AIJ49510.1|Comamonas testosteroni
WP 016449105.1|Delftia acidovorans
P 012202897.1|Delftia
EZP45394.1|Delftia sp.
WP 011897717.1|Corynebacterium glutamicum
P 004567611.1|Corynebacterium
WP 031511539.1|Corynebacterium crenatum
WP 028208260.1|Burkholderia nodosa
WP 012346364.1|Leptothrix cholodnii
WP 007884053.1|Herbaspirillum sp.
WP 010098813.1|Burkholderia ubonensis
WP 010118329.1|Burkholderia oklahomensis
WP 011660788.1|Burkholderia ambifaria
WP 033291842.1|Amycolatopsis jejuensis
WP 027019580.1|Corynebacterium sputi
WP 022869884.1|Yaniella halotolerans
WP 020420832.1|Amycolatopsis sp.
WP 026845592.1|Geodermatophilaceae bacterium
EPS27468.1|Penicillium oxalicum
7000001155818482|Aspfl1
XP 007835508.1|Pestalotiopsis fici
XP 380303.1|Fusarium graminearum
CEF71975.1|Fusarium graminearum
XP 007278374.1|Colletotrichum gloeosporioides
CCT66346.1|Fusarium fujikuroi
EXM03807.1|Fusarium oxysporum
A49124.1|Fusarium oxysporum
EXK88627.1|Fusarium oxysporum
GU88430.1|Fusarium oxysporum
XM37433.1|Fusarium oxysporum
ENH69987.1|Fusarium oxysporum
FWY97774.1|Fusarium oxysporum
XK41211.1|Fusarium oxysporum
NZ43851.1|Fusarium oxysporum
EWZ99280.1|Fusarium oxysporum
red: fungi
blue: bacteria
found in small number of fungi
ancient transfer from bacteria

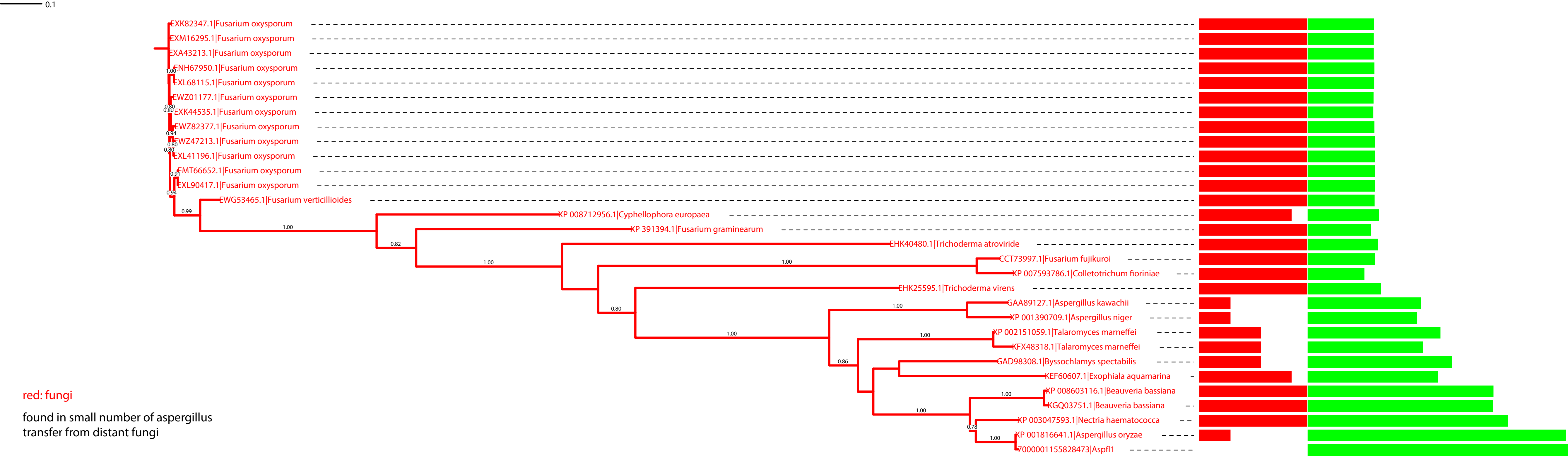

0.1
EXK82347.1|Fusarium oxysporum
EXM16295.1|Fusarium oxysporum
EXA43213.1|Fusarium oxysporum
FNH67950.1|Fusarium oxysporum
EXL68115.1|Fusarium oxysporum
EWZ01177.1|Fusarium oxysporum
EXK44535.1|Fusarium oxysporum
EWZ82377.1|Fusarium oxysporum
EWZ47213.1|Fusarium oxysporum
EXL41196.1|Fusarium oxysporum
FMT66652.1|Fusarium oxysporum
EXL90417.1|Fusarium oxysporum
EWG53465.1|Fusarium verticillioides
XP 008712956.1|Cyphellophora europaea
XP 391394.1|Fusarium graminearum
EHK40480.1|Trichoderma atroviride
CCT73997.1|Fusarium fujikuroi
XP 007593786.1|Colletotrichum fioriniae
EHK25595.1|Trichoderma virens
GAA89127.1|Aspergillus kawachii
XP 001390709.1|Aspergillus niger
XP 002151059.1|Talaromyces marneffei
KFX48318.1|Talaromyces marneffei
GAD98308.1|Byssochlamys spectabilis
KEF60607.1|Exophiala aquamarina
XP 008603116.1|Beauveria bassiana
KGQ03751.1|Beauveria bassiana
XP 003047593.1|Nectria haematococca
XP 001816641.1|Aspergillus oryzae
7000001155828473|Aspfl1
red: fungi
found in small number of aspergillus
transfer from distant fungi

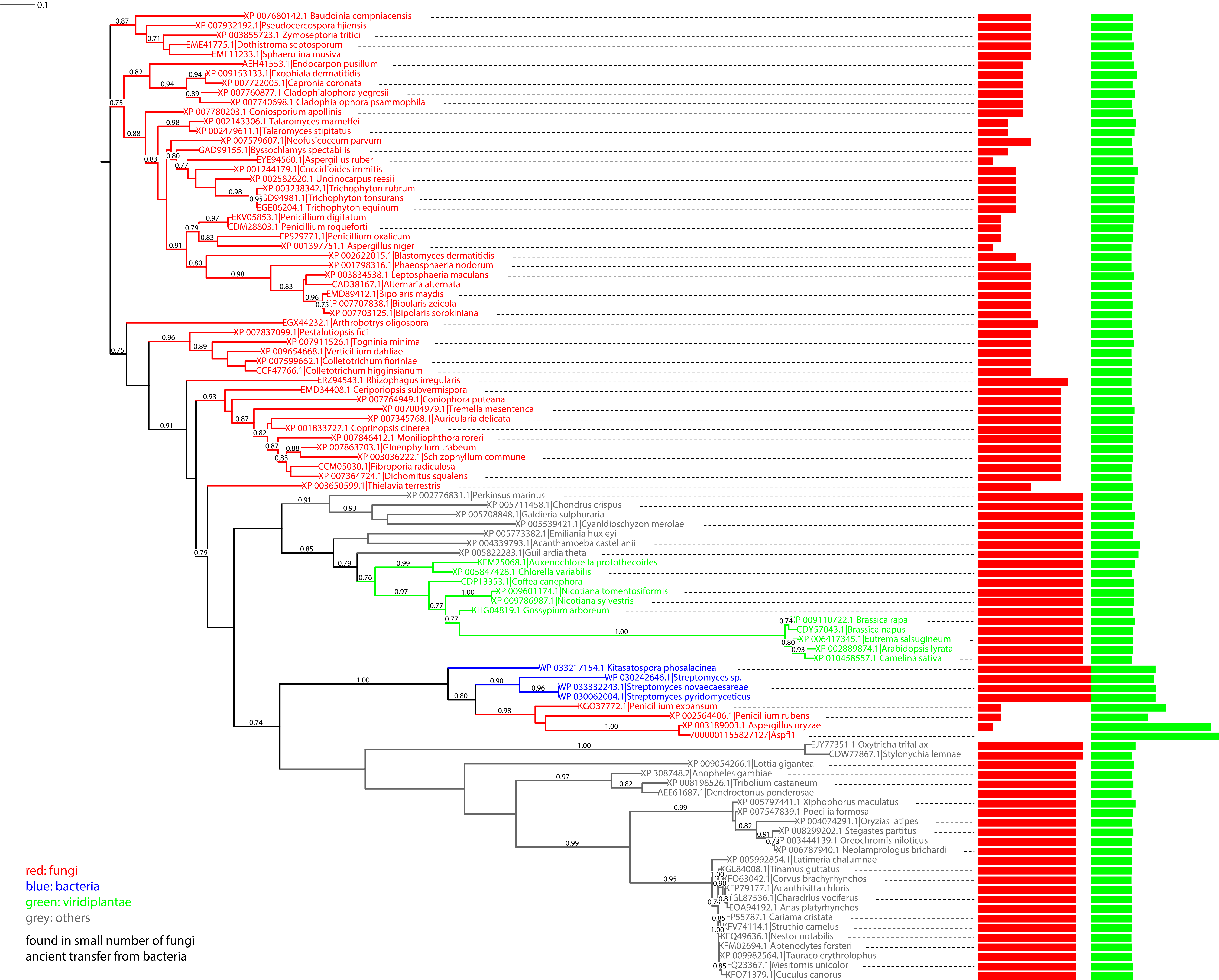
0.1
XP 007680142.1|Baudoinia compniacensis
XP 007932192.1|Pseudocercospora fijiensis
XP 003855723.1|Zymoseptoria tritici
EME41775.1|Dothistroma septosporum
EMF11233.1|Sphaerulina musiva
AEH41553.1|Endocarpon pusillum
XP 009153133.1|Exophiala dermatitidis
XP 007722005.1|Capronia coronata
XP 007760877.1|Cladophialophora yegresii
XP 007740698.1|Cladophialophora psammophila
XP 007780203.1|Coniosporium apollinis
XP 002143306.1|Talaromyces marneffei
XP 002479611.1|Talaromyces stipitatus
XP 007579607.1|Neofusicoccum parvum
GAD99155.1|Byssochlamys spectabilis
EYE94560.1|Aspergillus ruber
XP 001244179.1|Coccidioides immitis
XP 002582620.1|Uncinocarpus reesii
XP 003238342.1|Trichophyton rubrum
GD94981.1|Trichophyton tonsurans
EGE06204.1|Trichophyton equinum
EKV05853.1|Penicillium digitatum
CDM28803.1|Penicillium roqueforti
EPS29771.1|Penicillium oxalicum
XP 001397751.1|Aspergillus niger
XP 002622015.1|Blastomyces dermatitidis
XP 001798316.1|Phaeosphaeria nodorum
XP 003834538.1|Leptosphaeria maculans
CAD38167.1|Alternaria alternata
EMD89412.1|Bipolaris maydis
P 007707838.1|Bipolaris zeicola
XP 007703125.1|Bipolaris sorokiniana
EGX44232.1|Arthrobotrys oligospora
XP 007837099.1|Pestalotiopsis fici
XP 007911526.1|Togninia minima
XP 009654668.1|Verticillium dahliae
XP 007599662.1|Colletotrichum fioriniae
CCF47766.1|Colletotrichum higginsianum
ERZ94543.1|Rhizophagus irregularis
EMD34408.1|Ceriporiopsis subvermispora
XP 007764949.1|Coniophora puteana
XP 007004979.1|Tremella mesenterica
XP 007345768.1|Auricularia delicata
XP 001833727.1|Coprinopsis cinerea
XP 007846412.1|Moniliophthora roreri
XP 007863703.1|Gloeophyllum trabeum
XP 003036222.1|Schizophyllum commune
CCM05030.1|Fibroporia radiculosa
XP 007364724.1|Dichomitus squalens
XP 003650599.1|Thielavia terrestris
XP 002776831.1|Perkinsus marinus
XP 005711458.1|Chondrus crispus
XP 005708848.1|Galdieria sulphuraria
XP 005539421.1|Cyanidioschyzon merolae
XP 005773382.1|Emiliania huxleyi
XP 004339793.1|Acanthamoeba castellanii
XP 005822283.1|Guillardia theta
KFM25068.1|Auxenochlorella protothecoides
XP 005847428.1|Chlorella variabilis
CDP13353.1|Coffea canephora
XP 009601174.1|Nicotiana tomentosiformis
XP 009786987.1|Nicotiana sylvestris
KHG04819.1|Gossypium arboreum
P 009110722.1|Brassica rapa
CDY57043.1|Brassica napus
XP 006417345.1|Eutrema salsugineum
XP 002889874.1|Arabidopsis lyrata
XP 010458557.1|Camelina sativa
WP 033217154.1|Kitasatospora phosalacinea
WP 030242646.1|Streptomyces sp.
WP 033332243.1|Streptomyces novaecaesareae
WP 030062004.1|Streptomyces pyridomyceticus
KGO37772.1|Penicillium expansum
XP 002564406.1|Penicillium rubens
XP 003189003.1|Aspergillus oryzae
7000001155827127|Aspfl1
EJY77351.1|Oxytricha trifallax
CDW77867.1|Stylonychia lemnae
XP 009054266.1|Lottia gigantea
XP 308748.2|Anopheles gambiae
XP 008198526.1|Tribolium castaneum
AEE61687.1|Dendroctonus ponderosae
XP 005797441.1|Xiphophorus maculatus
XP 007547839.1|Poecilia formosa
XP 004074291.1|Oryzias latipes
XP 008299202.1|Stegastes partitus
P 003444139.1|Oreochromis niloticus
XP 006787940.1|Neolamprologus brichardi
XP 005992854.1|Latimeria chalumnae
KGL84008.1|Tinamus guttatus
KFO63042.1|Corvus brachyrhynchos
KFP79177.1|Acanthisitta chloris
GL87536.1|Charadrius vociferus
EOA94192.1|Anas platyrhynchos
P55787.1|Cariama cristata
KFV74114.1|Struthio camelus
KFQ49636.1|Nestor notabilis
KFM02694.1|Aptenodytes forsteri
XP 009982564.1|Tauraco erythrolophus
Q23367.1|Mesitornis unicolor
KFO71379.1|Cuculus canorus
red: fungi
blue: bacteria
green: viridiplantae
grey: others
found in small number of fungi
ancient transfer from bacteria

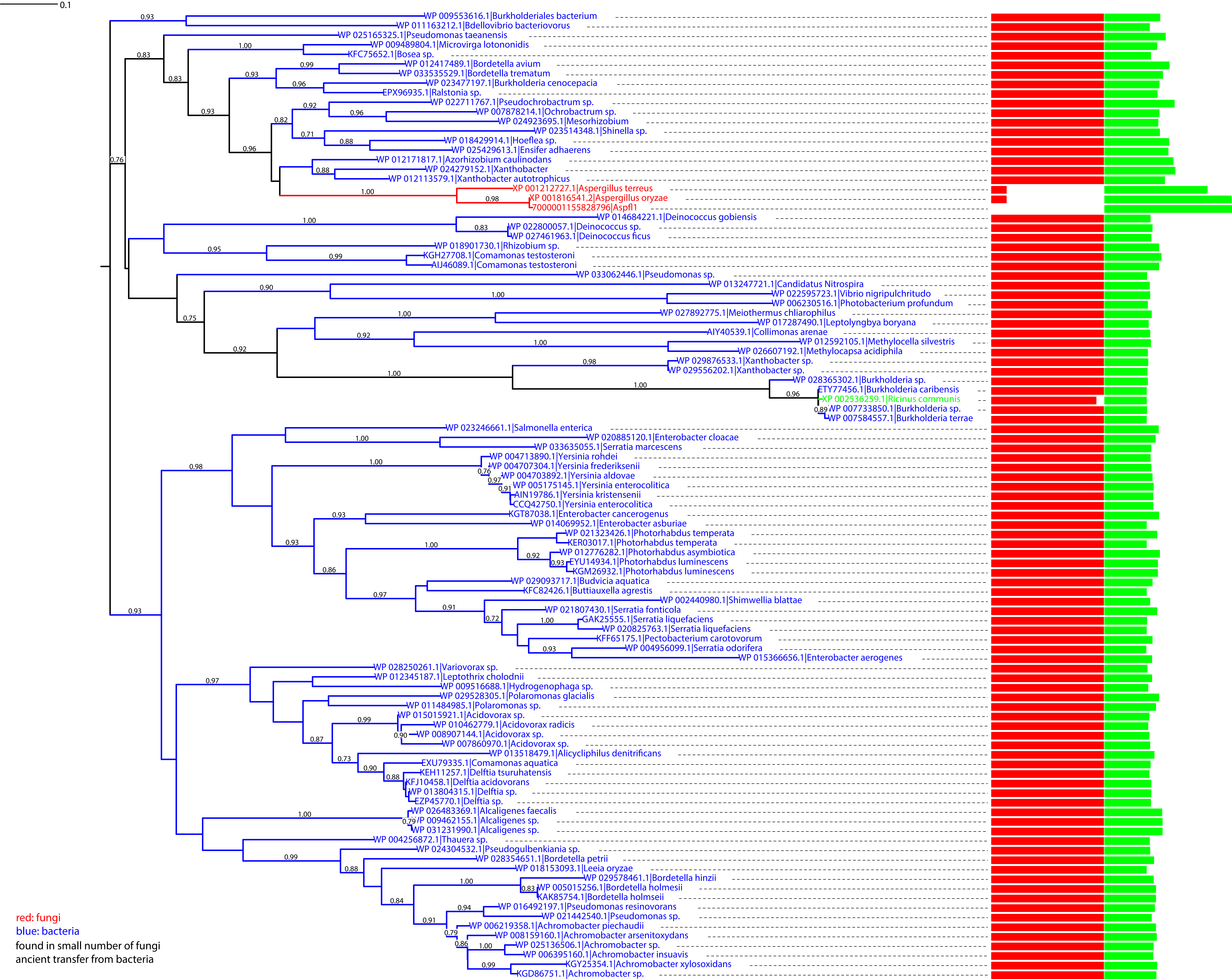

0.1
WP 009553616.1|Burkholderiales bacterium
WP 011163212.1|Bdellovibrio bacteriovorus
WP 025165325.1|Pseudomonas taeanensis
WP 009489804.1|Microvirga lotononidis
KFC75652.1|Bosea sp.
WP 012417489.1|Bordetella avium
WP 033535529.1|Bordetella trematum
WP 023477197.1|Burkholderia cenocepacia
EPX96935.1|Ralstonia sp.
WP 022711767.1|Pseudochrobactrum sp.
WP 007878214.1|Ochrobactrum sp.
WP 024923695.1|Mesorhizobium
WP 023514348.1|Shinella sp.
WP 018429914.1|Hoeflea sp.
WP 025429613.1|Ensifer adhaerens
WP 012171817.1|Azorhizobium caulinodans
WP 024279152.1|Xanthobacter
WP 012113579.1|Xanthobacter autotrophicus
XP 001212727.1|Aspergillus terreus
XP 001816541.2|Aspergillus oryzae
7000001155828796|Aspfl1
WP 014684221.1|Deinococcus gobiensis
WP 022800057.1|Deinococcus sp.
WP 027461963.1|Deinococcus ficus
WP 018901730.1|Rhizobium sp.
KGH27708.1|Comamonas testosteroni
AIJ46089.1|Comamonas testosteroni
WP 033062446.1|Pseudomonas sp.
WP 013247721.1|Candidatus Nitrospira
WP 022595723.1|Vibrio nigripulchritudo
WP 006230516.1|Photobacterium profundum
WP 027892775.1|Meiothermus chliarophilus
WP 017287490.1|Leptolyngbya boryana
AIY40539.1|Collimonas arenae
WP 012592105.1|Methylocella silvestris
WP 026607192.1|Methylocapsa acidiphila
WP 029876533.1|Xanthobacter sp.
WP 029556202.1|Xanthobacter sp.
WP 028365302.1|Burkholderia sp.
ETY77456.1|Burkholderia caribensis
XP 002536259.1|Ricinus communis
WP 007733850.1|Burkholderia sp.
WP 007584557.1|Burkholderia terrae
WP 023246661.1|Salmonella enterica
WP 020885120.1|Enterobacter cloacae
WP 033635055.1|Serratia marcescens
WP 004713890.1|Yersinia rohdei
WP 004707304.1|Yersinia frederiksenii
WP 004703892.1|Yersinia aldovae
WP 005175145.1|Yersinia enterocolitica
AIN19786.1|Yersinia kristensenii
CCQ42750.1|Yersinia enterocolitica
KGT87038.1|Enterobacter cancerogenus
WP 014069952.1|Enterobacter asburiae
WP 021323426.1|Photorhabdus temperata
KER03017.1|Photorhabdus temperata
WP 012776282.1|Photorhabdus asymbiotica
EYU14934.1|Photorhabdus luminescens
KGM26932.1|Photorhabdus luminescens
WP 029093717.1|Budvicia aquatica
KFC82426.1|Buttiauxella agrestis
WP 002440980.1|Shimwellia blattae
WP 021807430.1|Serratia fonticola
GAK25555.1|Serratia liquefaciens
WP 020825763.1|Serratia liquefaciens
KFF65175.1|Pectobacterium carotovorum
WP 004956099.1|Serratia odorifera
WP 015366656.1|Enterobacter aerogenes
WP 028250261.1|Variovorax sp.
WP 012345187.1|Leptothrix cholodnii
WP 009516688.1|Hydrogenophaga sp.
WP 029528305.1|Polaromonas glacialis
WP 011484985.1|Polaromonas sp.
WP 015015921.1|Acidovorax sp.
WP 010462779.1|Acidovorax radicis
WP 008907144.1|Acidovorax sp.
WP 007860970.1|Acidovorax sp.
WP 013518479.1|Alicycliphilus denitrificans
EXU79335.1|Comamonas aquatica
KEH11257.1|Delftia tsuruhatensis
KFJ10458.1|Delftia acidovorans
WP 013804315.1|Delftia sp.
EZP45770.1|Delftia sp.
WP 026483369.1|Alcaligenes faecalis
WP 009462155.1|Alcaligenes sp.
WP 031231990.1|Alcaligenes sp.
WP 004256872.1|Thauera sp.
WP 024304532.1|Pseudogulbenkiania sp.
WP 028354651.1|Bordetella petrii
WP 018153093.1|Leeia oryzae
WP 029578461.1|Bordetella hinzii
WP 005015256.1|Bordetella holmesii
KAK85754.1|Bordetella holmseii
WP 016492197.1|Pseudomonas resinovorans
WP 021442540.1|Pseudomonas sp.
WP 006219358.1|Achromobacter piechaudii
WP 008159160.1|Achromobacter arsenitoxydans
WP 025136506.1|Achromobacter sp.
WP 006395160.1|Achromobacter insuavis
KGY25354.1|Achromobacter xylosoxidans
KGD86751.1|Achromobacter sp.
red: fungi
blue: bacteria
found in small number of fungi
ancient transfer from bacteria

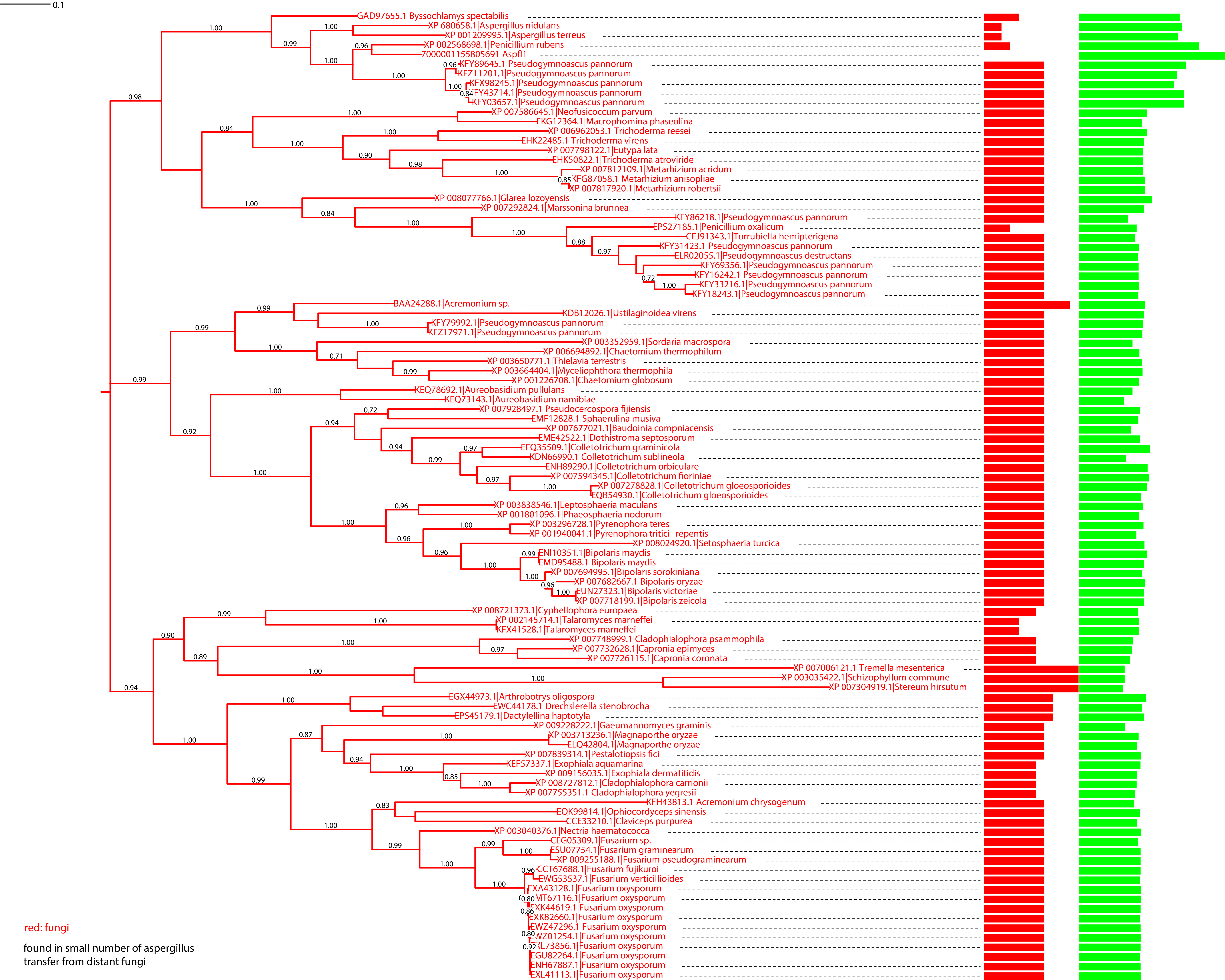

red: fungi

found in small number of aspergillus
transfer from distant fungi

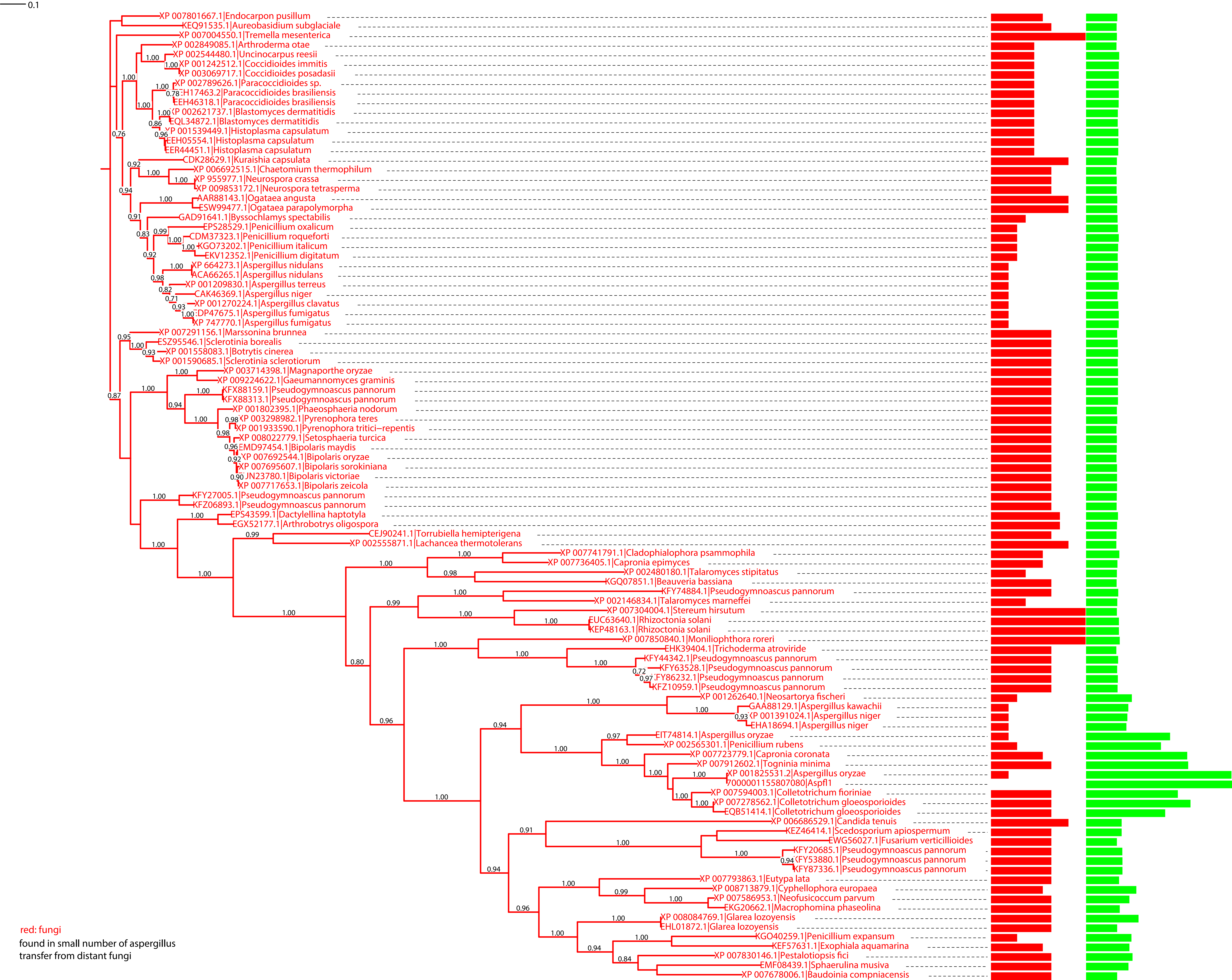
0.1
XP 007801667.1|Endocarpon pusillum
KEQ91535.1|Aureobasidium subglaciale
XP 007004550.1|Tremella mesenterica
XP 002849085.1|Arthroderma otae
XP 002544480.1|Uncinocarpus reesii
XP 001242512.1|Coccidioides immitis
XP 003069717.1|Coccidioides posadasii
XP 002789626.1|Paracoccidioides sp.
EEH17463.2|Paracoccidioides brasiliensis
EEH46318.1|Paracoccidioides brasiliensis
XP 002621737.1|Blastomyces dermatitidis
EQL34872.1|Blastomyces dermatitidis
XP 001539449.1|Histoplasma capsulatum
EEH05554.1|Histoplasma capsulatum
EER44451.1|Histoplasma capsulatum
CDK28629.1|Kuraishia capsulata
XP 006692515.1|Chaetomium thermophilum
XP 955977.1|Neurospora crassa
XP 009853172.1|Neurospora tetrasperma
AAR88143.1|Ogataea angusta
ESW99477.1|Ogataea parapolymorpha
GAD91641.1|Byssochlamys spectabilis
EPS28529.1|Penicillium oxalicum
CDM37323.1|Penicillium roqueforti
KGO73202.1|Penicillium italicum
EKV12352.1|Penicillium digitatum
XP 664273.1|Aspergillus nidulans
ACA66265.1|Aspergillus nidulans
XP 001209830.1|Aspergillus terreus
CAK46369.1|Aspergillus niger
XP 001270224.1|Aspergillus clavatus
EDP47675.1|Aspergillus fumigatus
XP 747770.1|Aspergillus fumigatus
XP 007291156.1|Marssonina brunnea
ESZ95546.1|Sclerotinia borealis
XP 001558083.1|Botrytis cinerea
XP 001590685.1|Sclerotinia sclerotiorum
XP 003714398.1|Magnaporthe oryzae
XP 009224622.1|Gaeumannomyces graminis
KFX88159.1|Pseudogymnoascus pannorum
KFX88313.1|Pseudogymnoascus pannorum
XP 001802395.1|Phaeosphaeria nodorum
XP 003298982.1|Pyrenophora teres
XP 001933590.1|Pyrenophora tritici−repentis
XP 008022779.1|Setosphaeria turcica
EMD97454.1|Bipolaris maydis
XP 007692544.1|Bipolaris oryzae
XP 007695607.1|Bipolaris sorokiniana
EUN23780.1|Bipolaris victoriae
XP 007717653.1|Bipolaris zeicola
KFY27005.1|Pseudogymnoascus pannorum
KFZ06893.1|Pseudogymnoascus pannorum
EPS43599.1|Dactylellina haptotyla
EGX52177.1|Arthrobotrys oligospora
CEJ90241.1|Torrubiella hemipterigena
XP 002555871.1|Lachancea thermotolerans
XP 007741791.1|Cladophialophora psammophila
XP 007736405.1|Capronia epimyces
XP 002480180.1|Talaromyces stipitatus
KGQ07851.1|Beauveria bassiana
KFY74884.1|Pseudogymnoascus pannorum
XP 002146834.1|Talaromyces marneffei
XP 007304004.1|Stereum hirsutum
EUC63640.1|Rhizoctonia solani
KEP48163.1|Rhizoctonia solani
XP 007850840.1|Moniliophthora roreri
EHK39404.1|Trichoderma atroviride
KFY44342.1|Pseudogymnoascus pannorum
KFY63528.1|Pseudogymnoascus pannorum
KFY86232.1|Pseudogymnoascus pannorum
KFZ10959.1|Pseudogymnoascus pannorum
XP 001262640.1|Neosartorya fischeri
GAA88129.1|Aspergillus kawachii
XP 001391024.1|Aspergillus niger
EHA18694.1|Aspergillus niger
EIT74814.1|Aspergillus oryzae
XP 002565301.1|Penicillium rubens
XP 007723779.1|Capronia coronata
XP 007912602.1|Togninia minima
XP 001825531.2|Aspergillus oryzae
7000001155807080|Aspfl1
XP 007594003.1|Colletotrichum fioriniae
XP 007278562.1|Colletotrichum gloeosporioides
EQB51414.1|Colletotrichum gloeosporioides
XP 006686529.1|Candida tenuis
KEZ46414.1|Scedosporium apiospermum
EWG56027.1|Fusarium verticillioides
KFY20685.1|Pseudogymnoascus pannorum
KFY53880.1|Pseudogymnoascus pannorum
KFY87336.1|Pseudogymnoascus pannorum
XP 007793863.1|Eutypa lata
XP 008713879.1|Cyphellophora europaea
XP 007586953.1|Neofusicoccum parvum
EKG20662.1|Macrophomina phaseolina
XP 008084769.1|Glarea lozoyensis
EHL01872.1|Glarea lozoyensis
KGO40259.1|Penicillium expansum
KEF57631.1|Exophiala aquamarina
XP 007830146.1|Pestalotiopsis fici
EMF08439.1|Sphaerulina musiva
XP 007678006.1|Baudoinia compniacensis
red: fungi
found in small number of aspergillus
transfer from distant fungi

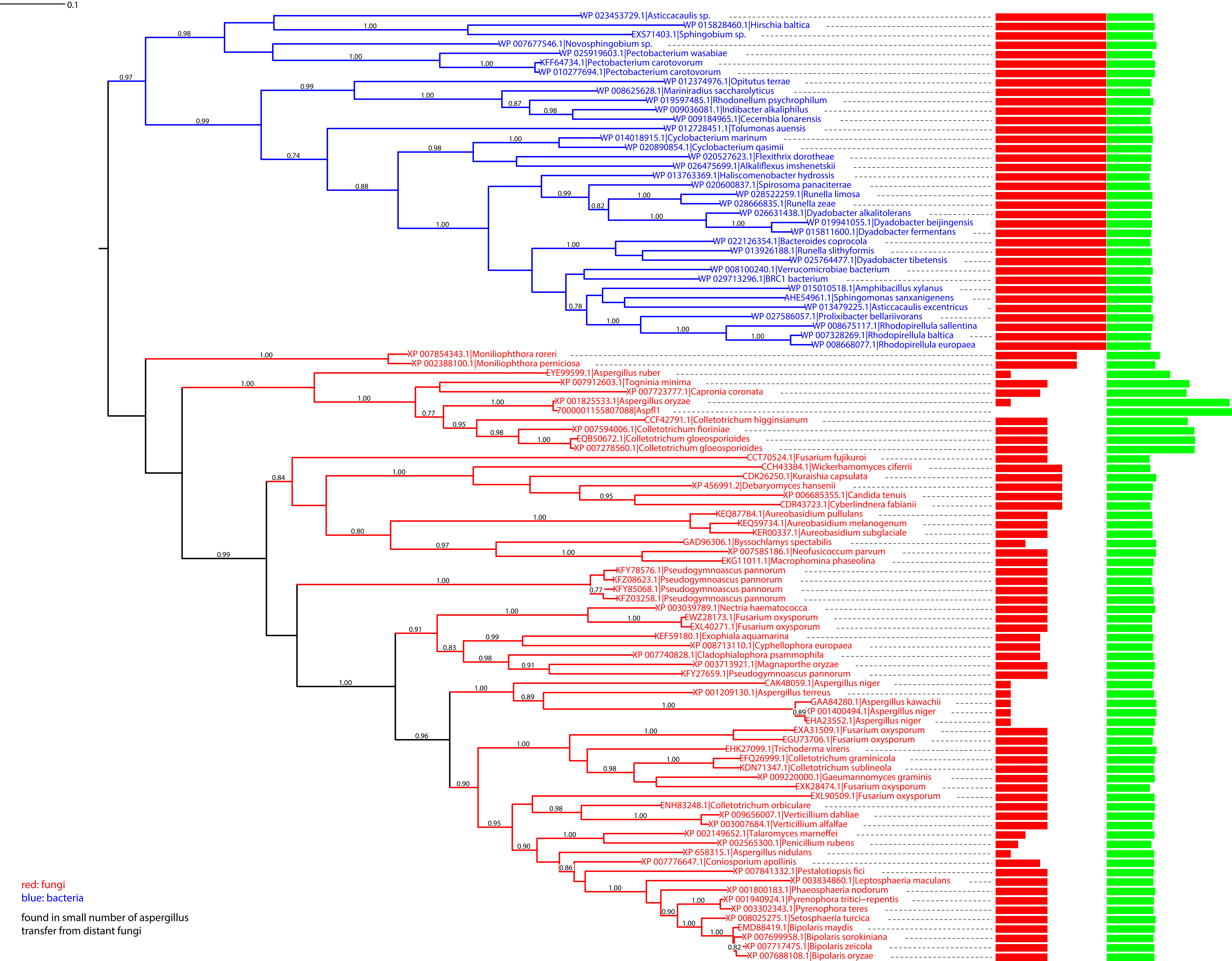

0.1
red: fungi
blue: bacteria
found in small number of aspergillus
transfer from distant fungi

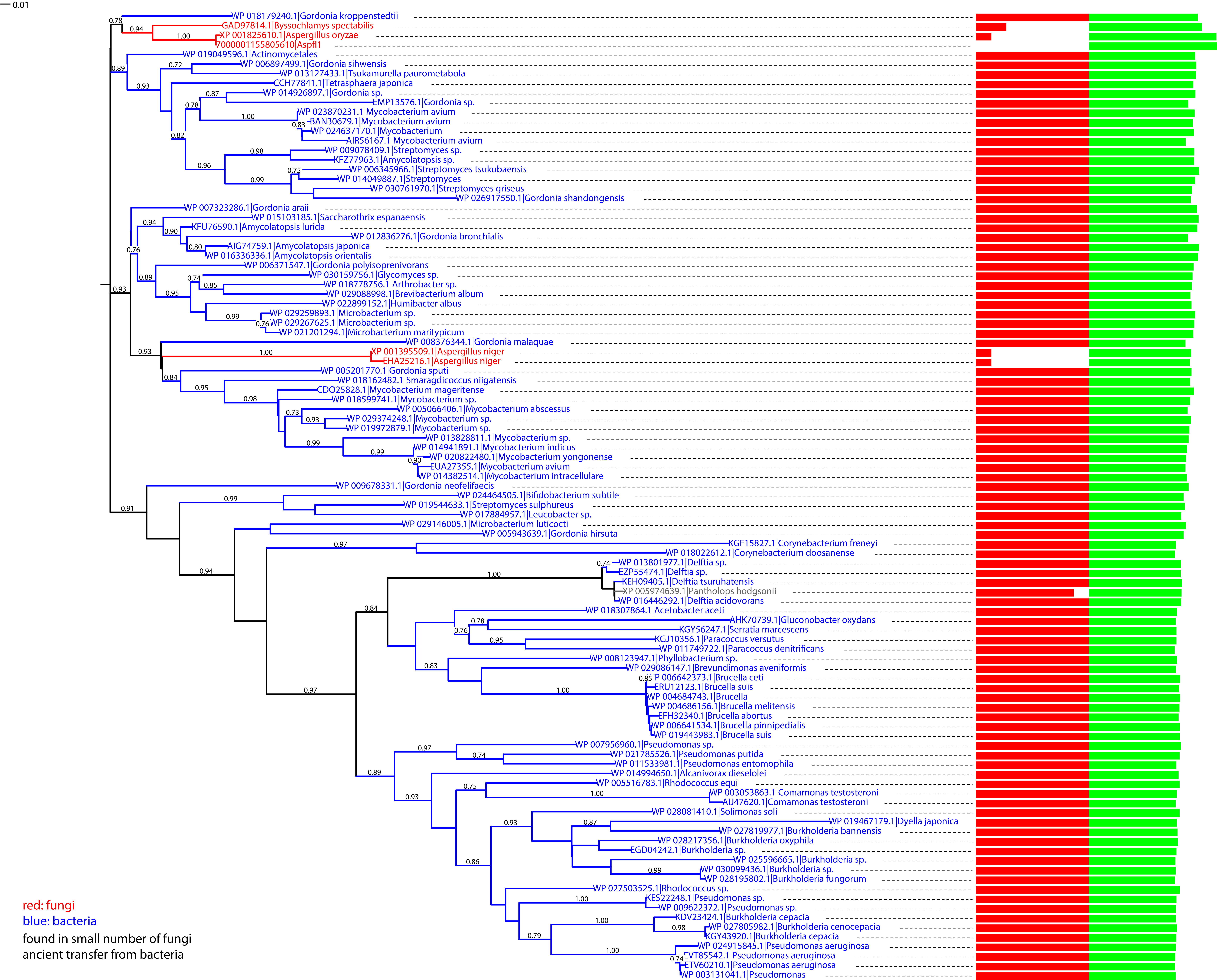
0.01
WP 018179240.1|Gordonia kroppenstedtii
GAD97814.1|Byssochlamys spectabilis
XP 001825610.1|Aspergillus oryzae
7000001155805610|Aspfl1
WP 019049596.1|Actinomycetales
WP 006897499.1|Gordonia sihwensis
WP 013127433.1|Tsukamurella paurometabola
CCH77841.1|Tetrasphaera japonica
WP 014926897.1|Gordonia sp.
EMP13576.1|Gordonia sp.
WP 023870231.1|Mycobacterium avium
BAN30679.1|Mycobacterium avium
WP 024637170.1|Mycobacterium
AIR56167.1|Mycobacterium avium
WP 009078409.1|Streptomyces sp.
KFZ77963.1|Amycolatopsis sp.
WP 006345966.1|Streptomyces tsukubaensis
WP 014049887.1|Streptomyces
WP 030761970.1|Streptomyces griseus
WP 026917550.1|Gordonia shandongensis
WP 007323286.1|Gordonia araii
WP 015103185.1|Saccharothrix espanaensis
KFU76590.1|Amycolatopsis lurida
WP 012836276.1|Gordonia bronchialis
AIG74759.1|Amycolatopsis japonica
WP 016336336.1|Amycolatopsis orientalis
WP 006371547.1|Gordonia polyisoprenivorans
WP 030159756.1|Glycomyces sp.
WP 018778756.1|Arthrobacter sp.
WP 029088998.1|Brevibacterium album
WP 022899152.1|Humibacter albus
WP 029259893.1|Microbacterium sp.
WP 029267625.1|Microbacterium sp.
WP 021201294.1|Microbacterium maritypicum
WP 008376344.1|Gordonia malaquae
XP 001395509.1|Aspergillus niger
EHA25216.1|Aspergillus niger
WP 005201770.1|Gordonia sputi
WP 018162482.1|Smaragdicoccus niigatensis
CDO25828.1|Mycobacterium mageritense
WP 018599741.1|Mycobacterium sp.
WP 005066406.1|Mycobacterium abscessus
WP 029374248.1|Mycobacterium sp.
WP 019972879.1|Mycobacterium sp.
WP 013828811.1|Mycobacterium sp.
WP 014941891.1|Mycobacterium indicus
WP 020822480.1|Mycobacterium yongonense
EUA27355.1|Mycobacterium avium
WP 014382514.1|Mycobacterium intracellulare
WP 009678331.1|Gordonia neofelifaecis
WP 024464505.1|Bifidobacterium subtile
WP 019544633.1|Streptomyces sulphureus
WP 017884957.1|Leucobacter sp.
WP 029146005.1|Microbacterium luticocti
WP 005943639.1|Gordonia hirsuta
KGF15827.1|Corynebacterium freneyi
WP 018022612.1|Corynebacterium doosanense
WP 013801977.1|Delftia sp.
EZP55474.1|Delftia sp.
KEH09405.1|Delftia tsuruhatensis
XP 005974639.1|Pantholops hodgsonii
WP 016446292.1|Delftia acidovorans
WP 018307864.1|Acetobacter aceti
AHK70739.1|Gluconobacter oxydans
KGY56247.1|Serratia marcescens
KGJ10356.1|Paracoccus versutus
WP 011749722.1|Paracoccus denitrificans
WP 008123947.1|Phyllobacterium sp.
WP 029086147.1|Brevundimonas aveniformis
P 006642373.1|Brucella ceti
ERU12123.1|Brucella suis
WP 004684743.1|Brucella
WP 004686156.1|Brucella melitensis
EFH32340.1|Brucella abortus
WP 006641534.1|Brucella pinnipedialis
WP 019443983.1|Brucella suis
WP 007956960.1|Pseudomonas sp.
WP 021785526.1|Pseudomonas putida
WP 011533981.1|Pseudomonas entomophila
WP 014994650.1|Alcanivorax dieselolei
WP 005516783.1|Rhodococcus equi
WP 003053863.1|Comamonas testosteroni
AIJ47620.1|Comamonas testosteroni
WP 028081410.1|Solimonas soli
WP 019467179.1|Dyella japonica
WP 027819977.1|Burkholderia bannensis
WP 028217356.1|Burkholderia oxyphila
EGD04242.1|Burkholderia sp.
WP 025596665.1|Burkholderia sp.
WP 030099436.1|Burkholderia sp.
WP 028195802.1|Burkholderia fungorum
WP 027503525.1|Rhodococcus sp.
KES22248.1|Pseudomonas sp.
WP 009622372.1|Pseudomonas sp.
KDV23424.1|Burkholderia cepacia
WP 027805982.1|Burkholderia cenocepacia
KGY43920.1|Burkholderia cepacia
WP 024915845.1|Pseudomonas aeruginosa
EVT85542.1|Pseudomonas aeruginosa
ETV60210.1|Pseudomonas aeruginosa
WP 003131041.1|Pseudomonas
red: fungi
blue: bacteria
found in small number of fungi
ancient transfer from bacteria

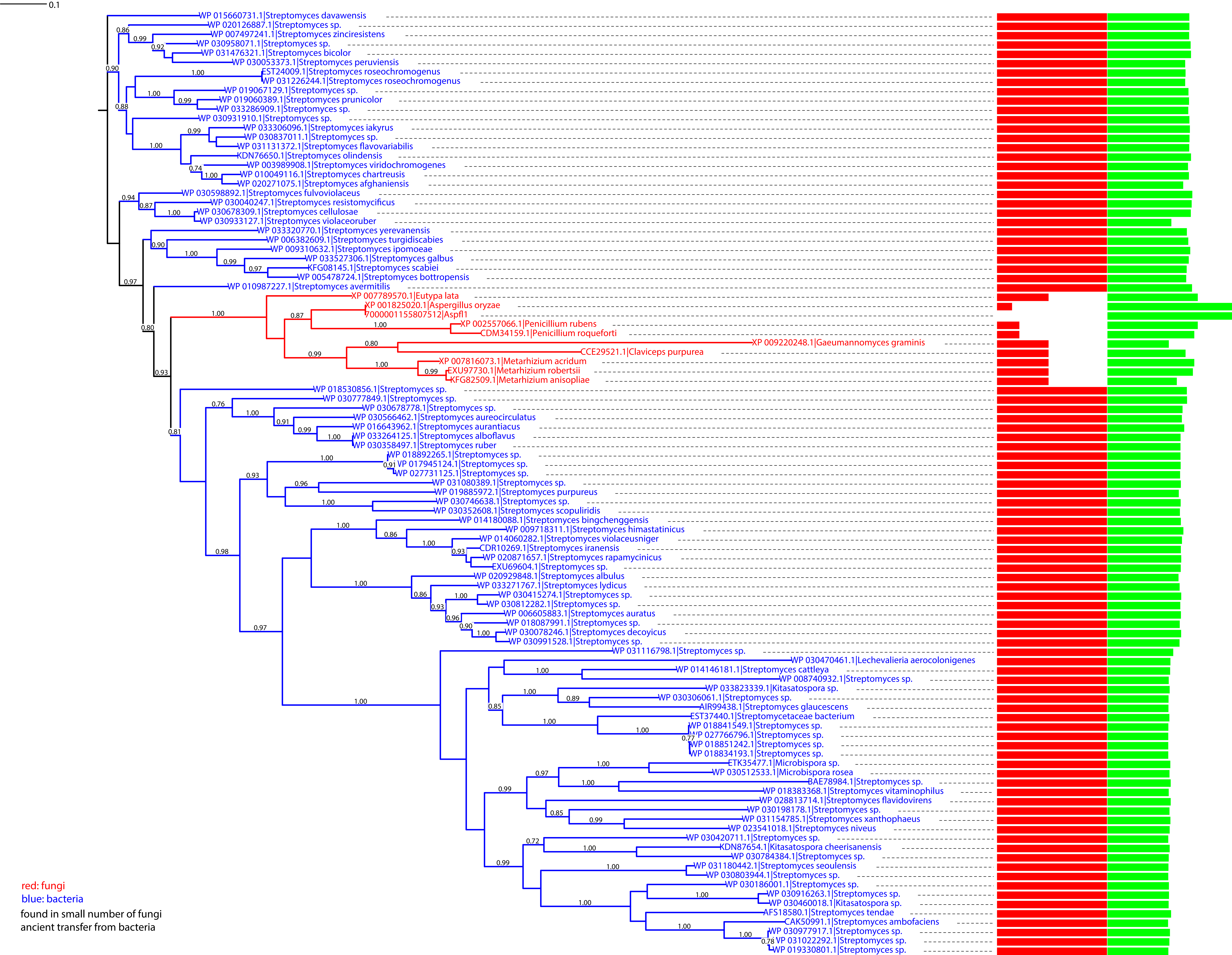
0.1
WP 015660731.1|Streptomyces davawensis
WP 020126887.1|Streptomyces sp.
WP 007497241.1|Streptomyces zincíresistens
WP 030958071.1|Streptomyces sp.
WP 031476321.1|Streptomyces bicolor
WP 030053373.1|Streptomyces peruviensis
EST24009.1|Streptomyces roseochromogenus
WP 031226244.1|Streptomyces roseochromogenus
WP 019067129.1|Streptomyces sp.
WP 019060389.1|Streptomyces prunicolor
WP 033286909.1|Streptomyces sp.
WP 030931910.1|Streptomyces sp.
WP 033306096.1|Streptomyces iakyrus
WP 030837011.1|Streptomyces sp.
WP 031131372.1|Streptomyces flavovariabilis
KDN76650.1|Streptomyces olindensis
WP 003989908.1|Streptomyces viridochromogenes
WP 010049116.1|Streptomyces chartreusis
WP 020271075.1|Streptomyces afghaniensis
WP 030598892.1|Streptomyces fulvoviolaceus
WP 030040247.1|Streptomyces resistomycificus
WP 030678309.1|Streptomyces cellulosae
WP 030933127.1|Streptomyces violaceoruber
WP 033320770.1|Streptomyces yerevanensis
WP 006382609.1|Streptomyces turgidiscabies
WP 009310632.1|Streptomyces ipomoeae
WP 033527306.1|Streptomyces galbus
KFG08145.1|Streptomyces scabiei
WP 005478724.1|Streptomyces bottropensis
WP 010987227.1|Streptomyces avermitilis
XP 007789570.1|Eutypa lata
XP 001825020.1|Aspergillus oryzae
7000001155807512|Aspfl1
XP 002557066.1|Penicillium rubens
CDM34159.1|Penicillium roqueforti
XP 009220248.1|Gaeumannomyces graminis
CCE29521.1|Claviceps purpurea
XP 007816073.1|Metarhizium acridum
EXU97730.1|Metarhizium robertsii
KFG82509.1|Metarhizium anisopliae
WP 018530856.1|Streptomyces sp.
WP 030777849.1|Streptomyces sp.
WP 030678778.1|Streptomyces sp.
WP 030566462.1|Streptomyces aureocirculatus
WP 016643962.1|Streptomyces aurantiacus
WP 033264125.1|Streptomyces alboflavus
WP 030358497.1|Streptomyces ruber
WP 018892265.1|Streptomyces sp.
WP 017945124.1|Streptomyces sp.
WP 027731125.1|Streptomyces sp.
WP 031080389.1|Streptomyces sp.
WP 019885972.1|Streptomyces purpureus
WP 030746638.1|Streptomyces sp.
WP 030352608.1|Streptomyces scopuliridis
WP 014180088.1|Streptomyces bingchenggensis
WP 009718311.1|Streptomyces himastatinicus
WP 014060282.1|Streptomyces violaceusniger
CDR10269.1|Streptomyces iranensis
WP 020871657.1|Streptomyces rapamycinicus
EXU69604.1|Streptomyces sp.
WP 020929848.1|Streptomyces albulus
WP 033271767.1|Streptomyces lydicus
WP 030415274.1|Streptomyces sp.
WP 030812282.1|Streptomyces sp.
WP 006605883.1|Streptomyces auratus
WP 018087991.1|Streptomyces sp.
WP 030078246.1|Streptomyces decoyicus
WP 030991528.1|Streptomyces sp.
WP 031116798.1|Streptomyces sp.
WP 030470461.1|Lechevalieria aerocolonigenes
WP 014146181.1|Streptomyces cattleya
WP 008740932.1|Streptomyces sp.
WP 033823339.1|Kitasatospora sp.
WP 030306061.1|Streptomyces sp.
AIR99438.1|Streptomyces glaucescens
EST37440.1|Streptomycetaceae bacterium
WP 018841549.1|Streptomyces sp.
WP 027766796.1|Streptomyces sp.
WP 018851242.1|Streptomyces sp.
WP 018834193.1|Streptomyces sp.
ETK35477.1|Microbispora sp.
WP 030512533.1|Microbispora rosea
BAE78984.1|Streptomyces sp.
WP 018383368.1|Streptomyces vitaminophilus
WP 028813714.1|Streptomyces flavidovirens
WP 030198178.1|Streptomyces sp.
WP 031154785.1|Streptomyces xanthophaeus
WP 023541018.1|Streptomyces niveus
WP 030420711.1|Streptomyces sp.
KDN87654.1|Kitasatospora cheerisanensis
WP 030784384.1|Streptomyces sp.
WP 031180442.1|Streptomyces seoulensis
WP 030803944.1|Streptomyces sp.
WP 030186001.1|Streptomyces sp.
WP 030916263.1|Streptomyces sp.
WP 030460018.1|Kitasatospora sp.
AFS18580.1|Streptomyces tendae
CAK50991.1|Streptomyces ambofaciens
WP 030977917.1|Streptomyces sp.
WP 031022292.1|Streptomyces sp.
WP 019330801.1|Streptomyces sp.
red: fungi
blue: bacteria
found in small number of fungi
ancient transfer from bacteria

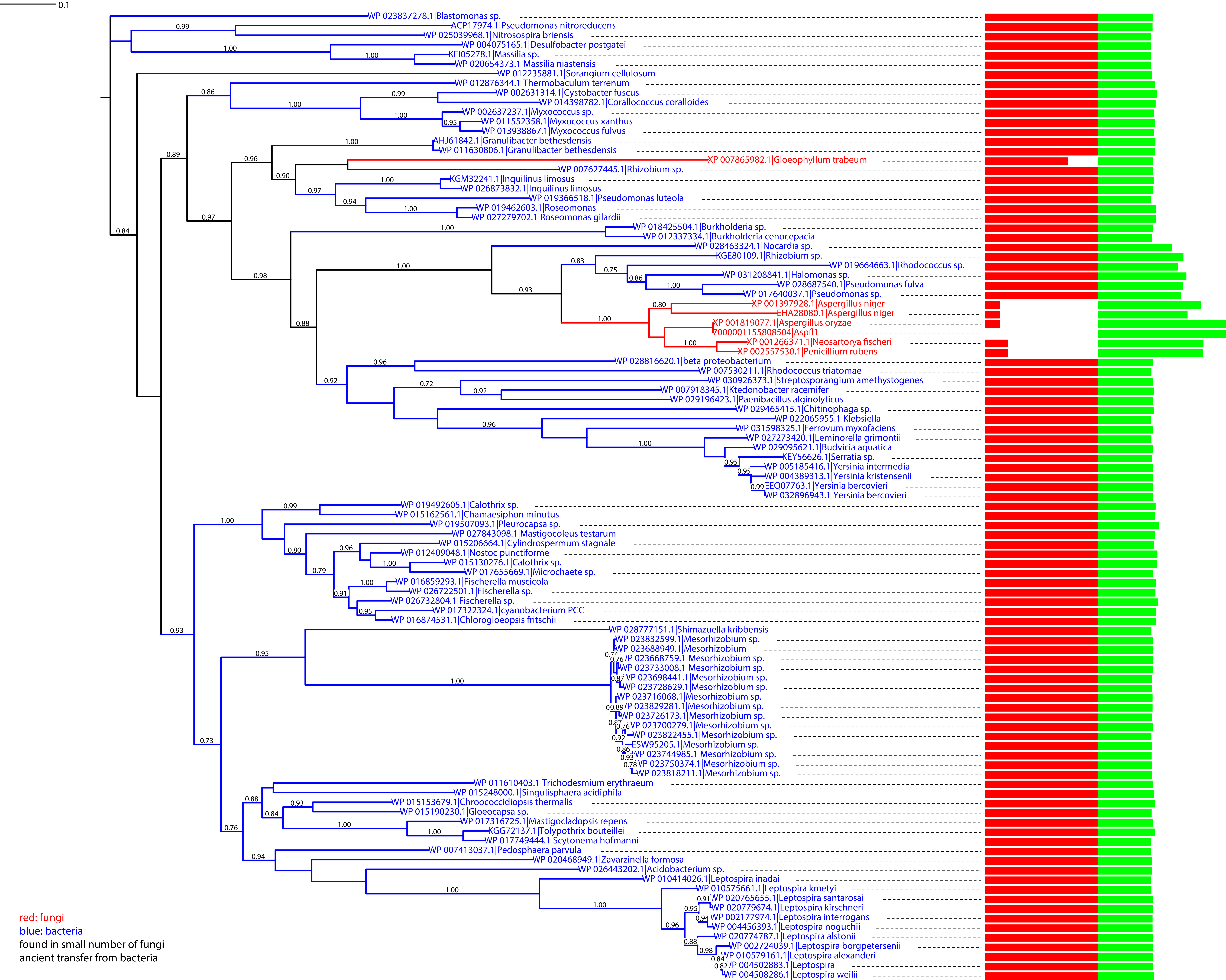

0.1
WP 023837278.1|Blastomonas sp.
ACP17974.1|Pseudomonas nitroreducens
WP 025039968.1|Nitrosospira briensis
WP 004075165.1|Desulfobacter postgatei
KFI05278.1|Massilia sp.
WP 020654373.1|Massilia niastensis
WP 012235881.1|Sorangium cellulosum
WP 012876344.1|Thermobaculum terrenum
WP 002631314.1|Cystobacter fuscus
WP 014398782.1|Corallococcus coralloides
WP 002637237.1|Myxococcus sp.
WP 011552358.1|Myxococcus xanthus
WP 013938867.1|Myxococcus fulvus
AHJ61842.1|Granulibacter bethesdensis
WP 011630806.1|Granulibacter bethesdensis
XP 007865982.1|Gloeophyllum trabeum
WP 007627445.1|Rhizobium sp.
KGM32241.1|Inquilinus limosus
WP 026873832.1|Inquilinus limosus
WP 019366518.1|Pseudomonas luteola
WP 019462603.1|Roseomonas
WP 027279702.1|Roseomonas gilardii
WP 018425504.1|Burkholderia sp.
WP 012337334.1|Burkholderia cenocepacia
WP 028463324.1|Nocardia sp.
KGE80109.1|Rhizobium sp.
WP 019664663.1|Rhodococcus sp.
WP 031208841.1|Halomonas sp.
WP 028687540.1|Pseudomonas fulva
WP 017640037.1|Pseudomonas sp.
XP 001397928.1|Aspergillus niger
EHA28080.1|Aspergillus niger
XP 001819077.1|Aspergillus oryzae
7000001155808504|Aspfl1
XP 001266371.1|Neosartorya fischeri
XP 002557530.1|Penicillium rubens
WP 028816620.1|beta proteobacterium
WP 007530211.1|Rhodococcus triatomae
WP 030926373.1|Streptosporangium amethystogenes
WP 007918345.1|Ktedonobacter racemifer
WP 029196423.1|Paenibacillus alginolyticus
WP 029465415.1|Chitinophaga sp.
WP 022065955.1|Klebsiella
WP 031598325.1|Ferrovum myxofaciens
WP 027273420.1|Leminorella grimontii
WP 029095621.1|Budvicia aquatica
KEY56626.1|Serratia sp.
WP 005185416.1|Yersinia intermedia
WP 004389313.1|Yersinia kristensenii
EEQ07763.1|Yersinia bercovieri
WP 032896943.1|Yersinia bercovieri
WP 019492605.1|Calothrix sp.
WP 015162561.1|Chamaesiphon minutus
WP 019507093.1|Pleurocapsa sp.
WP 027843098.1|Mastigocoleus testarum
WP 015206664.1|Cylindrospermum stagnale
WP 012409048.1|Nostoc punctiforme
WP 015130276.1|Calothrix sp.
WP 017655669.1|Microchaete sp.
WP 016859293.1|Fischerella muscicola
WP 026722501.1|Fischerella sp.
WP 026732804.1|Fischerella sp.
WP 017322324.1|cyanobacterium PCC
WP 016874531.1|Chlorogloeopsis fritschii
WP 028777151.1|Shimazuella kribbensis
WP 023832599.1|Mesorhizobium sp.
WP 023688949.1|Mesorhizobium
WP 023668759.1|Mesorhizobium sp.
WP 023733008.1|Mesorhizobium sp.
WP 023698441.1|Mesorhizobium sp.
WP 023728629.1|Mesorhizobium sp.
WP 023716068.1|Mesorhizobium sp.
WP 023829281.1|Mesorhizobium sp.
WP 023726173.1|Mesorhizobium sp.
WP 023700279.1|Mesorhizobium sp.
WP 023822455.1|Mesorhizobium sp.
ESW95205.1|Mesorhizobium sp.
WP 023744985.1|Mesorhizobium sp.
WP 023750374.1|Mesorhizobium sp.
WP 023818211.1|Mesorhizobium sp.
WP 011610403.1|Trichodesmium erythraeum
WP 015248000.1|Singulisphaera acidiphila
WP 015153679.1|Chroococcidiopsis thermalis
WP 015190230.1|Gloeocapsa sp.
WP 017316725.1|Mastigocladopsis repens
KGG72137.1|Tolypothrix bouteillei
WP 017749444.1|Scytonema hofmanni
WP 007413037.1|Pedosphaera parvula
WP 020468949.1|Zavarzinella formosa
WP 026443202.1|Acidobacterium sp.
WP 010414026.1|Leptospira inadai
WP 010575661.1|Leptospira kmetyi
WP 020765655.1|Leptospira santarosai
WP 020779674.1|Leptospira kirschneri
WP 002177974.1|Leptospira interrogans
WP 004456393.1|Leptospira noguchii
WP 020774787.1|Leptospira alstonii
WP 002724039.1|Leptospira borgpetersenii
WP 010579161.1|Leptospira alexanderi
WP 004502883.1|Leptospira
WP 004508286.1|Leptospira weilii
red: fungi
blue: bacteria
found in small number of fungi
ancient transfer from bacteria

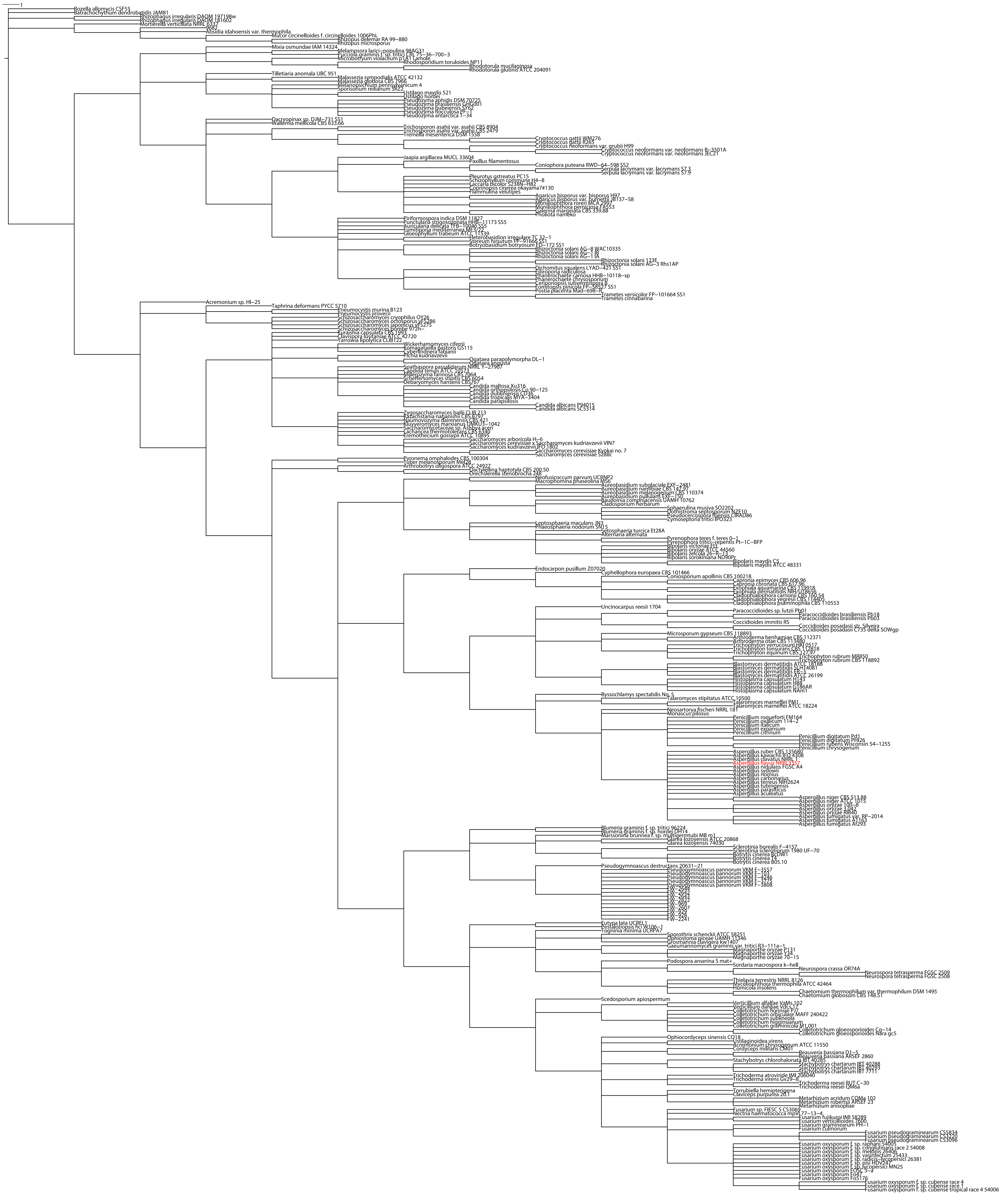
1
Rozella allomycis CSF55
Batrachochytrium dendrobatidis JAM81
Rhizophagus irregularis DAOM 197198w
Rhizophagus irregularis DAOM 181602
Mortierella verticillata NRRL 6337
S682
Absidia idahoensis var. thermophila
Mucor circinelloides f. circinelloides 1006PhL
Rhizopus delemar RA 99−880
Rhizopus microsporus
Mixia osmundae IAM 14324
Melampsora larici−populina 98AG31
Puccinia graminis f. sp. tritici CRL 75−36−700−3
Microbotryum violaceum p1A1 Lamole
Rhodosporidium toruloides NP11
Rhodotorula mucilaginosa
Rhodotorula glutinis ATCC 204091
Tilletiaria anomala UBC 951
Malassezia sympodialis ATCC 42132
Malassezia globosa CBS 7966
Melanopsichium pennsylvanicum 4
Sporisorium reilianum SRZ2
Ustilago maydis 521
Ustilago hordei
Pseudozyma aphidis DSM 70725
Pseudozyma brasiliensis GHG001
Pseudozyma hubeiensis SY62
Pseudozyma flocculosa PF−1
Pseudozyma antarctica T−34
Dacryopinax sp. DJM−731 SS1
Wallemia mellicola CBS 633.66
Trichosporon asahii var. asahii CBS 8904
Trichosporon asahii var. asahii CBS 2479
Tremella mesenterica DSM 1558
Cryptococcus gattii WM276
Cryptococcus gattii R265
Cryptococcus neoformans var. grubii H99
Cryptococcus neoformans var. neoformans B−3501A
Cryptococcus neoformans var. neoformans JEC21
Jaapia argillacea MUCL 33604
Paxillus filamentosus
Coniophora puteana RWD−64−598 SS2
Serpula lacrymans var. lacrymans S7.3
Serpula lacrymans var. lacrymans S7.9
Pleurotus ostreatus PC15
Schizophyllum commune H4−8
Laccaria bicolor S238N−H82
Coprinopsis cinerea okayama7#130
Flammulina velutipes
Agaricus bisporus var. bisporus H97
Agaricus bisporus var. burnettii JB137−S8
Moniliophthora roreri MCA 2997
Moniliophthora perniciosa FA553
Galerina marginata CBS 339.88
Pholiota nameko
Piriformospora indica DSM 11827
Punctularia strigosozonata HHB−11173 SS5
Auricularia delicata TFB−10046 SS5
Fomitiporia mediterranea MF3/22
Gloeophyllum trabeum ATCC 11539
Heterobasidion irregulare TC 32−1
Stereum hirsutum FP−91666 SS1
Botryobasidium botryosum FD−172 SS1
Rhizoctonia solani AG−8 WAC10335
Rhizoctonia solani AG−1 IB
Rhizoctonia solani AG−1 IA
Rhizoctonia solani 123E
Rhizoctonia solani AG−3 Rhs1AP
Dichomitus squalens LYAD−421 SS1
Fibroporia radiculosa
Phanerochaete carnosa HHB−10118−sp
Phanerochaete chrysosporium
Ceriporiopsis subvermispora B
Fomitopsis pinicola FP−58527 SS1
Postia placenta Mad−698−R
Trametes versicolor FP−101664 SS1
Trametes cinnabarina
Acremonium sp. HI−25
Taphrina deformans PYCC 5710
Pneumocystis murina B123
Pneumocystis jirovecii
Schizosaccharomyces cryophilus OY26
Schizosaccharomyces octosporus yFS286
Schizosaccharomyces japonicus yFS275
Schizosaccharomyces pombe 972h−
Kuraishia capsulata CBS 1993
Clavispora lusitaniae ATCC 42720
Yarrowia lipolytica CLIB122
Wickerhamomyces ciferrii
Komagataella pastoris GS115
Cyberlindnera fabianii
Pichia kudriavzevii
Ogataea parapolymorpha DL−1
Ogataea angusta
Spathaspora passalidarum NRRL Y−27907
Candida tenuis ATCC 10573
Millerozyma farinosa CBS 7064
Scheffersomyces stipitis CBS 6054
Debaryomyces hansenii CBS767
Candida maltosa Xu316
Candida orthopsilosis Co 90−125
Candida dubliniensis CD36
Candida tropicalis MYA−3404
Candida parapsilosis
Candida albicans P94015
Candida albicans SC5314
Zygosaccharomyces bailii CLIB 213
Kazachstania naganishii CBS 8797
Naumovozyma dairenensis CBS 421
Kluyveromyces marxianus DMKU3−1042
Saccharomycetaceae sp. Ashbya aceri
Lachancea thermotolerans CBS 6340
Eremothecium gossypii ATCC 10895
Saccharomyces arboricola H−6
Saccharomyces cerevisiae x Saccharomyces kudriavzevii VIN7
Saccharomyces kudriavzevii IFO 1802
Saccharomyces cerevisiae Kyokai no. 7
Saccharomyces cerevisiae S288c
Pyronema omphalodes CBS 100304
Tuber melanosporum Mel28
Arthrobotrys oligospora ATCC 24927
Dactylellina haptotyla CBS 200.50
Drechslerella stenobrocha 248
Neofusicoccum parvum UCRNP2
Macrophomina phaseolina MS6
Aureobasidium subglaciale EXF−2481
Aureobasidium namibiae CBS 147.97
Aureobasidium melanogenum CBS 110374
Aureobasidium pullulans EXF−150
Baudoinia compniacensis UAMH 10762
Cladosporium herbarum
Sphaerulina musiva SO2202
Dothistroma septosporum NZE10
Pseudocercospora fijiensis CIRAD86
Zymoseptoria tritici IPO323
Leptosphaeria maculans JN3
Phaeosphaeria nodorum SN15
Setosphaeria turcica Et28A
Alternaria alternata
Pyrenophora teres f. teres 0−1
Pyrenophora tritici−repentis Pt−1C−BFP
Bipolaris victoriae FI3
Bipolaris oryzae ATCC 44560
Bipolaris zeicola 26−R−13
Bipolaris sorokiniana ND90Pr
Bipolaris maydis C5
Bipolaris maydis ATCC 48331
Endocarpon pusillum Z07020
Cyphellophora europaea CBS 101466
Coniosporium apollinis CBS 100218
Capronia epimyces CBS 606.96
Capronia coronata CBS 617.96
Exophiala aquamarina CBS 119918
Exophiala dermatitidis NIH/UT8656
Cladophialophora carrionii CBS 160.54
Cladophialophora yegresii CBS 114405
Cladophialophora psammophila CBS 110553
Uncinocarpus reesii 1704
Paracoccidioides sp. lutzii Pb01
Paracoccidioides brasiliensis Pb18
Paracoccidioides brasiliensis Pb03
Coccidioides immitis RS
Coccidioides posadasii str. Silveira
Coccidioides posadasii C735 delta SOWgp
Microsporum gypseum CBS 118893
Arthroderma benhamiae CBS 112371
Arthroderma otae CBS 113480
Trichophyton verrucosum HKI 0517
Trichophyton tonsurans CBS 112818
Trichophyton equinum CBS 127.97
Trichophyton rubrum MR850
Trichophyton rubrum CBS 118892
Blastomyces dermatitidis ATCC 18188
Blastomyces dermatitidis SLH14081
Blastomyces dermatitidis ER−3
Blastomyces dermatitidis ATCC 26199
Histoplasma capsulatum H143
Histoplasma capsulatum H88
Histoplasma capsulatum G186AR
Histoplasma capsulatum NAm1
Byssochlamys spectabilis No. 5
Talaromyces stipitatus ATCC 10500
Talaromyces marneffei PM1
Talaromyces marneffei ATCC 18224
Neosartorya fischeri NRRL 181
Monascus pilosus
Penicillium roqueforti FM164
Penicillium oxalicum 114−2
Penicillium italicum
Penicillium expansum
Penicillium citrinum
Penicillium digitatum Pd1
Penicillium digitatum PHI26
Penicillium rubens Wisconsin 54−1255
Penicillium chrysogenum
Aspergillus ruber CBS 135680
Aspergillus kawachii IFO 4308
Aspergillus clavatus NRRL 1
Aspergillus flavus NRRL 3357
Aspergillus nidulans FGSC A4
Aspergillus sydowii
Aspergillus nomius
Aspergillus carbonarius
Aspergillus terreus NIH2624
Aspergillus tubingensis
Aspergillus parasiticus
Aspergillus aculeatus
Aspergillus niger CBS 513.88
Aspergillus niger ATCC 1015
Aspergillus oryzae 100−8
Aspergillus oryzae 3.042
Aspergillus oryzae RIB40
Aspergillus fumigatus var. RP−2014
Aspergillus fumigatus A1163
Aspergillus fumigatus Af293
Blumeria graminis f. sp. tritici 96224
Blumeria graminis f. sp. hordei DH14
Marssonina brunnea f. sp. multigermtubi MB m1
Glarea lozoyensis ATCC 20868
Glarea lozoyensis 74030
Sclerotinia borealis F−4157
Sclerotinia sclerotiorum 1980 UF−70
Botrytis cinerea BcDW1
Botrytis cinerea T4
Botrytis cinerea B05.10
Pseudogymnoascus destructans 20631−21
Pseudogymnoascus pannorum VKM F−3557
Pseudogymnoascus pannorum VKM F−103
Pseudogymnoascus pannorum VKM F−4246
Pseudogymnoascus pannorum VKM F−3775
Pseudogymnoascus pannorum VKM F−3808
FW−2642
FW−2643
FW−2645
FW−2822
FW−969
FW−2607
FW−929
FW−928
FW−2241
Eutypa lata UCREL1
Pestalotiopsis fici W106−1
Togninia minima UCRPA7
Sporothrix schenckii ATCC 58251
Ophiostoma piceae UAMH 11346
Grosmannia clavigera kw1407
Gaeumannomyces graminis var. tritici R3−111a−1
Magnaporthe oryzae P131
Magnaporthe oryzae Y34
Magnaporthe oryzae 70−15
Podospora anserina S mat+
Sordaria macrospora k−hell
Neurospora crassa OR74A
Neurospora tetrasperma FGSC 2509
Neurospora tetrasperma FGSC 2508
Thielavia terrestris NRRL 8126
Myceliophthora thermophila ATCC 42464
Humicola insolens
Chaetomium thermophilum var. thermophilum DSM 1495
Chaetomium globosum CBS 148.51
Scedosporium apiospermum
Verticillium alfalfae VaMs.102
Verticillium dahliae VdLs.17
Colletotrichum fioriniae PJ7
Colletotrichum orbiculare MAFF 240422
Colletotrichum higginsianum
Colletotrichum sublineola
Colletotrichum graminicola M1.001
Colletotrichum gloeosporioides Cg−14
Colletotrichum gloeosporioides Nara gc5
Ophiocordyceps sinensis CO18
Ustilaginoidea virens
Acremonium chrysogenum ATCC 11550
Cordyceps militaris CM01
Beauveria bassiana D1−5
Beauveria bassiana ARSEF 2860
Stachybotrys chlorohalonata IBT 40285
Stachybotrys chartarum IBT 40288
Stachybotrys chartarum IBT 40293
Stachybotrys chartarum IBT 7711
Trichoderma atroviride IMI 206040
Trichoderma virens Gv29−8
Trichoderma reesei RUT C−30
Trichoderma reesei QM6a
Torrubiella hemipterigena
Claviceps purpurea 20.1
Metarhizium acridum CQMa 102
Metarhizium robertsii ARSEF 23
Metarhizium anisopliae
Fusarium sp. FIESC 5 CS3069
Nectria haematococca mpVI 77−13−4
Fusarium fujikuroi IMI 58289
Fusarium verticillioides 7600
Fusarium graminearum PH−1
Fusarium culmorum
Fusarium pseudograminearum CS5834
Fusarium pseudograminearum CS3220
Fusarium pseudograminearum CS3096
Fusarium oxysporum f. sp. raphani 54005
Fusarium oxysporum f. sp. conglutinans race 2 54008
Fusarium oxysporum f. sp. melonis 26406
Fusarium oxysporum f. sp. vasinfectum 25433
Fusarium oxysporum f. sp. radicis−lycopersici 26381
Fusarium oxysporum f. sp. pisi HDV247
Fusarium oxysporum f. sp. lycopersici MN25
Fusarium oxysporum FOSC 3−a
Fusarium oxysporum Fo47
Fusarium oxysporum Fo5176
Fusarium oxysporum f. sp. cubense race 4
Fusarium oxysporum f. sp. cubense race 1
Fusarium oxysporum f. sp. cubense tropical race 4 54006
